# A Model Ensemble Approach Enables Data-Driven Property Prediction for Chemically Deconstructable Thermosets in the Low-Data Regime

**DOI:** 10.1021/acscentsci.3c00502

**Published:** 2023-09-14

**Authors:** Yasmeen S. AlFaraj, Somesh Mohapatra, Peyton Shieh, Keith E. L. Husted, Douglass G. Ivanoff, Evan M. Lloyd, Julian C. Cooper, Yutong Dai, Avni P. Singhal, Jeffrey S. Moore, Nancy R. Sottos, Rafael Gomez-Bombarelli, Jeremiah A. Johnson

**Affiliations:** † Department of Chemistry, Massachusetts Institute of Technology, Cambridge, Massachusetts 02139, United States of America; ‡ Department of Materials Science and Engineering, Massachusetts Institute of Technology, Cambridge, Massachusetts 02139, United States of America; § Department of Materials Science and Engineering, University of Illinois at Urbana—Champaign, Urbana, Illinois 61801, United States of America; ∥ The Beckman Institute for Advanced Science and Technology, University of Illinois at Urbana—Champaign, Urbana, Illinois 61801, United States of America; ⊥ Department of Chemistry, University of Illinois at Urbana—Champaign, Urbana, Illinois 61801, United States of America

## Abstract

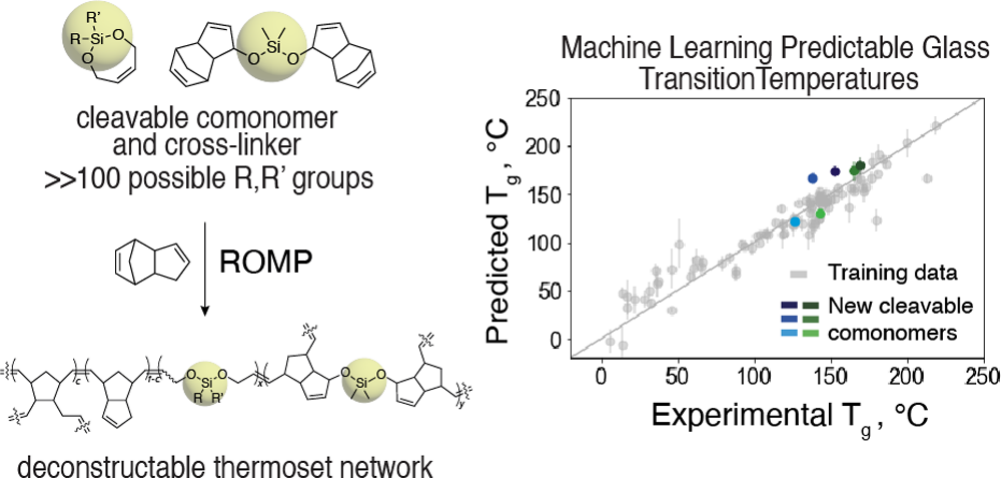

Thermosets present sustainability challenges that could potentially be addressed through the design of deconstructable variants with tunable properties; however, the combinatorial space of possible thermoset molecular building blocks (e.g., monomers, cross-linkers, and additives) and manufacturing conditions is vast, and predictive knowledge for how combinations of these molecular components translate to bulk thermoset properties is lacking. Data science could overcome these problems, but computational methods are difficult to apply to multicomponent, amorphous, statistical copolymer materials for which little data exist. Here, leveraging a data set with 101 examples, we introduce a closed-loop experimental, machine learning (ML), and virtual screening strategy to enable predictions of the glass transition temperature (*T*_g_) of polydicyclopentadiene (pDCPD) thermosets containing cleavable bifunctional silyl ether (BSE) comonomers and/or cross-linkers with varied compositions and loadings. Molecular features and formulation variables are used as model inputs, and uncertainty is quantified through model ensembling, which together with heavy regularization helps to avoid overfitting and ultimately achieves predictions within <15 °C for thermosets with compositionally diverse BSEs. This work offers a path to predicting the properties of thermosets based on their molecular building blocks, which may accelerate the discovery of promising plastics, rubbers, and composites with improved functionality and controlled deconstructability.

## Abstract 2

A machine learning model that predicts *T*_g_ for deconstructable thermosets with varied bifunctional silyl ether comonomer loadings and structures is introduced and experimentally validated.

## Introduction

Materials discovery is often cited as a bottleneck in addressing time-critical challenges in medicine, sustainable chemistry, and engineering design.^1−3^ Even when a class of materials is found to be promising for a given application, traditional experimental approaches for screening can be arduous, costly, and inefficient for zeroing in on a desired combination of properties. Recent computational advances in molecular representations, machine learning architectures like graph neural networks,^1,4,5^ and high-throughput virtual screening have enabled data-driven materials discovery in specific materials contexts (e.g., for molecular crystals, organic light-emitting diodes,^6^ or zeolite catalysts);^7−10^ however, these methods remain difficult to apply to other common classes of materials such as compositionally complex, amorphous polymers and polymer networks. For example, while curated databases like PoLyInfo,^11^ Polymer Genome,^12^ or the PI1M library^13^ have allowed researchers to train machine learning models to predict the glass transition temperature (*T*_g_),^14−16^ thermal conductivity,^17^ dielectric constant,^18^ crystallization tendency,^19^ and bandgap^20^ for linear homopolymers (i.e., polymers of a single composition) and some copolymers,^21−25^ there is little curated data available for three-dimensional (co)polymers such as thermosets.^26−29^ Representing the chemical and topological diversity of thermosets in a way that enables extrapolation to new (co)monomers and cross-linking patterns is a challenge to ML strategies that are typically overfit to known monomer chemistries and linear architectures.

Thermosets are covalently cross-linked, three-dimensional polymer networks that irreversibly adopt a given shape upon curing. These materials make up ∼20% of manufactured polymers today, and they are often designed for high-temperature, chemically harsh, and/or mechanically extreme environments, e.g., in the aerospace industry, automotive design, renewable energy, and marine applications.^30−32^ Thus, by their very nature, they are difficult to chemically deconstruct; most are mechanically downcycled, incinerated, or disposed of in landfills, where they persist for decades/centuries. The strategic introduction of cleavable bonds at the time of manufacturing could enable the production of deconstructable thermosets with properties on par with existing materials yet new end-of-life options.^33−40^

**Figure 1:**
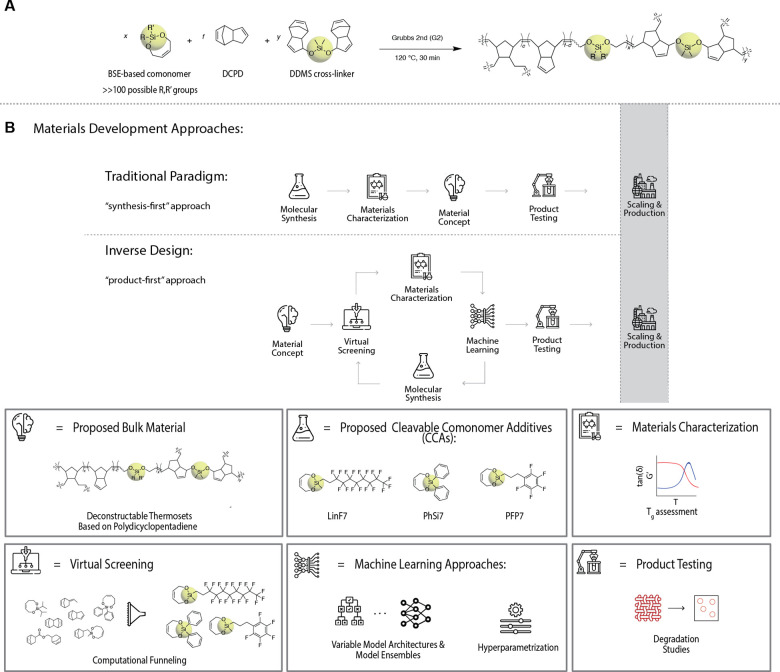
Overview of experimental synthesis–machine learning–virtual screening loop enabling the identification of new cleavable comonomer additives for pDCPD. (A) Scheme for the experimental synthesis of bifunctional silyl ether (BSE)-based cleavable comonomers with DCPD and cleavable cross-linker **DDMS**. (B) Comparison between the traditional paradigm for materials discovery, which relies on a synthesis-first approach, and proposed paradigms for accelerated materials discovery, which rely on an inverse-design and “product-first” approach. Specifics of this work to the proposed discovery paradigm are further clarified in the legend. Materials concept: synthesis of deconstructable thermosets. Molecular synthesis: specifying proposed cleavable comonomers. Machine learning: specifies deep learning and model ensemble architecture and hyperparameter determination. Virtual screening: determination of cleavable comonomers resulting in desirable bulk material properties from a library of possible cleavable comonomers as determined by precursor commercial availability. Materials characterization and product testing: validation of experimentally obtained glass transition temperatures, *T*_g_, of resulting deconstructable thermosets against predicted values and performance of deconstruction studies for these materials.

We have leveraged cleavable comonomers containing bifunctional silyl ethers (BSEs) as additives to facilitate the manufacturing of deconstructable variants of the industrial thermoset polydicyclopentadiene (pDCPD) (Figure 1A).^41−47^ BSEs are versatile cleavable bonds for deconstructable thermosets, as they are easy to synthesize from low-cost components (e.g., diols and dichlorosilanes), they feature thermally and oxidatively stable Si–O bonds, and they have diverse mechanisms for selective Si–O bond cleavage.^42−46,48^ Moreover, the properties of BSEs are closely linked to their Si substituents, of which there are hundreds of possible combinations that are synthesizable from commercially available reagents (e.g., a search of the keyword “dichlorosilane” on the Gelest web-catalog returns >100 unique products). This advantage also raises key challenges: it is impractical to synthesize and experimentally test all possible BSEs and their combinations experimentally, and it is difficult to predict through intuition alone how BSE comonomer structures and loadings will impact the properties of thermosets.

Here, we propose a closed-loop strategy to overcome the challenges associated with applying data science to thermosets in the low-data regime. In particular, we focus on mapping the choice of BSE-based comonomer and/or cross-linker to *T*_g_, a critical property of thermosets that determines their operating temperature range. Our strategy addresses two main challenges: (i) the unavailability of structure-based representations, by representing only the chemistry of the precursors, and (ii) the scarcity of data, by training heavily regularized model ensembles that enable uncertainty quantification and less noisy predictions.

Linear homopolymers or ordered copolymers have well-defined chemical structures and hence can be represented through an ordered sequence of per-monomer chemical features, or through graph convolutional neural networks acting on the whole repeat unit.^49^ 3D stochastic polymers, however, cannot be easily represented as the numerical vectors that ML models require as inputs. Recently, hierarchical ML models have been applied to tackle the representation of 3D networks of known structure or stochastic linear polymers, but no tools are available for polymers of complex, stochastic, and unknown topologies.^50−53^ Our approach relies on representing the individual chemical precursors as small molecules through low-dimensional physicochemical features, such as hydrophobicity and partial charges. Doing so captures domain-specific heuristics, lowers the dimensionality of the chemical input space, and most importantly avoids the need to represent the unknown network topology of the polymer. We also utilize model ensembling, that is, using the consensus prediction from multiple models instead of single prediction, to increase the transferability of our predictions to inputs away from the training data.^47^ We combine ensembling over random seeds for model initialization, over data subsets (bagging, boosting), and over hyperparameter choices, i.e., varying the overall architecture of the models themselves, combining different classes of regressors from linear models to decision trees and support vector machines (Figure 1B).^54^ A key advantage of model ensembles, which are widely used in the general ML literature but less so in the predictive design of three-dimensional polymer networks, is that each individual model’s prediction tends to differ when predicting far from the training data, so their variance can be used as a measure of uncertainty and their mean will provide higher accuracy and more robustness than any particular member of the ensemble.^55−58^

We show that our resulting model, which is trained on only 101 data points (i.e., the “low data regime”) taken from the literature and results from our combined laboratories, enables predictions of how BSE-based cleavable comonomer and cross-linker loadings and Si substituents impact the *T*_g_ of deconstructable pDCPD thermosets within ±15 °C experimental accuracy across a wide temperature range (0–220 °C). Thus, this work provides a route to predict the impacts of cleavable comonomer/cross-linker composition and loading on the *T*_g_ of deconstructable pDCPD thermosets and outlines a framework for predictive (co)polymer and polymer network design in the low-data regime.

## Results and Discussion

### Data Collection for the Machine Learning Model

**Figure 2:**
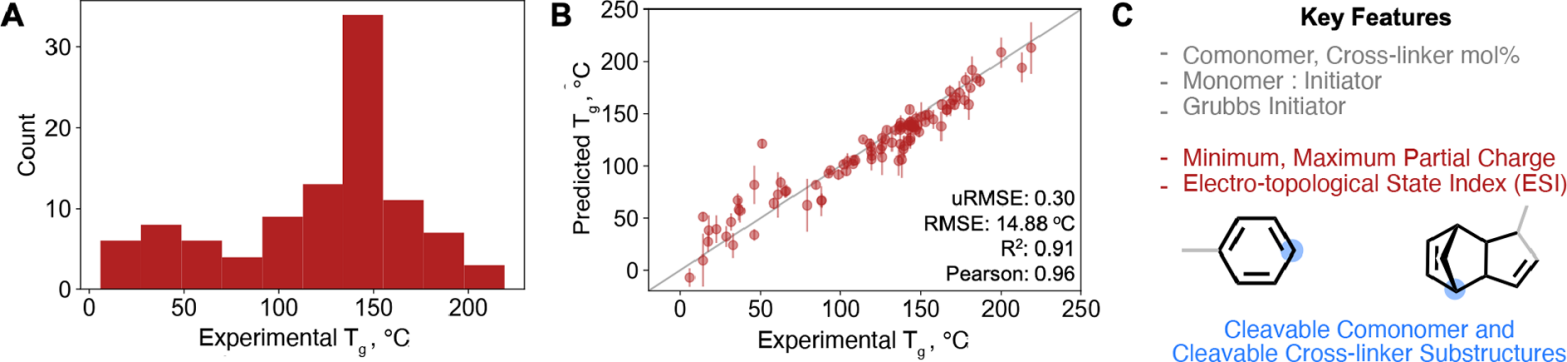
Model ensemble predicts *T*_g_ with high fidelity and highlights key input variables for prediction of *T*_g_. (A) Distribution of *T*_g_ for 101 data points in the training data set. (B) Parity plot for fingerprint-based model shows good agreement of predicted and experimental *T*_g_ for the held-out test data set. Predictions for the test data set are based on an ensemble of different models, and mean and standard deviation of the different predictions are reported. The inset text notes the model ensemble performance metrics. Error bars note the standard deviation of the predicted *T*_g_ values using the different models in the ensemble. The *y* = *x* line in the figure is meant to be used as the parity line to ascertain the difference between the predicted and the experimental *T*_g_ values. If the dot is on the line, then the predicted *T*_g_ is the same as the experimental *T*_g_; if it is off the line, then it is under/overpredicted. (C) Key features identified by fingerprint- and descriptor-based models are shown. Features common to both models are colored gray, descriptors are colored red, and substructures identified for the cleavable comonomer and cross-linker have a blue label. The node of the substructure is denoted by a blue dot; bonds in the atomic neighborhood, for the respective substructure, are colored black; and remaining bonds are colored gray.

Robust *T*_g_ prediction for deconstructable pDCPD requires a training data set with information on the chemical compositions of cleavable comonomers and cross-linkers as well as the thermoset curing parameters. To build an appropriate training data set, we collected data from existing literature examples and in-house experiments (Figure 2A, Figures S1–S87, Tables S1–S3),^59−66^ providing 101 different combinations of cleavable additives (9 BSE-based and 1 acetal-based), ratios of comonomers and/or cross-linkers to the curing initiator, and the initiator type (first- or second-generation Grubbs catalyst), along with their experimentally measured *T*_g_ values obtained from either differential scanning calorimetry (DSC) or estimated by dynamic mechanical analysis (DMA) after curing. For samples that displayed multiple tan(δ) peaks, which occurred in some cases at high comonomer loading or with non-BSE-based monomers (i.e., acetal-based), we used the global maximum of tan(δ) as the *T*_g_ for training our model.^43,46^

### Machine Learning Model Ensemble Architecture for Low-Data Regime Prediction

Machine learning models for materials design are often trained to map synthesis parameters and affordable, high-information descriptors, which can be obtained for materials before they are synthesized, to measured experimental target properties.^67,68^ Because important synthetic details for polymers are missing in publicly available data sets such as PoLyInfo,^11^ data-driven prediction models struggle to reach high accuracy despite the availability of over 5000 data points. For example, a recent review benchmarking machine learning models to predict *T*_g_ values for homopolymers reported an accuracy of only ±39 °C.^14^ Ramprasad and co-workers recently reported an error of 29 °C when combining a total of 9500 homopolymer and copolymer data points, combining data ensembles and a metalearner model using only chemical formulas as inputs.^21,57^ Moreover, limitations in materials data accessibility often result in lower predictive accuracy as a result of overfitting to the available training data. For example, Hu et al. applied a materials genome strategy to control the mechanical properties of cross-linked epoxies. This approach requires the definition of “gene” substructures of interest (epoxides and amines in this case) and uses a graph convolution network to extrapolate information about these small molecules. Subsequently, transfer learning was employed alongside polymer descriptors including molecular weight and an estimated cross-linking density to predict the properties of the bulk material. They provided experimental validation of one prediction and molecular dynamics simulations of additional ones. The models struggled with overfitting and with extrapolation due to high performance metrics, with both ML predictions and validations being close to the mean performance of the training data.^69^

In this work, we instead utilize model ensembles across data splits and architectural choices to achieve robust predictions for thermoset copolymers in the low-data regime using chemical features such as physicochemical descriptors, as well as structural features based on molecular connectivity, as inputs. The model ensemble has the following independent variables: cleavable BSE comonomer and cross-linker representations and respective concentrations in mol %, monomer to initiator ratio, and initiator type. The cleavable comonomers were represented as extended connectivity fingerprints, which encode the chemical substructures in a barcode-like representation unique to each molecule.^70−72^ This approach captures both the common and unique attributes of the individual molecules. The cleavable comonomer mol % and monomer to initiator ratios were represented as numbers, while the initiator type was treated as a categorical variable. Another model was trained with the cleavable comonomers represented with physicochemical descriptors, such as electro-topological state indices (ESI), partial charge, number of carbocycles and saturated rings, to benchmark the fingerprint representations (see Supporting Information Figures S88 and S89, Table S1).

As noted above, many machine learning models require large data sets—often more than 1000 data points—which poses a major challenge in materials research.^73,74^ Cross-validation and data-ensembling are typically used to help overfitting to a singular section of the data set in the process.^74^ To enable the robust prediction of *T*_g_ for pDCPD thermosets in the low-data regime, we used a model ensemble consisting of multiple models with different architectures and hyperparameters, i.e., parameters that define the model architecture and cannot be estimated from the data. The model ensemble consisted of 5 hyperparameter-optimized model architectures, each trained on 10 random splits of the data set, for a total of 50 individual models. For each model, 60% of the data were used for training, 20% were used to validate the model during training, and 20% were held-out to test the model ensemble after training.^75−79^ The top 5 model architectures were chosen based on the least root mean squared error (RMSE) on the test data set after running a similar experiment for more than 10 model architectures, including Gaussian process regression, decision trees, linear models, and multilayer perceptrons. For the final model ensemble, the RMSE of the test set was 0.30 of the standard deviation of the training data, or 14.9 °C (Figure 2B). It is noteworthy that the model exhibited lower error and uncertainty around 150 °C, which is an expected feature, as the glass transition of deconstructable pDCPD thermosets in the training set primarily exhibit *T*_g_ values close to this value. These results suggest that the model ensemble was able to balance the bias-variance trade-off, with a mixture of underfitting high bias and low variance simpler models and overfitting low bias and high variance complex models. By focusing on one class of highly diverse compounds, our approach achieves lower error than large models trained on thousands of data points across homo- and copolymer space, which have errors closer to 30 °C or higher.^14,21^

In addition to the fingerprint representation for the cleavable comonomers and cross-linkers, we benchmarked the descriptor representation. The descriptor-based model ensemble performed slightly worse than fingerprint-based models, with test set RMSE of 0.35 of the standard deviation of the training data, or 17.5 °C (see Supporting Information Figure S90). The fingerprint-based models depend on the substructures and their implicit influence on the chemical properties of the molecules to map the molecules to their properties, while the descriptor-based models explicitly provide information about the different chemical properties of the molecules in the model. The fingerprint representation could be easily obtained for new molecules; however, descriptors might not be readily available and may require experiments or quantum chemical calculations. Additionally, the hand-crafted list of descriptors might not always include all possible features that affect the property of interest, while fingerprints do not have such a problem.

To understand the decision-making process of the model ensemble, we analyzed the importance of input features for prediction of *T*_g_ in both the fingerprint- and descriptor-based models through so-called attribution techniques (Figure 2C, see Supporting Information Figures S88 and S89). Cleavable comonomer mol %, initiator type, and monomer to initiator ratios were observed to be important across both models. The importance attributed to these features is in line with known experimental observations that high concentrations of BSE-based cleavable comonomers lead to decreases in *T*_g_. The fingerprint-based models associated higher *T*_g_ with aryl substituents and norbornene-based cross-linkers. We interpret this prediction to the possibility that aryl substituents may induce stronger noncovalent interactions between cleavable comonomers, while the norbornene-based cross-linkers introduce rigid, inflexible structures that decrease the free volume. The descriptor-based models, which rely on physicochemical features rather than molecular graphs, highlighted the number of aliphatic and aryl rings as being critical for *T*_g_. The Electrotopological State Index, a combined structure and electronic descriptor commonly used in pharmaceuticals,^77^ and partial charges were also noted as key features, indicating the influence of the overall molecular topology and electronic charge distribution on *T*_g_. These observations help in elucidating design principles for cleavable comonomers in high-*T*_g_ pDCPD thermosets.

### *T*_g_ Prediction for Mixtures of BSE-Based Cleavable Comonomers and Cross-Linkers

**Figure 3:**
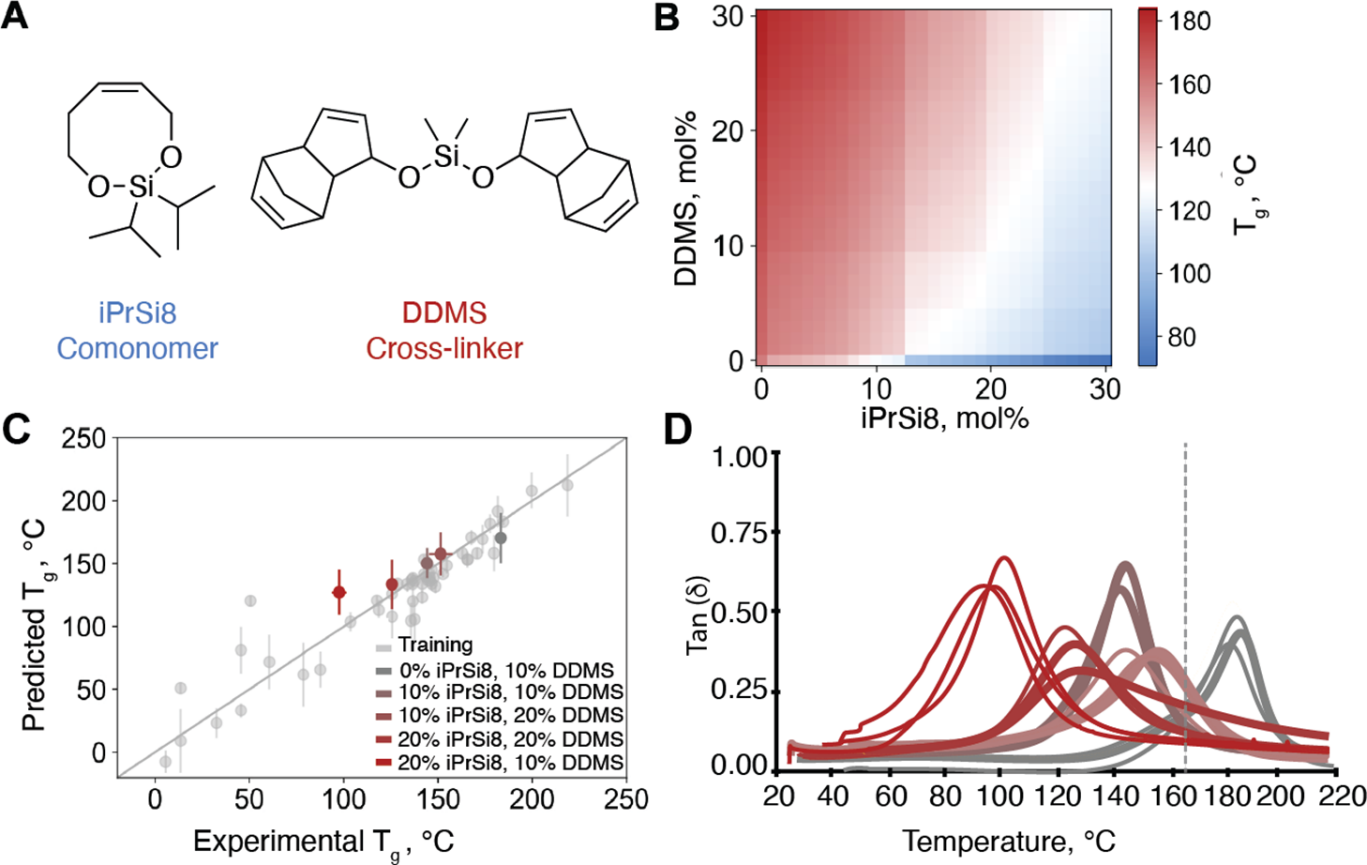
Model ensemble successfully captures the variation in *T*_g_ in a titration study with a previously reported **iPrSi8** comonomer and **DDMS** cross-linker. (A) Chemical structures of **iPrSi8** comonomer and **DDMS** cross-linker used in the mol % sweep experiment. (B) 2D plot of predicted *T*_g_ values for different ratios of **iPrSi8** (cleavable comonomer) and **DDMS** (cleavable cross-linker) shows the decreasing *T*_g_ with the increasing comonomer mol % and decreasing cross-linker mol %. The sharp boundaries in the plot are a consequence of limited data around the respective compositions. (C) Parity plot showing the reasonable agreement of experimental and predicted *T*_g_ values for the five selected compositions of **iPrSi8** and **DDMS**. Vertical error bars note standard deviation of the *T*_g_ predicted using the different models in the ensemble, and horizontal error bars note the standard deviation across the experimental replicates. The *y* = *x* line in the figure is meant to be used as the parity line to ascertain the difference between the predicted and experimental *T*_g_ values. If the dot is on the line, then the predicted *T*_g_ is same as the experimental *T*_g_; if it is off the line, then it is under/overpredicted. (D) Dynamic mechanical analysis (DMA) temperature sweeps of **iPrSi8** and **DDMS**-containing pDCPD thermosets at variable mol % with *T*_g_ as the peak of tan(δ). Color follows the legend of panel C. The dashed line shows the *T*_g_ of virgin pDCPD.

We previously showed that treatment of **iPrSi**-based pDCPD thermosets with a fluoride or acid source can facilitate deconstruction of the thermoset to soluble products, while networks containing cleavable cross-linkers show a decline in cross-linking density without solubilization under similar cleavage conditions (Figure 3A).^43^ Here, we first assessed our model ensemble’s performance using mixtures of **iPrSi8**, which was present in the training set at different mol % values, and the cleavable cross-linker didicyclopentadiene methyl silyl ether (**DDMS**), which was assessed at a fixed loading (10 mol %) and thus comprised one data point in the training set (Figure 3A, Figures S79–81 and S91–S107, Tables S3 and S4). **iPrSi8** copolymerizes with DCPD to introduce cleavable bonds within the strands of the cross-linked pDCPD network without introducing any new cross-links, thereby lowering the cross-linking density of the material and the *T*_g_.^43^ By contrast, **DDMS** introduces new cleavable cross-links, leading to an overall increased cross-linking density that is expected to increase *T*_g_. Here, we sought to combine **iPrSi8** and **DDMS** to enable pDCPD deconstruction while also tuning the cross-link density. Figure 3B shows predicted *T*_g_ values from our fingerprint-based model ensemble as a function of the mol % of **DDMS** and **iPrSi**. As expected, increasing the mol % of **iPrSi8** lowers *T*_g_ (*x*-axis, blue), while increasing **DDMS** incorporation raises *T*_g_ (*y*-axis, red) relative to virgin pDCPD (lower left corner). To test the validity of these predictions, we synthesized pDCPD thermosets bearing 0–20 mol % **iPrSi8** and 10–20 mol % **DDMS** and estimated their *T*_g_ values as the peaks in the tan(δ) curves as measured by DMA. The predicted and experimental results were within the expected confidence interval as indicated by the standard deviation of the machine learning model, suggesting that the model captures key aspects of how the molecular features of **iPrSi8** and **DDMS** impact the *T*_g_ of these thermosets (Figure 3C). For example, in a sample comprising 10% **iPrSi8** and 20% **DDMS**, the measured *T*_g_ value was 152 ± 6 °C, which agrees well with the predicted *T*_g_ value of 158 ± 17 °C. Full chemical deconstructability of this sample was confirmed by exposure to tetrabutylammonium fluoride (TBAF) in tetrahydrofuran (THF) at 50 °C for 24 h. Thus, this predicted combination of **iPrSi8** and **DDMS** provides a pDCPD thermoset that is chemically deconstructable, with a *T*_g_ that is 13 °C greater than our previously reported deconstructable pDCPD based on **iPrSi8** alone and close to the value of virgin pDCPD (166 ± 5 °C).

### Screening, Identifying, And Experimentally Validating Predicted Properties of New BSE-Based Cleavable Comonomers

**Figure 4:**
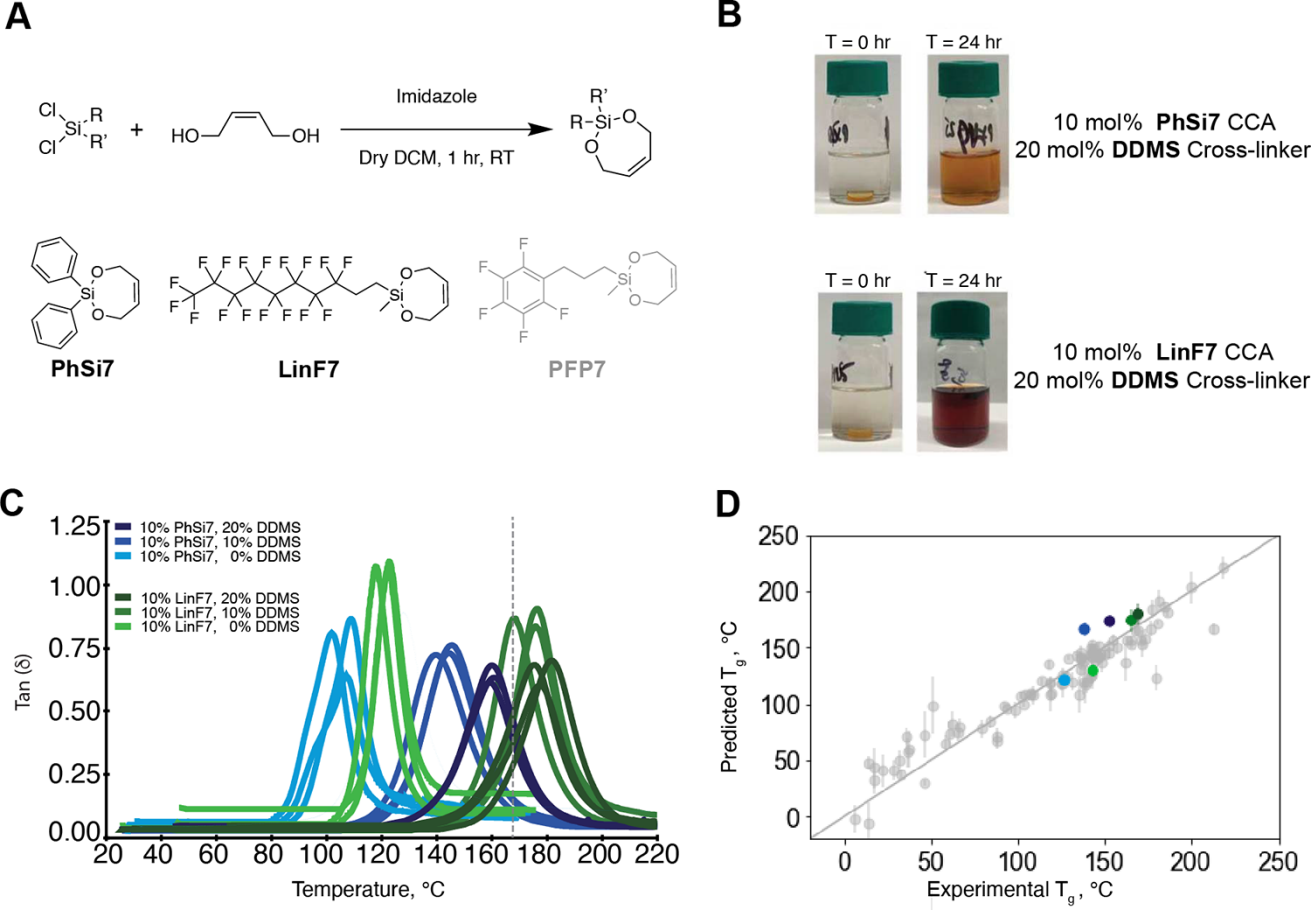
Model ensemble reliably predicts *T*_g_ values of deconstructable pDCPD thermosets with novel BSE-based cleavable comonomers and **DDMS**. (A) General scheme for BSE-based cleavable comonomer synthesis. Structures of comonomers synthesized, predicted, and experimentally verified by our model are shown in black (**PhSi7** and **LinF7**), and that of comonomer synthesized and predicted but not verified in bulk material due to lack of stability is shown in gray (**PFP7**). (B) Images of pDCPD samples with 10 mol % comonomer and 20 mol %. **DDMS** in 0.2 M TBAF in THF shows full dissolution. Samples shown contain **PhSi7** and **LinF7**, respectively. (C) Dynamic mechanical analysis (DMA) temperature sweeps of pDCPD thermoset samples doped with 10% comonomer, 10% comonomer, and 10% **DDMS** and those with 10% comonomer and 20% **DDMS** showing *T*_g_ from the peak of tan(δ). Dashed line shows *T*_g_ of virgin pDCPD. (D) Parity plot shows reasonable agreement of experimental and predicted *T*_g_ for the six selected compositions of **PhSi7**, **LinF7**, and **DDMS**. Vertical error bars note standard deviation of the *T*_g_ predicted using the different models in the ensemble, and horizontal error bars note the standard deviation across the experimental replicates. Color scheme follows the legend of panel C. The *y* = *x* line in the figure is meant to be used as the parity line to ascertain the difference between the predicted and experimental *T*_g_ values. If the dot is on the line, then the predicted *T*_g_ is same as the experimental *T*_g_; if it is off the line, then it is under/overpredicted.

Having shown that our model can predict the *T*_g_ values of pDCPD thermosets using various concentrations of **iPrSi8** mixed with **DDMS**, we sought to explore how the Si substituents in BSE-based cleavable comonomers could be leveraged to tune this property further. BSE comonomers such as **iPrSi8** can be readily prepared through substitution reactions of an appropriate diol (e.g., (*Z*)-pent-2-ene-1,5-diol or (*Z*)-but-2-ene-1,4-diol) and a dichlorosilane (SiCl_2_R_2_) (Figure 4A). As noted above, there are over 100 commercially available dichlorosilanes; synthesizing all possible BSE comonomers to study their effect on the *T*_g_ of pDCPD would be impractical. Thus, we used the same model described above to screen a library of BSE comonomer candidates that could, in principle, be synthesized from commercially available starting materials (Figure 4A). Our model suggested three BSEs, which were not in the training data set, with desirable predicted *T*_g_ values: the previously reported comonomer **PhSi7** and two novel BSE comonomers **LinF7** and **PFP7**, which were synthesized on the ∼2 g scale through cyclization of the corresponding dichlorosilane with (*Z*)-but-2-ene-1,4-diol in the presence of imidazole in dichloromethane (Figures S108–S114). ^1^H NMR, ^13^C NMR, and high-resolution mass spectrometry confirmed the structures of these comonomers. The thermal stabilities of pDCPD thermosets formed with 10% BSE comonomer loadings were assessed using thermogravimetric analysis (TGA) prior to thermomechanical characterization. Sufficient stability at the elevated temperatures required for DMA analysis was observed for materials containing **PhSi7** and **LinF7**, but not **PFP7** (Figure S127), and hence all subsequent experimental studies were carried out on samples containing **LinF7** and **PhSi7**. Deconstructable pDCPD samples (Figure 4B) were prepared by mixing 10 mol % of each BSE comonomer into neat DCPD, followed by addition of Grubbs second-generation catalyst at a final concentration of 2 mg/mL (∼3100:1 monomer to catalyst ratio) and curing in an oven at 120 °C for 30 min. Samples with 10 mol % of each comonomer along with 10 or 20 mol % **DDMS** cross-linker were also prepared for comparison (Tables S4 and S5). *T*_g_ values were determined experimentally for each sample through DMA analysis (Figure 4C, Figures S116–S136) for comparison to our model predictions.

The measured *T*_g_ value for each pDCPD sample containing 10 mol % **LinF7** was within the expected error as presented by the model’s standard deviation for the predicted *T*_g_ (predicted, 141 ± 19 °C; experimentally measured, 143 ± 1 °C). Agreement between the prediction and experiment was also observed for **LinF7** samples containing **DDMS**. For example, samples with 10 mol % **LinF7** and 10 mol % **DDMS** had a predicted *T*_g_ of 174 ± 10 °C and an experimental *T*_g_ within the predicted error at 166 ± 4 °C. This *T*_g_ value is ∼20 °C greater than that of **iPrSi8** samples loaded with similar concentrations of **DDMS**, showing that our method not only accurately predicts *T*_g_ values in these systems but also can be used to fine-tune and explore molecular subspaces to provide deconstructable thermosets with improved thermomechanical properties. Finally, we were interested in targeting deconstructable thermosets with predicted *T*_g_ values similar to that of virgin, nondeconstructable pDCPD. Our model predicted a *T*_g_ of 180 ± 9 °C for samples containing 10% **LinF7** and 20% **DDMS**. Experimental verification showed that these samples did match the *T*_g_ of virgin pDCPD (167 ± 5 °C), with *T*_g_ values of 170 ± 1 °C.

Predictions for samples containing **PhSi7** deviated more than justified by the model variance, suggesting a model bias. Nevertheless, the predictions remained within reasonable agreement with experiment, particularly compared to existing computational methods. For example, for thermosets containing 10 mol % **PhSi7**, the predicted *T*_g_ was 118 ± 6 °C, while the experimental value was 127 ± 2 °C. Additionally, for thermosets containing 10% **PhSi7** and 20% **DDMS**, the predicted *T*_g_ was 174 ± 6 °C, while the experimentally observed value was 153 ± 3 °C. (Figure 4D).

Finally, deconstruction of the samples containing the newly identified BSE-based cleavable comonomers into soluble fragments was confirmed upon treatment with TBAF for 24 h (Supporting Information Figures S137 and S138). For both materials, >97% of the mass of the puck fully dissolved to yield soluble fragments (Figure 4B). NMR spectroscopic analysis of the soluble fragments isolated from the deconstructed materials confirmed their composition (Figures S139–S142). We note that while **LinF7** was identified by the model to be a promising CCA based on *T*_g_, its use could result in the release of perfluoroalkylated substance (PFAS) waste. This fact highlights that our model is designed to predict a specific property (*T*_g_); the potential environmental ramifications of any predicted additives should also be considered in parallel with the property prediction before translation to real-world applications. With more data, models like ours could incorporate these considerations and others like cost in the future.

### Retrospective Prediction for Strand-Cleaving Cross-Linkers (SCCs)

**Figure 5:**
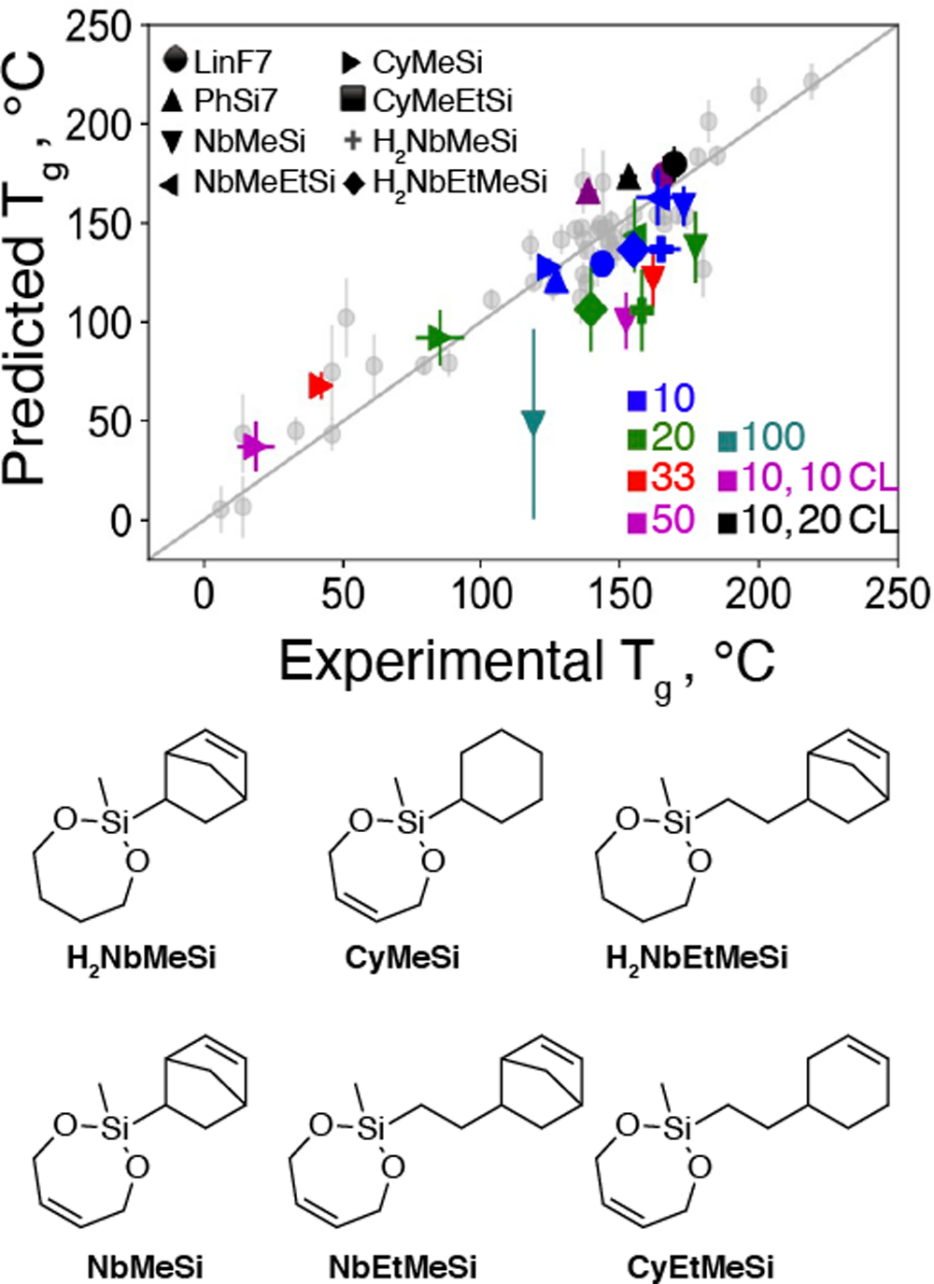
Parity plot of the retrospective analysis study shows reasonable agreement between experimental and predicted *T*_g_ values for reported comonomer/cross-linker combinations, including structures the model has not been trained on such as strand-cleaving cross-linkers (SCCs) (**NbMeSi** and **NbEtMeSi**), SCC control molecules (**H2NbMeSi**, **NbEtMeSiH2**, **CyEtMeSi**, and **CyMeSi**), and CCAs (**LinF7** and **PhSi7**) for comparison. A shape legend for the different molecules is noted in the top-left corner, and the concentrations are noted in the bottom-right corner. For a single number, the concentration is only for the comonomer, and for two numbers, separated by a comma, the first number is the comonomer concentration and the second preceded by CL (cross-linker) is the cross-linker concentration. Vertical error bars note the standard deviation of the *T*_g_ predicted using the different models in the ensemble, and horizontal error bars note the standard deviation across the experimental replicates. The values for the held-out data set are in gray. Structures from retrospective analysis shown below. The *y* = *x* line in the figure is meant to be used as the parity line to ascertain the difference between the predicted and experimental *T*_g_ values. If the dot is on the line, then the predicted *T*_g_ is same as the experimental *T*_g_; if it is off the line, then it is under/overpredicted.

In an effort to further assess our model’s performance, we conducted a retrospective analysis to predict the *T*_g_ values of a different class of previously reported cleavable additives for pDCPD that we refer to as “strand-cleaving crosslinkers” (SCCs, Figure 5), which were not in the training data.^46^ The model, despite having never seen any of these hybrid molecules, predicts *T*_g_ values comparable to the experimental values for all but one composition: a 100% v/v % **NbMeSi** thermoset that lacks DCPD. Given that this material is not a pDCPD thermoset, it is perhaps not surprising that our model inaccurately predicts its properties. Additionally, the prediction uncertainty for this sample, as quantified from the ensemble variance, was much higher than all other cases, demonstrating that the model exhibited a very low confidence in the predicted *T*_g_.

## Conclusions

Herein, we have demonstrated the use of data-driven strategies to streamline the discovery of BSE-based cleavable comonomers and cross-linkers to manufacture deconstructable pDCPD thermosets with targetable *T*_g_ values. Experimental validation showed good agreement with model predictions of *T*_g_ values for cleavable comonomer and cross-linker structures that were not in the model’s training set. Importantly, this process allows for the deconstruction of pDCPD variants with predictable, tailorable properties. Moreover, when combined with recycling and upcycling schemes that leverage the rich functionality of pDCPD deconstruction fragments,^41,80^ this approach could enhance the end-of-life options for pDCPD. Additionally, extension of these concepts to pDCPD composites may offer a powerful way to reuse the components of composites (e.g., fillers).^41,80^

This research also provides a framework to leverage closed-loop data-driven design and optimization in other areas where access to larger data sets may be limited, potentially allowing for the discovery of related deconstructable materials, including thermoplastics, other thermosets, and composites. Finally, extensions of this approach could enable predictions of additional thermomechanical properties for polymeric materials, such as elastic modulus, yield stress, and cross-link density, which could accelerate strategies for improving the functionality and sustainability of these materials.

## Supporting Information Available

Synthetic methods, characterization data, and machine learning model training and featurization data (PDF)
