# Supplementary material for: A Model Ensemble Approach Enables Data-Driven Property Prediction for Chemically Deconstructable Thermosets in the Low-Data Regime
Source: ACS Cent Sci. 2023 Sep 14;9(9):1810–9. doi: 10.1021/acscentsci.3c00502 (PMC10540282; doi:10.1021/acscentsci.3c00502)
Supplement: Supplementary file 1 — oc3c00502_si_001.pdf [file oc3c00502_si_001.pdf]

Supporting Information for:

A Model Ensemble Approach Enables Data-Driven Property Prediction for Chemically Deconstructable Thermosets in the Low Data Regime

Yasmeen S. AlFaraj,[†,1] Somesh Mohapatra,[†,2] Peyton Shieh,[1] Keith E. L. Husted,[1] Douglass G. Ivanoff,[3,4] Evan M. Lloyd,[4,5] Julian C. Cooper,[4,5] Yutong Dai,[1] Avni P. Singhal,[2] Jeffrey S. Moore,[3,4] Nancy R. Sottos,[3,4] Rafael Gomez-Bombarelli, [*,2] Jeremiah A. Johnson [*,1]

[1] Department of Chemistry, Massachusetts Institute of Technology, Cambridge, MA 02139, United States of America
[2] Department of Materials Science and Engineering, Massachusetts Institute of Technology, Cambridge, MA, 02139, United States of America
[3] Department of Materials Science and Engineering, University of Illinois at Urbana—Champaign, Urbana, Illinois 61801, United States of America
[4] The Beckman Institute for Advanced Science and Technology, University of Illinois at Urbana—Champaign, Urbana, Illinois 61801, United States of America
[5] Department of Chemistry, University of Illinois at Urbana—Champaign, Urbana, Illinois 61801, United States of America

[†] Indicates equal contribution.

*Authors to whom correspondence should be addressed; Email: rafagb@mit.edu, jaj2109@mit.edu

Table of contents

Materials and Methods……………………………………………………………………………S3

Small molecule synthesis……………………………………………………………………………S3

Cleavable comonomer additive (CCA) synthesis ……………………………………………………S3

Cleavable crosslinker (DDMS) synthesis……………………………………………………………S4

Comonomer doped pDCPD networks…………………………………………………………S5

Synthesis of comonomer doped pDCPD ……………………………………………………………S5

Dissolution of comonomer doped pDCPD …………………………………………………………S6

Determination of residual mass after degradation……………………………………………S6

Dynamic Mechanical Analysis ……………………………………………………………………S6

Sample preparation for dynamic mechanical analysis ……………………………………S6

Dynamic mechanical analysis ……………………………………………………………………S6

Machine Learning……………………………………………………………………………………S6

Featurization ………………………………………………………………………………………S6

Model Training ……………………………………………………………………………………S7

Feature Importance…………………………………………………………………………………S7

Figures ……………………………………………………………………………………………………S8

Training Data ………………………………………………………………………………………S8

Experimental Figures………………………………………………………………………………S98

Tables ……………………………………………………………………………………………………S149

References…………………………………………………………………………………………………S162

Materials and Experimental Methods

All reagents were purchased from commercial suppliers and were used without further purification unless otherwise noted. Grubbs 2nd Generation catalyst was purchased from Sigma-Aldrich, dissolved in dry dichloromethane, concentrated under vacuum, and finely powdered immediately before use. Using the catalyst directly resulted in poor dissolution upon addition of neat monomer.

^{1}H nuclear magnetic resonance (^{1}H NMR), ^{19}F nuclear magnetic resonance (^{19}F NMR) and ^{13}C nuclear magnetic resonance (^{13}C NMR) spectra were acquired at the MIT Department of Chemistry Instrumentation Facility on a Bruker AVANCE III DRX 400 or Neo 500. Chemical shifts are reported in ppm relative to signals from the NMR solvent: for $CDCl_3$, this corresponds to 7.26 for ^{1}H and 77.0 for ^{13}C spectra. High-resolution mass spectrometry (HRMS) measurements were obtained on a JEOL AccuTOF system at the MIT Department of Chemistry Instrumentation Facility. Specific materials characterization techniques are described in the relevant sections below.

Monomer Synthesis

Endo-dicyclopentadiene (Alfa) was distilled and immediately stored at 4 °C until ready to use. We observed that DCPD from other vendors resulted in variable results, including premature polymerization immediately upon monomer addition to catalyst. Cleavable comonomer synthesis followed general procedures for CCA synthesis from dichlorosilanes described in literature.[1]

Synthesis of PhSi7

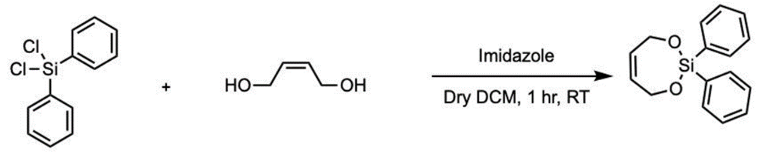

A 2 L 2-neck round bottom flask was dried and charged with a stir bar and 1 L of dry methylene chloride, cis-2-butene-1,4-diol (10 mmol, 0.82 mL), and imidazole (20 mmol, 1.4 g). 10 mmol of dichlorodiphenylsilane (2.07 mL) was dissolved in 50 mL of DCM, and added dropwise through an addition funnel at a rate of 2 drops/second. The reaction was stirred for an hour after addition. Precipitates were removed by filtration through a silica plug and concentrated to yield the analytically pure monomer as a clear oil (99% yield, 2.66 g).

^{1}H NMR (400 MHz, $CDCl_3$) δ 7.77 – 7.71 (m, 4H), 7.51 – 7.39 (m, 6H), 5.78 (d, J = 1.9 Hz, 2H), 4.59 (d, J = 1.7 Hz, 4H). ^{13}C NMR (101 MHz, $CDCl_3$) δ 134.79, 131.80, 130.61, 129.79, 127.92, 77.32, 77.00, 76.68, 62.12. HRMS (DART): Calculated for $C_{16}H_{17}O_2Si$ $[M+H]^+$: 269.0998, found 269.1003.

Synthesis of LinF7

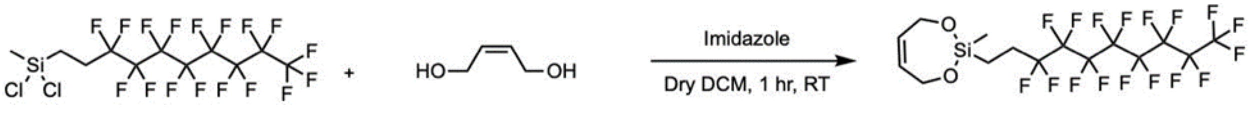

1 L of dry dichloromethane was added to an oven-dried 2 L 2-neck round bottom flask charged with a stir bar, cis-2-butene-1,4-diol (8 mmol, 0.656 mL) and imidazole (16 mmol, 1.12 g). 8 mmol of 1H,1H,2H,2H-perfluorodecyldichloromethylsilane (2.74 mL) was dissolved in 50 mL of DCM, and added dropwise through an addition funnel at a rate of 2 drops/second. The reaction was stirred for an hour following addition. Precipitates were removed by filtration through a silica plug and concentrated to yield the analytically pure monomer as a clear oil (45% yield, 2.08 g).

^{1}H NMR (400 MHz, $CDCl_3$) δ 5.72 (t, J = 1.8 Hz, 2H), 4.49 (d, J = 1.9 Hz, 4H), 2.27-2.09 (m, 2H), 0.98-0.90 (m, 2H), 0.25 (s, 3H). ^{13}C NMR (101 MHz, $CDCl_3$) δ 129.65, 61.98, 24.56, 3.43, -5.14. ^{19}F NMR (376 MHz, $CDCl_3$) δ -116.38, -121.73, -121.91, -122.70, -123.28, -126.08. HRMS (DART): Calculated for $C_{15}H_{14}F_{17}O_2Si$ $[M+H]^+$: 577.0492.

Synthesis of PFP7

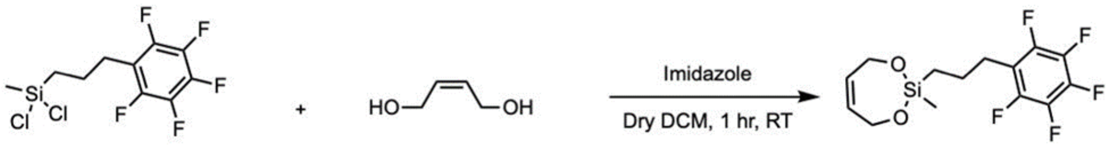

A 2 L 2-neck round bottom flask and stir bar were dried and charged with 1 L of dry methylene chloride, cis-2-butene-1,4-diol (10 mmol, 0.82 mL), and imidazole (20 mmol, 1.4 g). 10 mmol of pentafluorophenylpropylmethyldichlorosilane (2.3 mL) was dissolved in 50 mL of DCM, and added dropwise through an addition funnel at a rate of 2 drops/second. The reaction was stirred for an hour after addition was complete. Precipitates were removed by filtration through a silica plug, and concentrated to yield **PFP7** as a clear oil (68.8% yield, 2.33 g).

^{1}H NMR (400 MHz, $CDCl_3$) δ 5.70 (d, J = 2.0 Hz, 2H), 4.46 (s, 4H), 2.76 (t, J = 7.6 Hz, 2H), 1.79 – 1.59 (m, 2H), 0.80 – 0.71 (m, 2H), 0.20 (s, 3H). ^{13}C NMR (151 MHz, $CDCl_3$) δ 145.04, 139.45, 137.39, 130.66, 129.64, 77.21, 77.00, 76.79, 64.54, 58.52, 57.87, 25.44, 25.42, 25.34, 22.96, 22.83, 22.54, 16.98, 16.77, 16.75, 15.85, 15.37, 13.33, -1.84, -2.07.
^{19}F NMR (376 MHz, $CDCl_3$) δ -144.50, -158.02, -162.99. HRMS (DART): Calculated for $C_{14}H_{16}F_5O_2Si$ $[M+H]^+$: 339.0840, found 339.0874.

Synthesis of difunctional-DCPD-methyl-silyl-ether (DDMS)

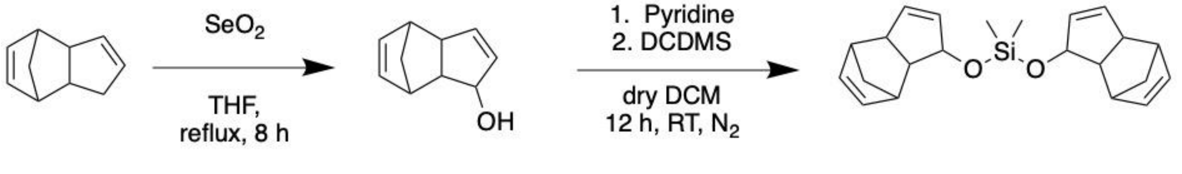

DCPD-OH was synthesized as follows via a literature procedure.[2] A 1000 mL three-neck round bottom flask was filled 500 mL of tetrahydrofuran, and DCPD (55.5g, 42 mmol) and selenium dioxide (26.7 g, 28.1 mmol) were added and allowed to stir at room temperature for 20 minutes. The reaction was allowed to reflux for 8 h. After cooling, the reaction mixture was filtered through

a celite pad, and the tetrahydrofuran was removed via reduced pressure. The reaction mixture was taken up in diethyl ether and washed twice with DI water. The organic phase was collected and dried over magnesium sulfate then filtered. The diethyl ether was removed via reduced pressure, and the red oil was purified via vacuum distillation (65-70 °C, 300 mTorr) to DCPD-OH, a clear oil that solidified into a white solid (34.0 g, 55%). ^{1}H and ^{13}C spectra agreed with values in the literature.[2]

Subsequently, a flame-dried 1000 mL three-neck round bottom flask was filled 500 mL of anhydrous DCM. Anhydrous pyridine (15.8 g, 200 mmol) and DCPD-OH (11.9 g, 80.0 mmol) were added to the flask. Dichlorodimethylsilane (DCDMS) (5.13 g, 39.7 mmol) was added dropwise to the flask over 15 minutes. The reaction was stirred for 12 h at room temperature under a nitrogen atmosphere. The mixture was washed with a saturated copper (II) sulfate aq. solution. The organic phase was collected and dried over sodium sulfate then filtered. The DCM was removed via reduced pressure, and the yellow liquid was purified via column chromatography in EtOAc /Hex (5:95) to yield CL1, a clear oil (10.7 g, 76%). ^{1}H and ^{13}C spectra and peak assignments in Section 6.5. ^{1}H and ^{13}C spectra in Section 6.5. ^{1}H NMR (500 MHz, $CDCl_3$) δ 5.98 (dd, *J* = 5.8, 3.0 Hz, 2H), 5.90 (dd, *J* = 5.9, 3.0 Hz, 2H), 5.79 (ddd, *J* = 5.0, 4.1, 2.2 Hz, 2H), 5.60 – 5.54 (m, 2H), 4.28 (t, *J* = 1.0 Hz, 2H), 3.53 – 3.35 (m, 2H), 3.04 (d, *J* = 3.0 Hz, 2H), 2.81 (d, *J* = 1.4 Hz, 2H), 2.64 (dddd, *J* = 7.9, 6.3, 4.3, 2.2 Hz, 2H), 1.62 – 1.57 (m, 2H), 1.48 – 1.40 (m, 2H), 0.26 – 0.06 (m, 6H). ^{13}C NMR (126 MHz, $CDCl_3$) δ 137.3, 135.8, 134.6, 132.5, 79.0, 55.0, 53.4, 51.5, 44.8, -1.56. HRMS-ESI (m/z): calculated for $C_{22}H_{28}O_2Si$ $[M+H]^+$, 353.1937, Found: 353.1926.

Synthesis of Comonomer Doped pDCPD

For mol% doped samples, addition was normalized against maintaining a 3100:1 monomer to Grubbs' 2nd generation catalyst (**G2**) initiator ratio. Keeping **G2** added at 2 mg (0.0024 mmol), a total of 7.44 mmol of monomer was added, with variations in accordance to the intended mol% of CCA, DCPD, or **DDMS**. For a 10 mol% CCA doped sample, 0.744 mmol of CCA was added by weight to a vial containing 2 mg of finely powdered **G2**. Subsequently, 6.696 mmol of DCPD was added (898 µL). For batch cured samples, the resulting mixture was added as 150 mg portions into glass vials, and heated at 120 °C for 30 minutes to cure. The vials were then broken to release the samples. For samples containing 10% comonomer and 20% **DDMS** crosslinker, 0.1488 mmol **DDMS** was added by weight (~ 52 mg) to a vial containing 2 mg **G2** prior to addition of CCA, and the remainder of the procedure were carried out as described above.

For a 10% v/v doped sample, DCPD (900 µL) was added to a comonomer (100 µL) then the mixture was added to a vial containing finely powdered Grubbs' 2nd generation catalyst (2 mg/mL). The resulting mixture was added as 150 mg portions into glass vials, and heated at 120 °C for 30 minutes to cure. The vials were then broken to release the samples.

For frontally polymerized samples indicated FROMP in the training set, the samples were prepared and initiated in accordance with the literature.[9] Training samples denoted "short oven cure" were prepared in accordance with the literature,[9] and batch cured for 30 minutes at 170 °C.

Dissolution of Comonomer Doped pDCPD

A sample was incubated with of 0.2 M TBAF in THF (5 mL) for 24-168 hours at 50 °C. The soluble fragments were carefully removed by pipette and the residual solids were resuspended in fresh THF. The fragments were redissolved in chloroform, concentrated, and characterized by NMR.

Determination of Residual Mass After Degradation

Samples of pDCPD with either 10 or 20 mol% comonomer were prepared, weighed, and added to pre-weighed 20 mL vials. Samples were then incubated in 0.2 M TBAF in THF (5 mL) for 48 hours. Residual solids were carefully isolated by removal of solution with a pipette. Samples were resuspsended in fresh THF (3 mL), which was immediately removed again to wash any deposited solids from the vials. Samples were then resuspended in THF (5 mL) and soaked for 2 days, after which THF was removed again and the vials were rinsed again. Vials were then dried under vacuum at 60 °C for 48 hours, then reweighed to determine the mass of remaining solids.

Sample Preparation for Dynamic Mechanical Analysis

Neat mixture of DCPD,comonomer, and crosslinker (DDMS) were added to a vial containing finely powdered Grubbs' 2nd generation catalyst (2 mg/mL) and vortexed until homogenous. The resulting mixture was added into a silicone mold with dimensions of c.a. 12 x 3 x 3 mm (approximately 300 µL per mold). The mold was prepared using MoldMax 50 (Reynolds Advanced Materials) poured over a dish containing 1 inch bars cut from a zinc-galvanized low-carbon steel bar (1/8" Thick, 1/8" Wide, 3 ft. long, McMaster-Carr). The samples were heated at 120 °C for 30 minutes, then taken out of the oven and cut out of the mold. The samples were further sanded before measurement by DMA to ensure suitable shape.

Dynamic Mechanical Analysis

Dynamic mechanical analysis was carried out in triplicate on a TA Instruments DMA Q800. Temperature sweeps were conducted from between -50 and 40 °C to 220 °C at a heating rate of 3 °C/min. A preload force of 0.01 N and 125.0% force tracking was applied during the measurements. Strain was applied at a frequency of 1 Hz. A TA instruments Liquid Nitrogen Purge Cooler (NPC) was used for samples which required low temperature measurements. Glass transition temperatures were extracted from the average global maxima in tan δ peaks across replicate runs. A minority of samples containing larger amounts of cleavable comonomer additives displayed some phase separation and exhibited multiple tan δ peaks. This can likely be explained by the breakdown of our statistical incorporation assumption for our CCAs, and instead, reactivity ratio considerations would be necessary for more accurate prediction of bulk material behaviour. For such outlier samples, in consistency with literature cited, the glass transition temperature was still extracted from the average global maxima, and the authors recognize the limitations this may incur for future investigation for samples including large quantities of CCAs with the current model at hand.

Machine Learning

Featurization

The chemical structures for degradable and non-degradable co-monomers and crosslinkers were represented as extended connectivity fingerprints (ECFPs)[3] and physicochemical descriptors, for

respective model ensembles. ECFPs with radius 3 and 128 bits were generated using RDKit.[4] 29 physicochemical descriptors, same as used in benchmarking in Mohapatra et al. Nat Mach Intel 2020,[5] were calculated using RDKit. The mol% for the comonomers and crosslinkers, and monomer to catalyst ratio were represented as numerical values, and Grubb's catalyst generation was noted as one-hot encoding. For each composition, all features were stacked together into a vector.

Model training

For both fingerprints and descriptors, 15 models with 10 train-test splits, a total of 150 models, were trained to predict Tg. Out of these models, top 5 model architectures and their splits in ascending order of root mean squared error on the test dataset, a total of 50 models, were chosen for virtual screening. Hypeparameters for each model were optimized using scikit-optimize. The choice of model architectures and hyperparamters are same as reported for benchmarking in Schissel, et al. biorXiv 2020.[6]

Feature importance

Feature importance was calculated by averaging absolute value of coefficients and normalizing by dividing the maximum value for linear models, and using the feature importance scores for non-linear models, wherever applicable. Further for both fingerprint and descriptor-based models, feature importance scores were averaged across all models.

Figures

Training Data

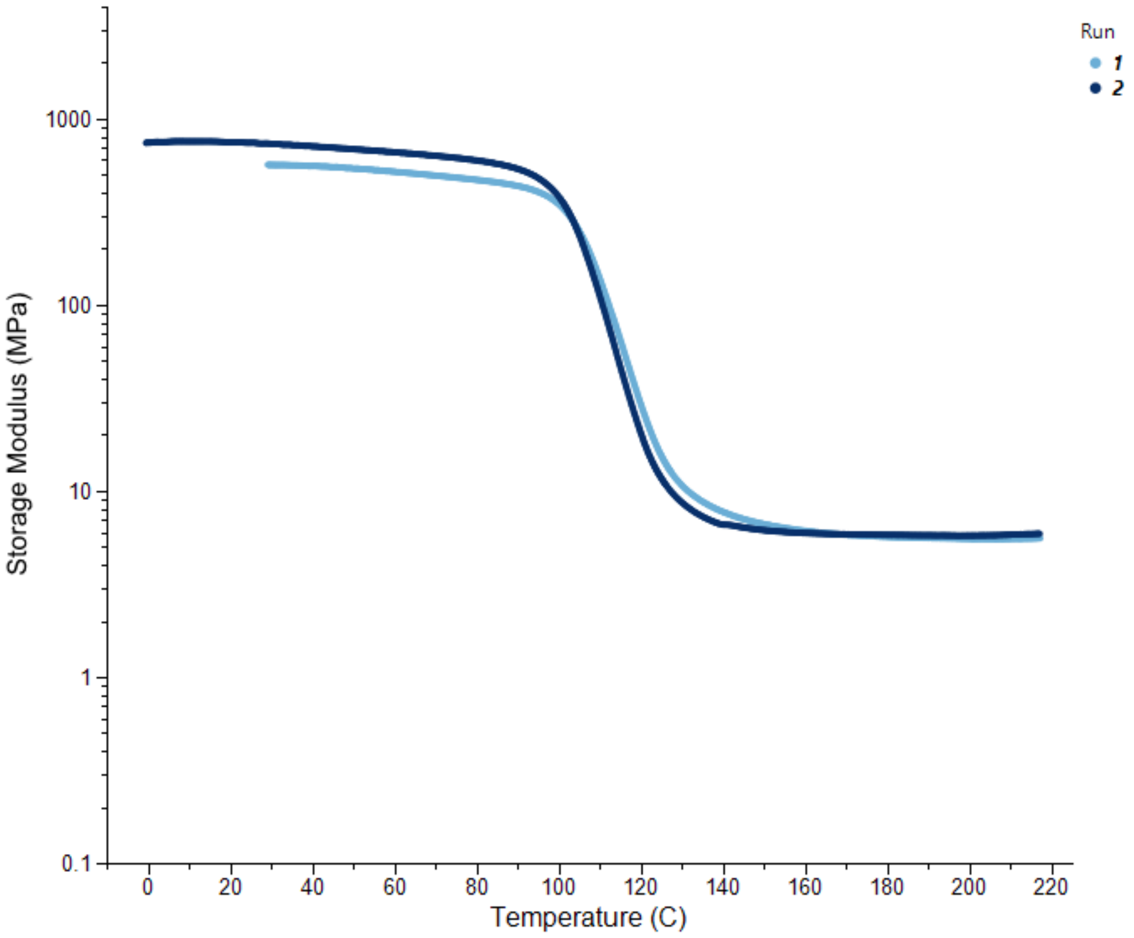

Figure S1. Storage Modulus Traces of pDCPD doped with 10% v/v **EtSi8.**[1]

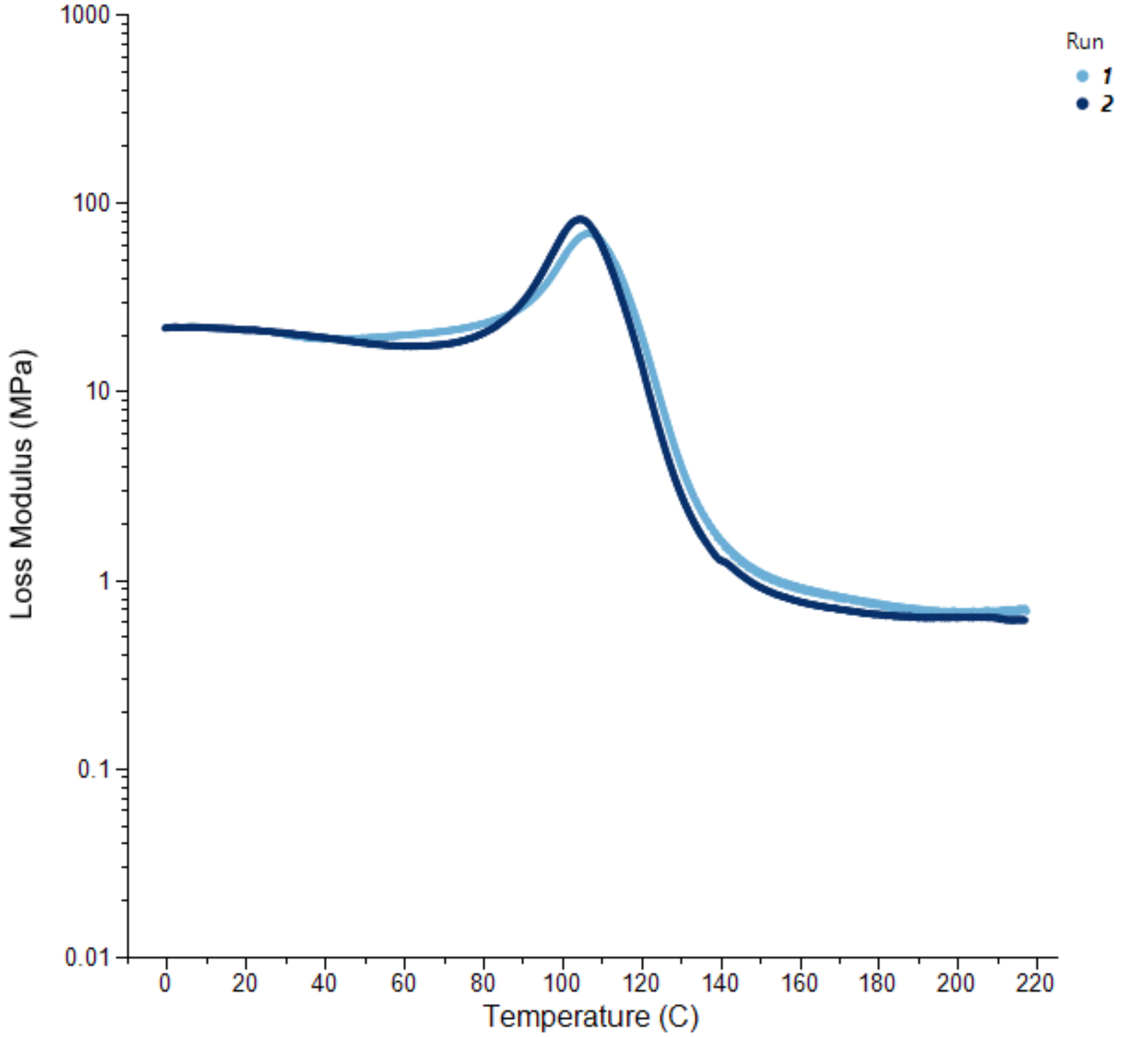

Figure S2. Loss modulus traces of pDCPD doped with 10% v/v **EtSi8.**

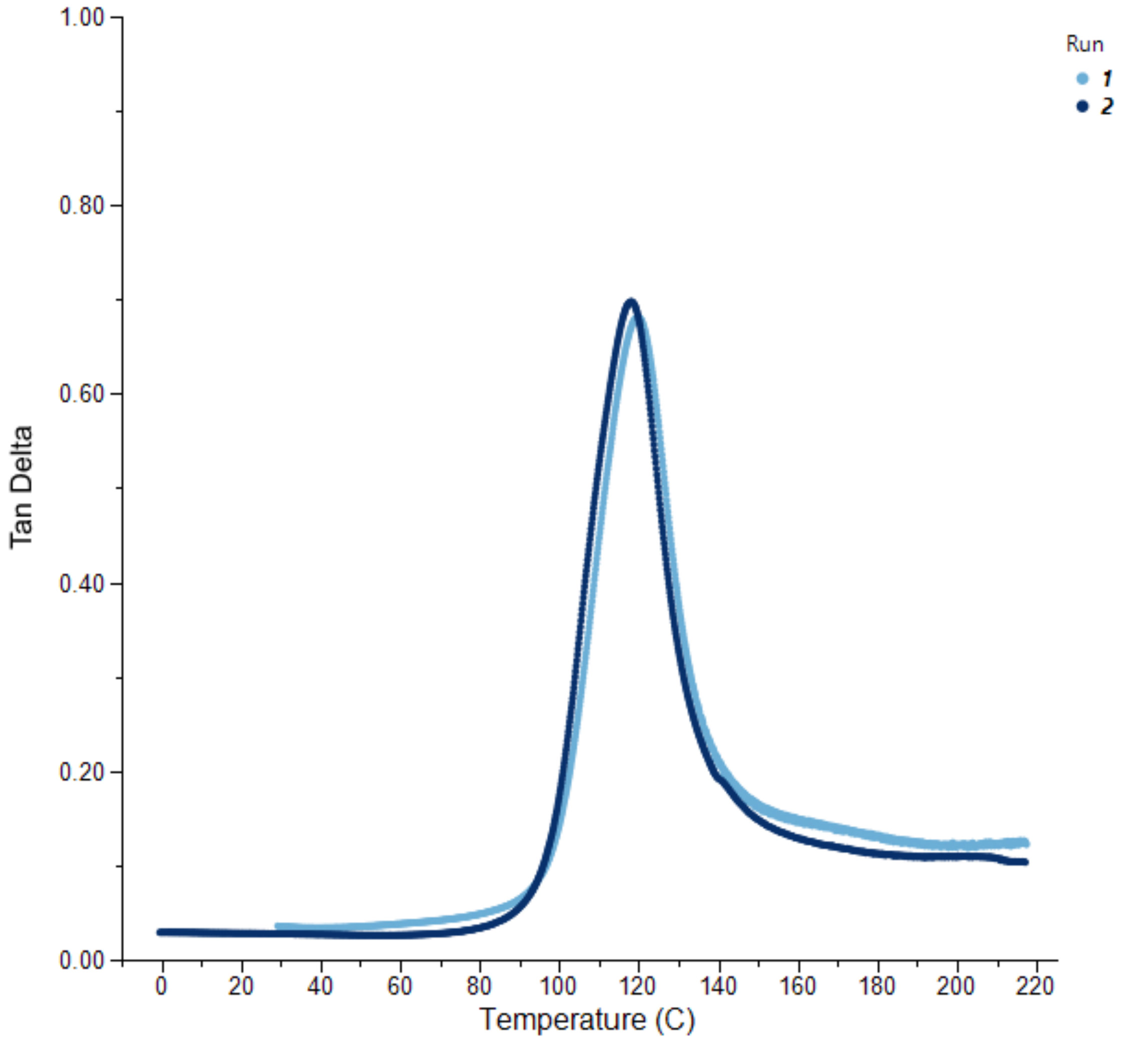

Figure S3. Tan delta traces of pDCPD doped with 10% v/v **EtSi8.**

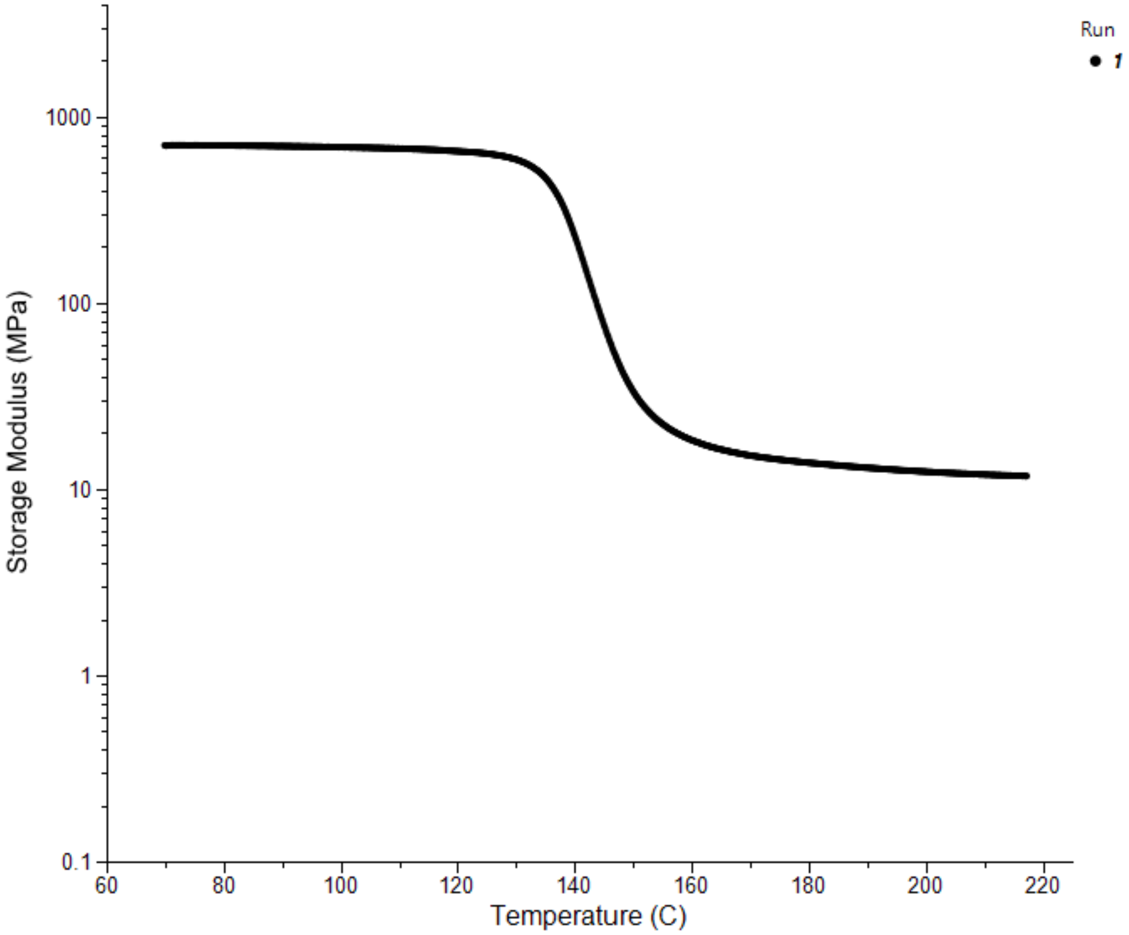

Figure S4. Storage modulus trace of pDCPD doped with 5% v/v **EtSi8.**

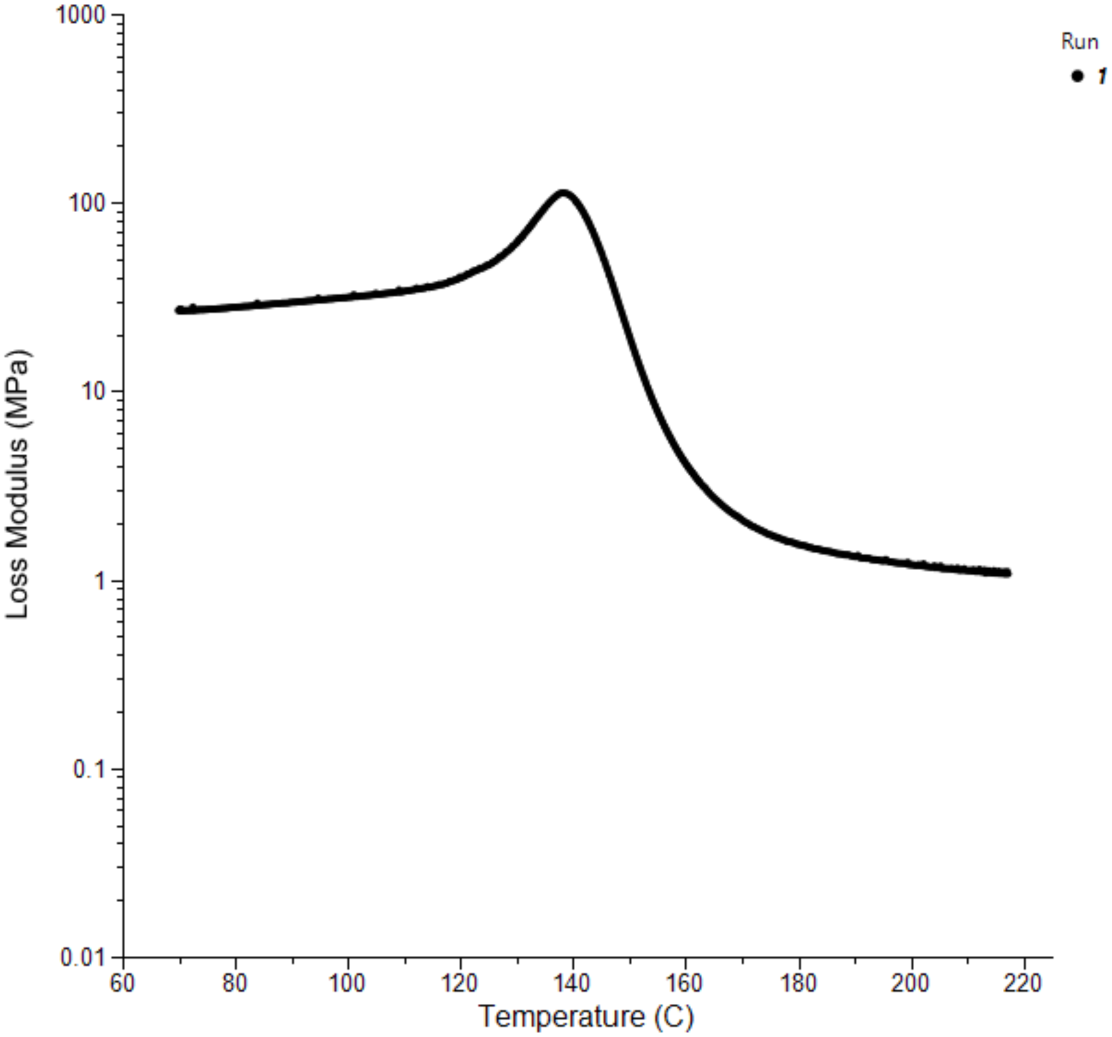

Figure S5. Loss modulus trace of pDCPD doped with 5% v/v **EtSi8**.

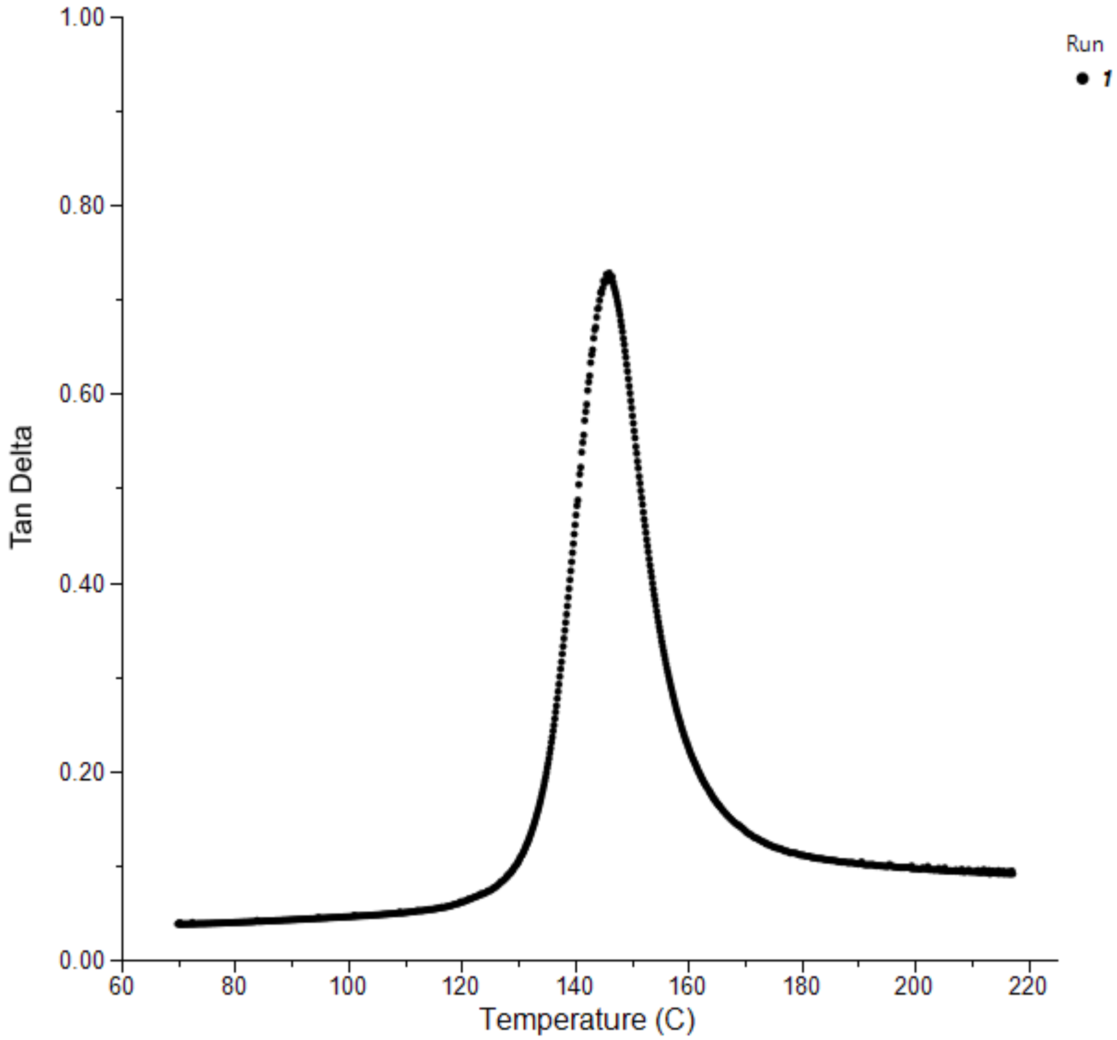

Figure S6. Tan delta trace of pDCPD doped with 5% v/v **EtSi8**.

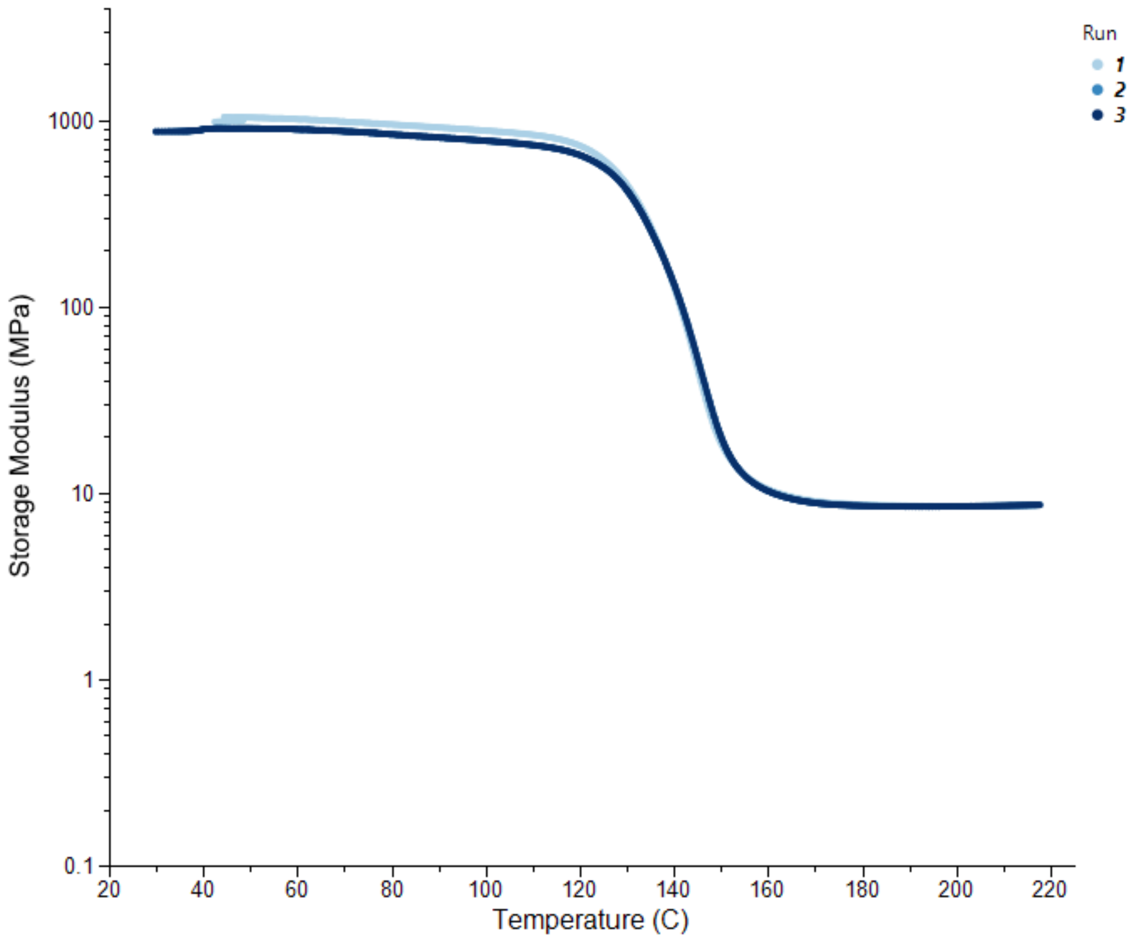

Figure S7. Storage modulus traces of pDCPD doped with 5% v/v **iPrSi7.**

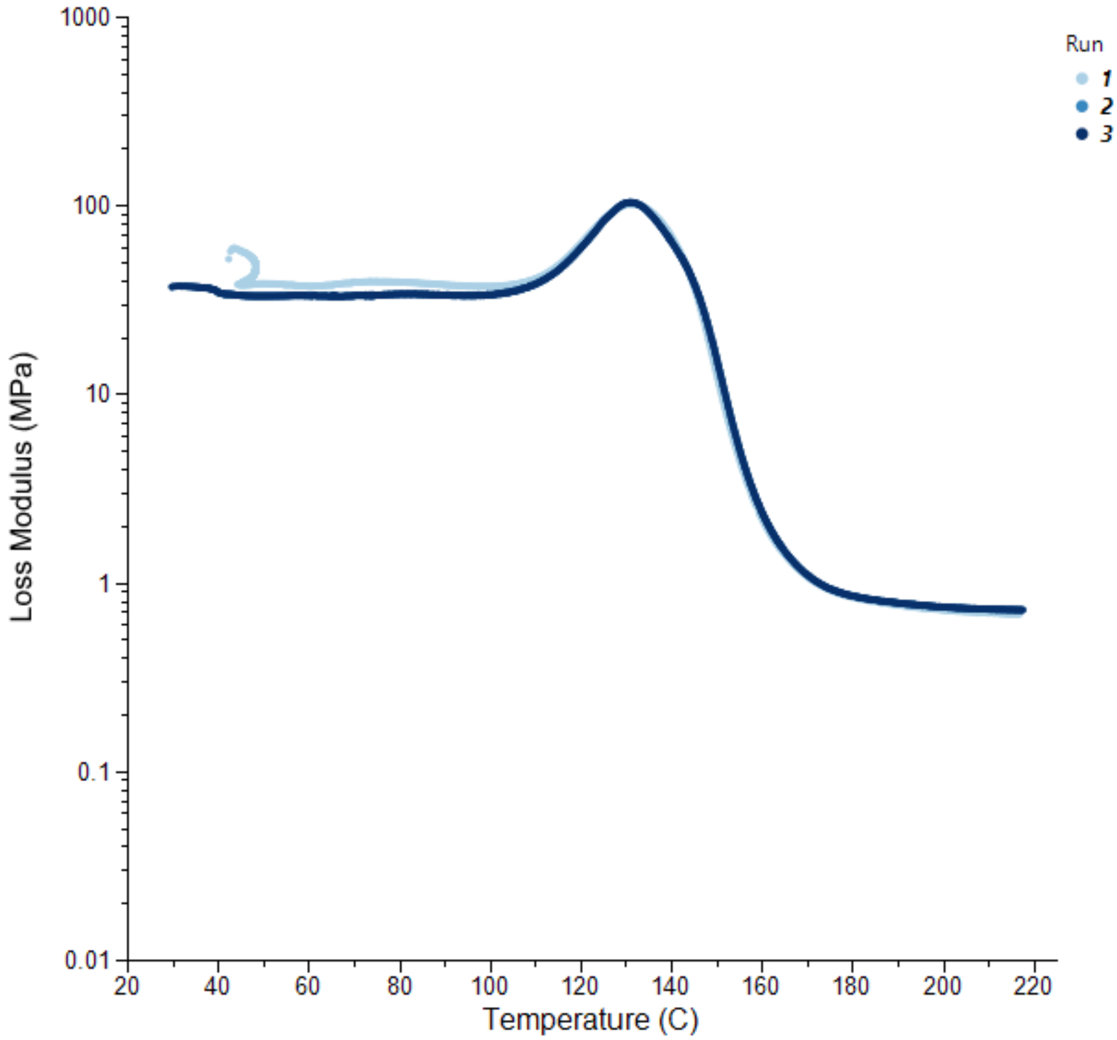

Figure S8. Loss modulus traces of pDCPD doped with 5% v/v **iPrSi7.**

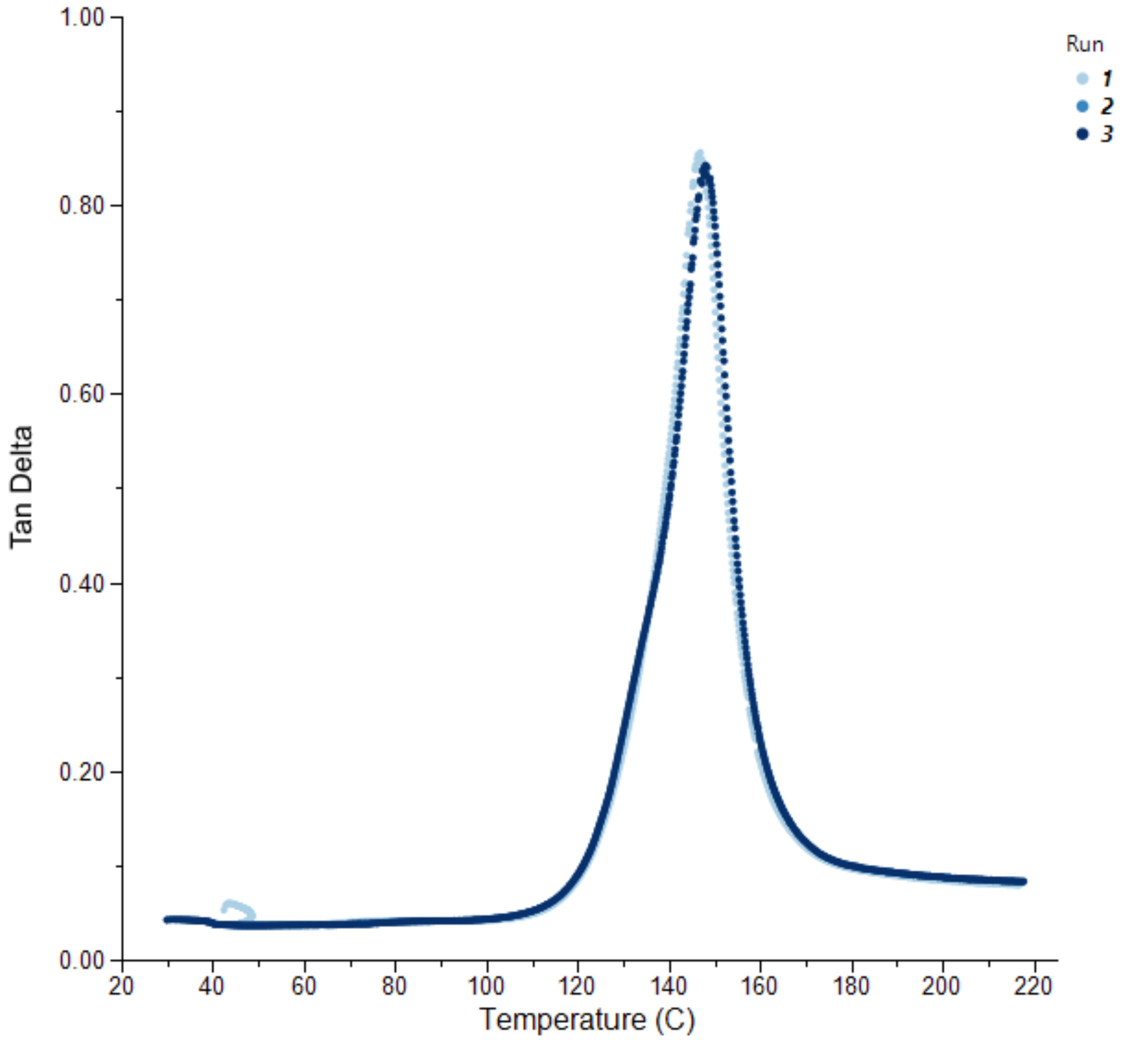

Figure S9. Tan delta traces of pDCPD doped with 5% v/v **iPrSi7.**

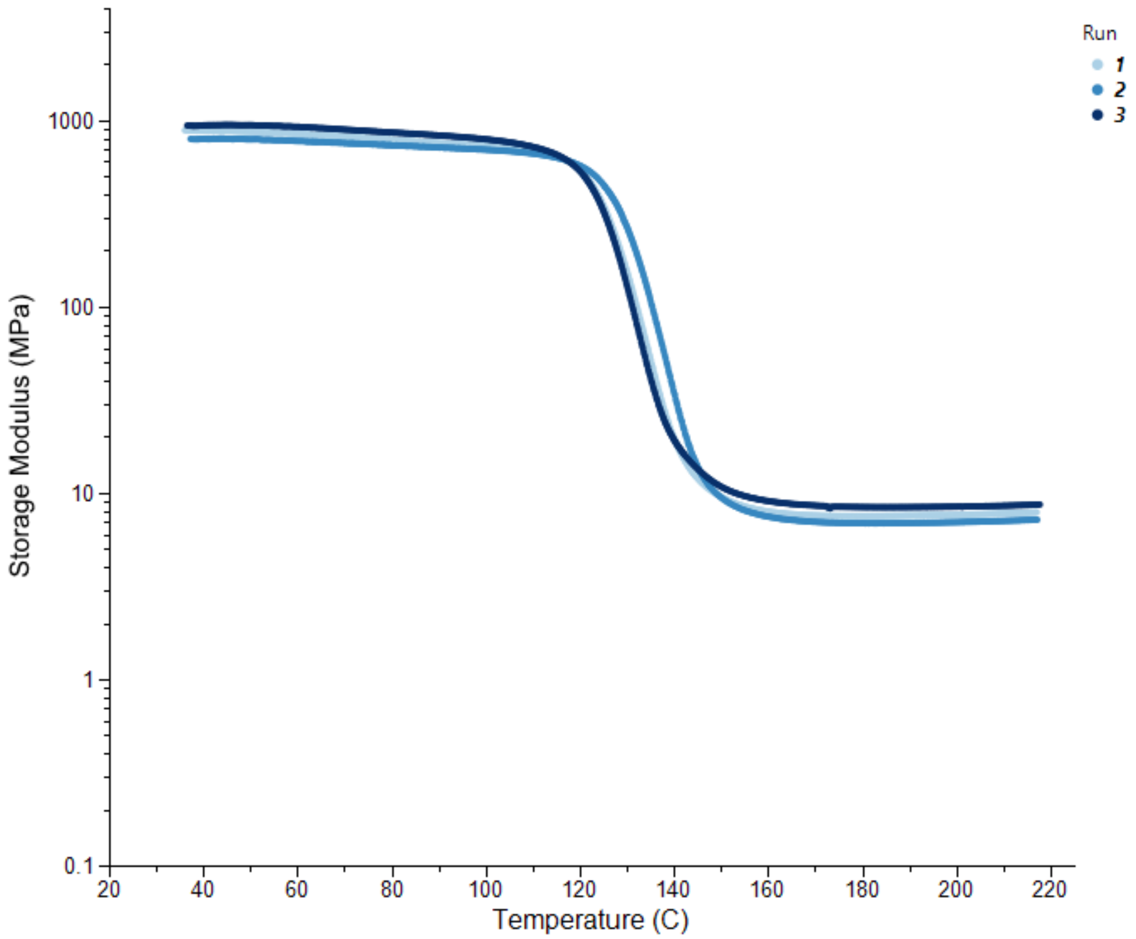

Figure S10. Storage modulus traces of pDCPD doped with 7.5% v/v **iPrSi7.**

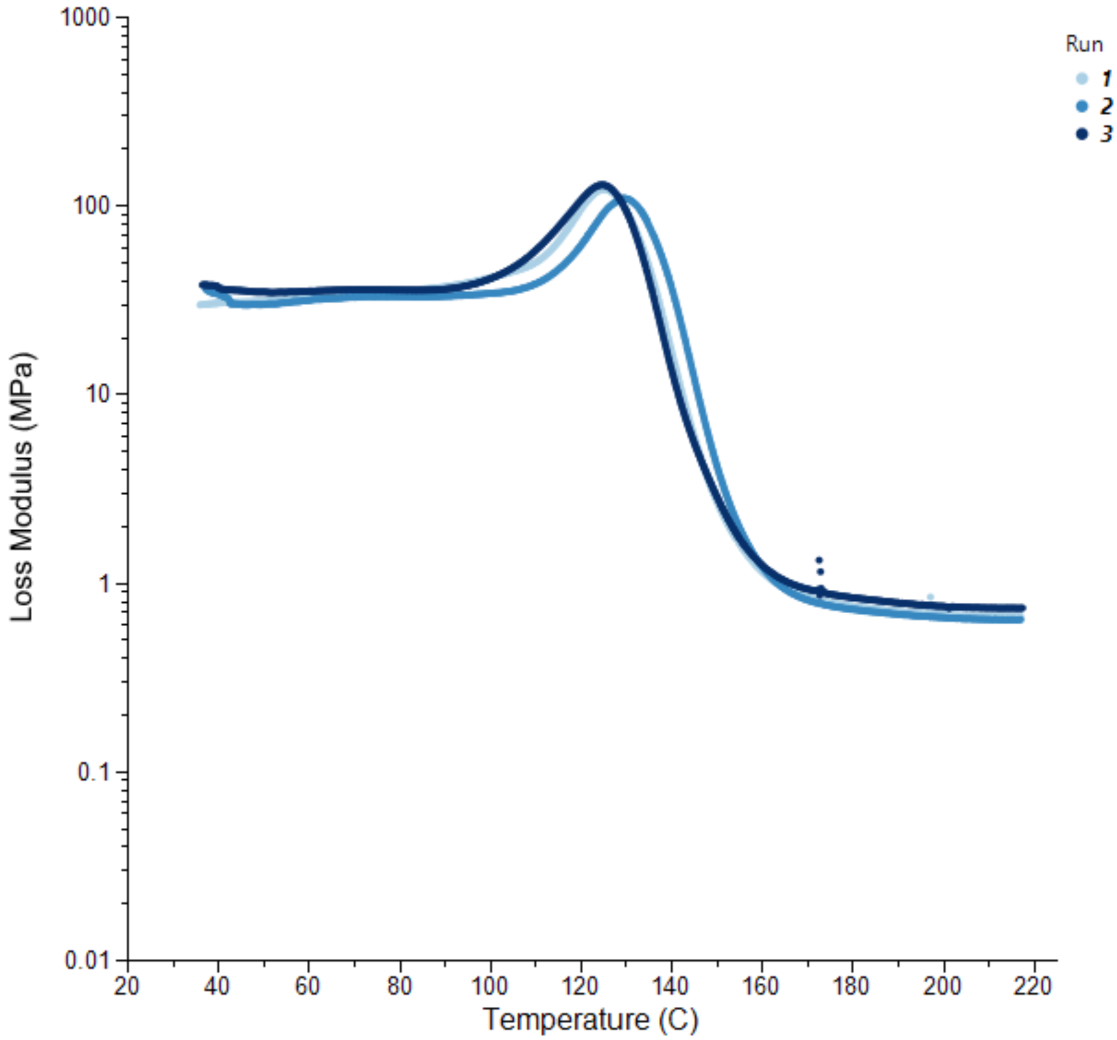

Figure S11. Loss modulus traces of pDCPD doped with 7.5% v/v **iPrSi7.**

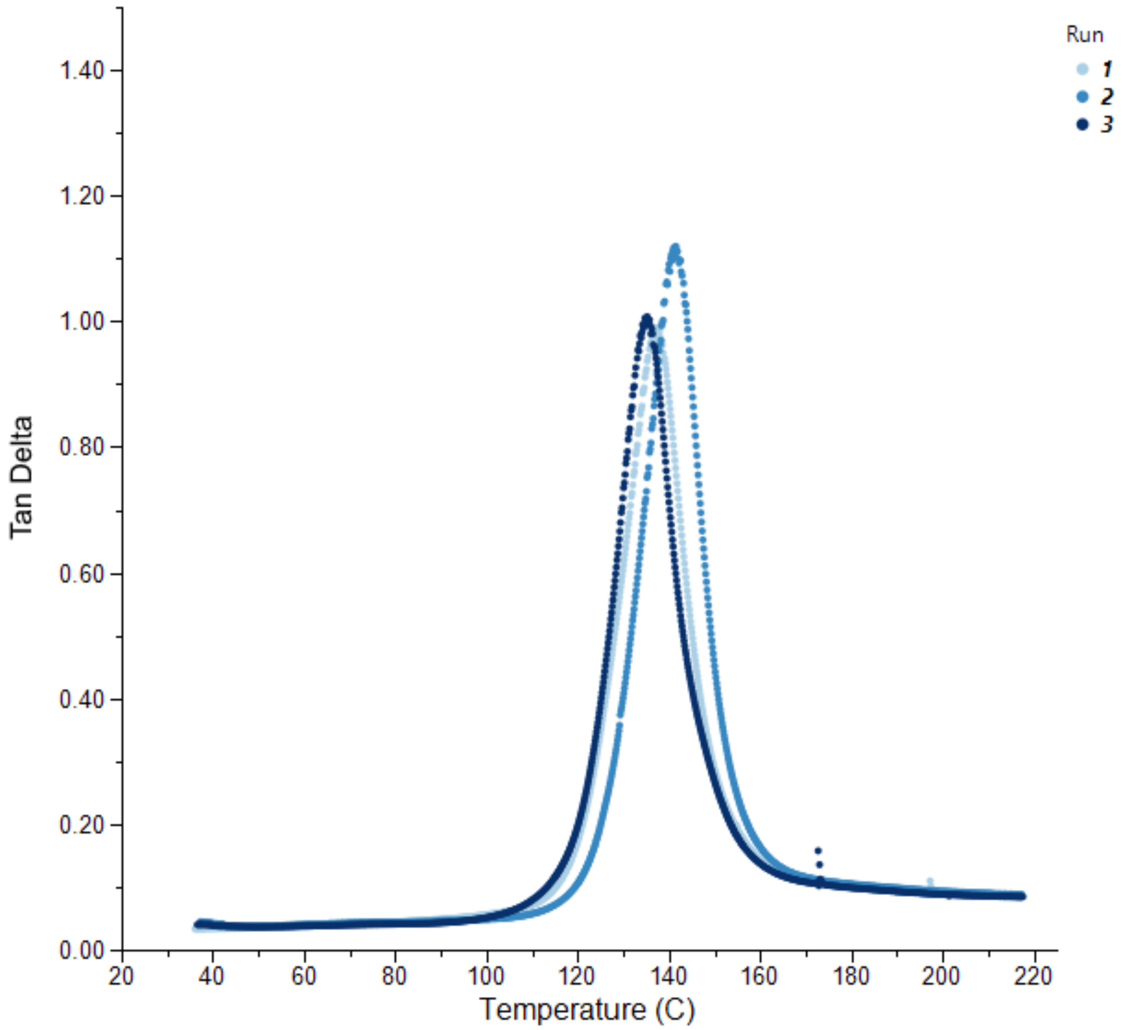

Figure S12. Tan delta traces of pDCPD doped with 7.5% v/v **iPrSi7.**

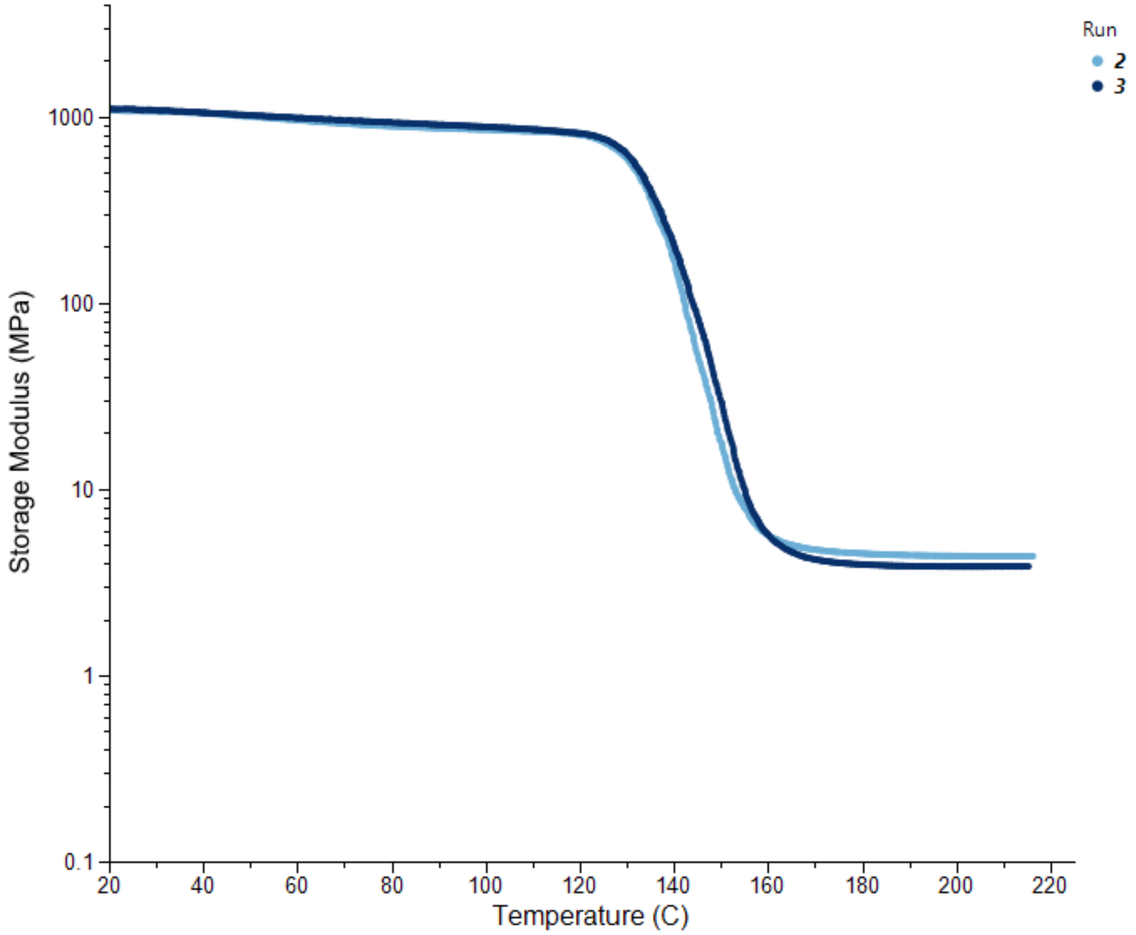

Figure S13. Storage modulus traces of pDCPD doped with 5% v/v **iPrSi8.**[1]

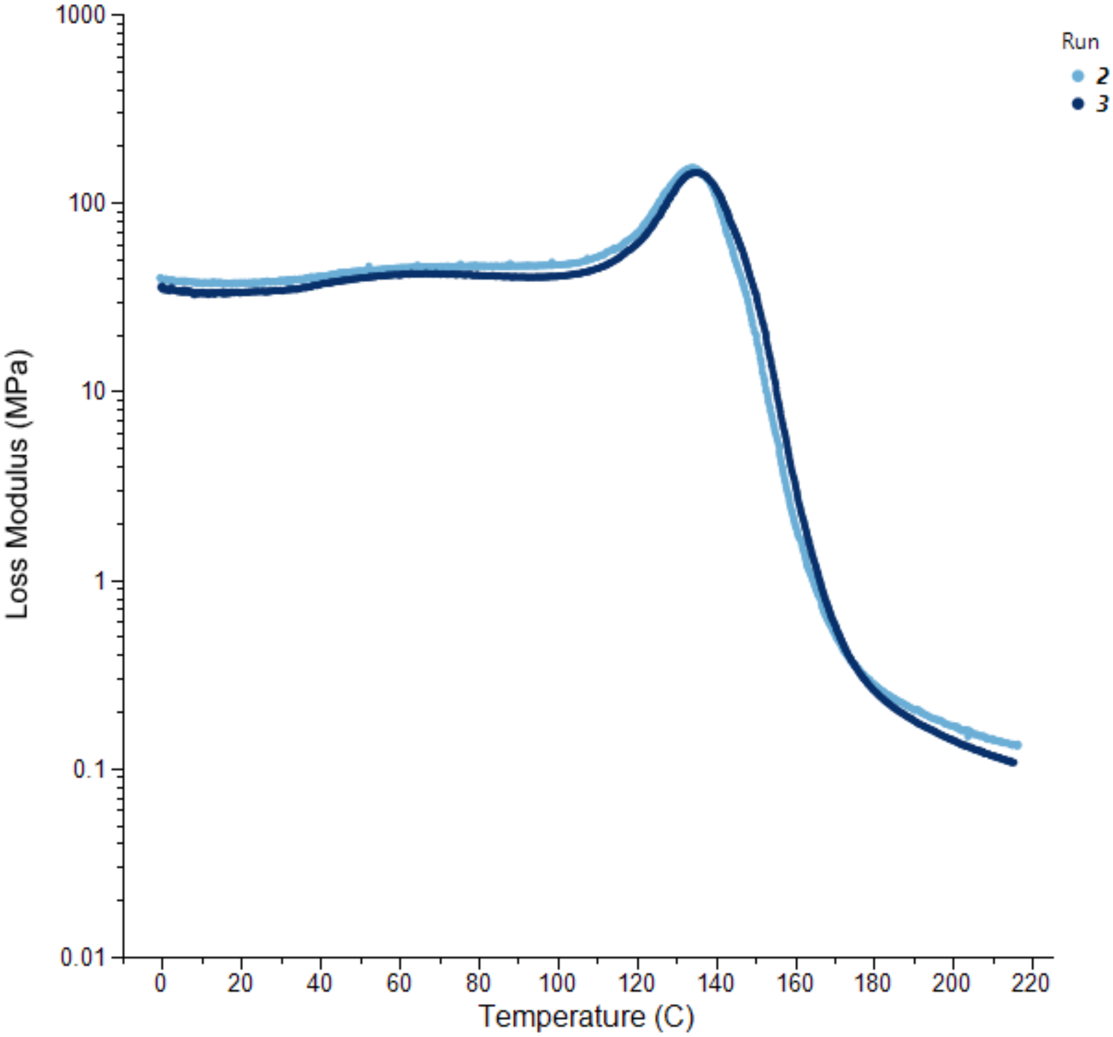

Figure S14. Loss modulus traces of pDCPD doped with 5% v/v **iPrSi8.**

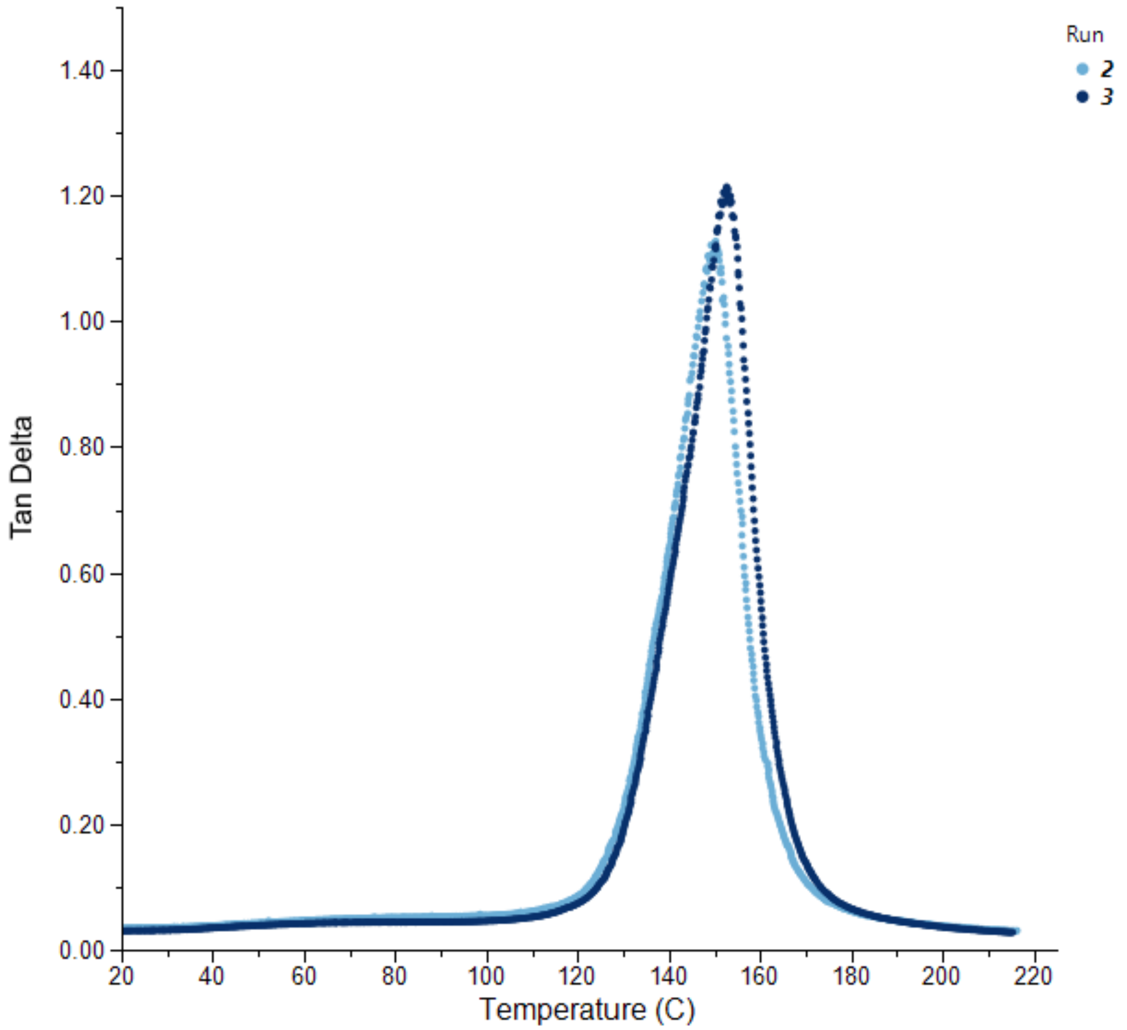

Figure S15. Tan delta traces of pDCPD doped with 5% v/v **iPrSi8.**

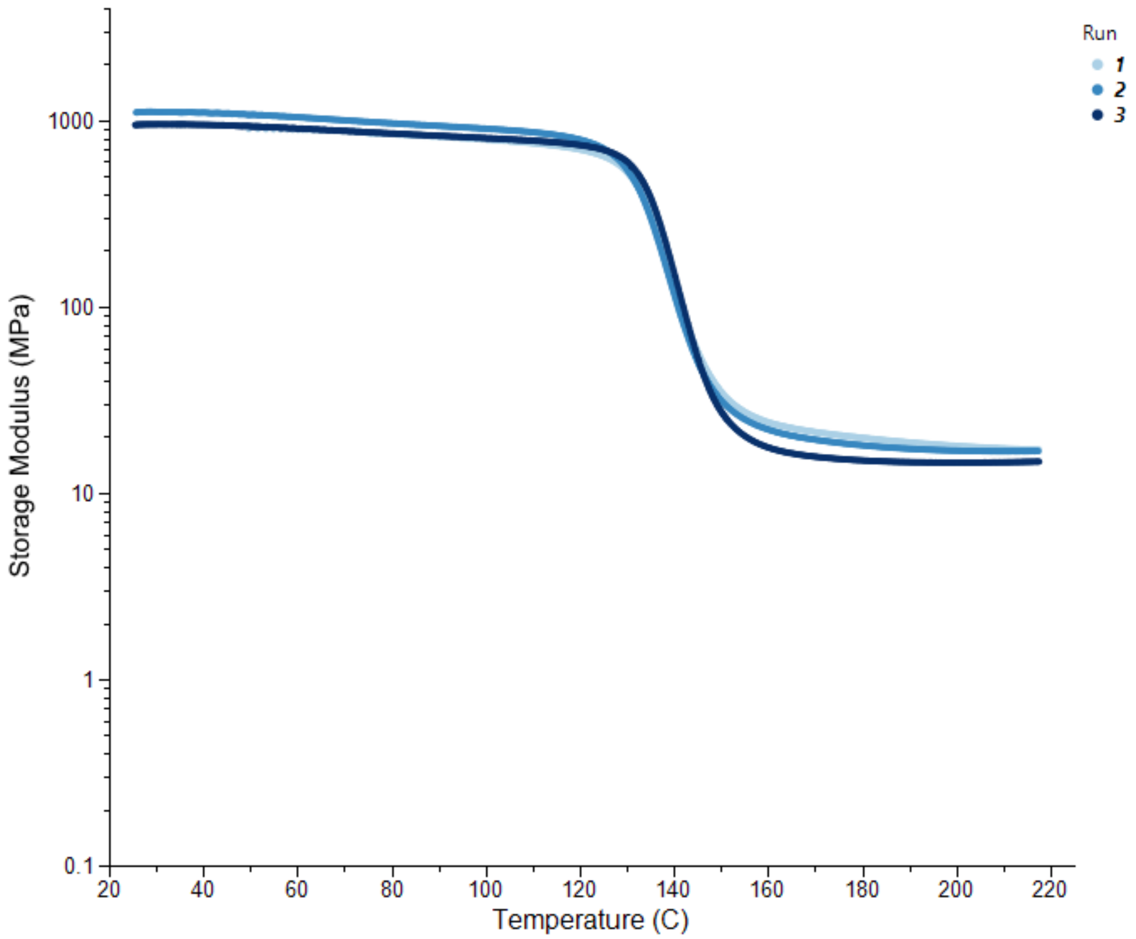

Figure S16. Storage modulus traces of pDCPD doped with 7.5% v/v **iPrSi8** and 10% v/v **SiXL.**[8]

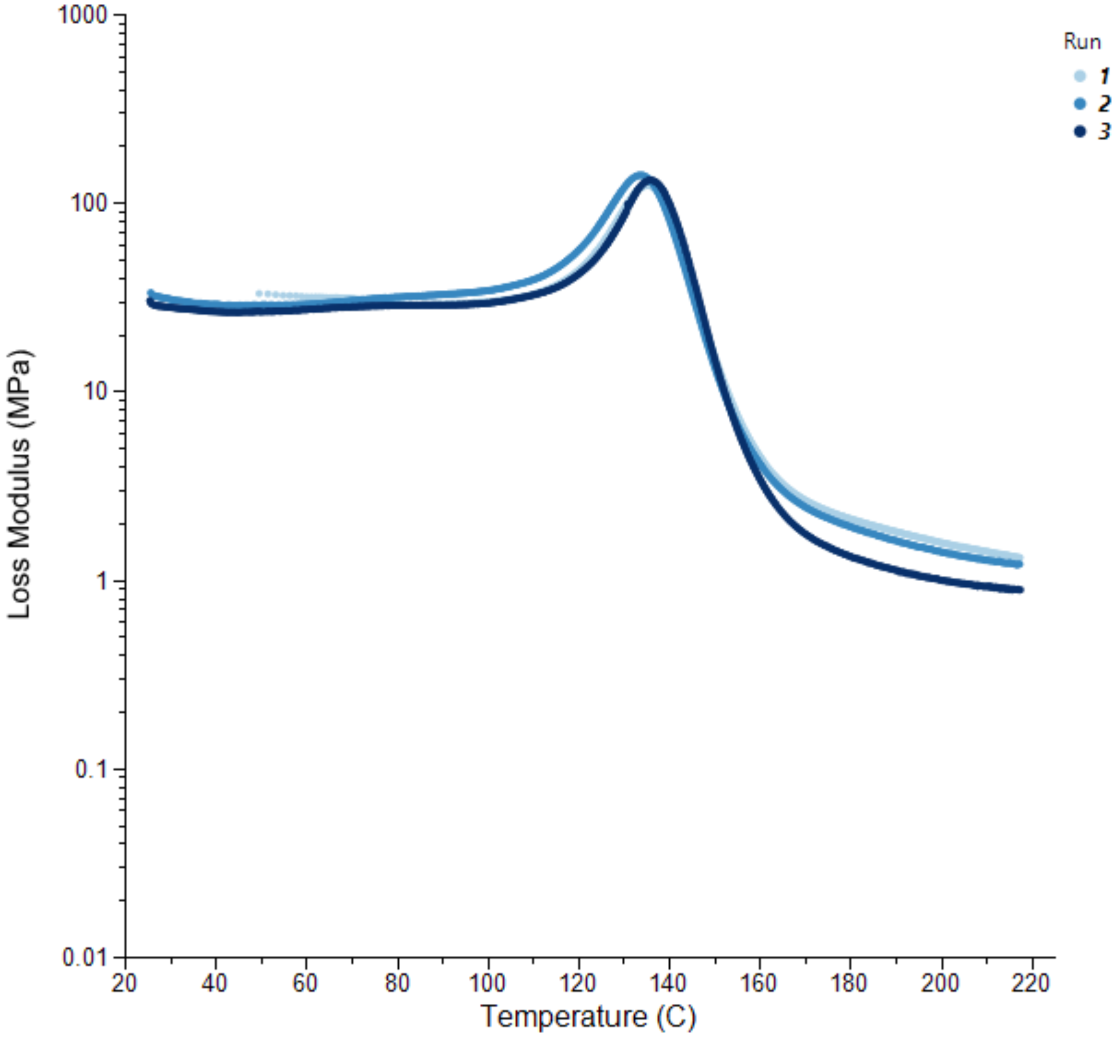

Figure S17. Loss modulus traces of pDCPD doped with 7.5% v/v **iPrSi8** and 10% v/v **SiXL.**

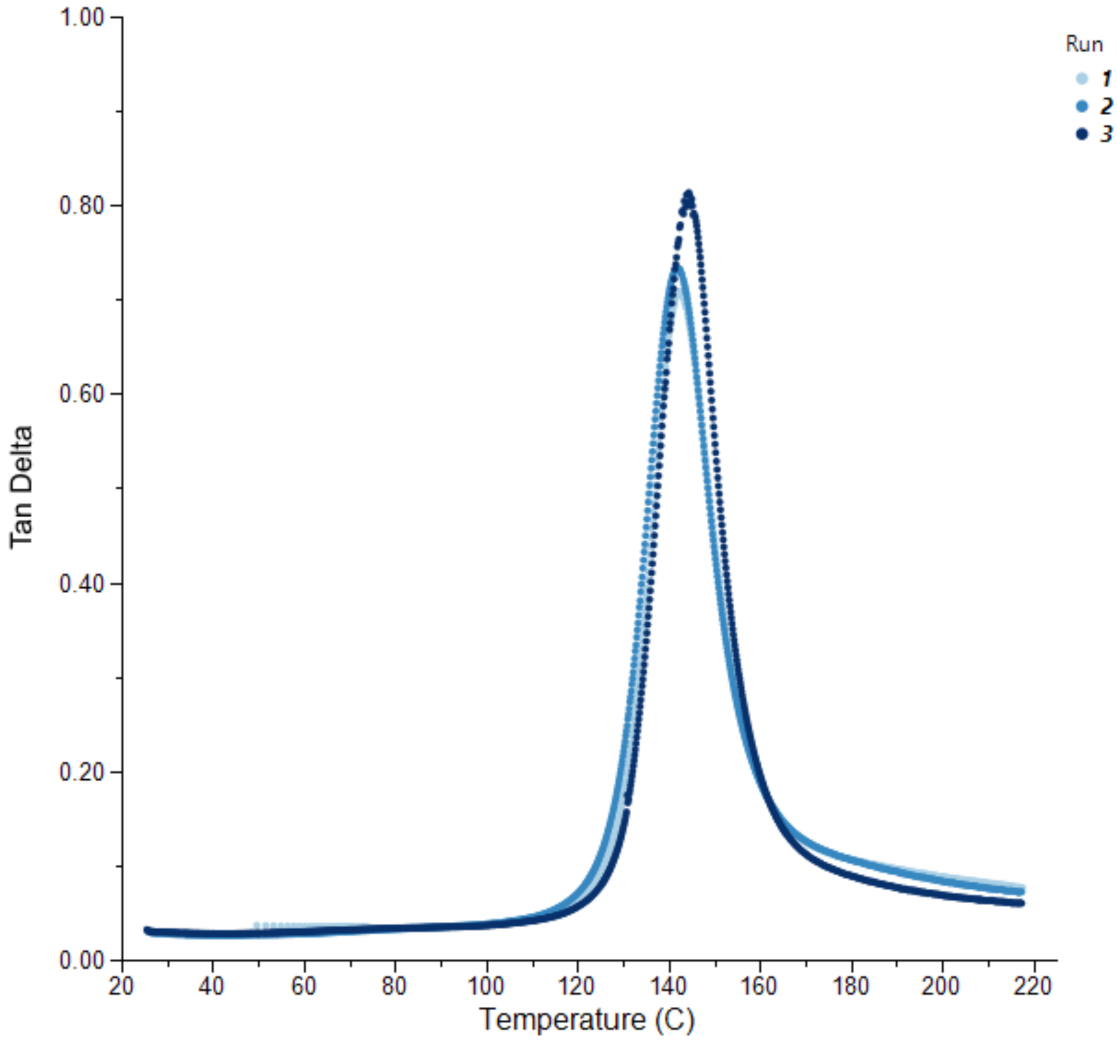

Figure S18. Tan delta traces of pDCPD doped with 7.5% v/v **iPrSi8** and 10% v/v **SiXL**.

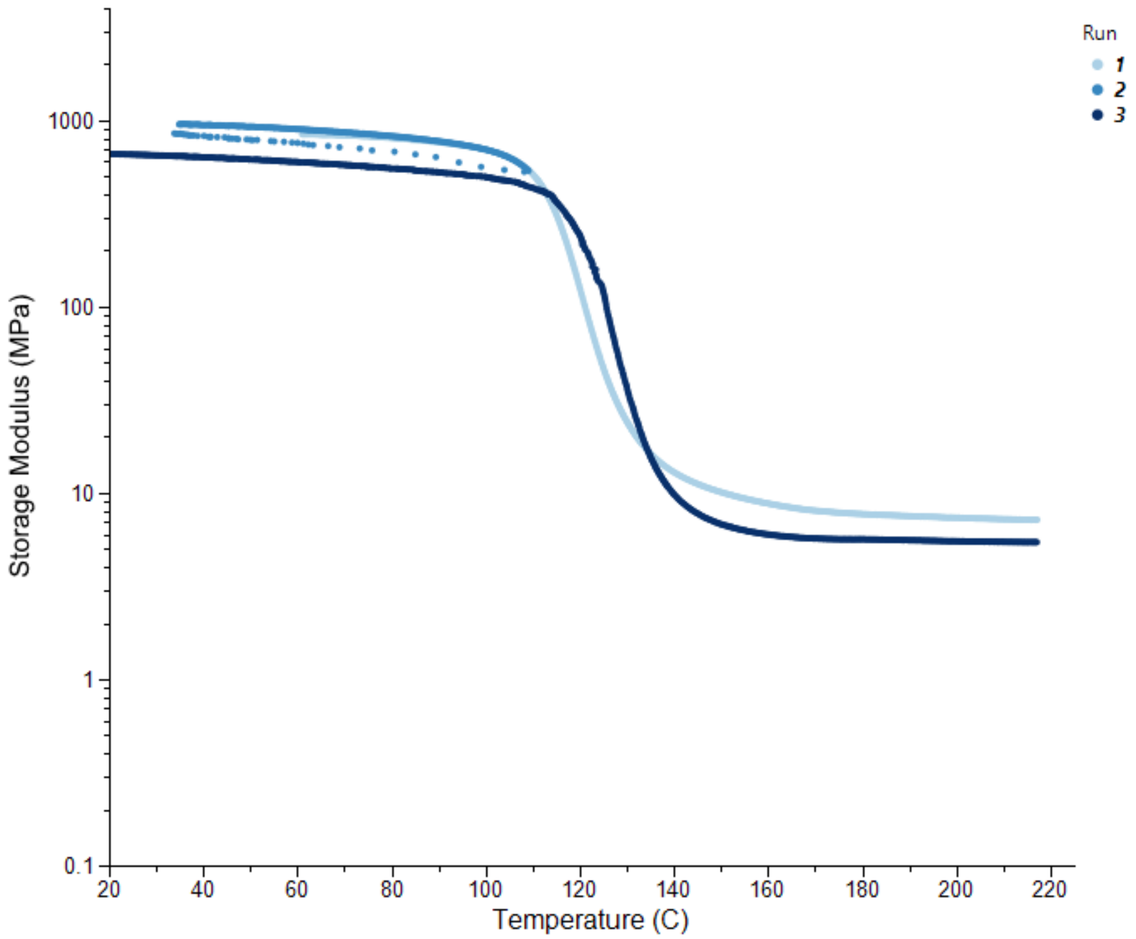

Figure S19. Storage modulus traces of pDCPD doped with 10% v/v **MeSi8**.[1]

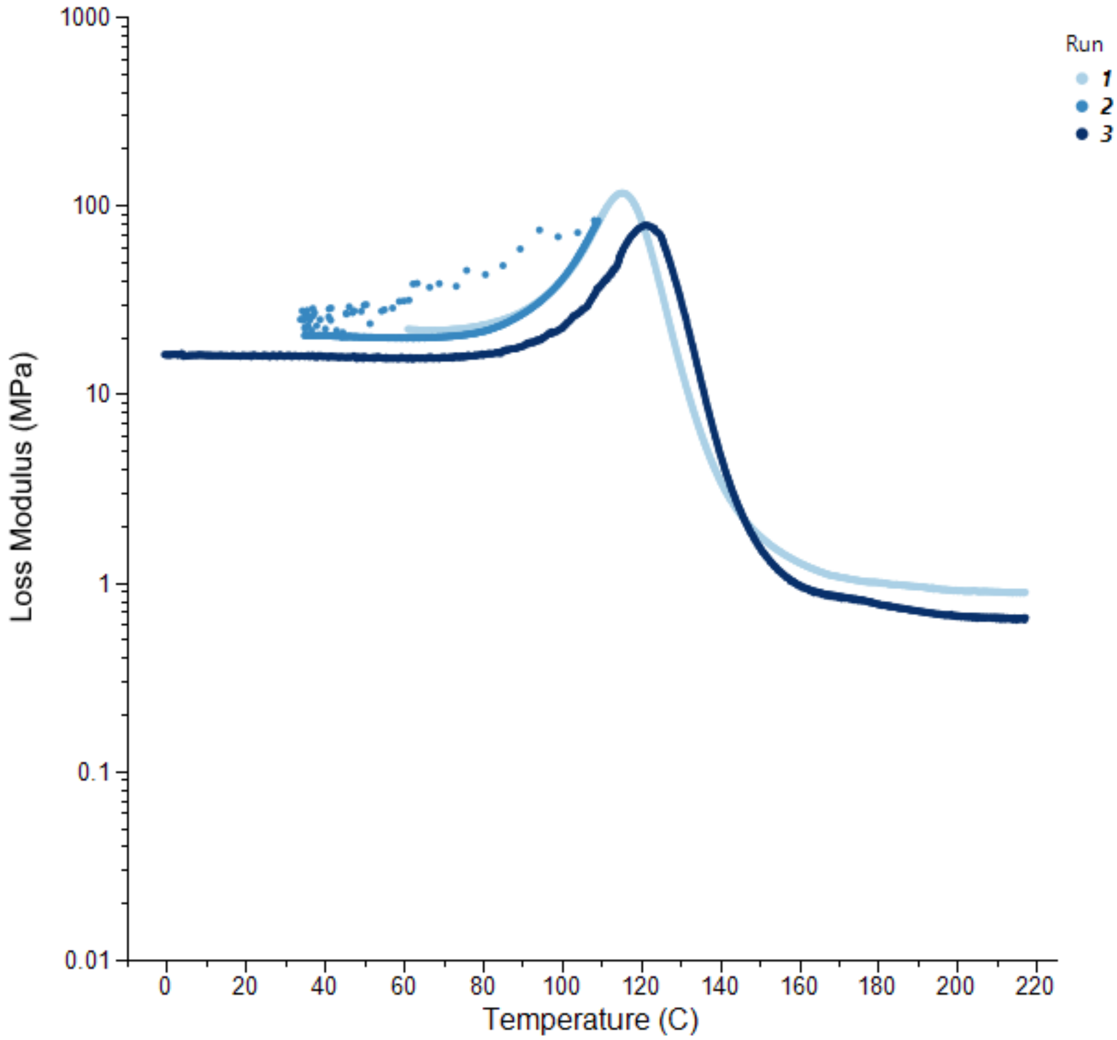

Figure S20. Loss modulus traces of pDCPD doped with 10% v/v **MeSi8.**

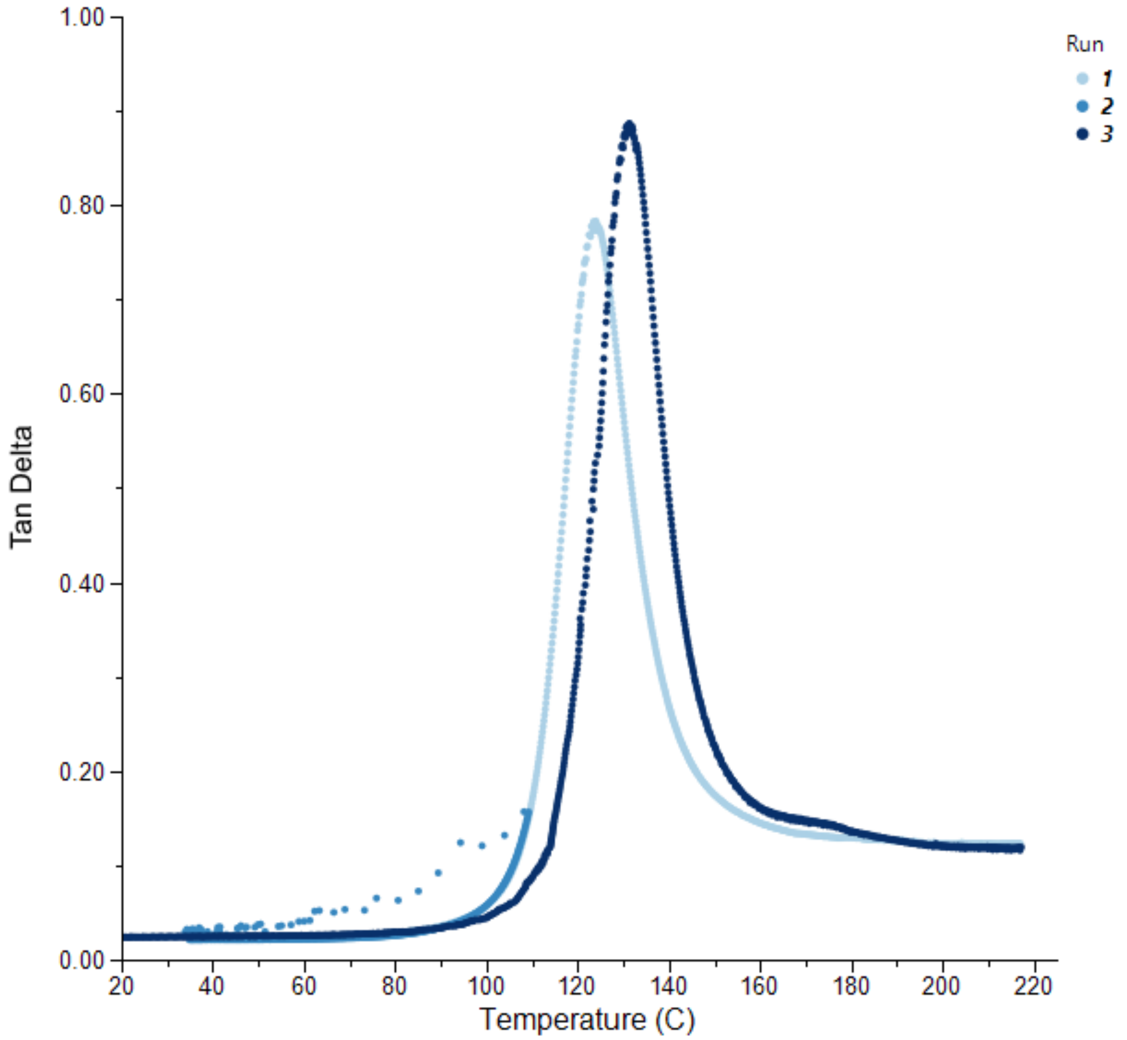

Figure S21. Tan delta traces of pDCPD doped with 10% v/v **MeSi8.**

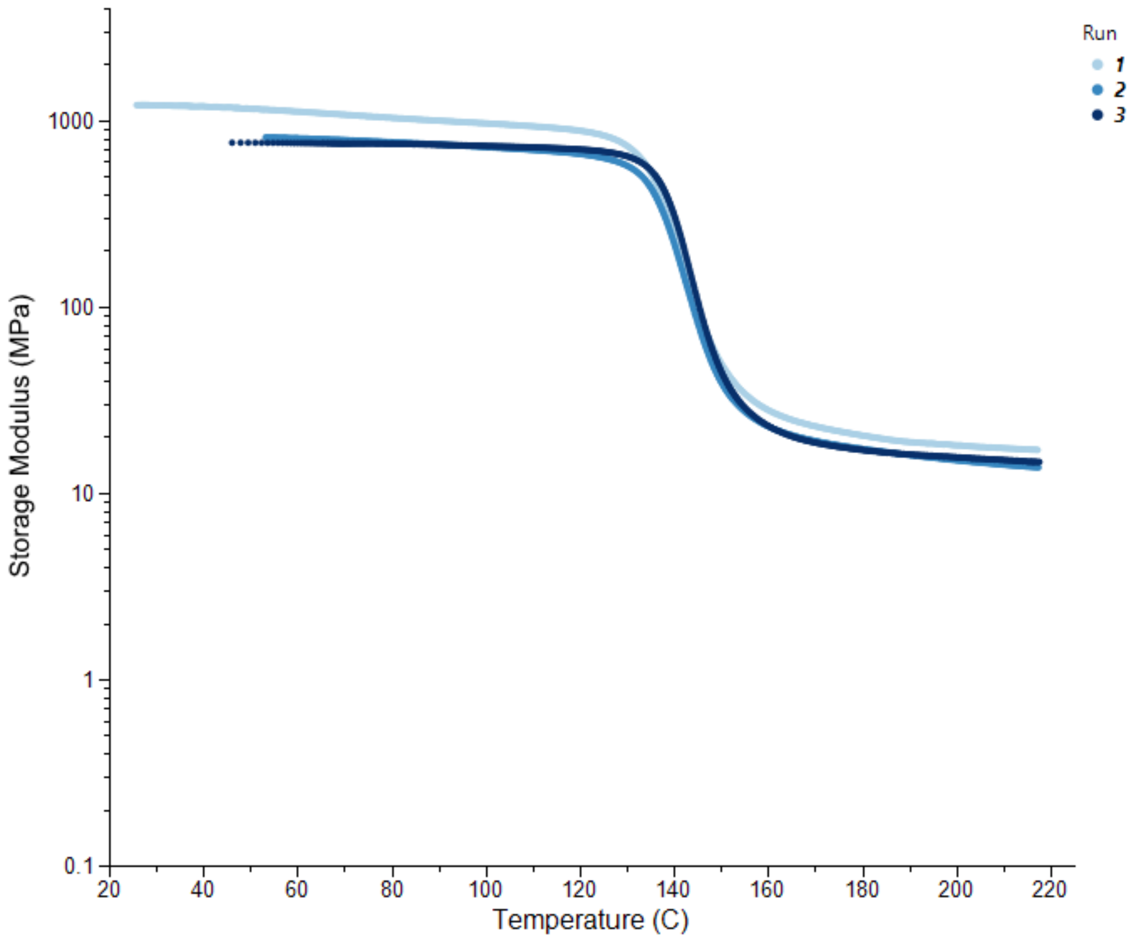

Figure S22. Storage modulus traces of pDCPD doped with 5% v/v **MeSi8**.

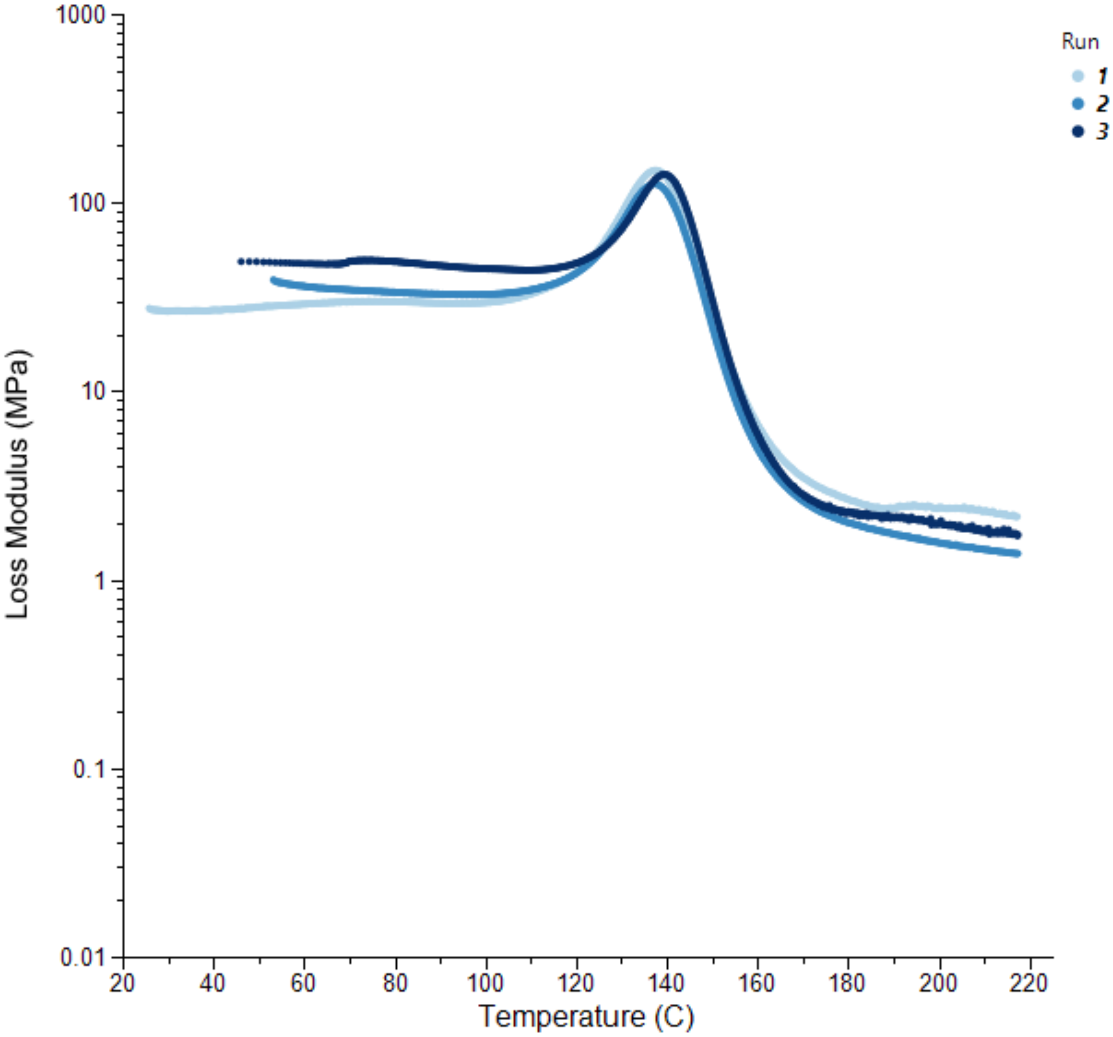

Figure S23. Loss modulus traces of pDCPD doped with 5% v/v **MeSi8**.

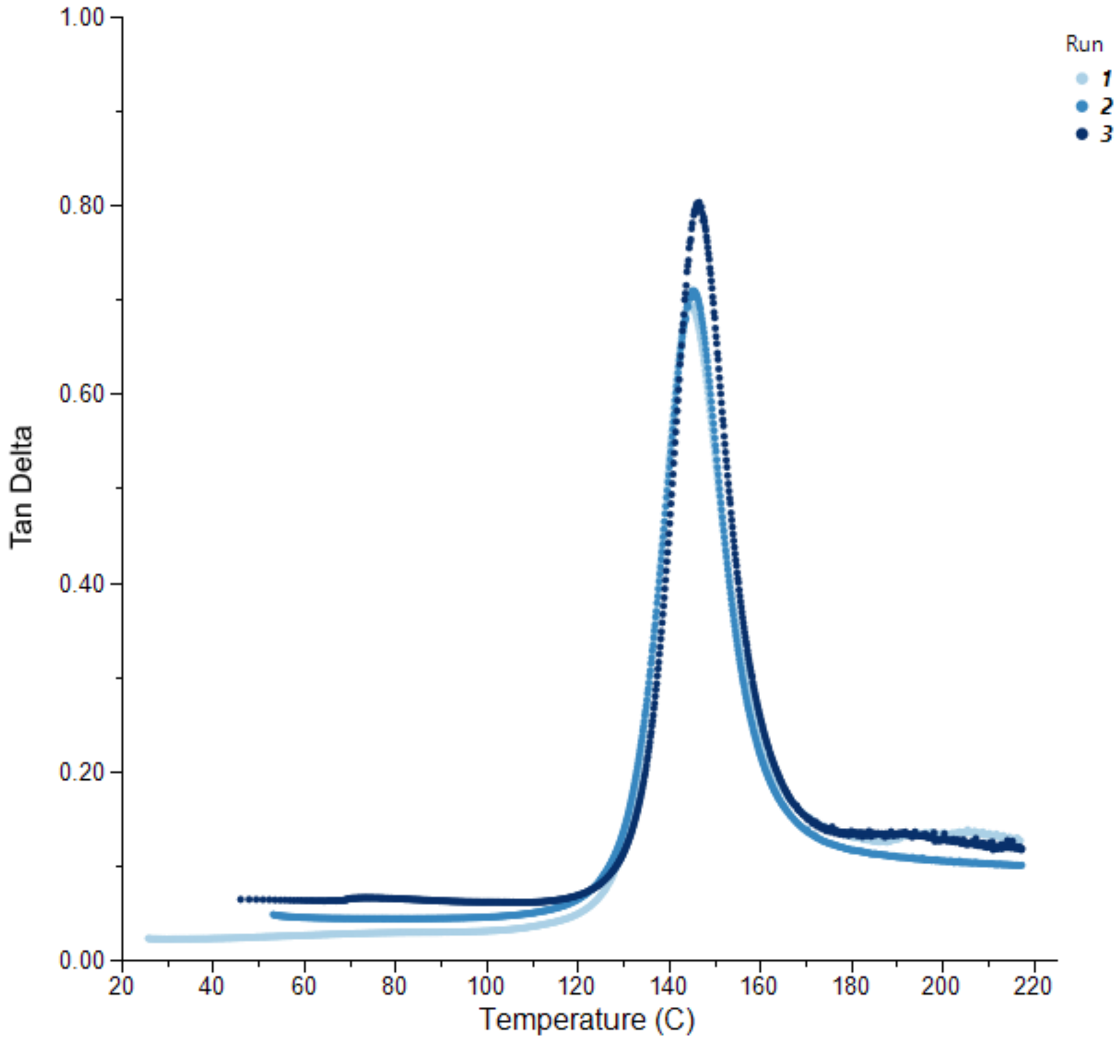

Figure S24. Tan delta traces of pDCPD doped with 5% v/v **MeSi8.**

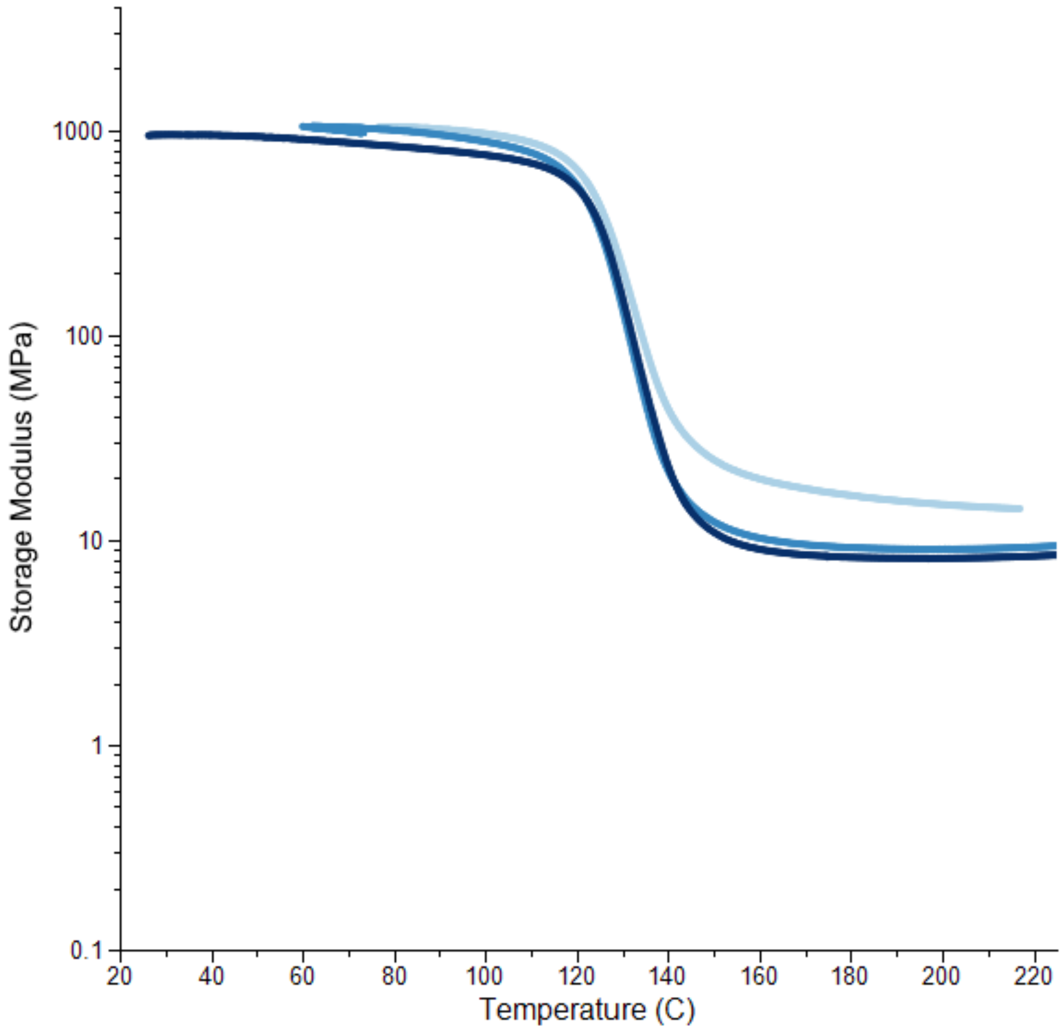

Figure S25. Storage modulus traces of pDCPD doped with 10% v/v **PhSi8.**[1]

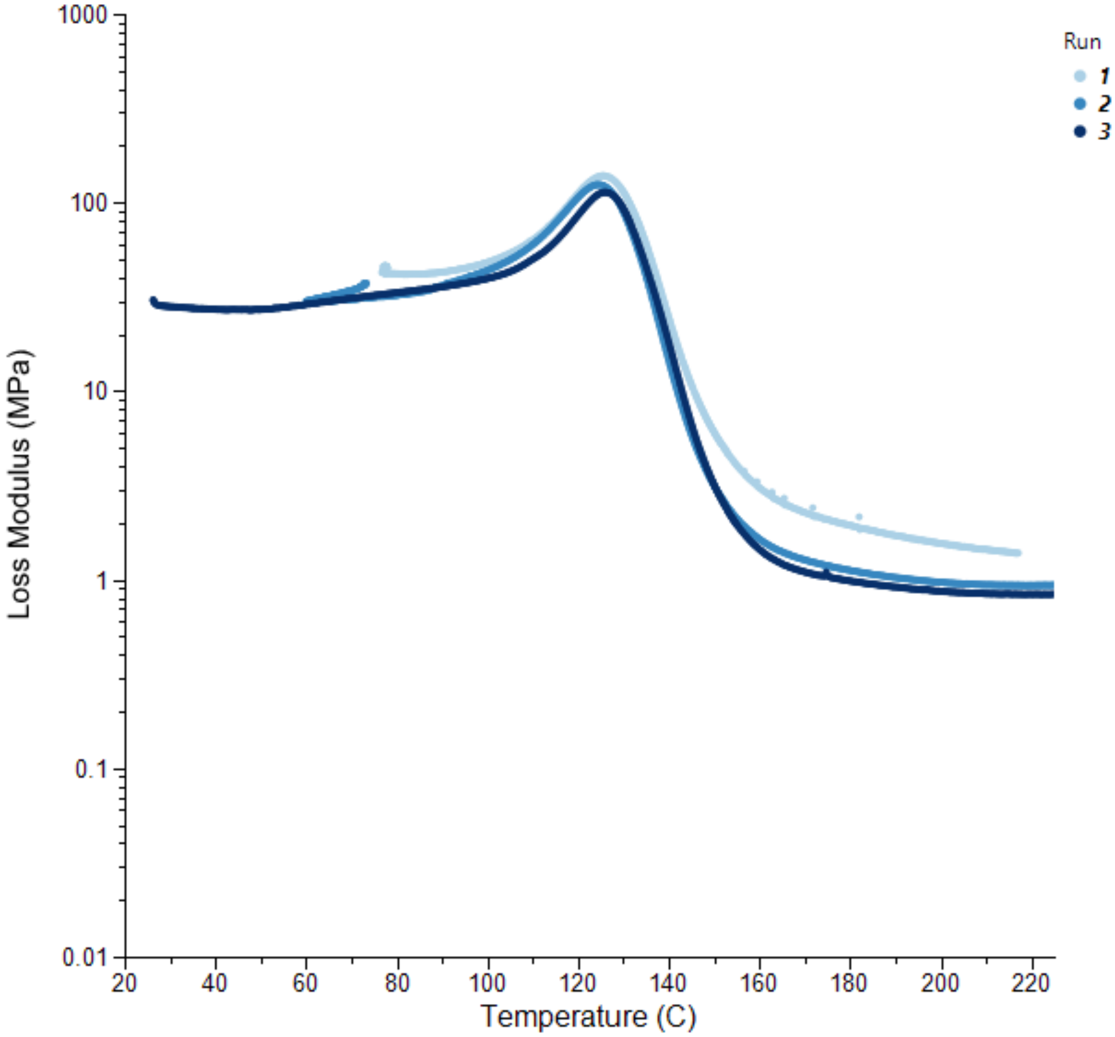

Figure S26. Loss modulus traces of pDCPD doped with 10% v/v **PhSi8.**

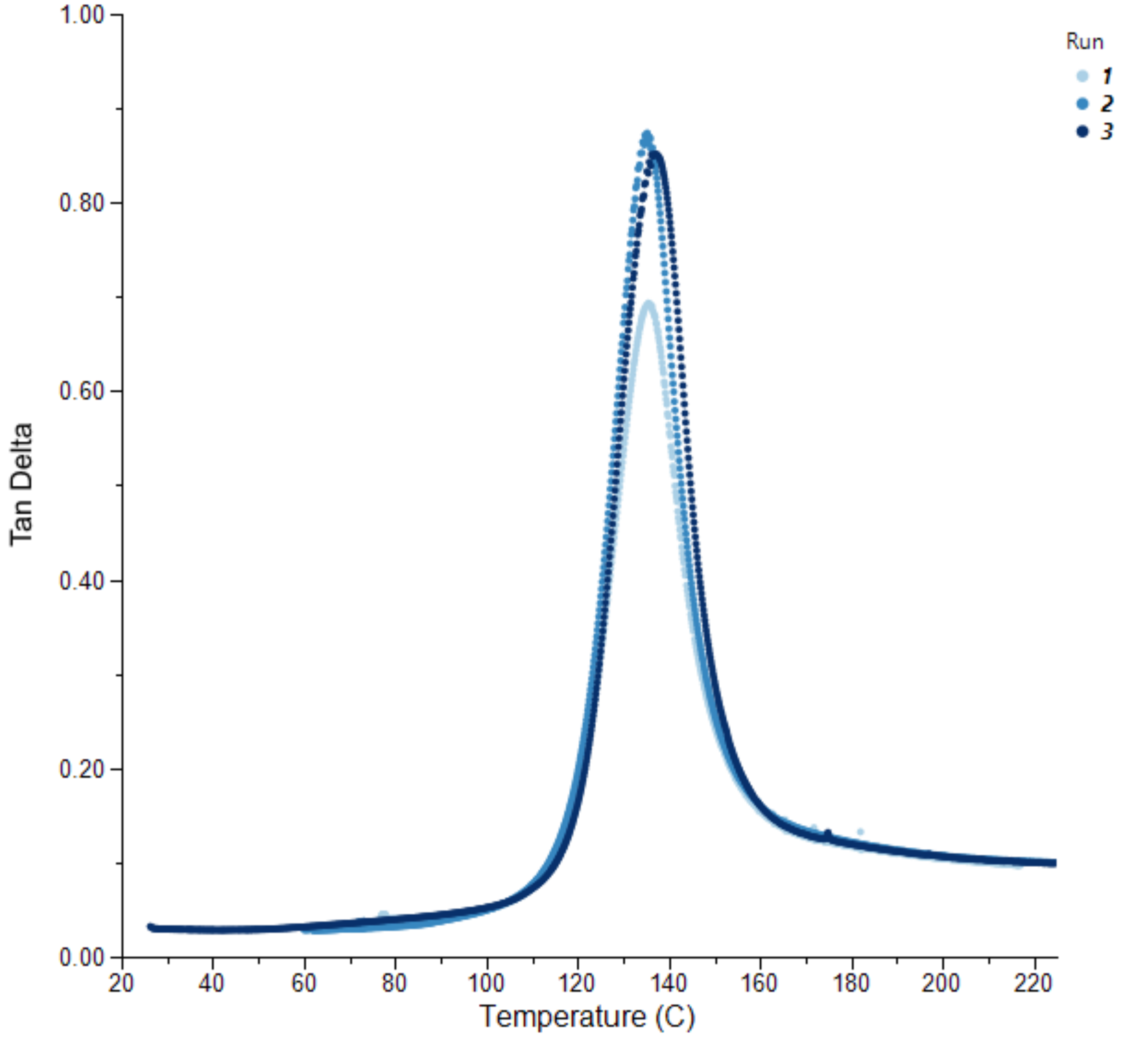

Figure S27. Tan delta traces of pDCPD doped with 10% v/v **PhSi8.**

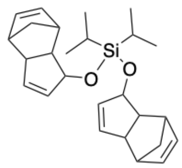

= DDi PrS i

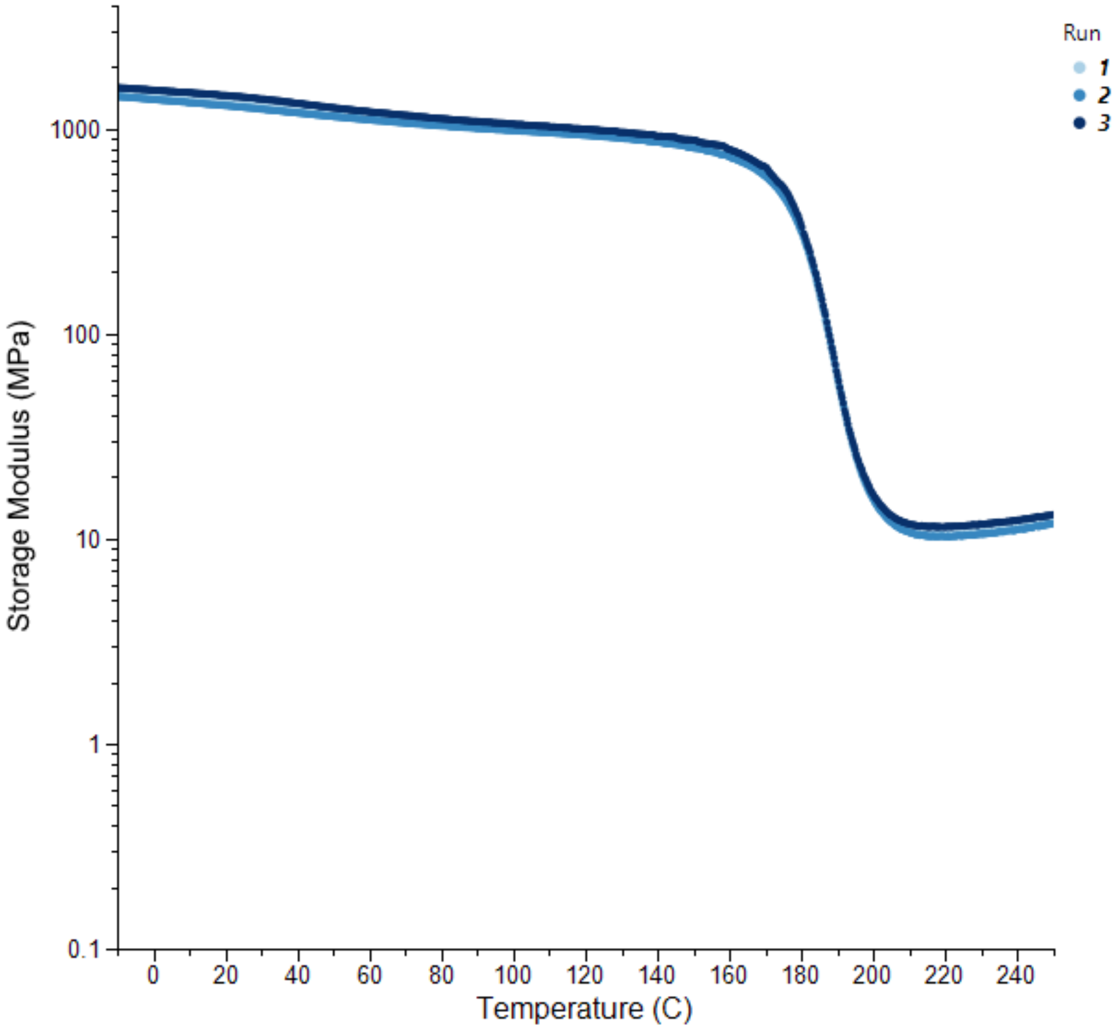

Figure S28. Storage modulus traces of pDCPD doped with 10 mol% **Difunctional-DCPD-isopropyl silyl ether** (**DDiPrSi).**

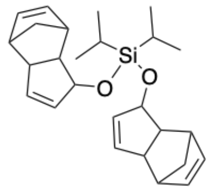

= DDi PrSi

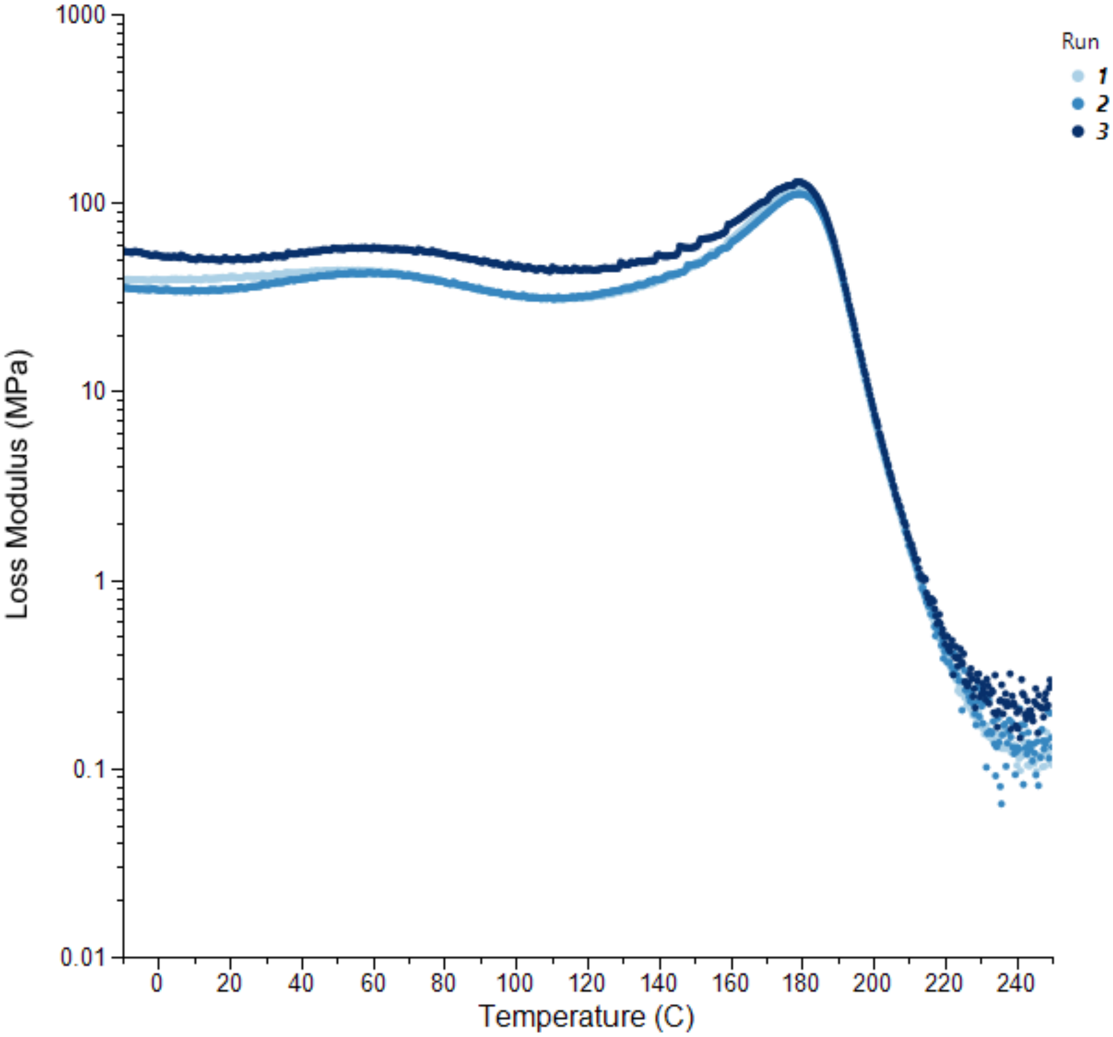

Figure S29. Loss modulus traces of pDCPD doped with 10 mol% **DDiPrSi.**

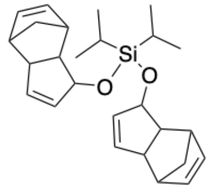

= DDiPrSi

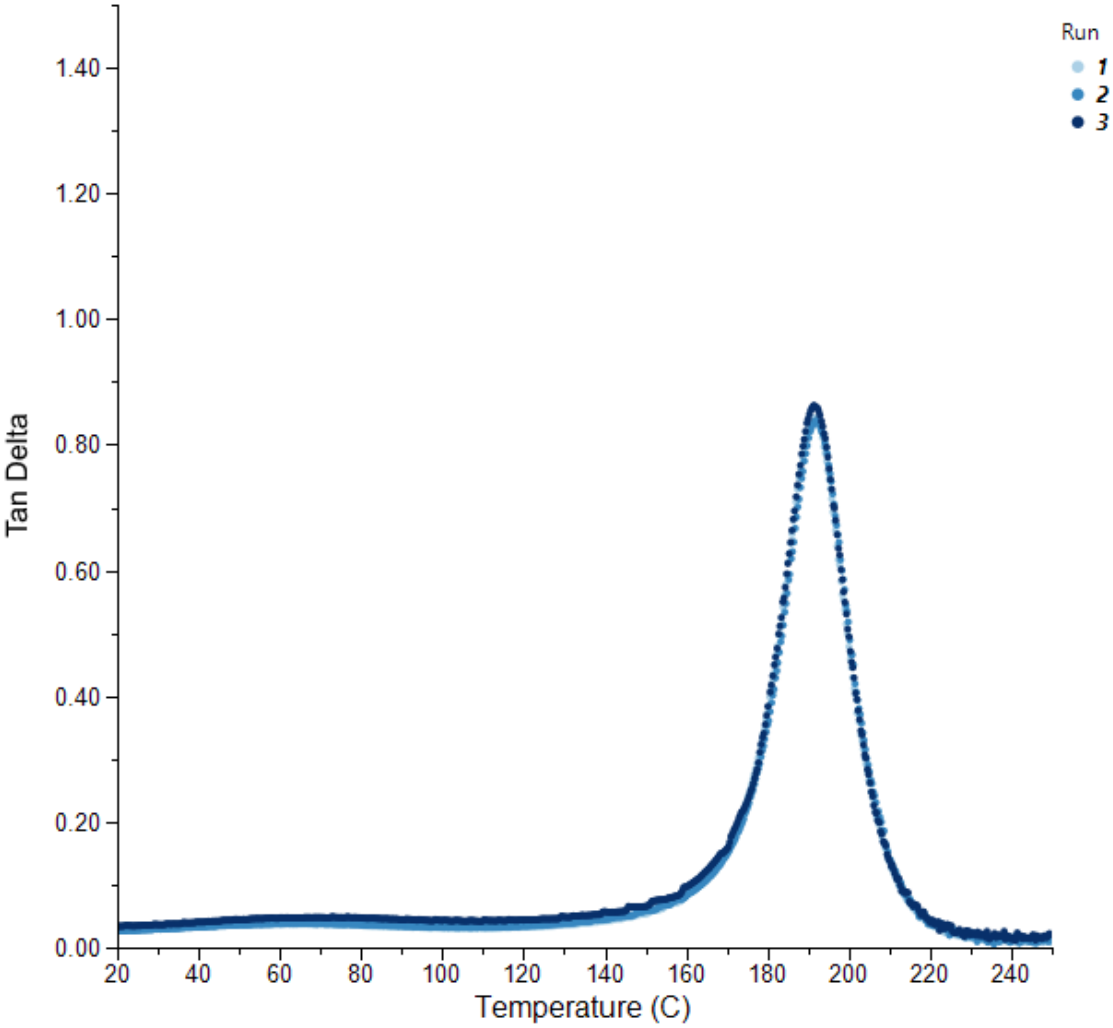

Figure S30. Tan delta traces of pDCPD doped with 10 mol% **DDiPrSi**.

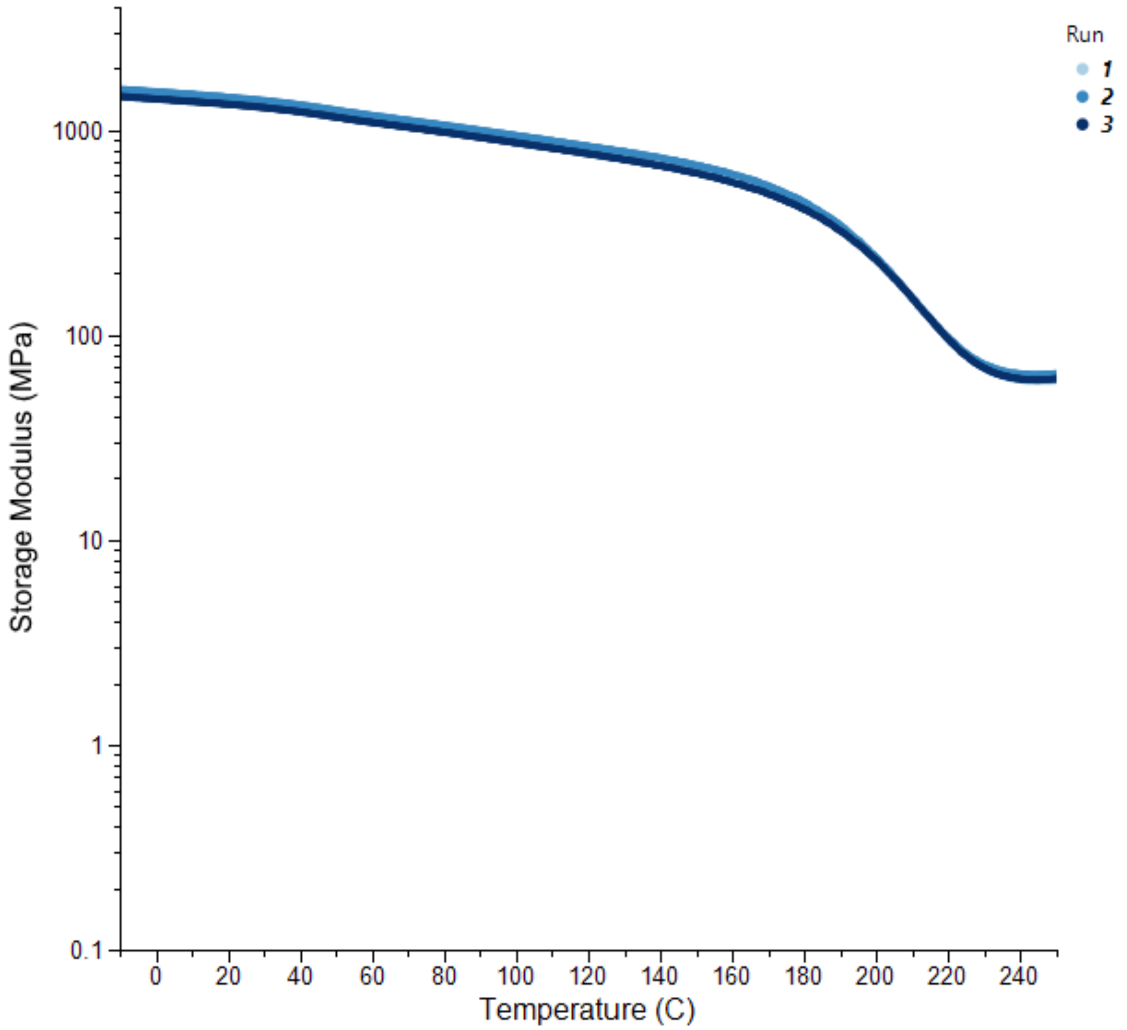

Figure S31. Storage modulus traces of pDCPD doped with 5 mol% **iPrSi8**,[1] 25 mol% short-chain ester (**SCE**).[9]

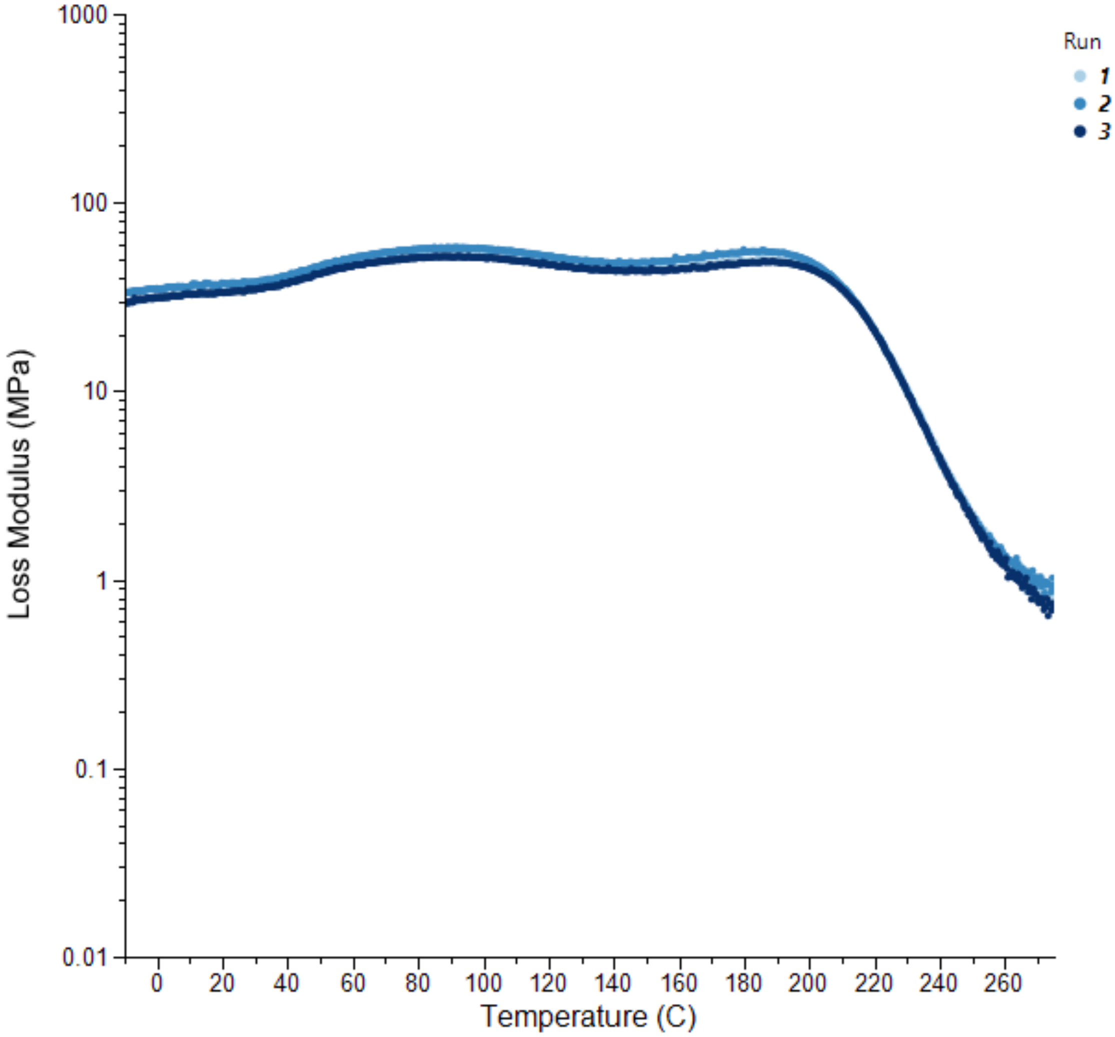

Figure S32. Loss modulus traces of pDCPD doped with 5 mol% **iPrSi8**, 25 mol% **SCE**.

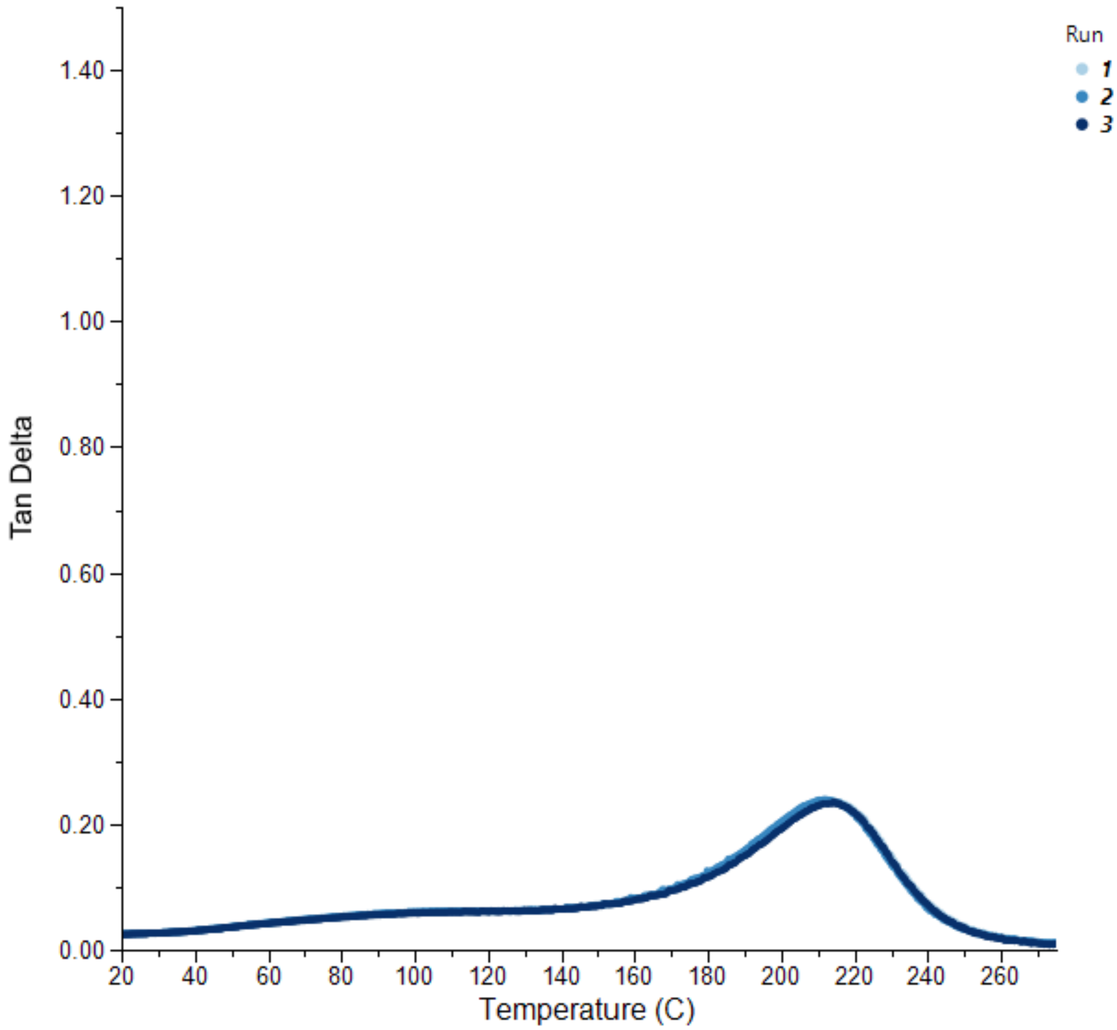

Figure S33. Tan delta traces of pDCPD doped with 5 mol% **iPrSi8**, 25 mol% **SCE**.

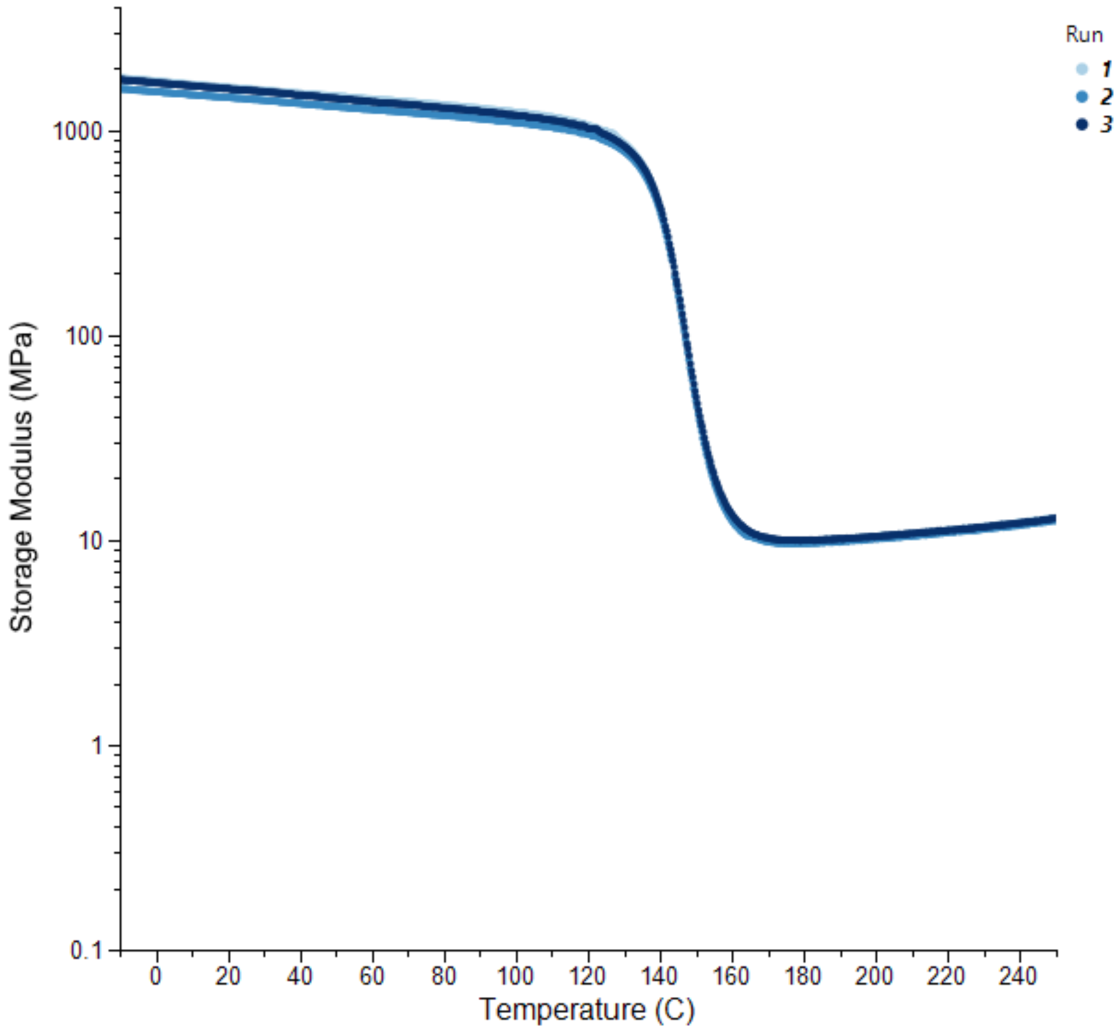

Figure S34. Storage modulus traces of pDCPD doped with 5 mol% **iPrSi8**, 5 mol% **SCE**.

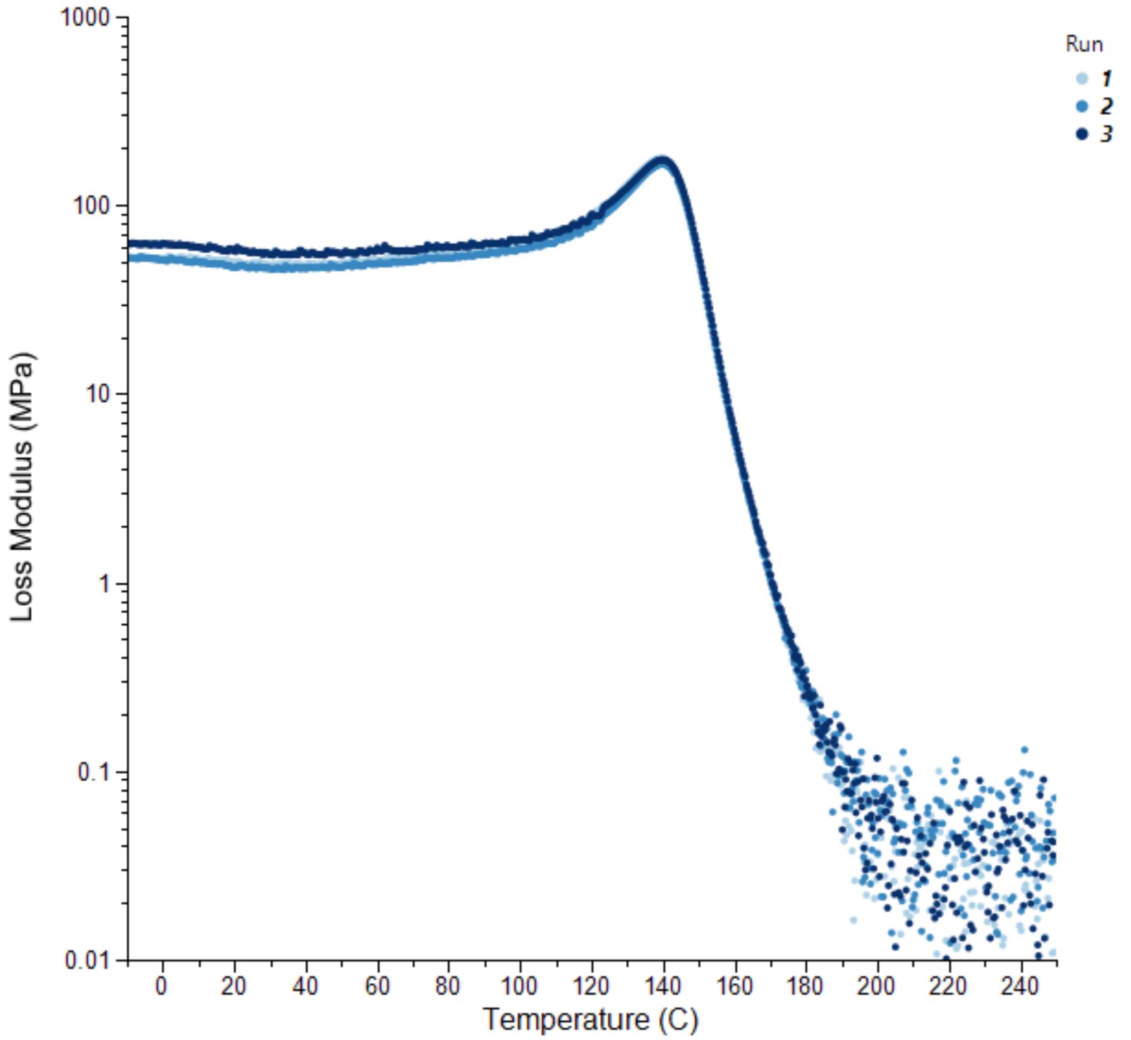

Figure S35. Loss modulus traces of pDCPD doped with 5 mol% **iPrSi8**, 5 mol% **SCE**.

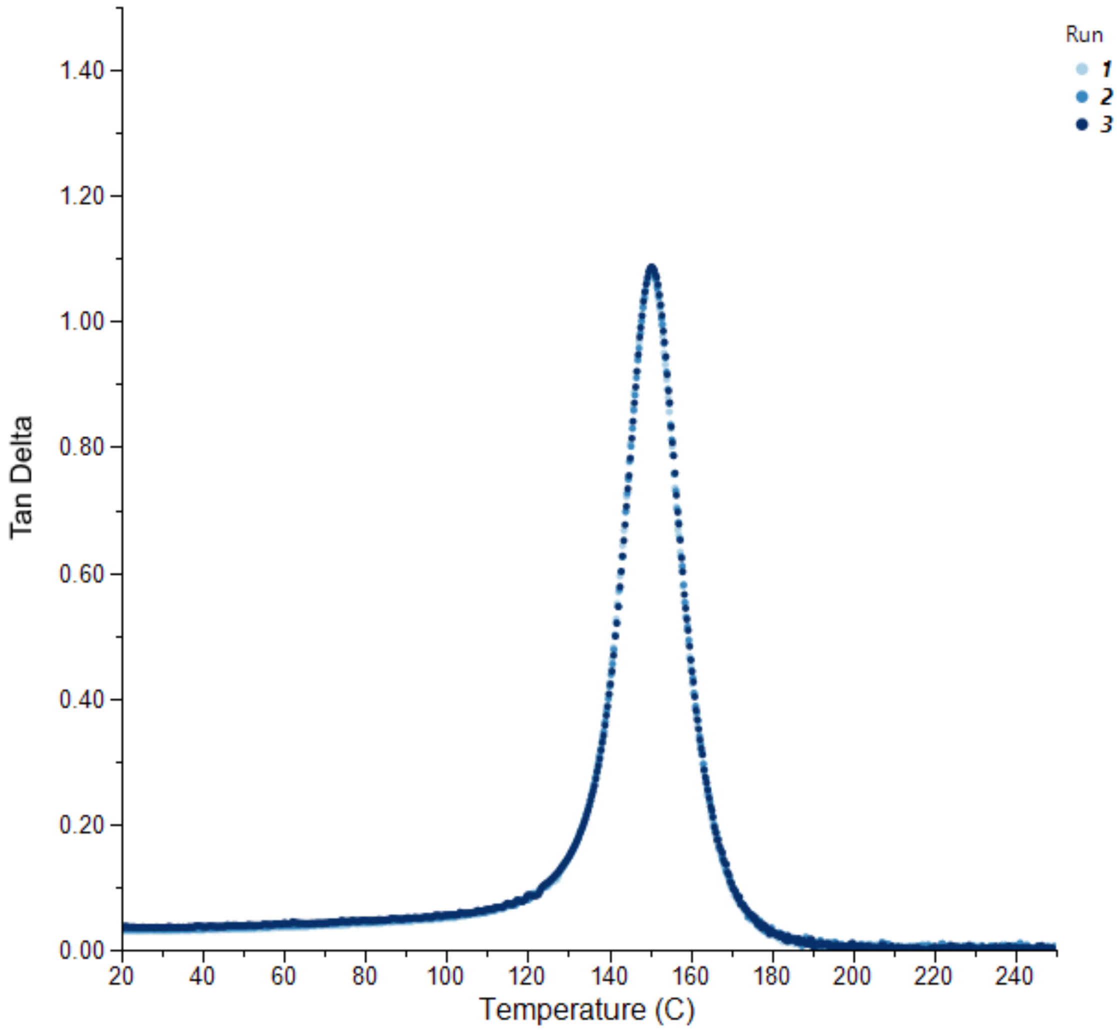

Figure S36. Tan delta traces of pDCPD doped with 5 mol% **iPrSi8**, 5 mol% **SCE**.

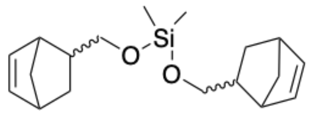

= DN MS

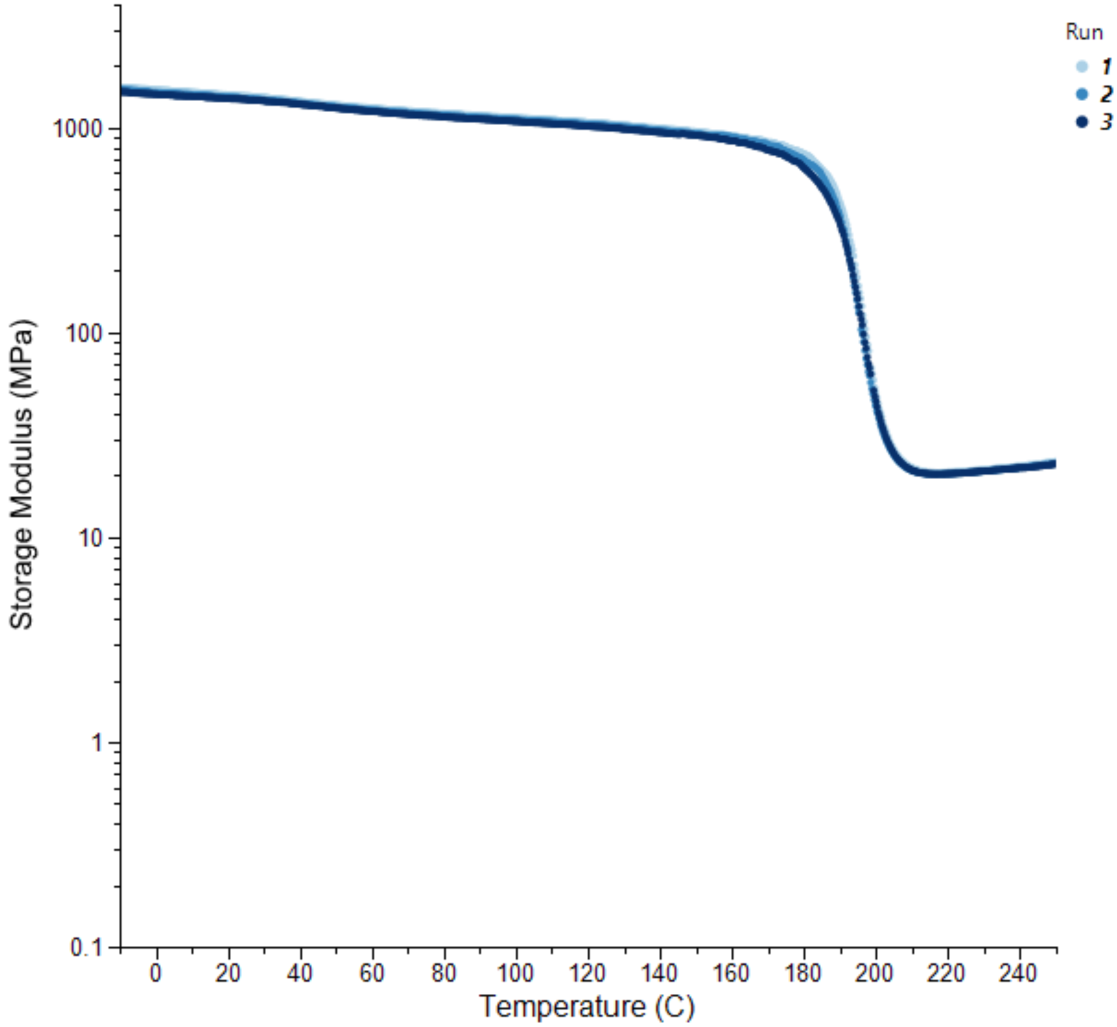

Figure S37. Storage modulus traces of pDCPD doped with 10 mol% **DNMS**, short oven cure.

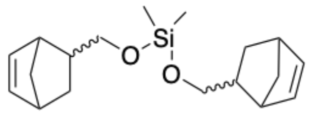

= DNMS

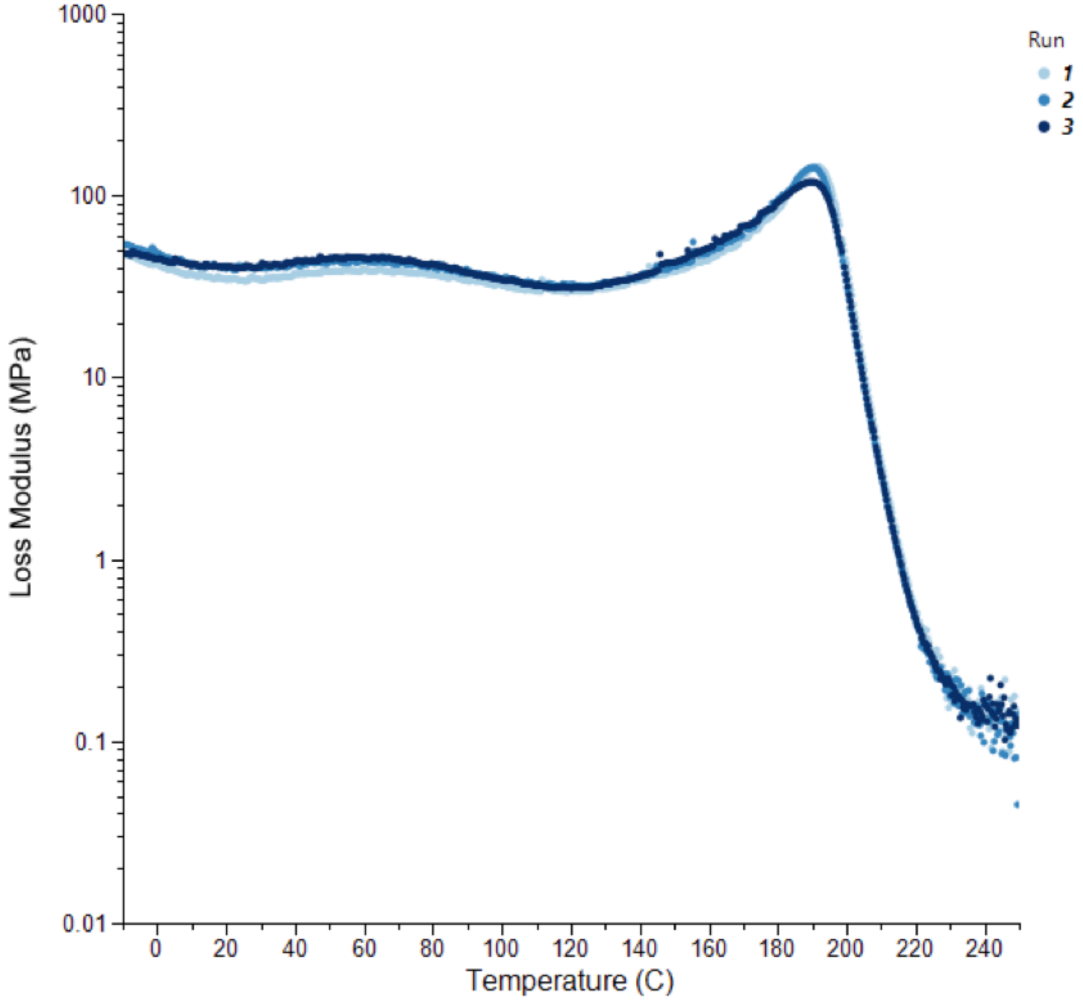

Figure S38. Loss modulus traces of pDCPD doped with 10 mol% **DNMS**, short oven cure.

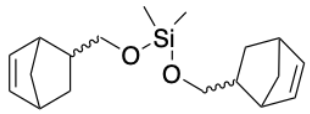

=
DN
MS

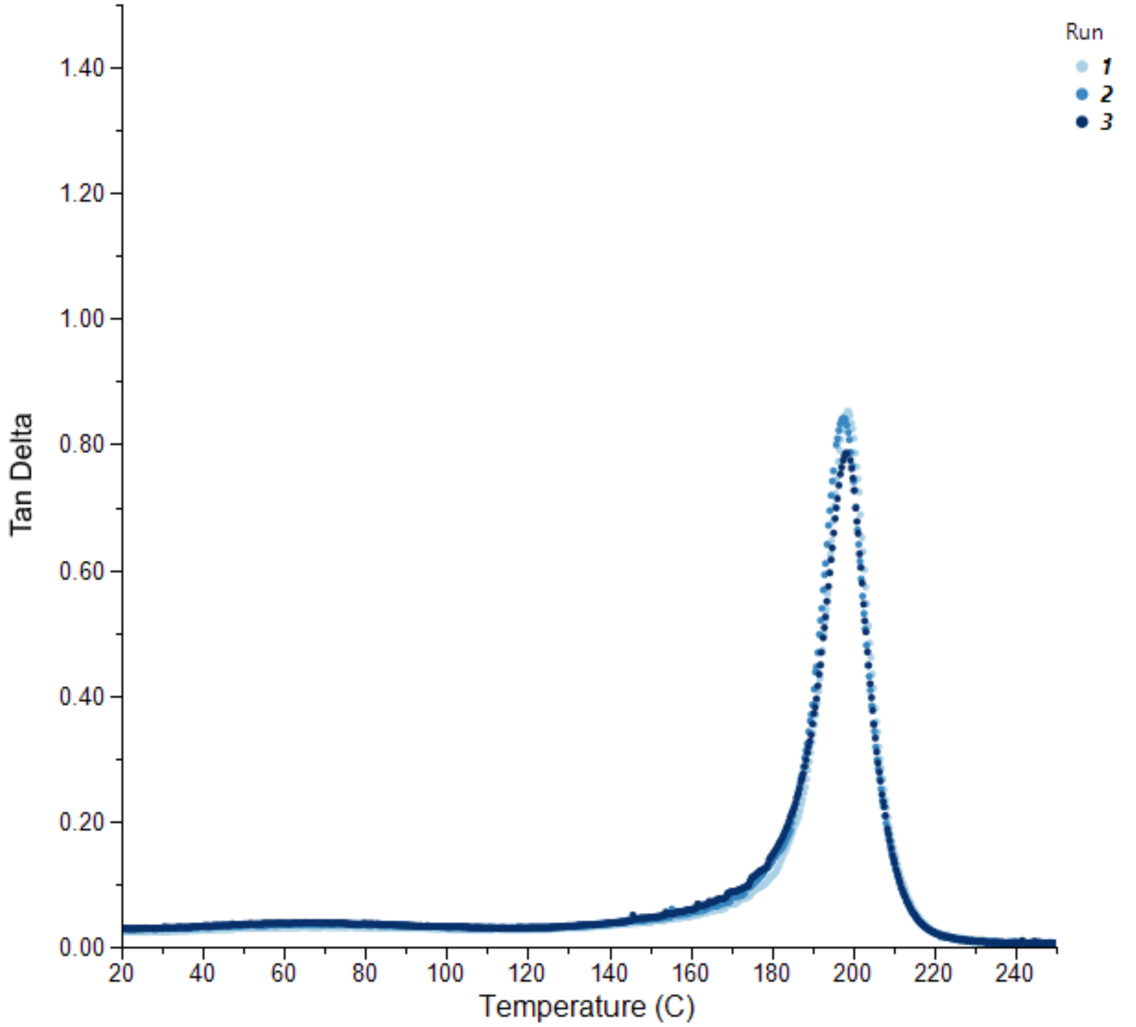

Figure S39. Tan delta traces of pDCPD doped with 10 mol% **DNMS**, short oven cure.

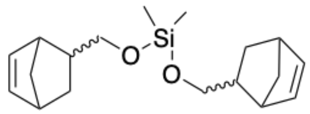

= DN MS

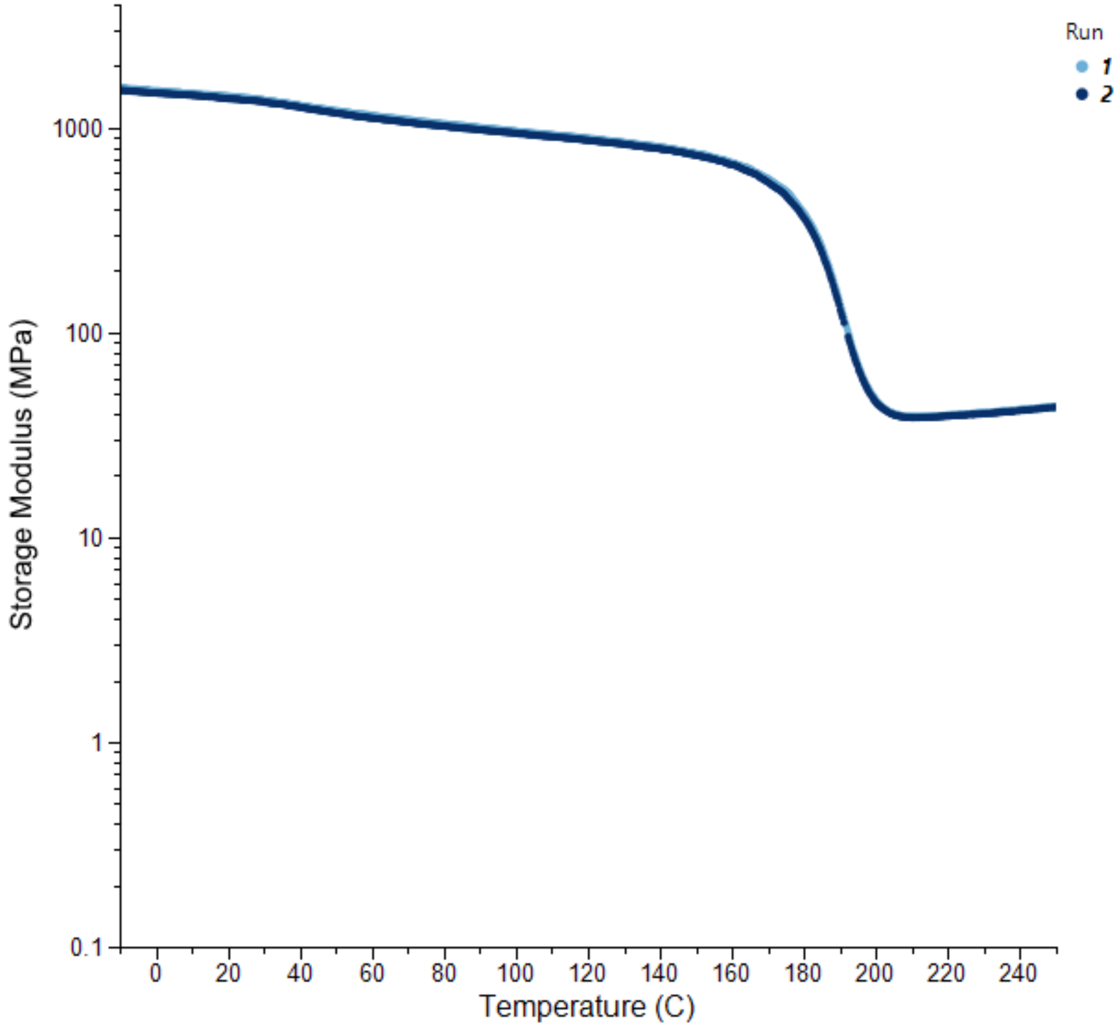

Figure S40. Storage modulus traces of pDCPD doped with 20 mol% **DNMS**, FROMP cure. All FROMP samples prepared in accordance with methods reported in the literature.[9]

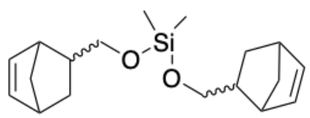

=
DN
MS

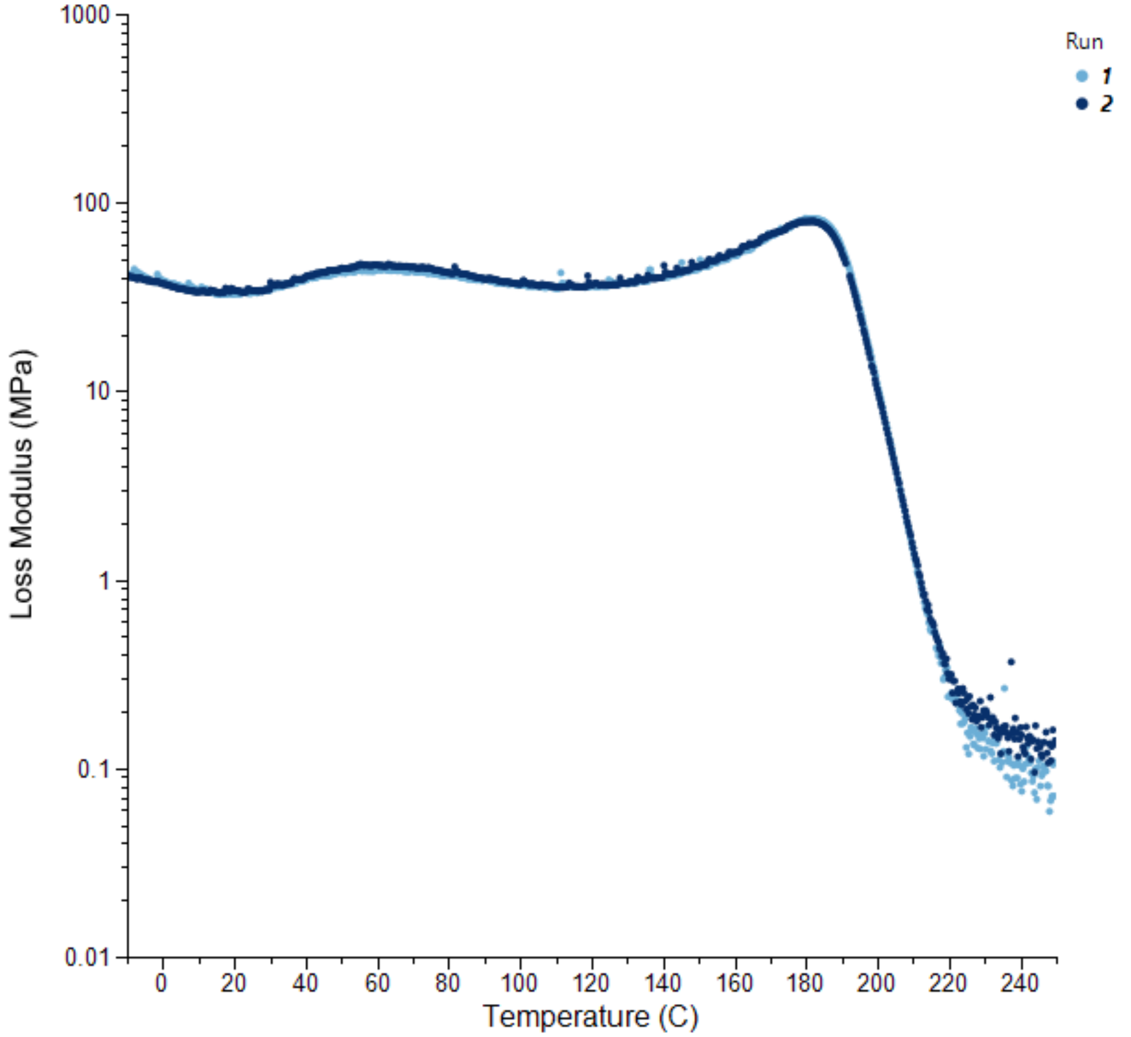

Figure S41. Loss modulus traces of pDCPD doped with 20 mol% **DNMS**, FROMP cure**.**

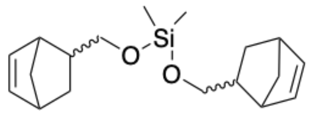

= DN MS

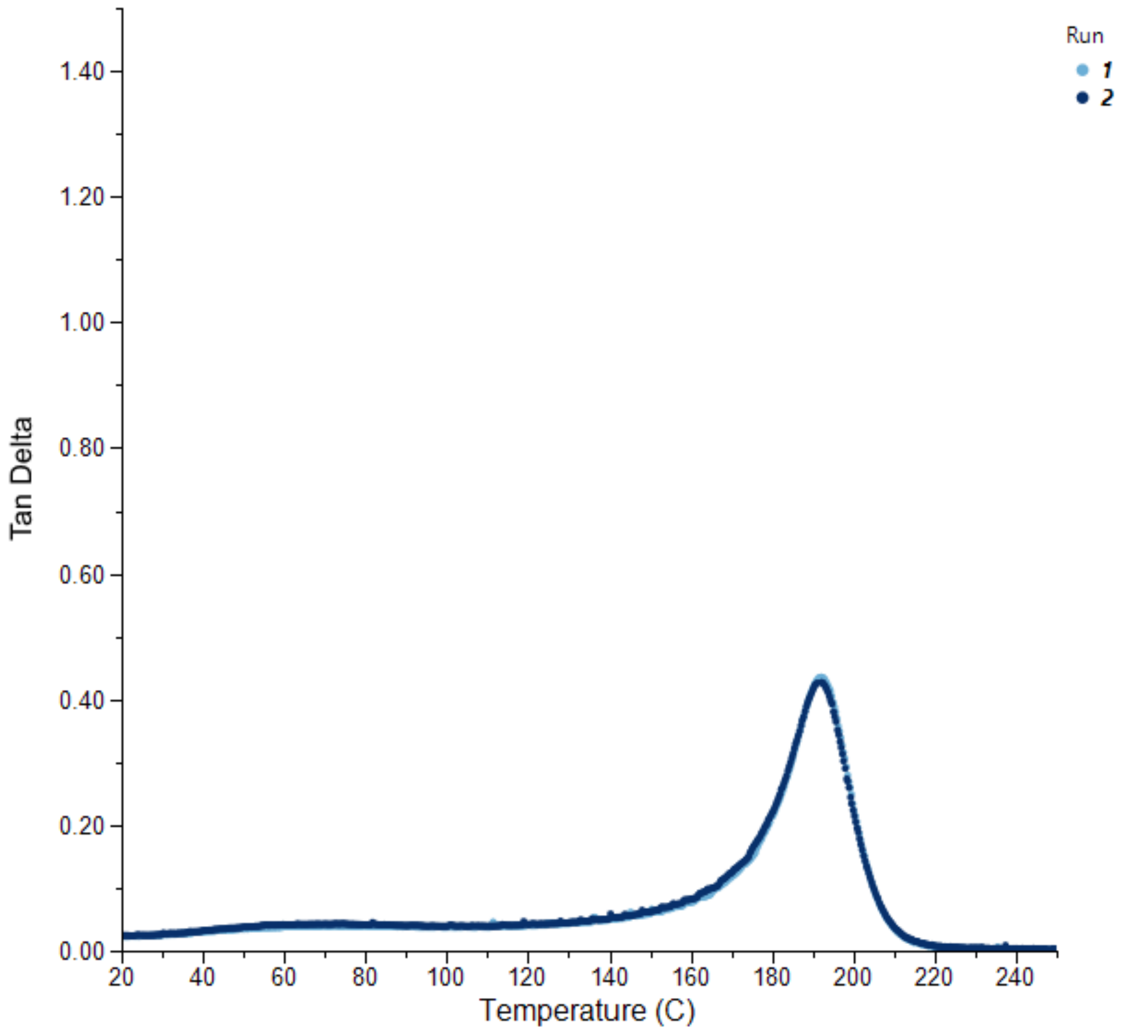

Figure S42. Tan delta traces of pDCPD doped with 20 mol% **DNMS**, FROMP cure.

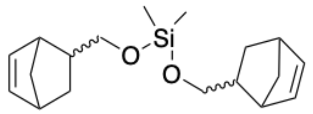

=
DN
MS

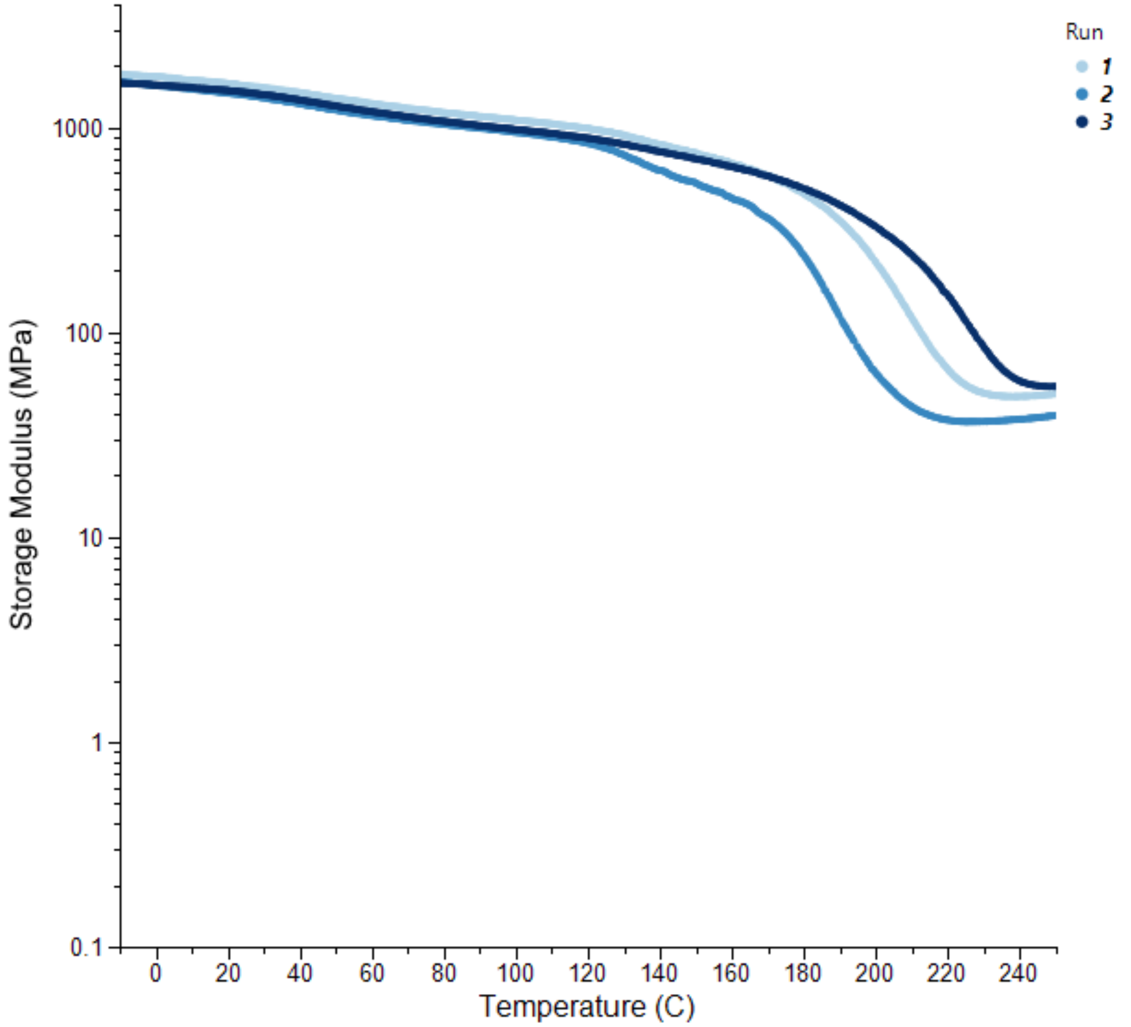

Figure S43. Storage modulus traces of pDCPD doped with 20 mol% **DNMS**, short oven cure.

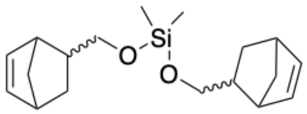

=
DN
MS

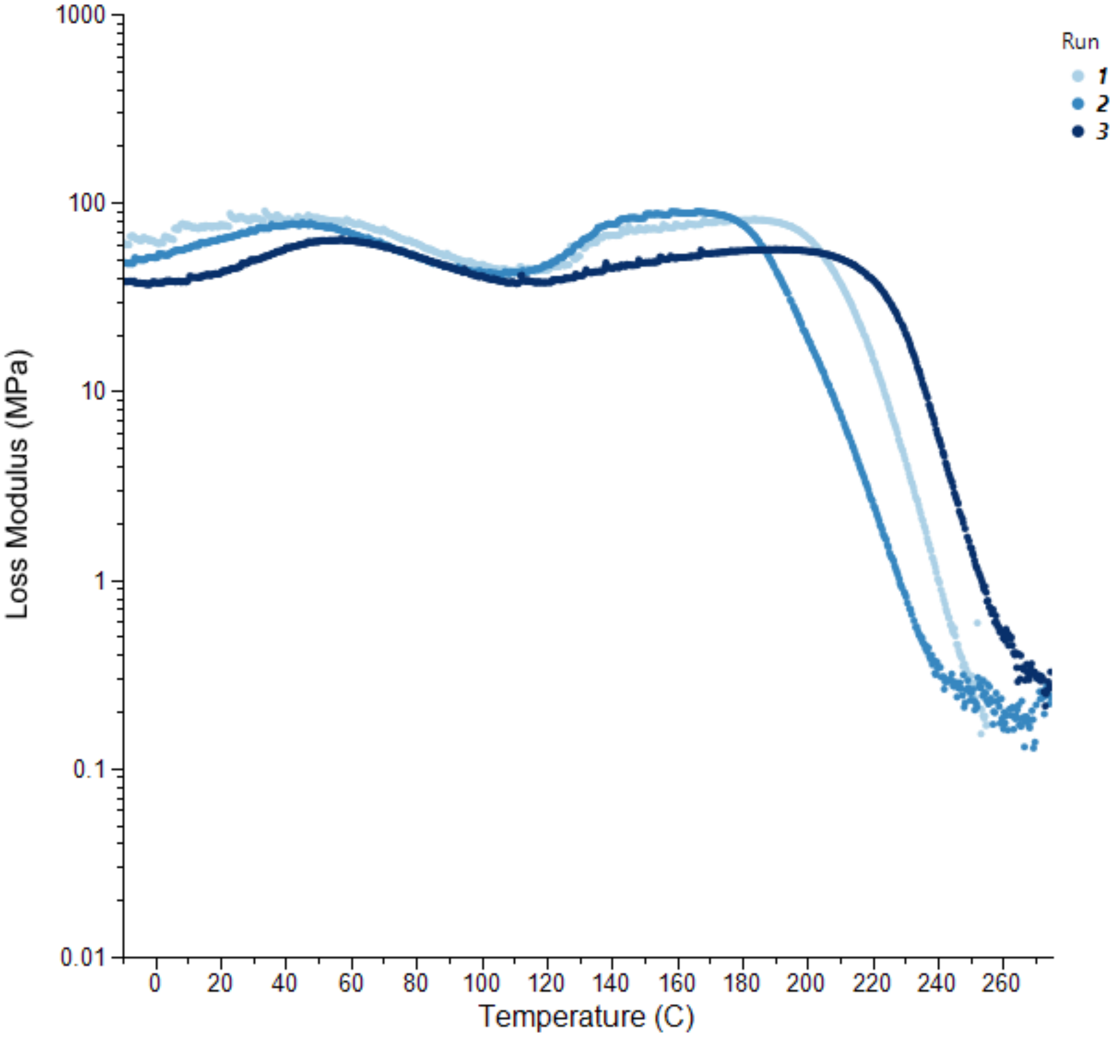

Figure S44. Loss modulus traces of pDCPD doped with 20 mol% **DNMS**, short oven cure.

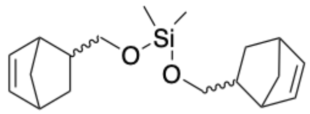

=
DN
MS

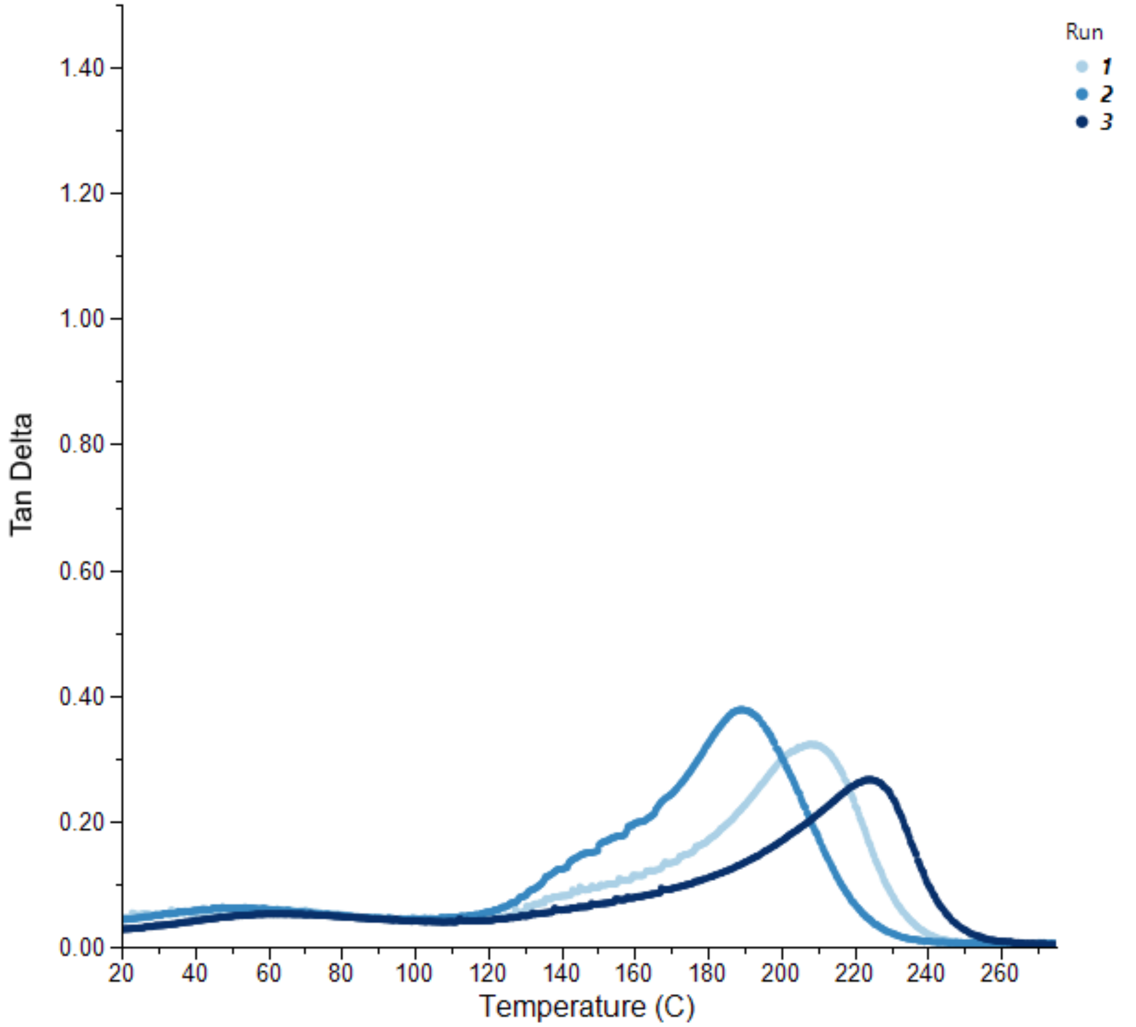

Figure S45. Tan delta traces of pDCPD doped with 20 mol% **DNMS**, short oven cure.

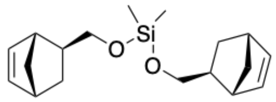

=
exoD
NMS

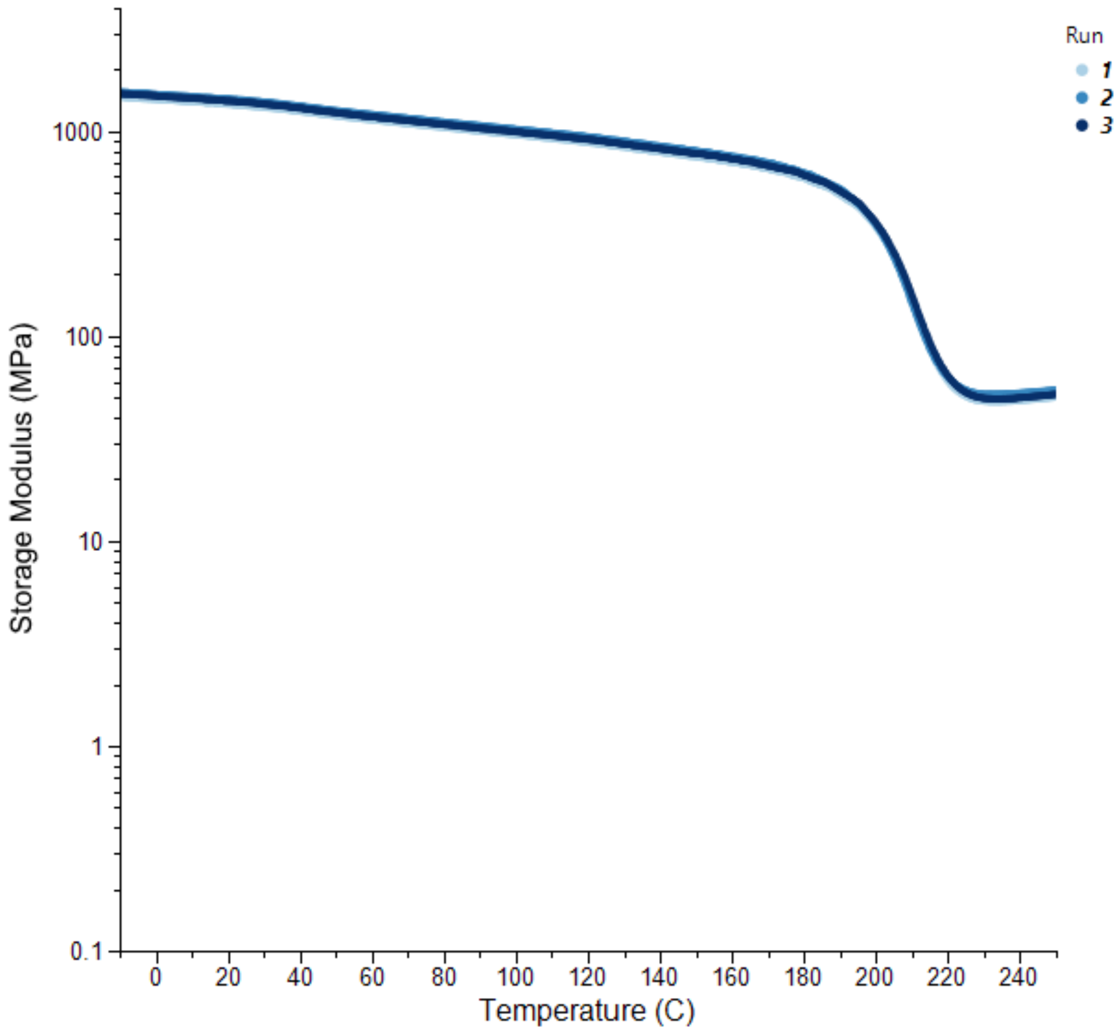

Figure S46. Storage modulus traces of pDCPD doped with 20 mol% **exoDNMS**, FROMP cure.

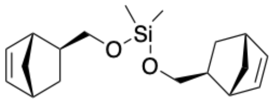

=
exoD
NMS

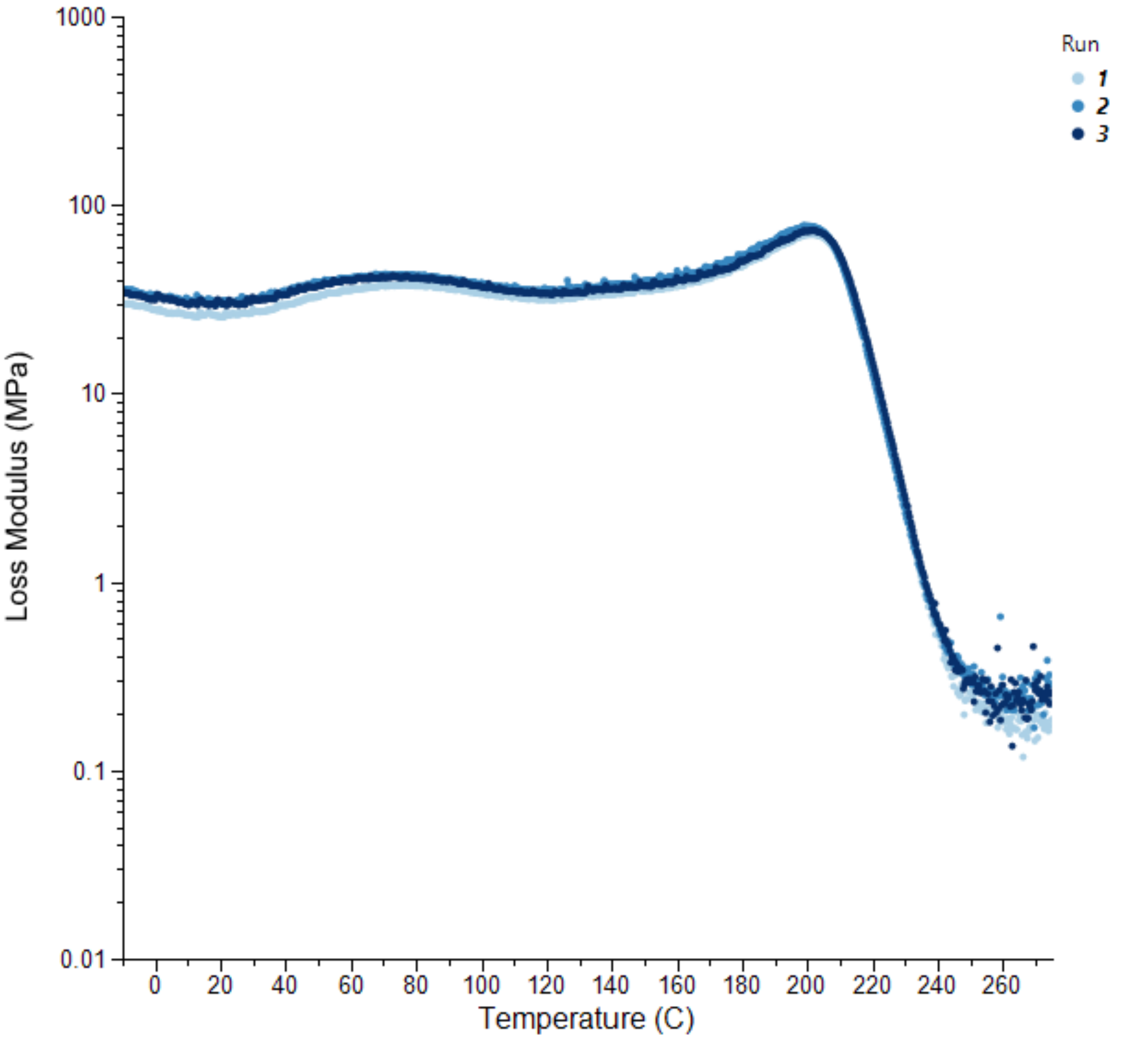

Figure S47. Loss modulus traces of pDCPD doped with 20 mol% **exoDNMS**, FROMP cure.

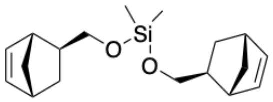

= exoD NMS

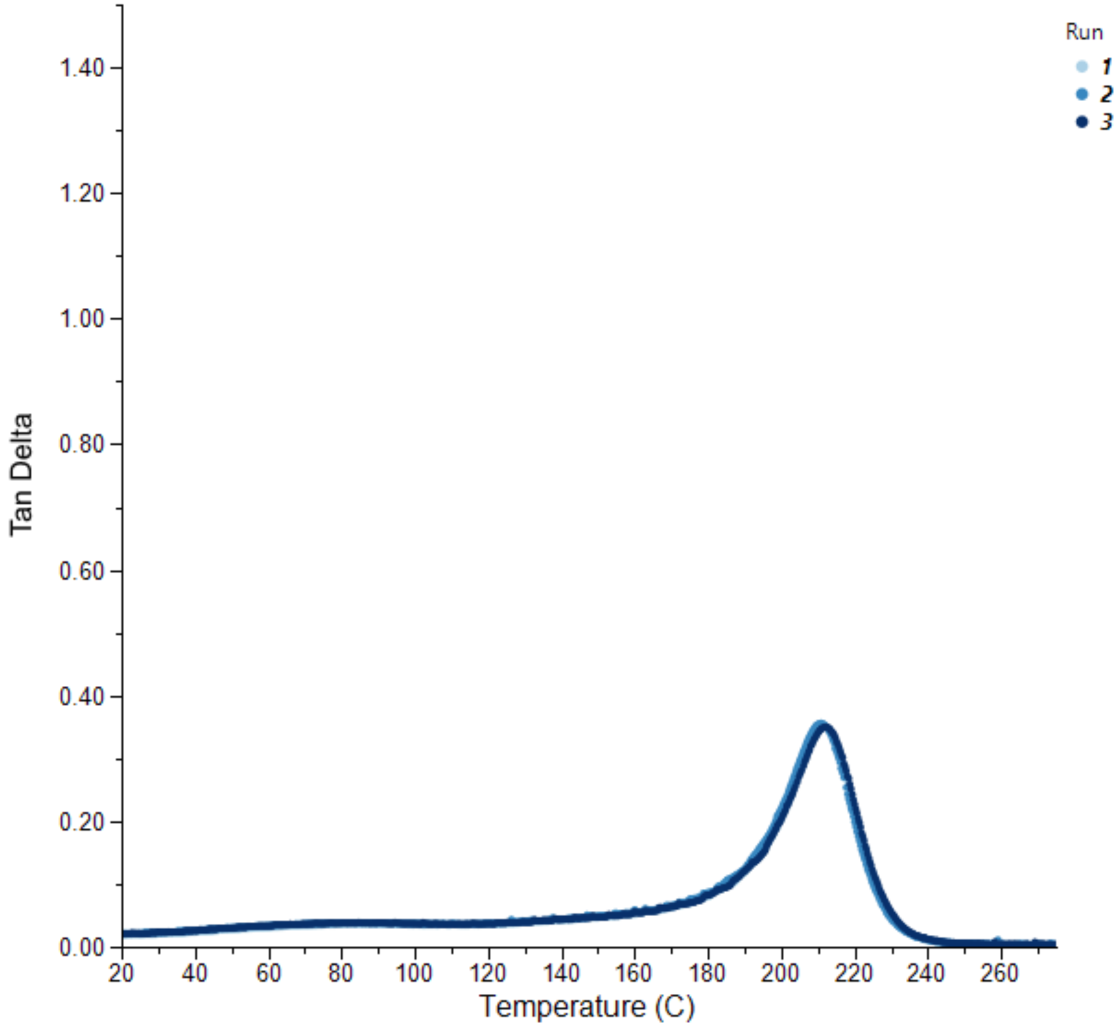

Figure S48. Tan delta traces of pDCPD doped with 20 mol% **exoDNMS**, FROMP cure.

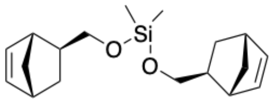

= exoDNMS

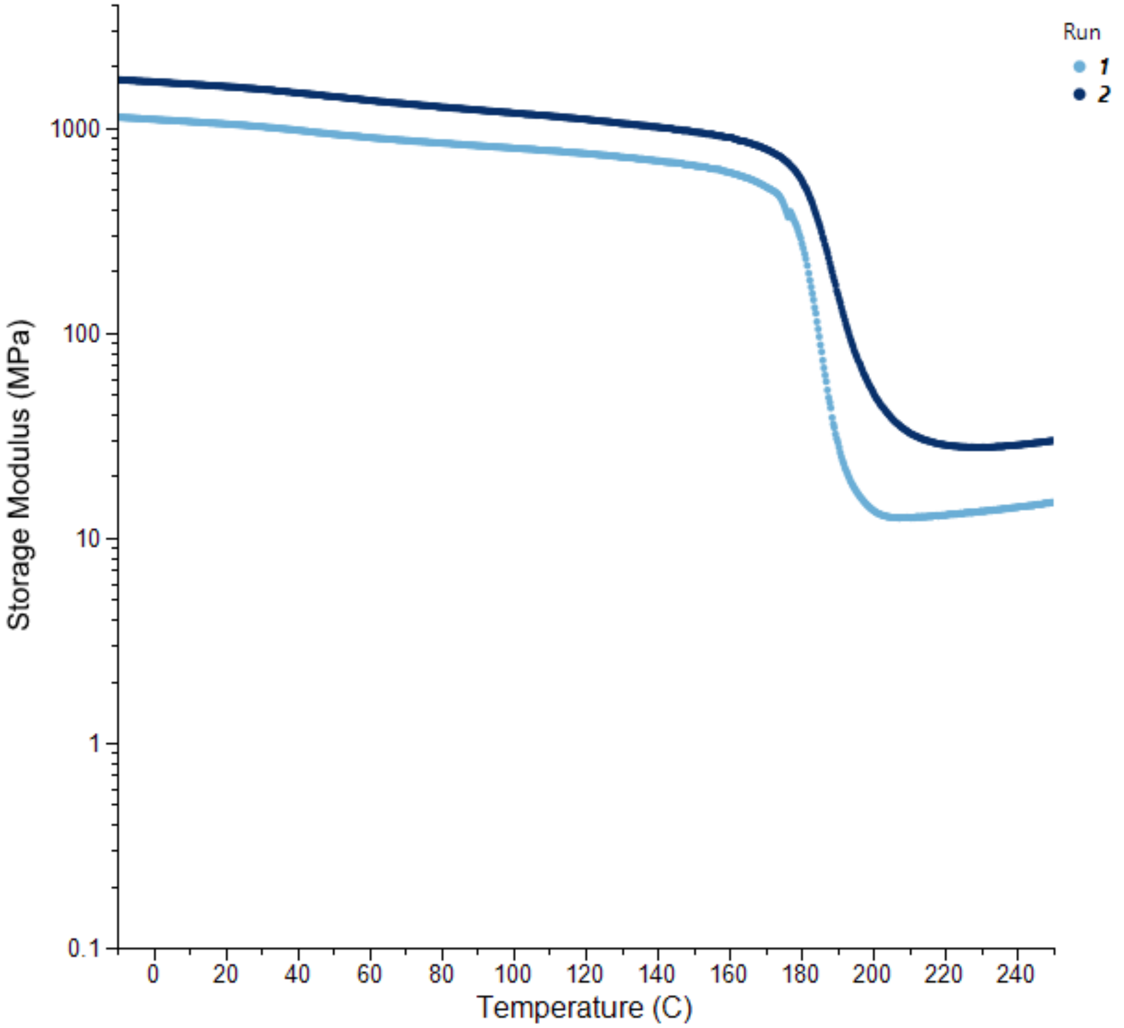

Figure S49. Storage modulus traces of pDCPD doped with 10 mol% **exoDNMS**, short oven cure.

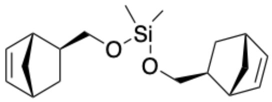

=
exoD
NMS

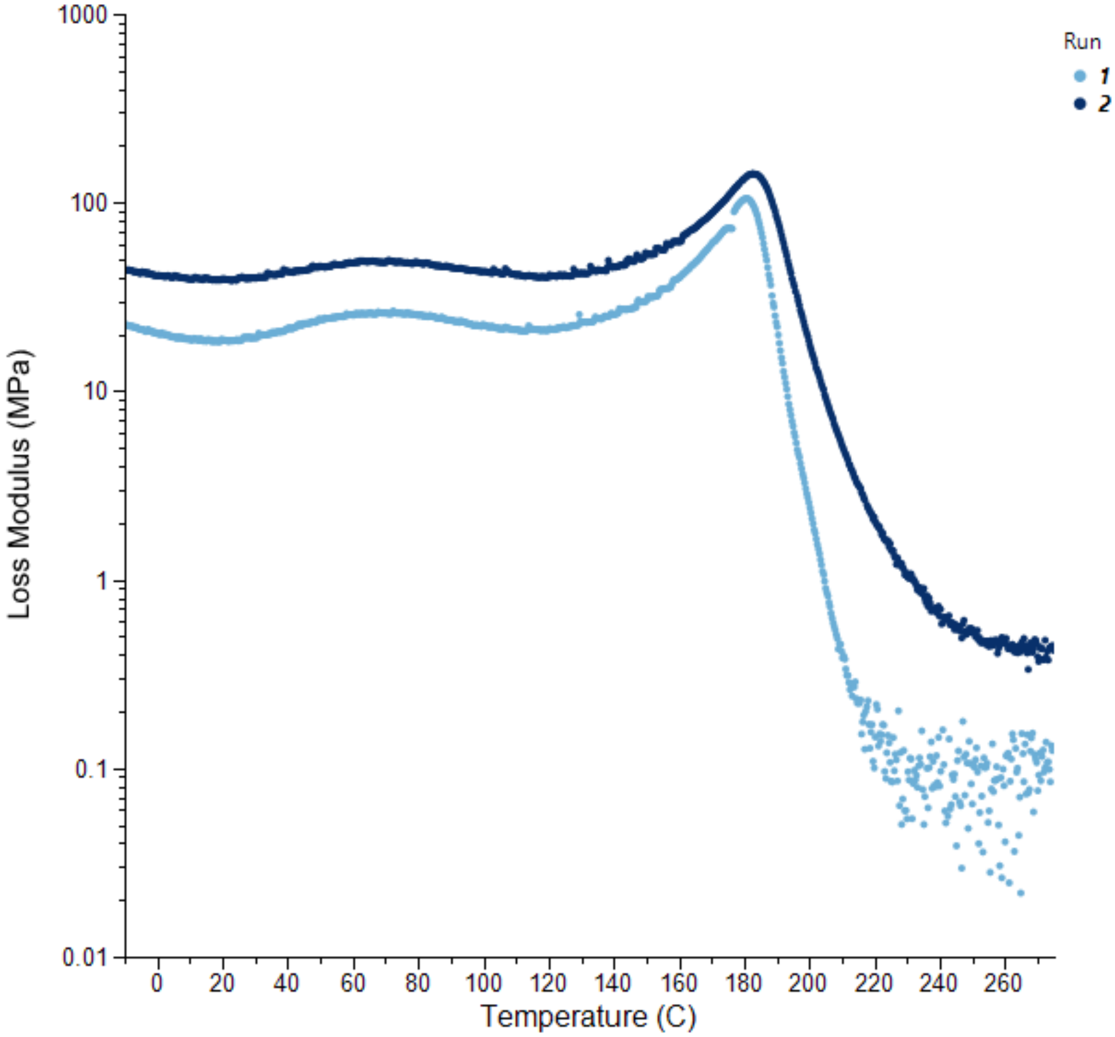

Figure S50. Loss modulus traces of pDCPD doped with 10 mol% **exoDNMS**, short oven cure.

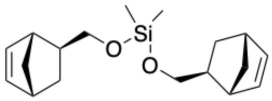

=
exoD
NMS

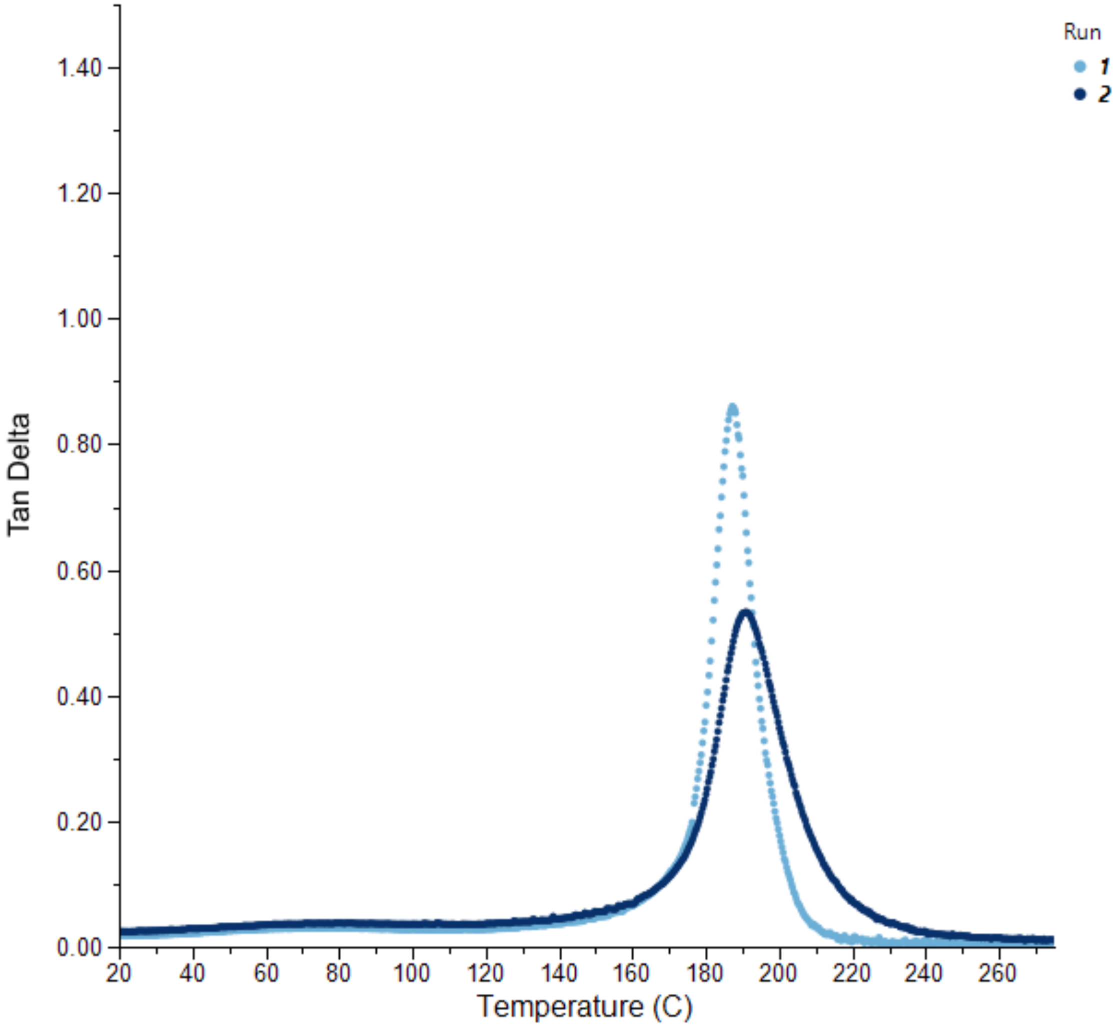

Figure S51. Tan delta traces of pDCPD doped with 10 mol% short **exoDNMS**, short oven cure.

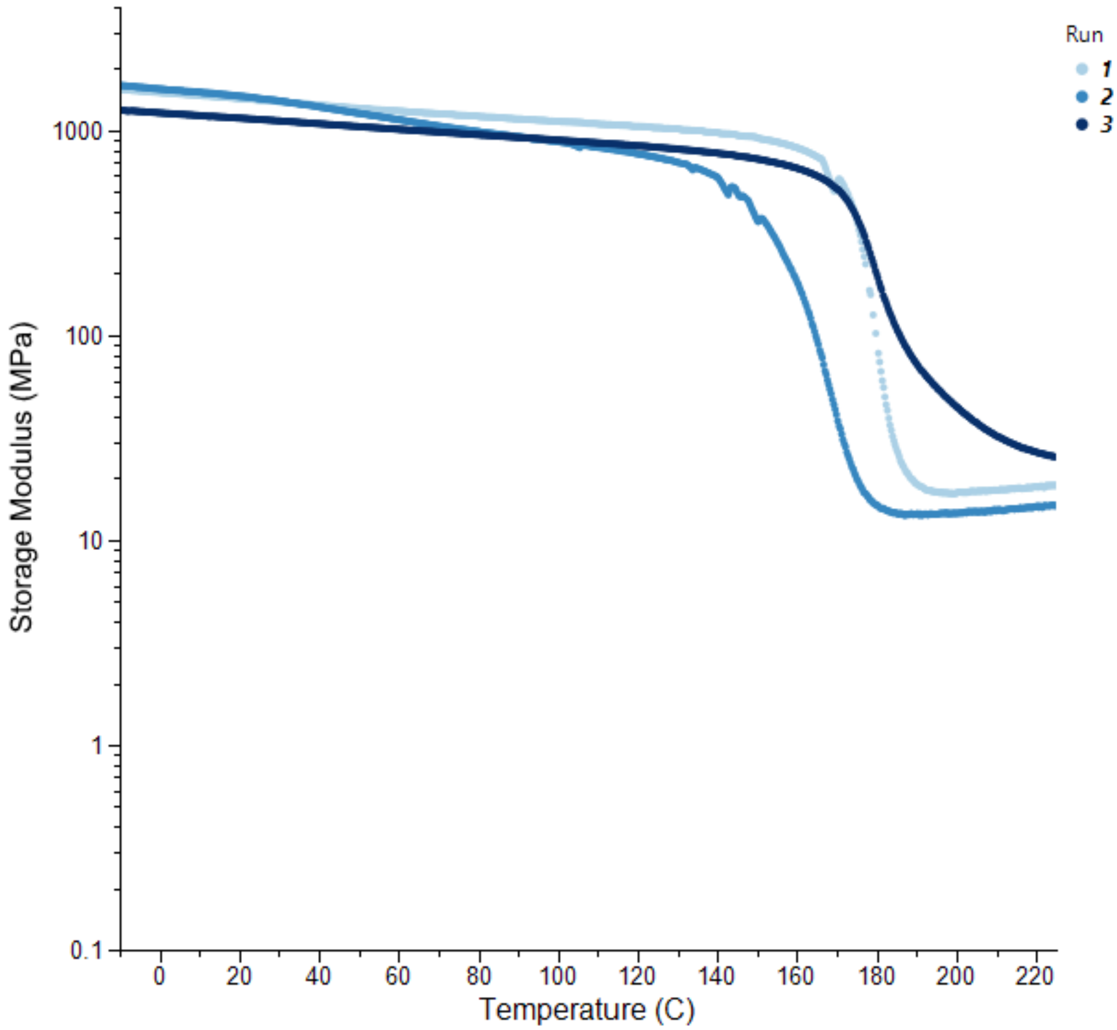

Figure S52. Storage modulus traces of pDCPD doped with 10 mol% **MeSi8**.[1]

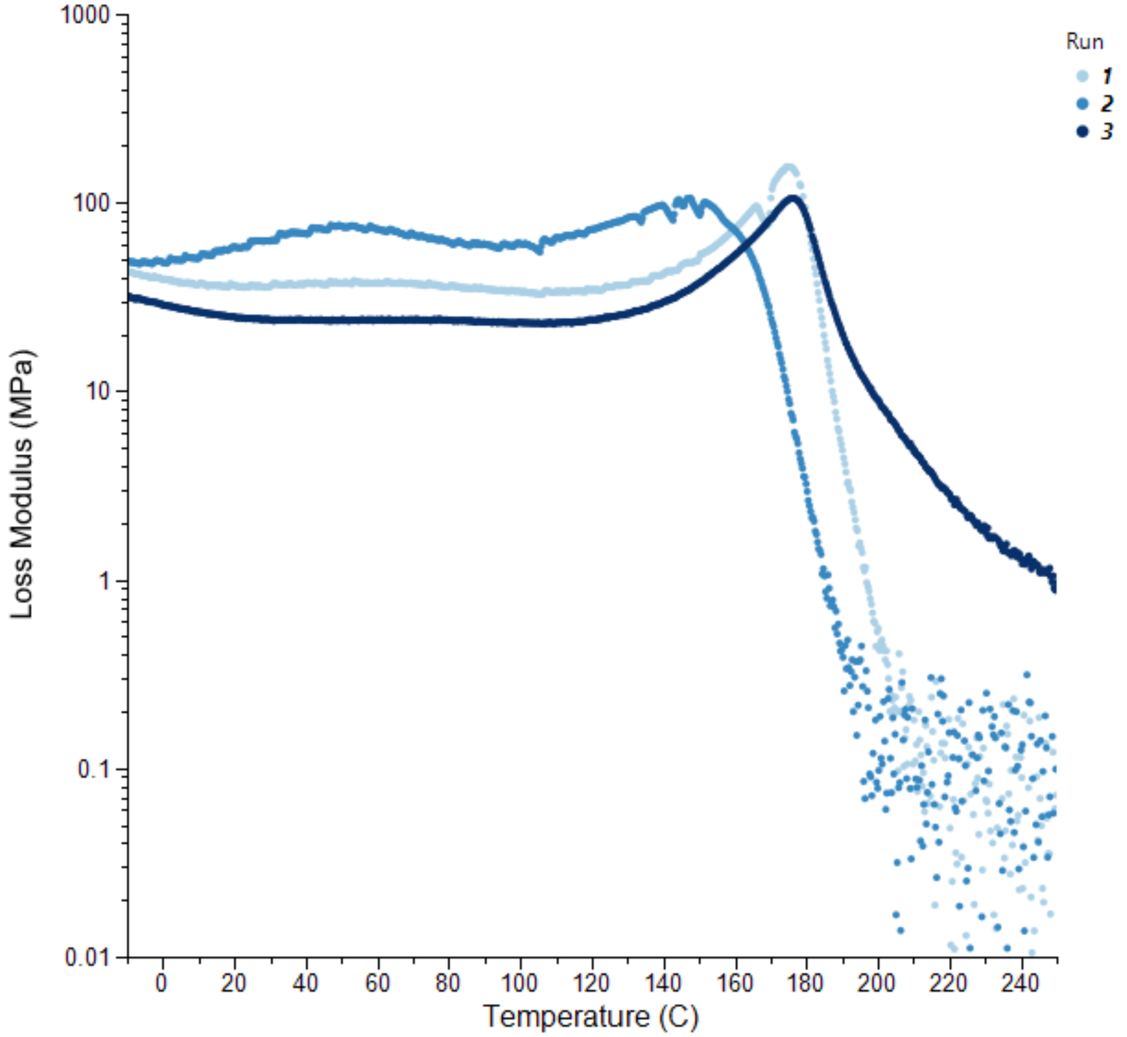

Figure S53. Loss modulus traces of pDCPD doped with 10 mol% **MeSi8**.

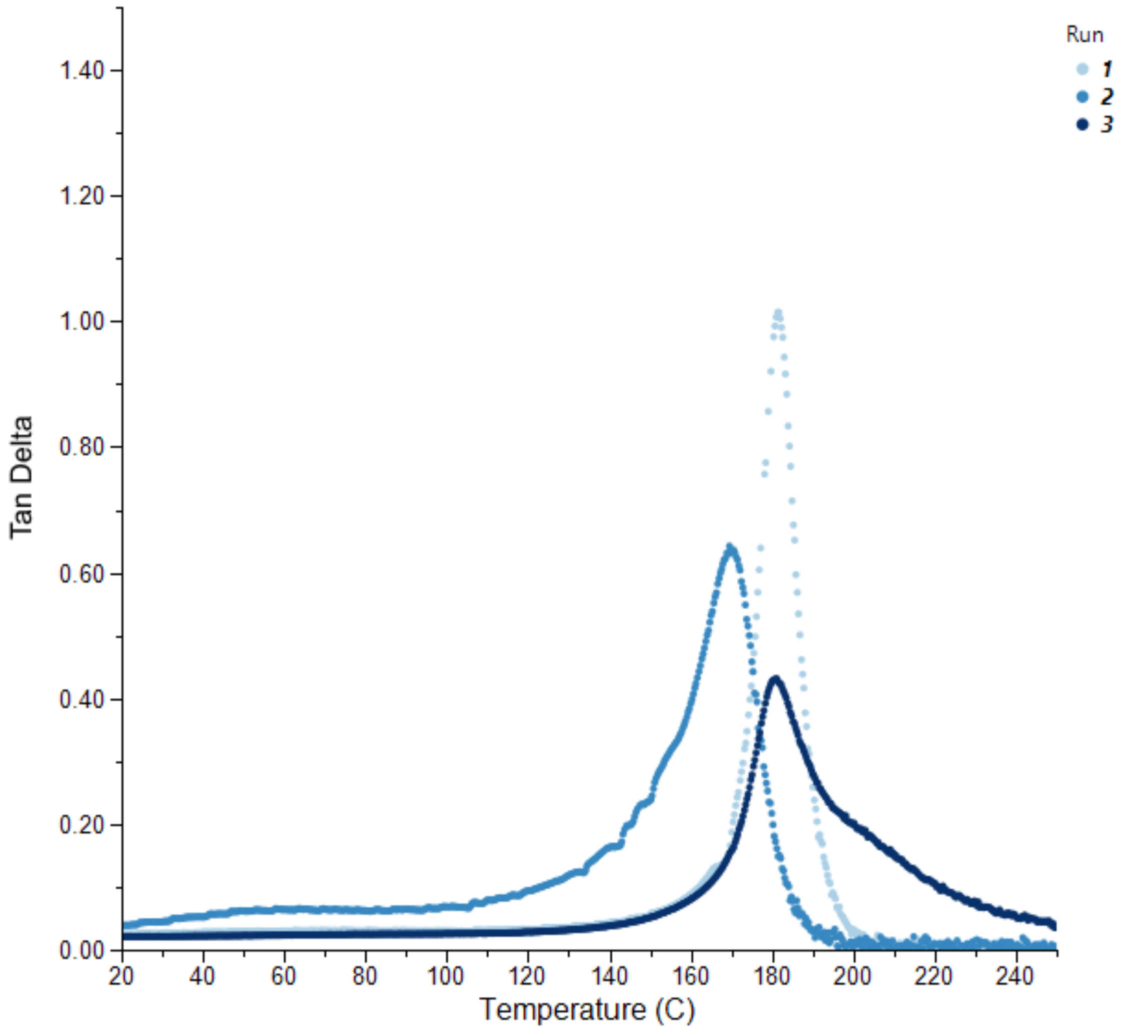

Figure S54. Tan delta traces of pDCPD doped with 10 mol% **MeSi8**.

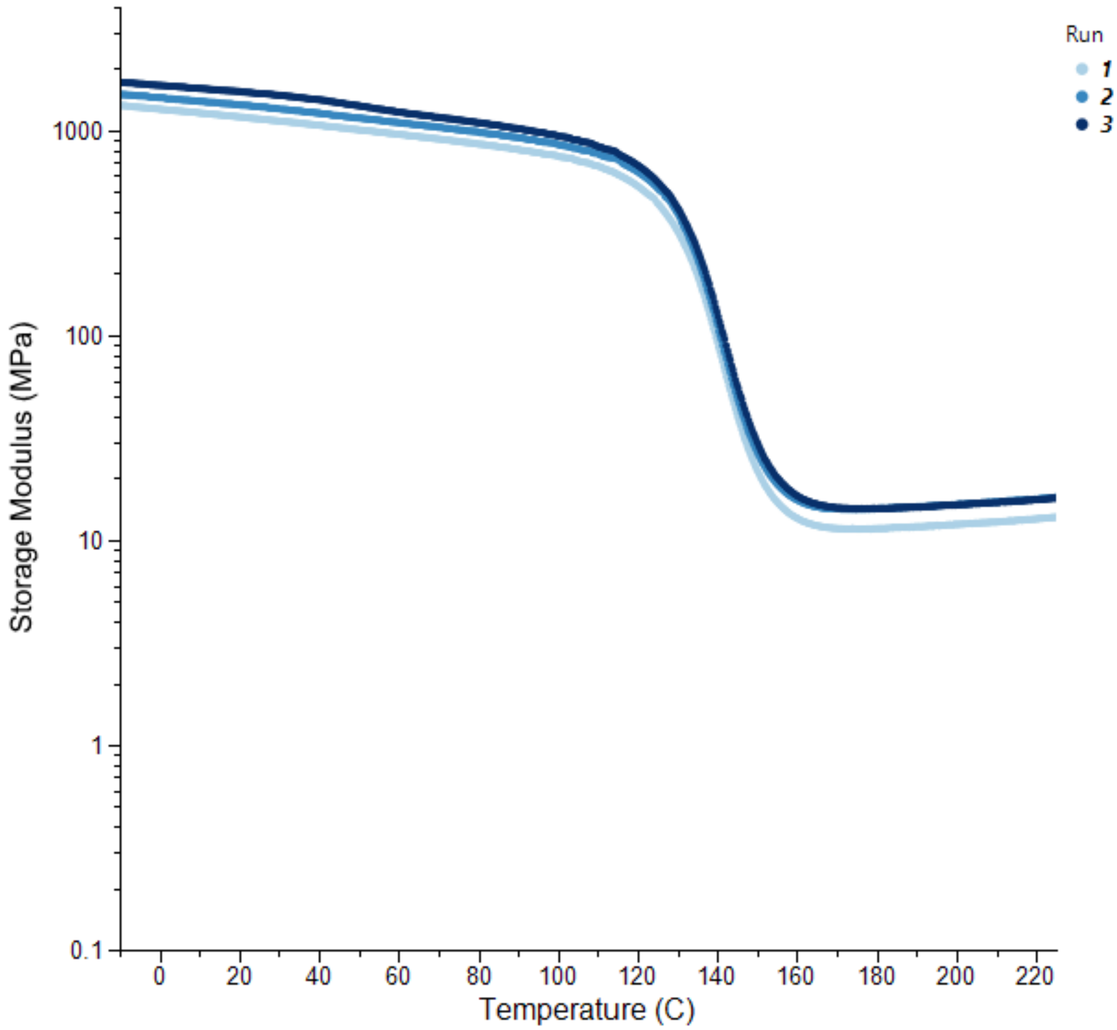

Figure S55. Storage modulus traces of pDCPD doped with 10 mol% **iPrSi8**, 10 mol% **DDMS**, FROMP cure.

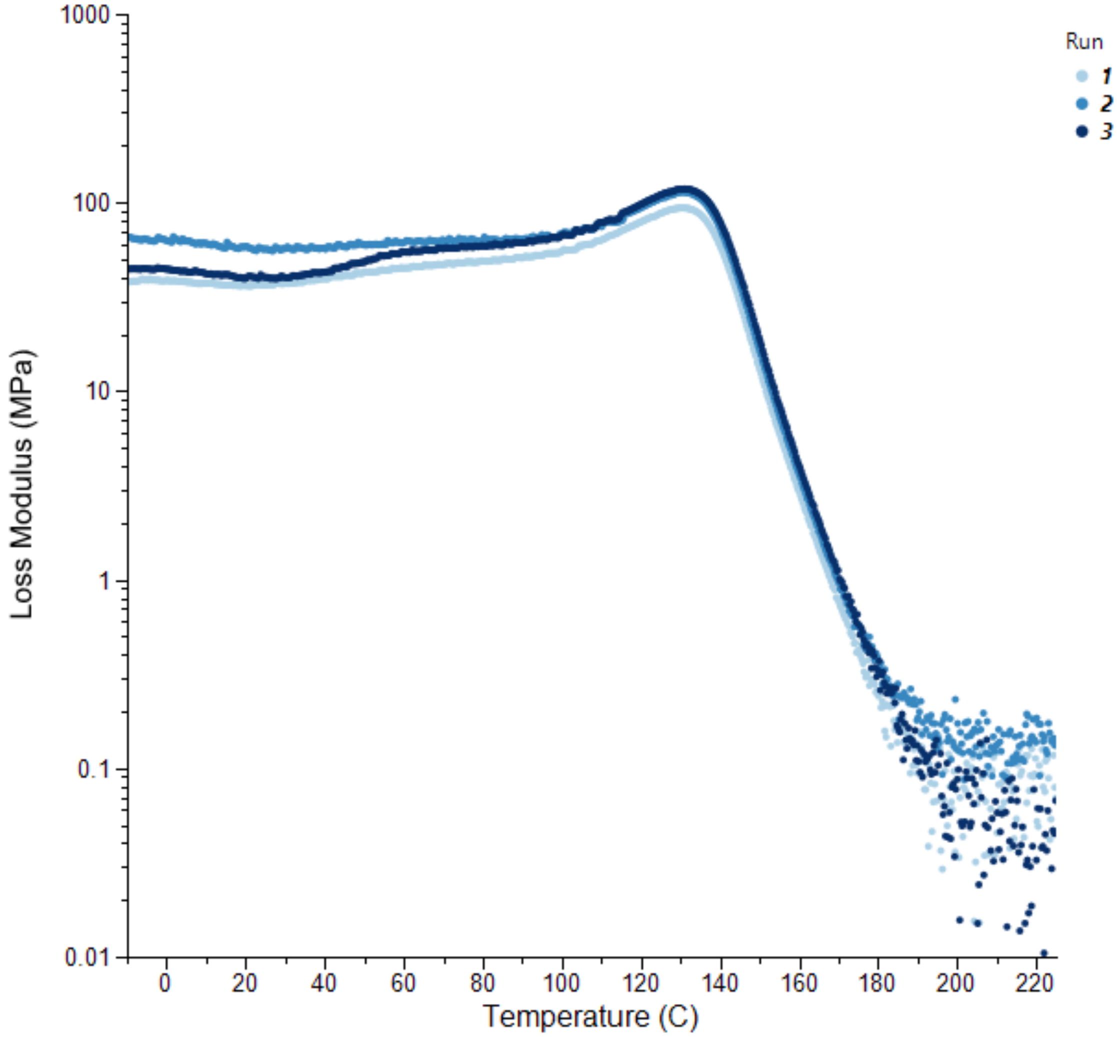

Figure S56. Loss modulus traces of pDCPD doped with 10 mol% **iPrSi8**, 10 mol% **DDMS**, FROMP cure**.**

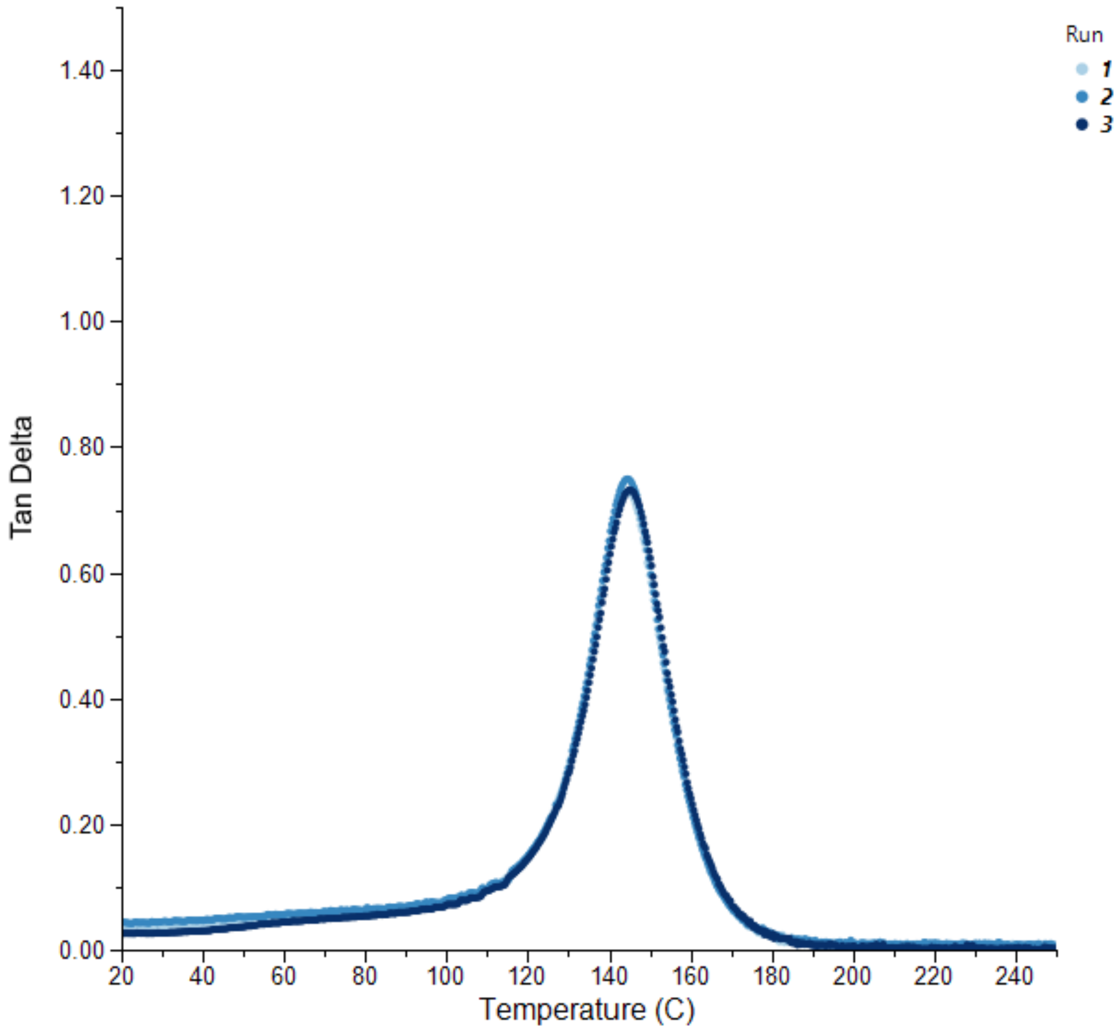

Figure S57. Tan delta traces of pDCPD doped with 10 mol% **iPrSi8**, 10 mol% **DDMS**, FROMP cure.

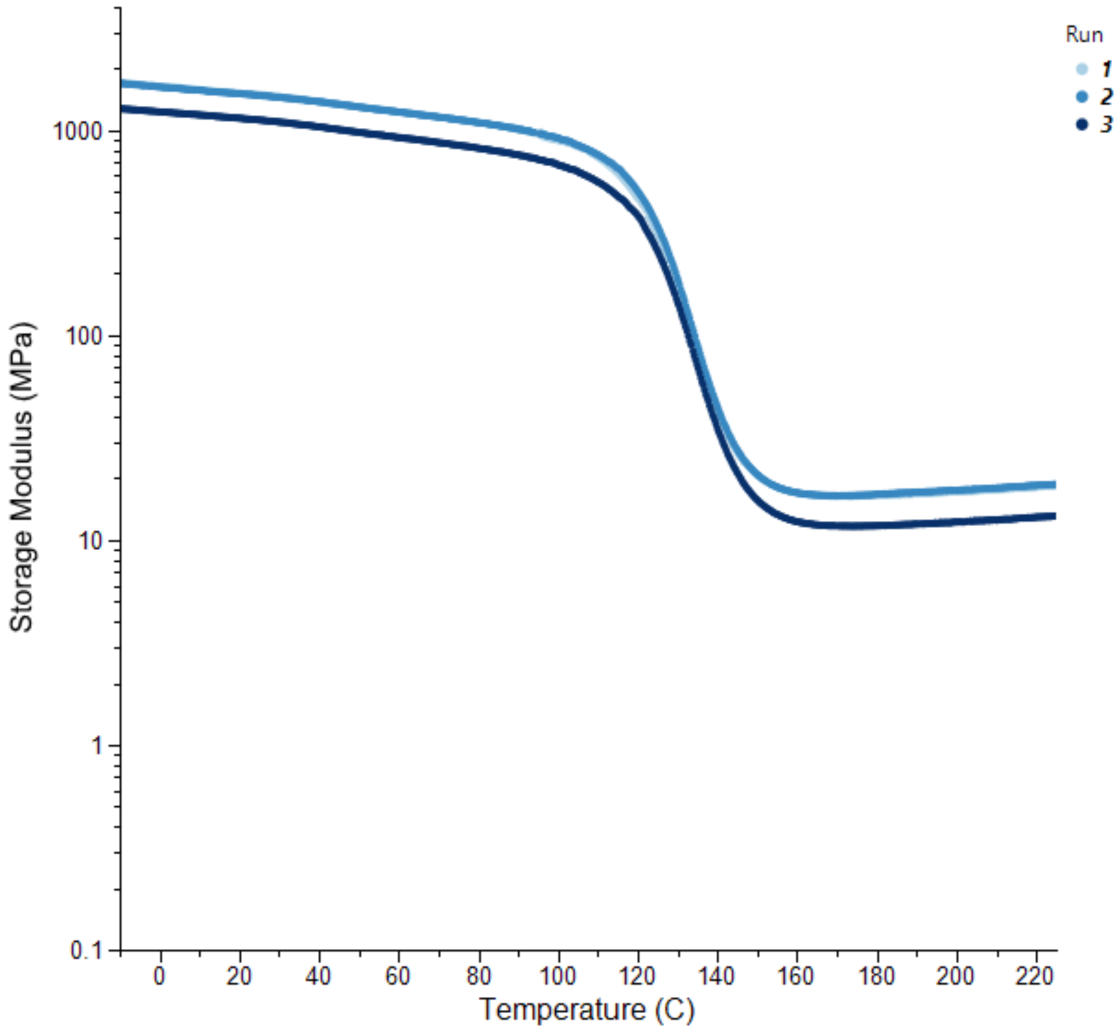

Figure S58. Storage modulus traces of pDCPD doped with 10 mol% **iPrSi8**, 10 mol% **SCE**.

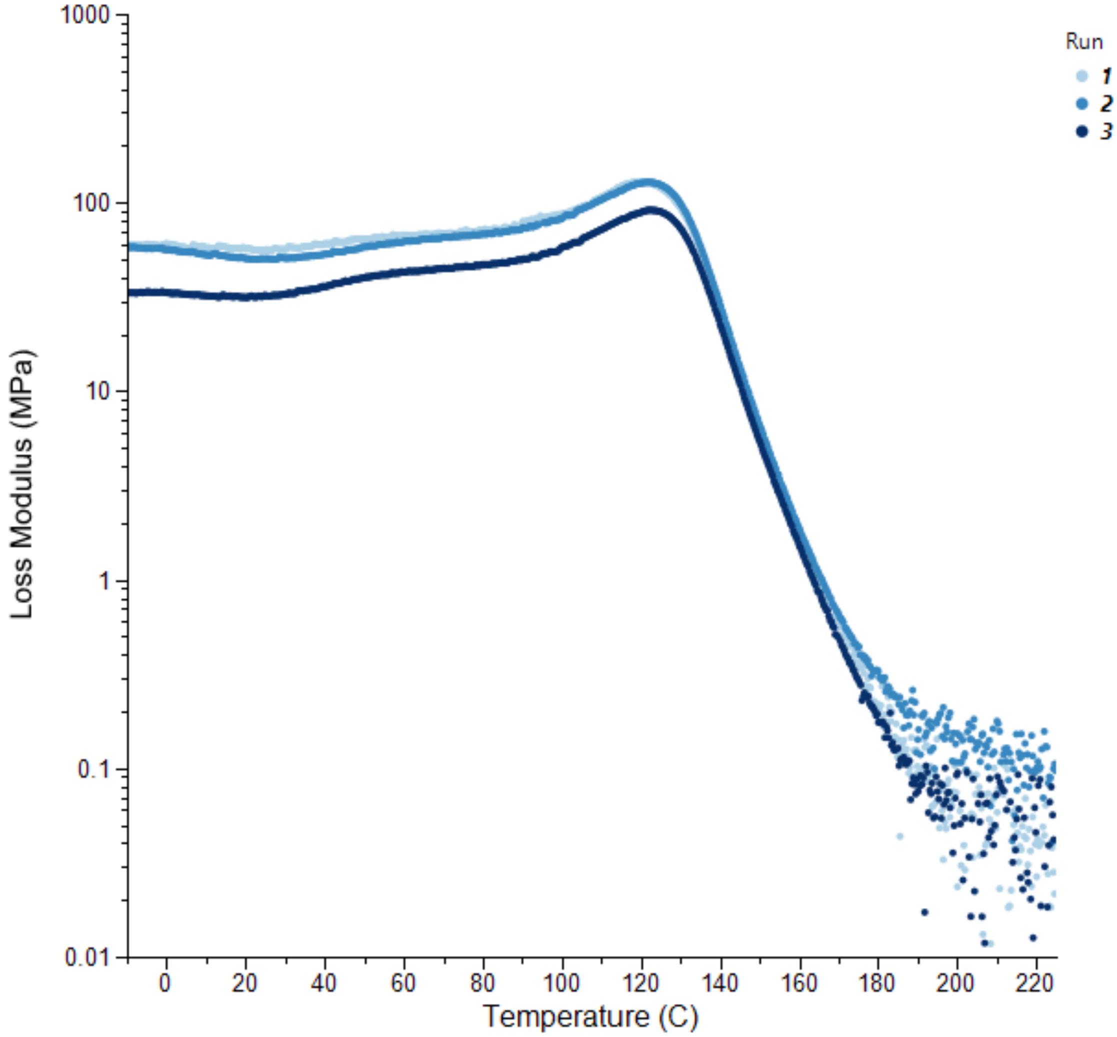

Figure S59. Loss modulus traces of pDCPD doped with 10 mol% **iPrSi8**, 10 mol% **SCE.**

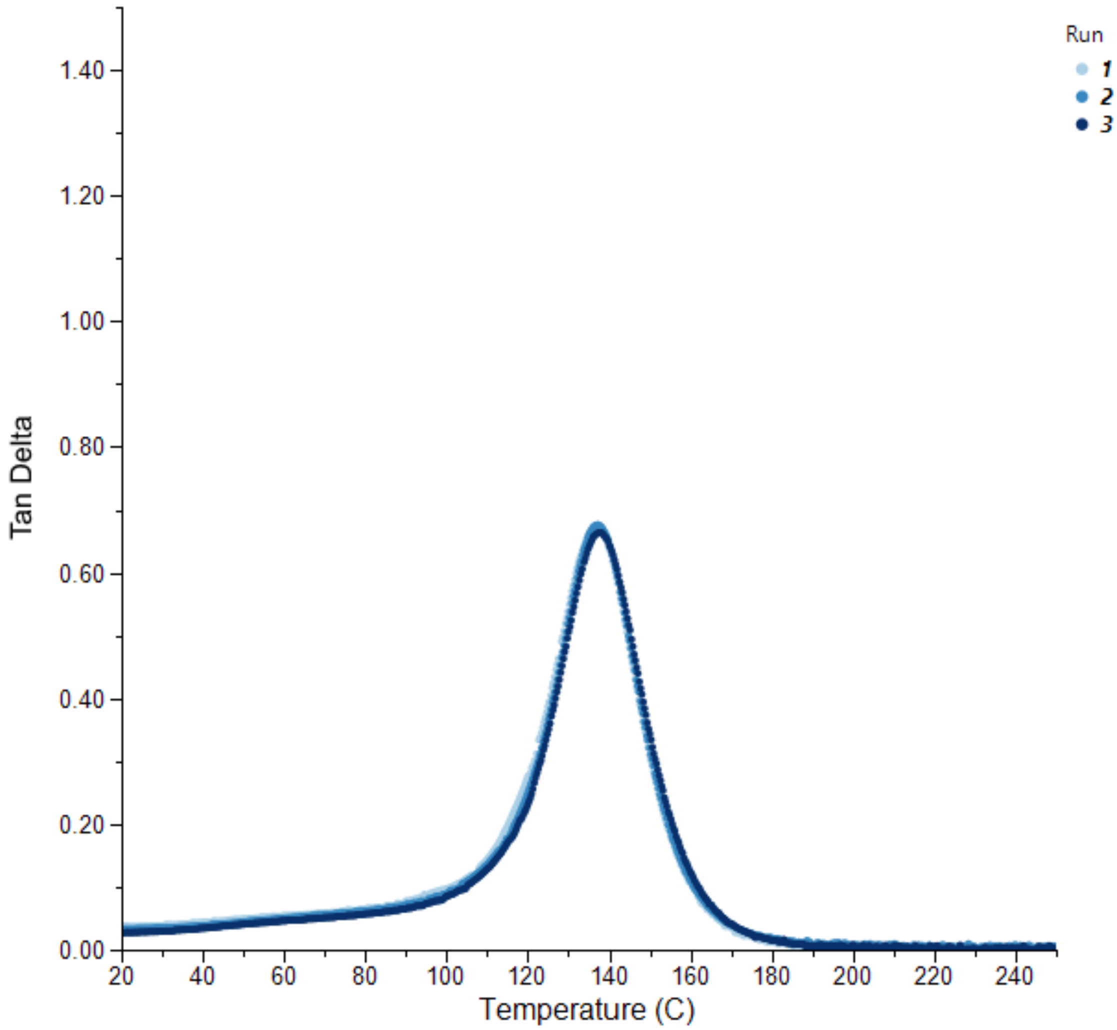

Figure S60. Tan delta traces of pDCPD doped with 10 mol% **iPrSi8**, 10 mol% **SCE**.

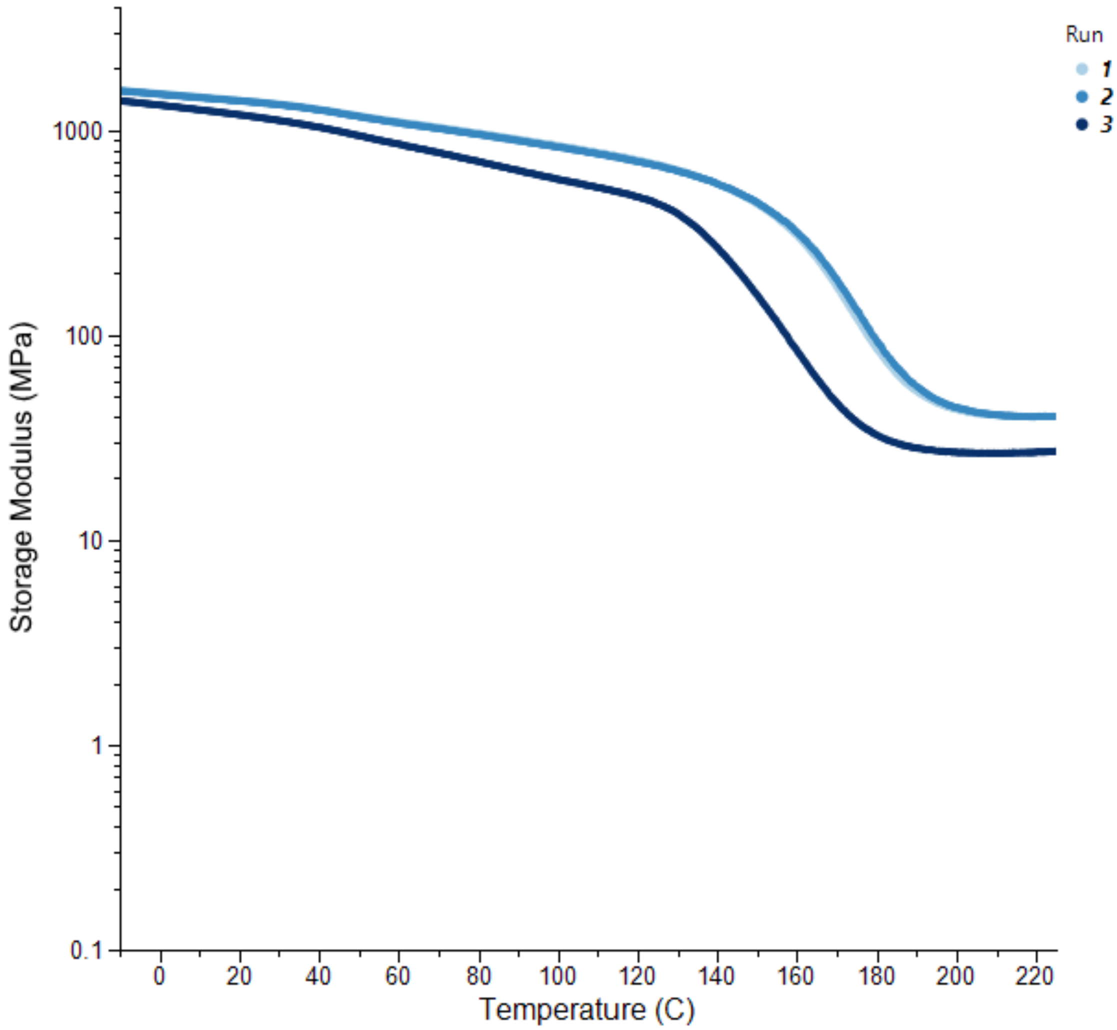

Figure S61. Storage modulus traces of pDCPD doped with 10 mol% **iPrSi8**, 20 mol% **SCE**.

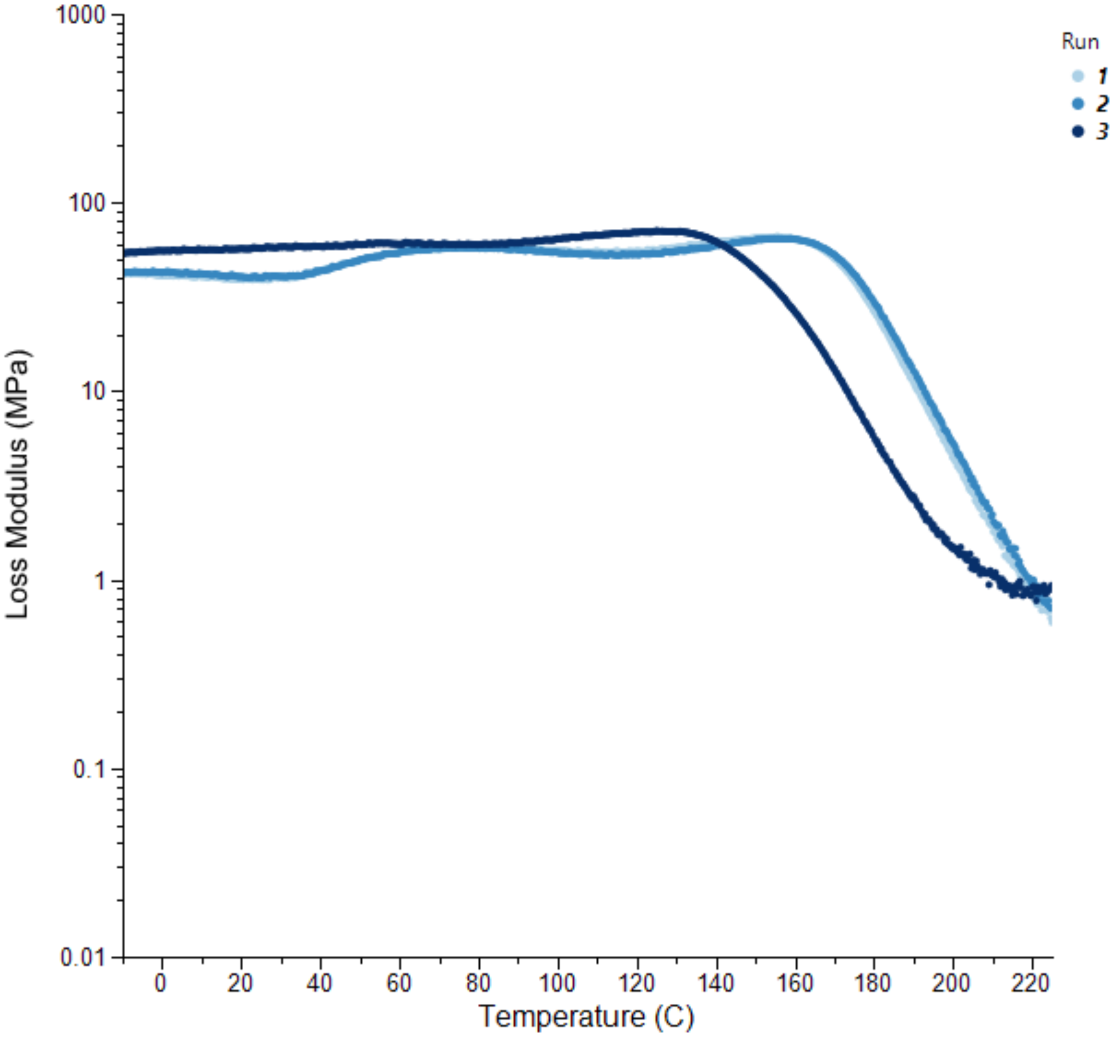

Figure S62. Loss modulus traces of pDCPD doped with 10 mol% **iPrSi8**, 20 mol% **SCE**.

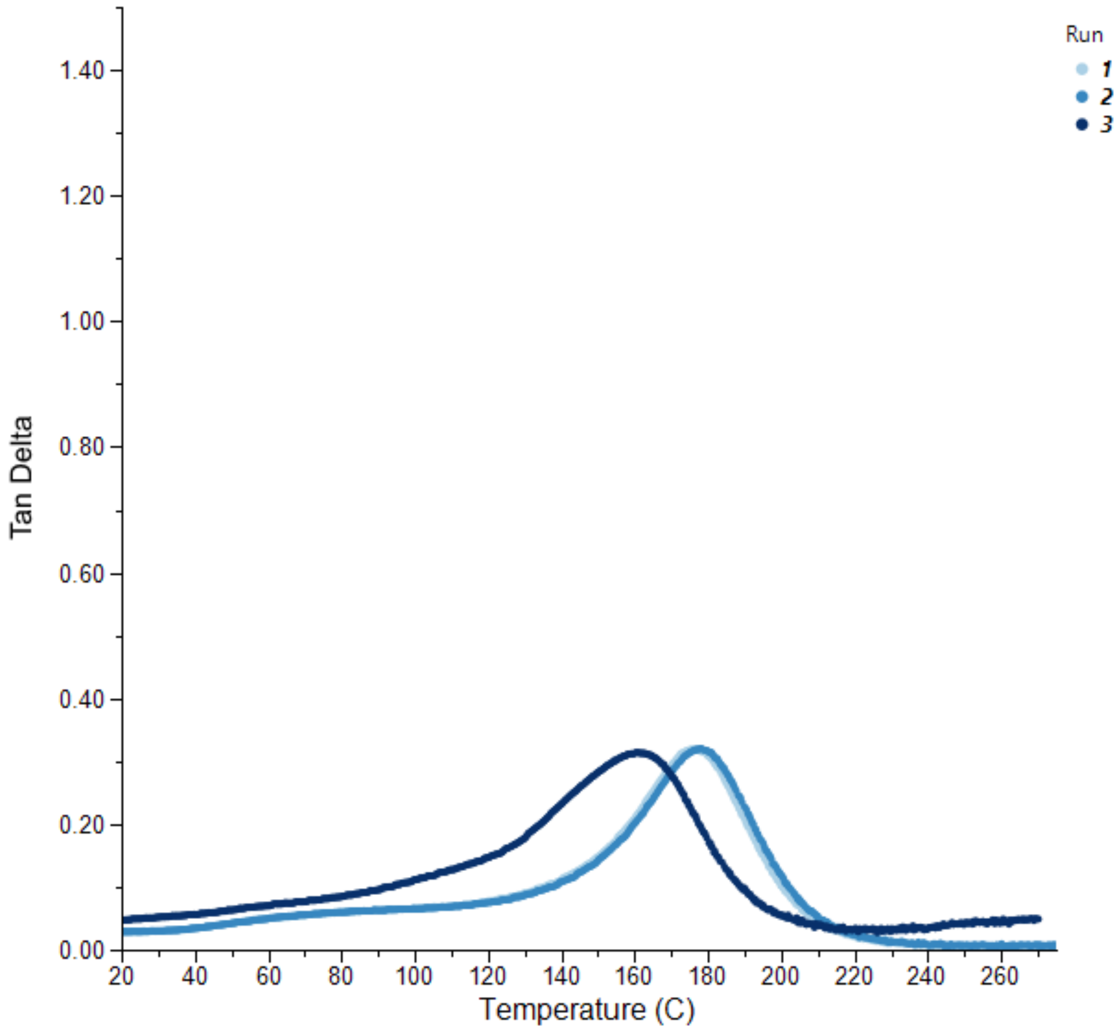

Figure S63. Tan delta traces of pDCPD doped with 10 mol% **iPrSi8**, 20 mol% **SCE**.

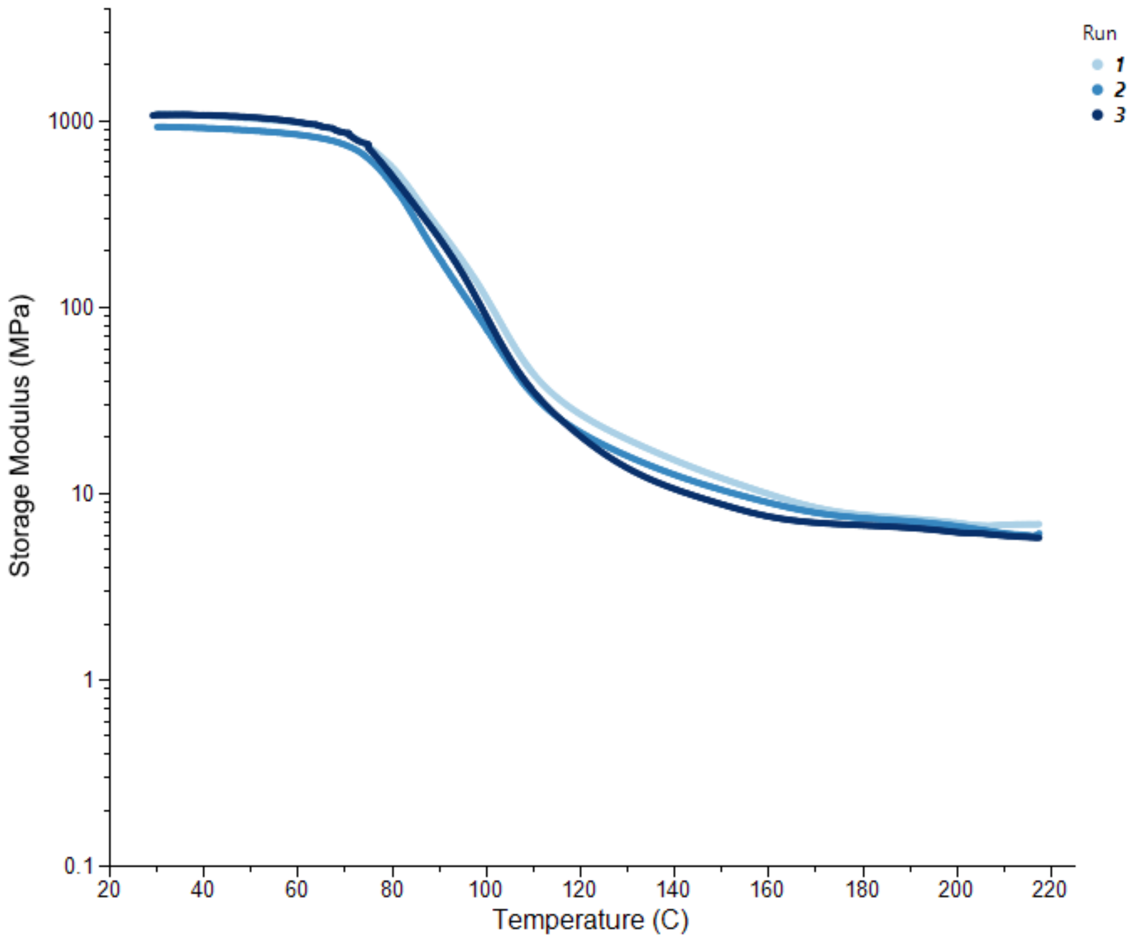

Figure S64. Storage Modulus Traces of pDCPD doped with 10% v/v **iPrAc7**.[7]

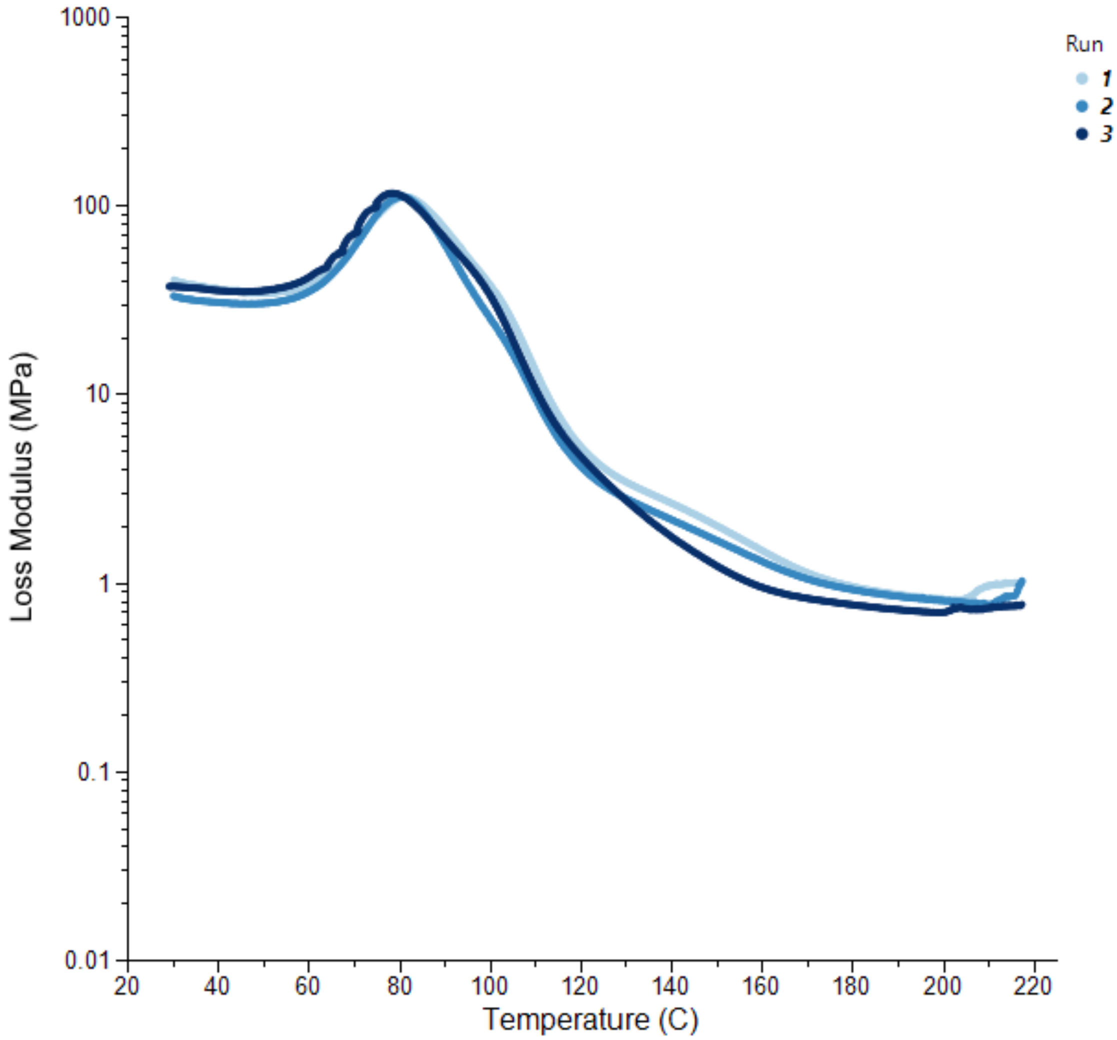

Figure S65. Loss modulus traces of pDCPD doped with 10% v/v **iPrAc7.**

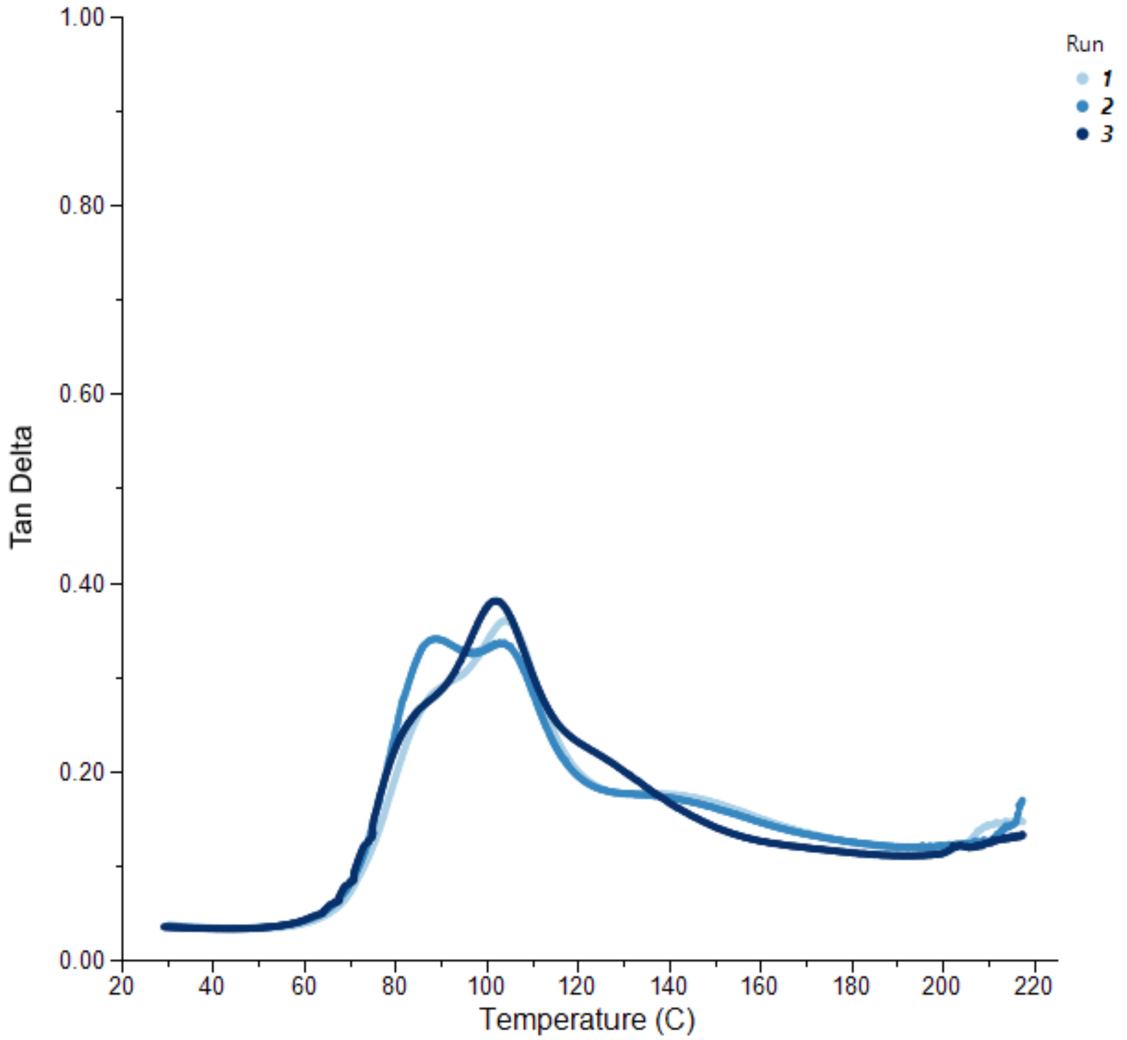

Figure S66. Tan delta traces of pDCPD doped with 10% v/v **iPrAc7.**

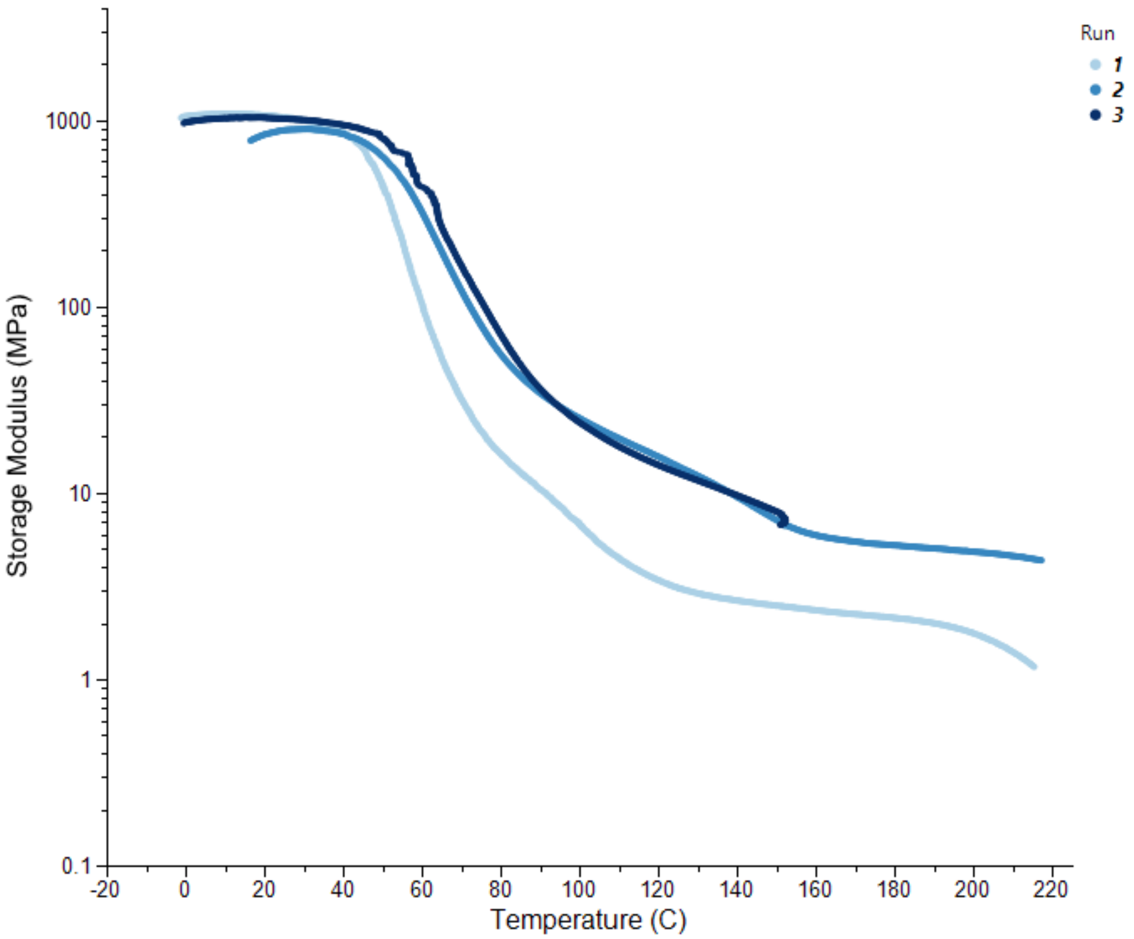

Figure S67. Storage modulus traces of pDCPD doped with 20% v/v **iPrAc7.**

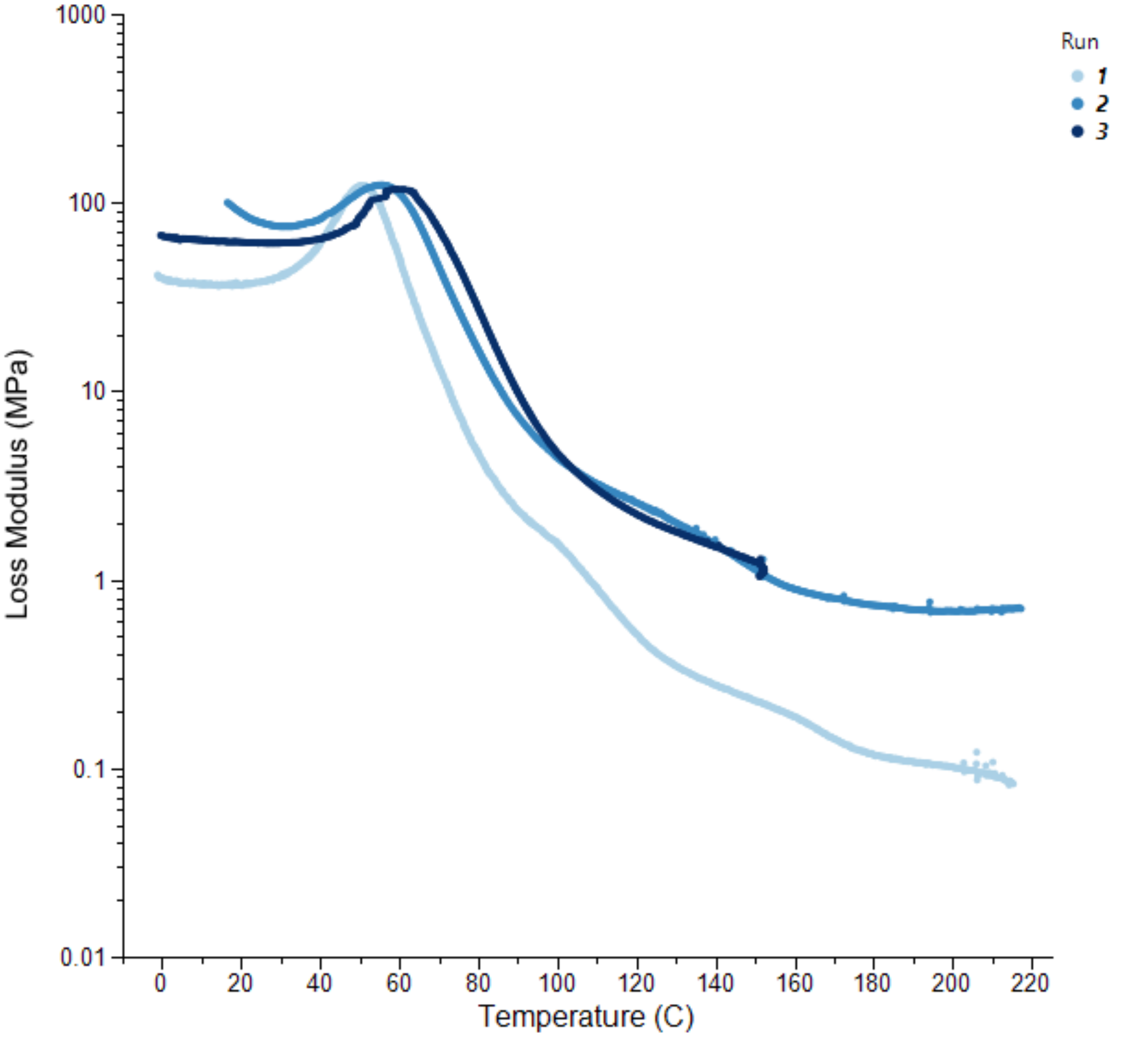

Figure S68. Loss modulus traces of pDCPD doped with 20% v/v **iPrAc7**.

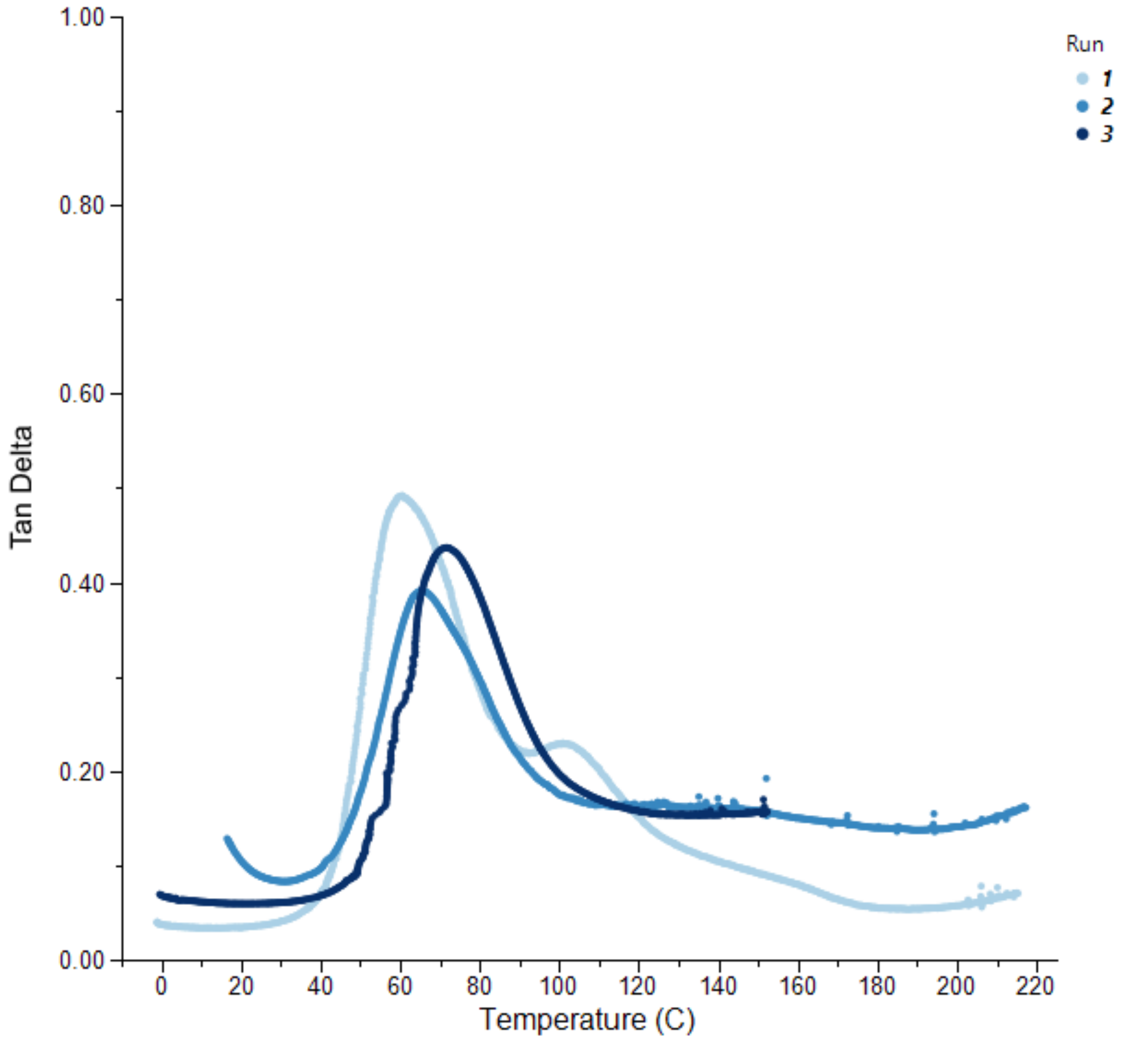

Figure S69. Tan delta traces of pDCPD doped with 20% v/v **iPrAc7.**

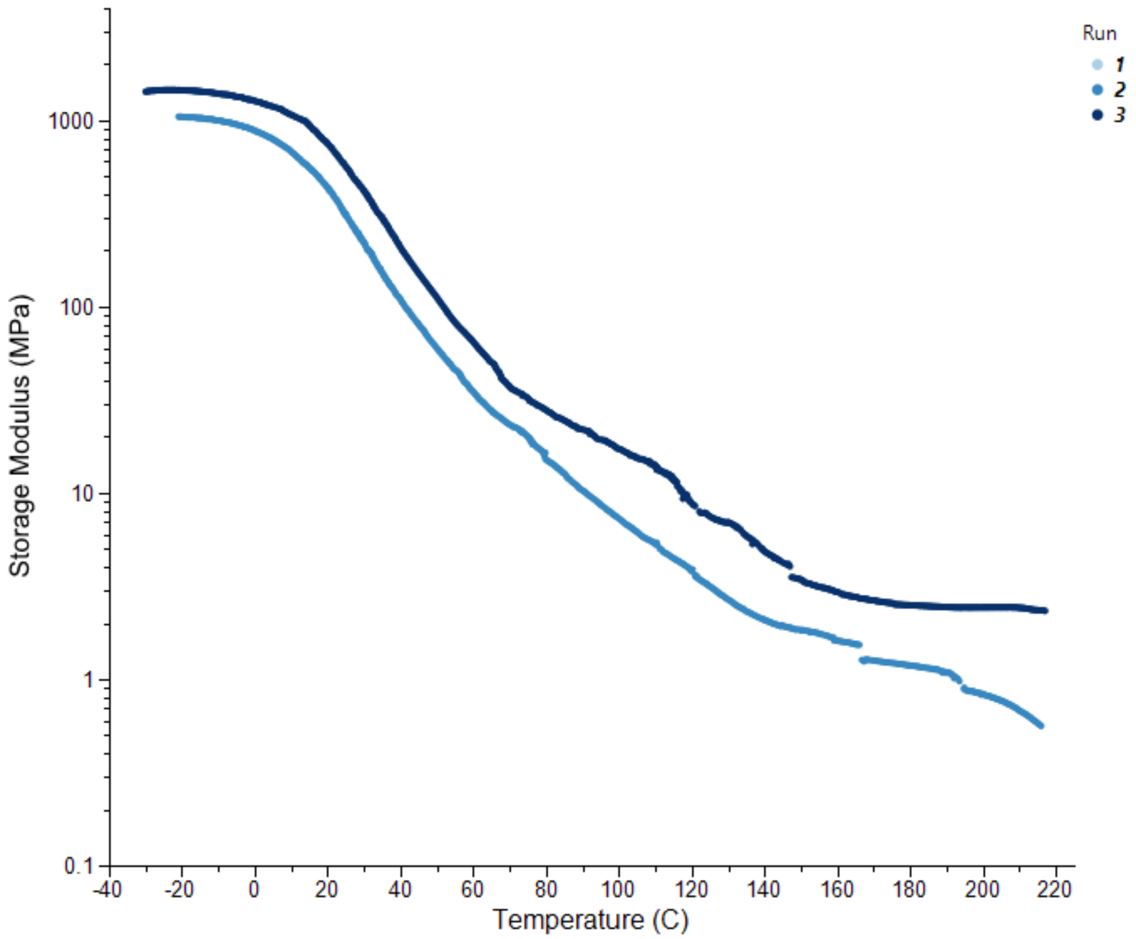

Figure S70. Storage modulus traces of pDCPD doped with 33% v/v **iPrAc7.**

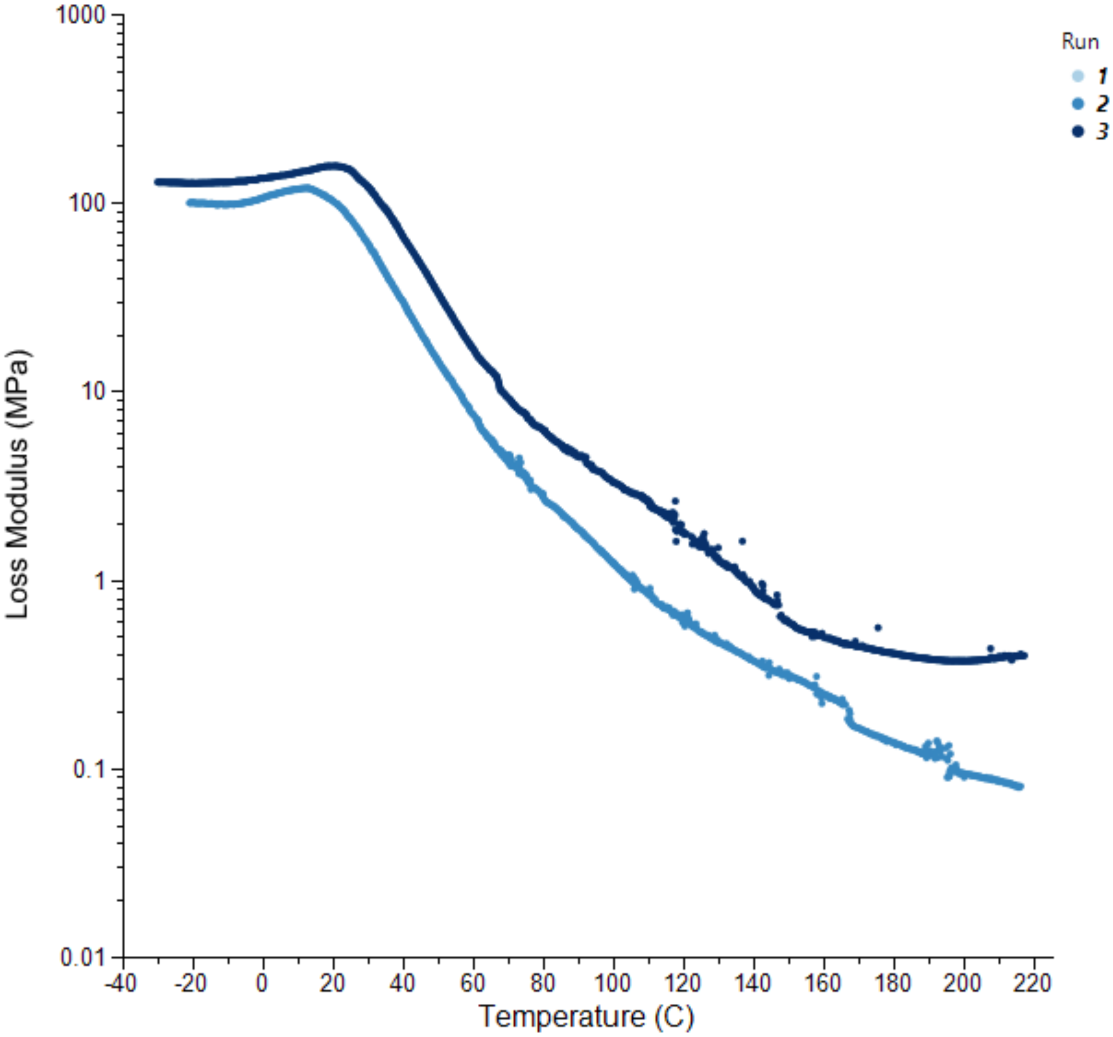

Figure S71. Loss modulus traces of pDCPD doped with 33% v/v **iPrAc7.**

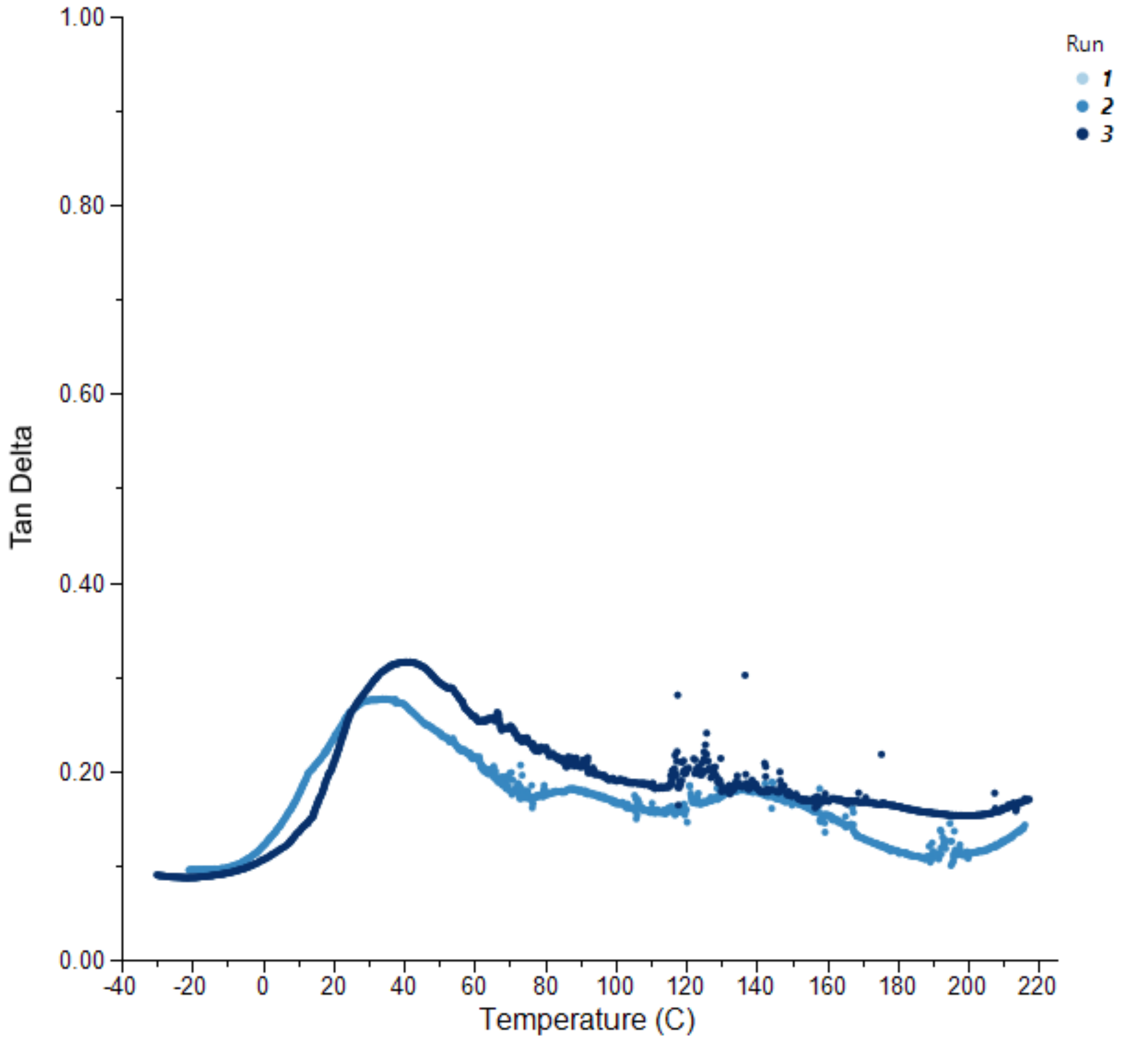

Figure S72. Tan delta traces of pDCPD doped with 33% v/v **iPrAc7.**

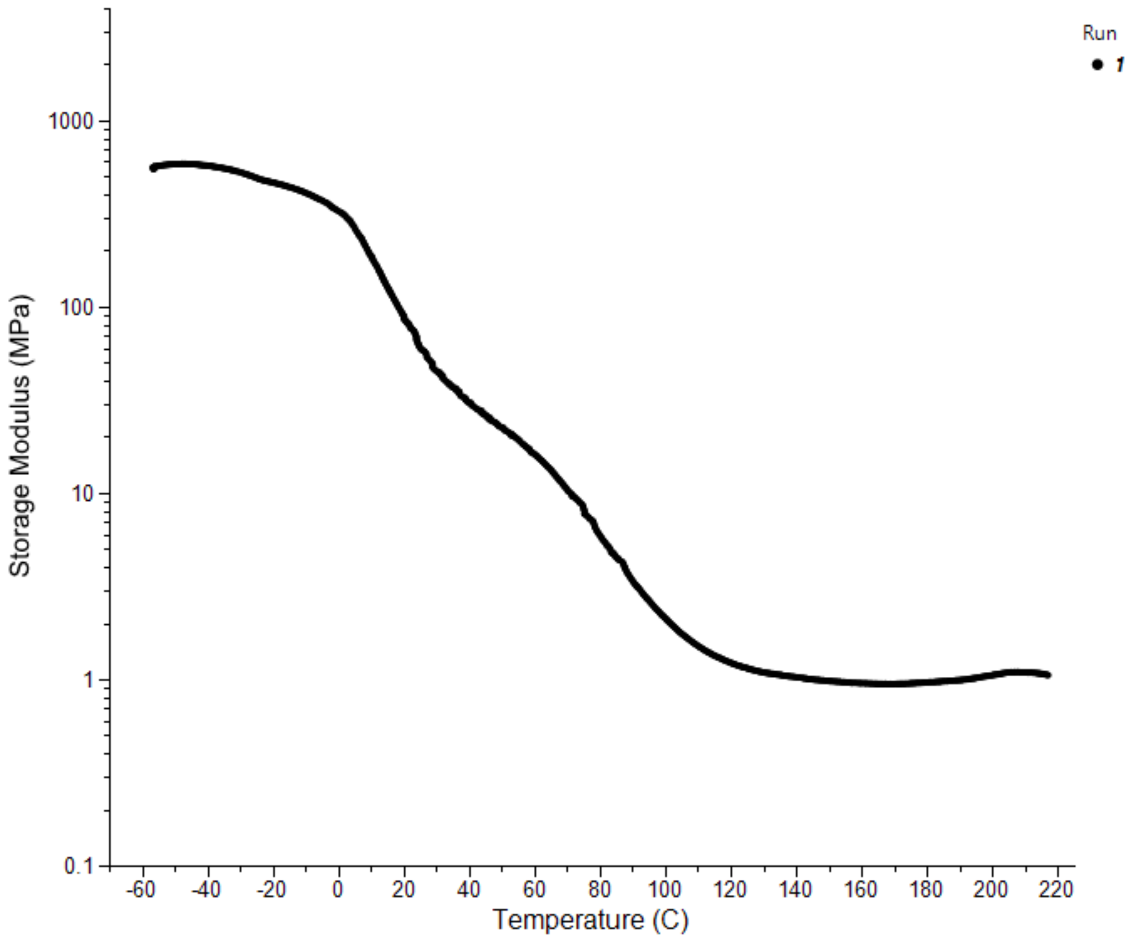

Figure S73. Storage modulus trace of pDCPD doped with 50% v/v **iPrAc7.**

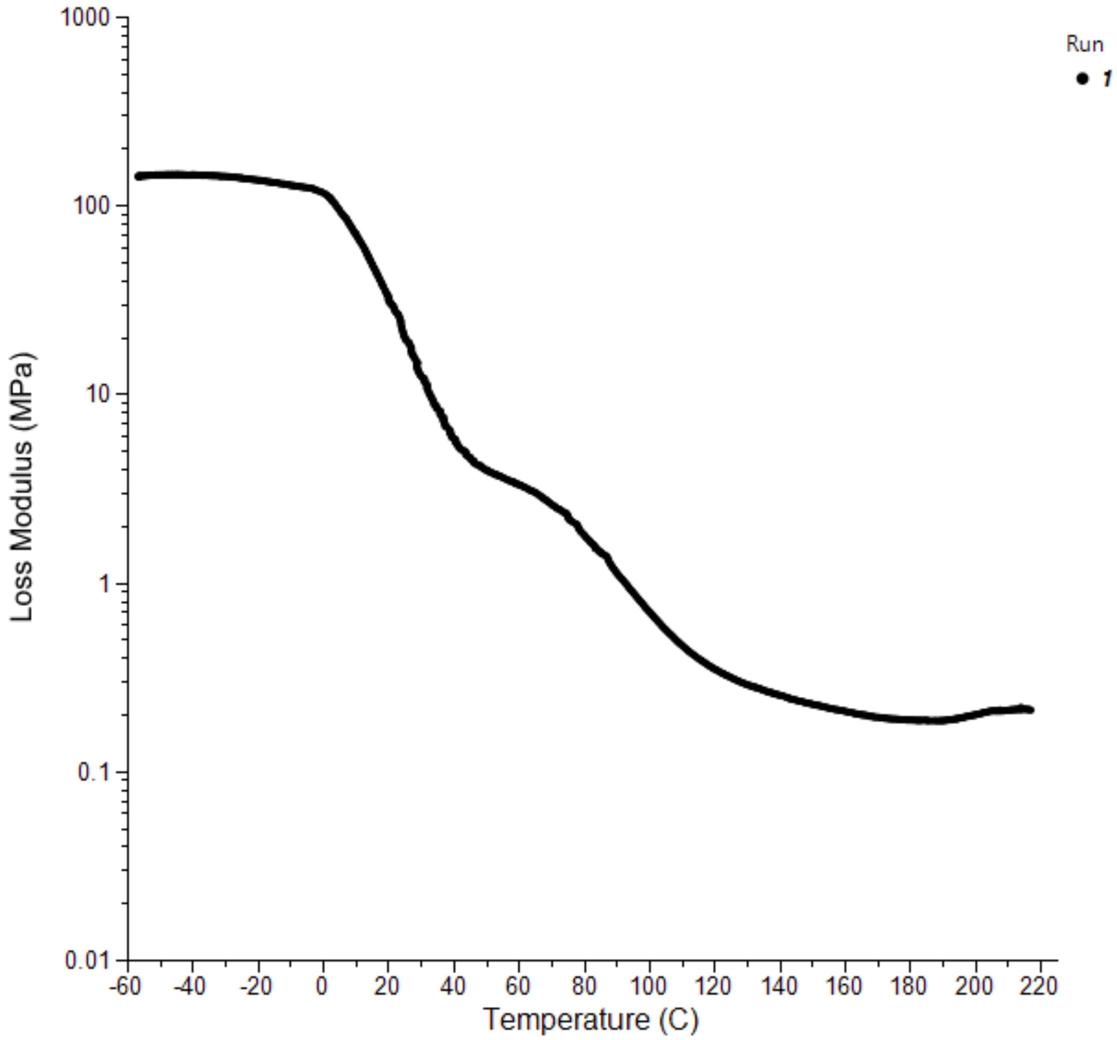

Figure S74. Loss modulus trace of pDCPD doped with 50% v/v **iPrAc7.**

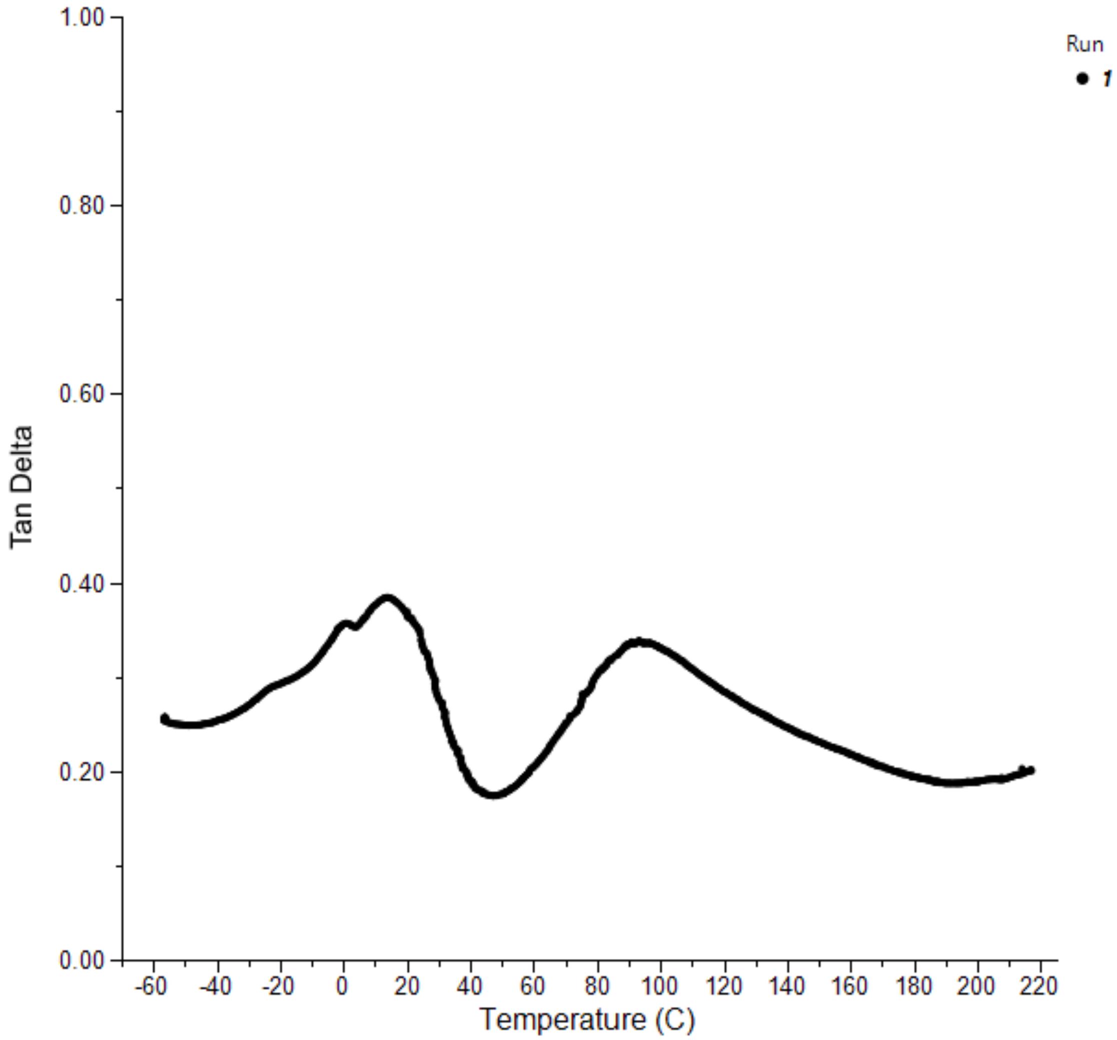

Figure S75. Tan delta trace of pDCPD doped with 50% v/v **iPrAc7.**

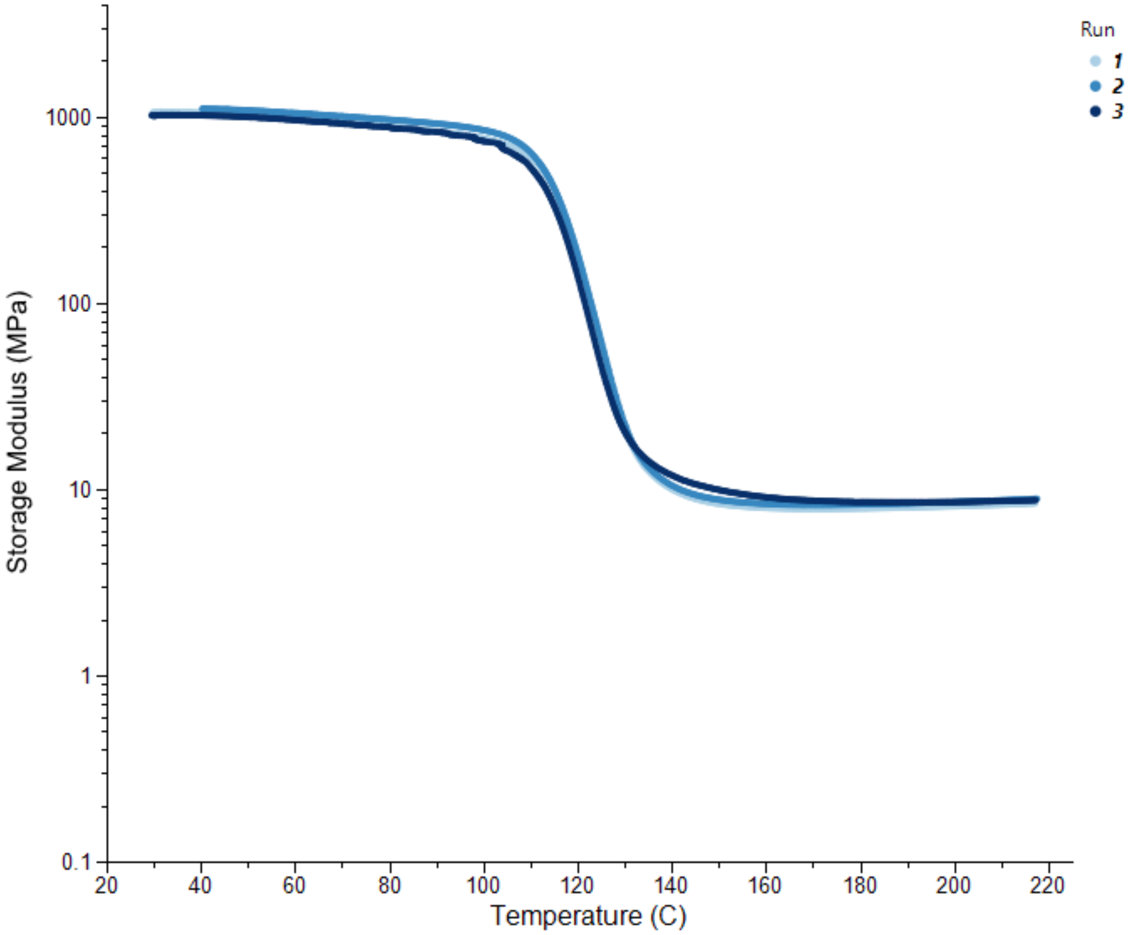

Figure S76. Storage modulus traces of pDCPD doped with 10% v/v **iPrSi7.**[1]

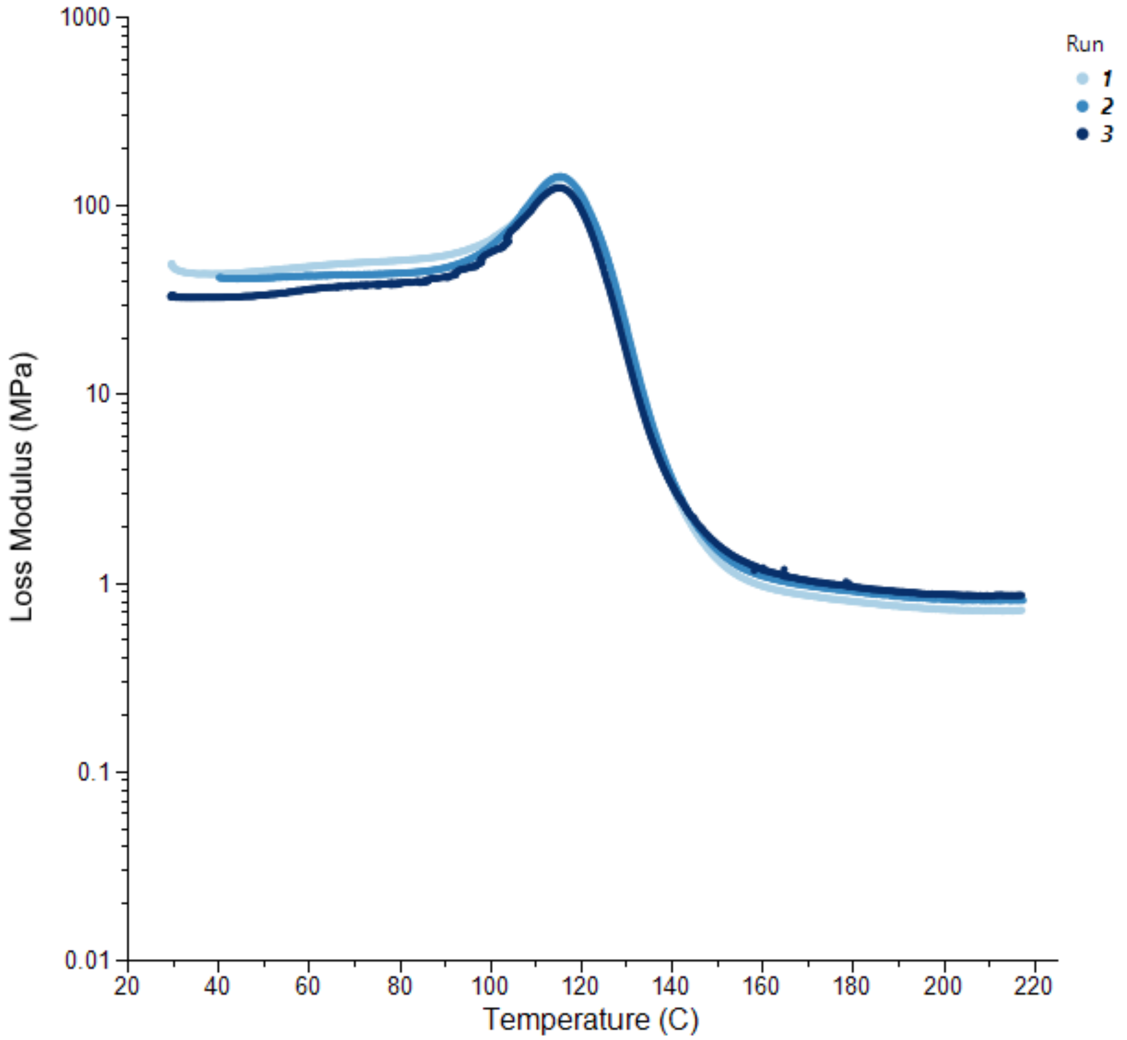

Figure S77. Loss modulus traces of pDCPD doped with 10% v/v **iPrSi7.**

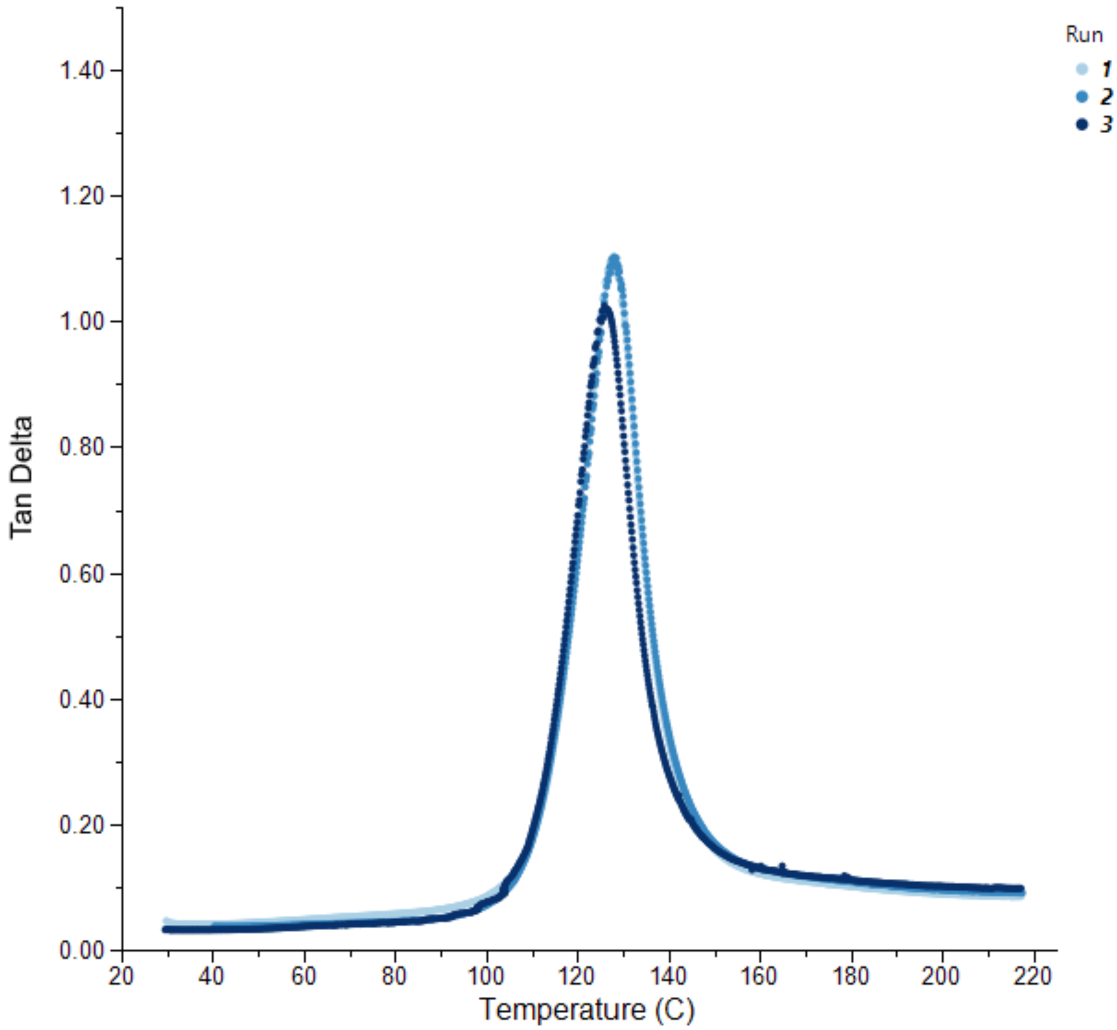

Figure S78. Tan delta traces of pDCPD doped with 10% v/v **iPrSi7.**

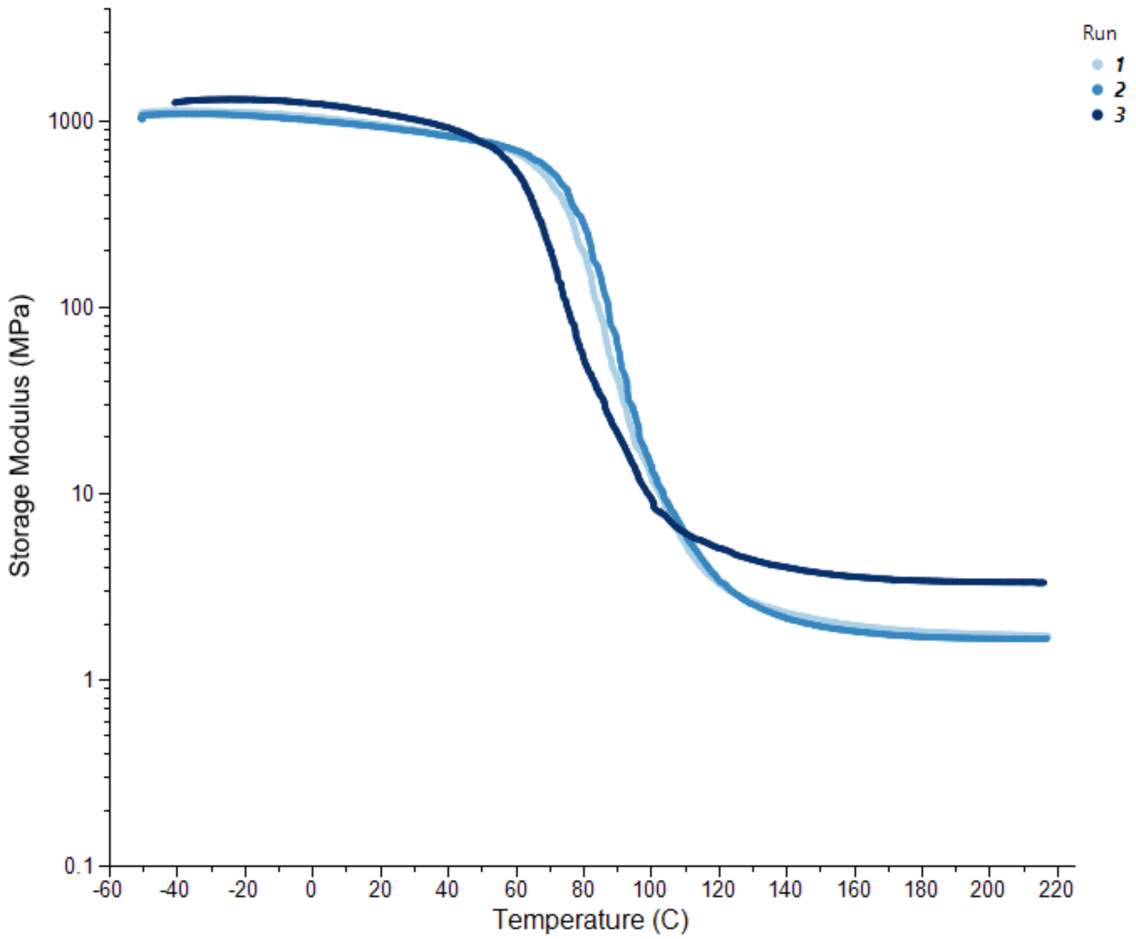

Figure S79. Storage modulus traces of pDCPD doped with 20% v/v **iPrSi7.**

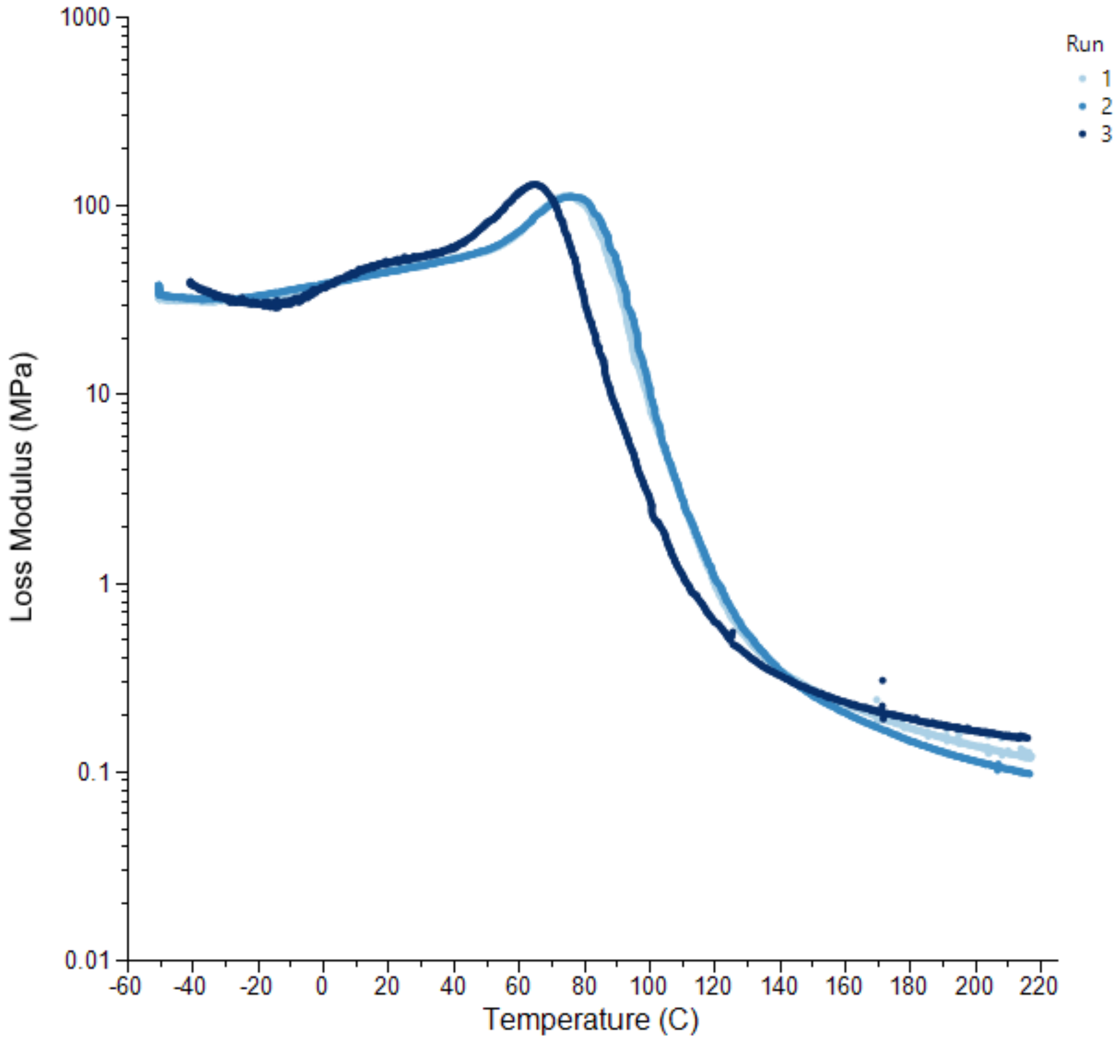

Figure S80. Loss modulus traces of pDCPD doped with 20% v/v **iPrSi7.**

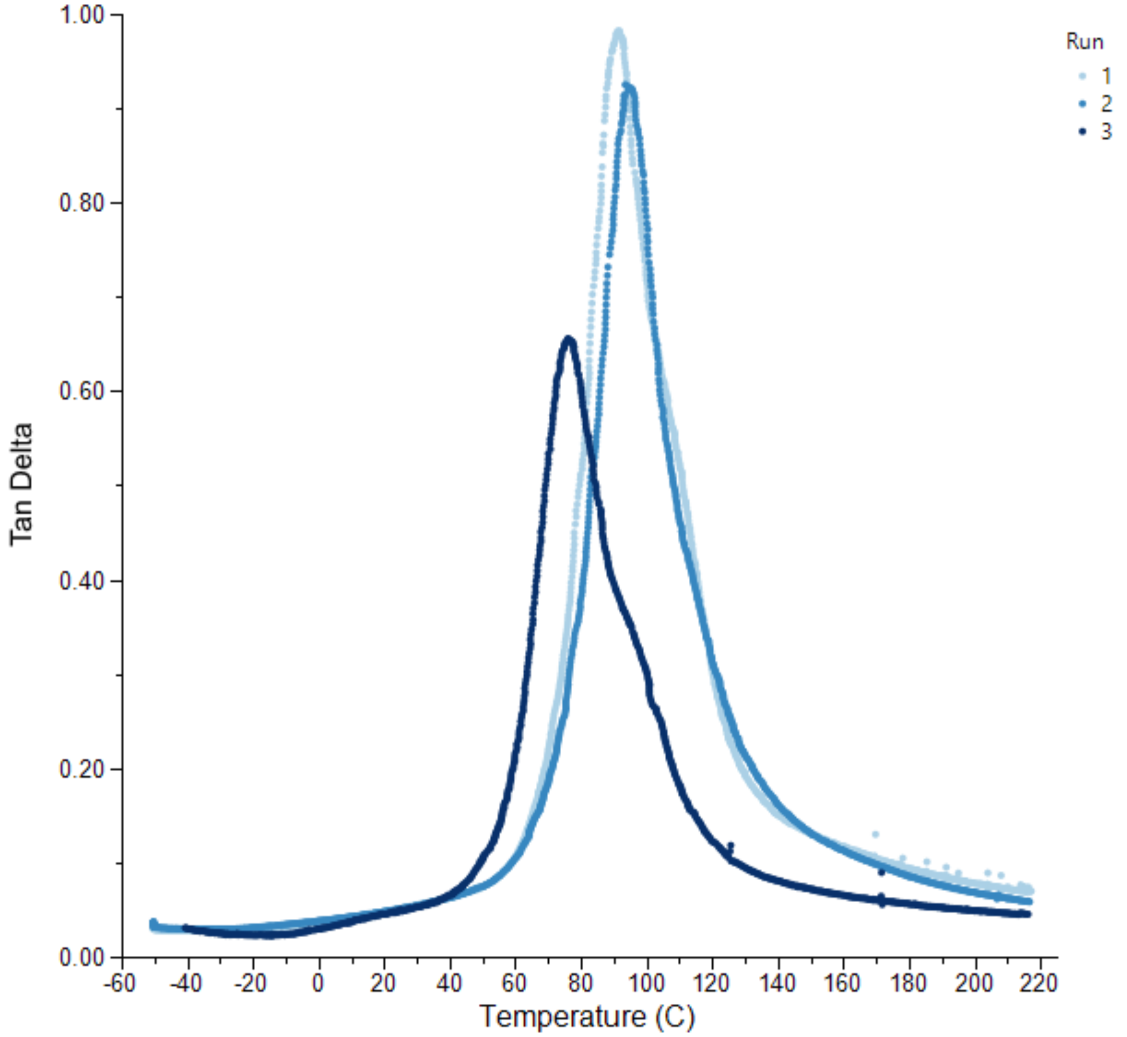

Figure S81. Tan delta traces of pDCPD doped with 20% v/v **iPrSi7.**

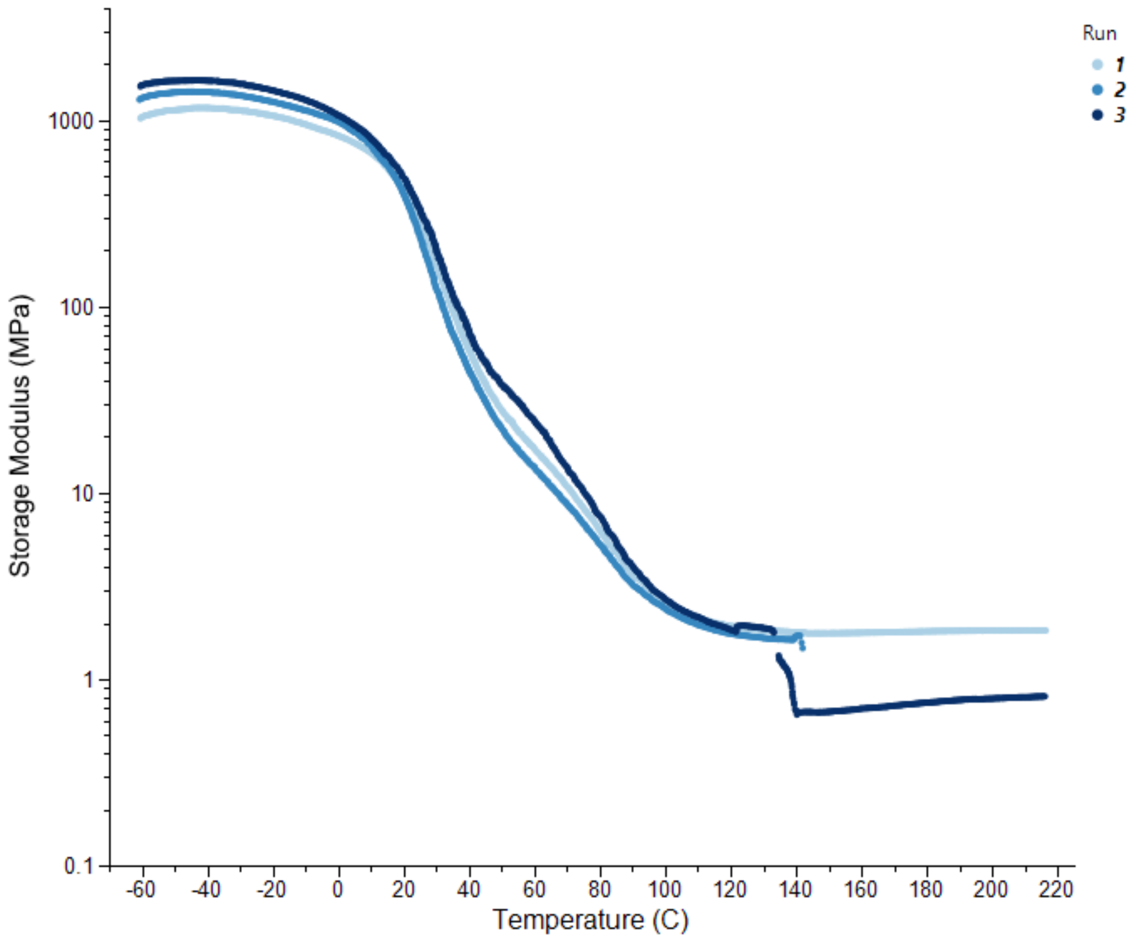

Figure S82. Storage modulus traces of pDCPD doped with 33% v/v **iPrSi7.**

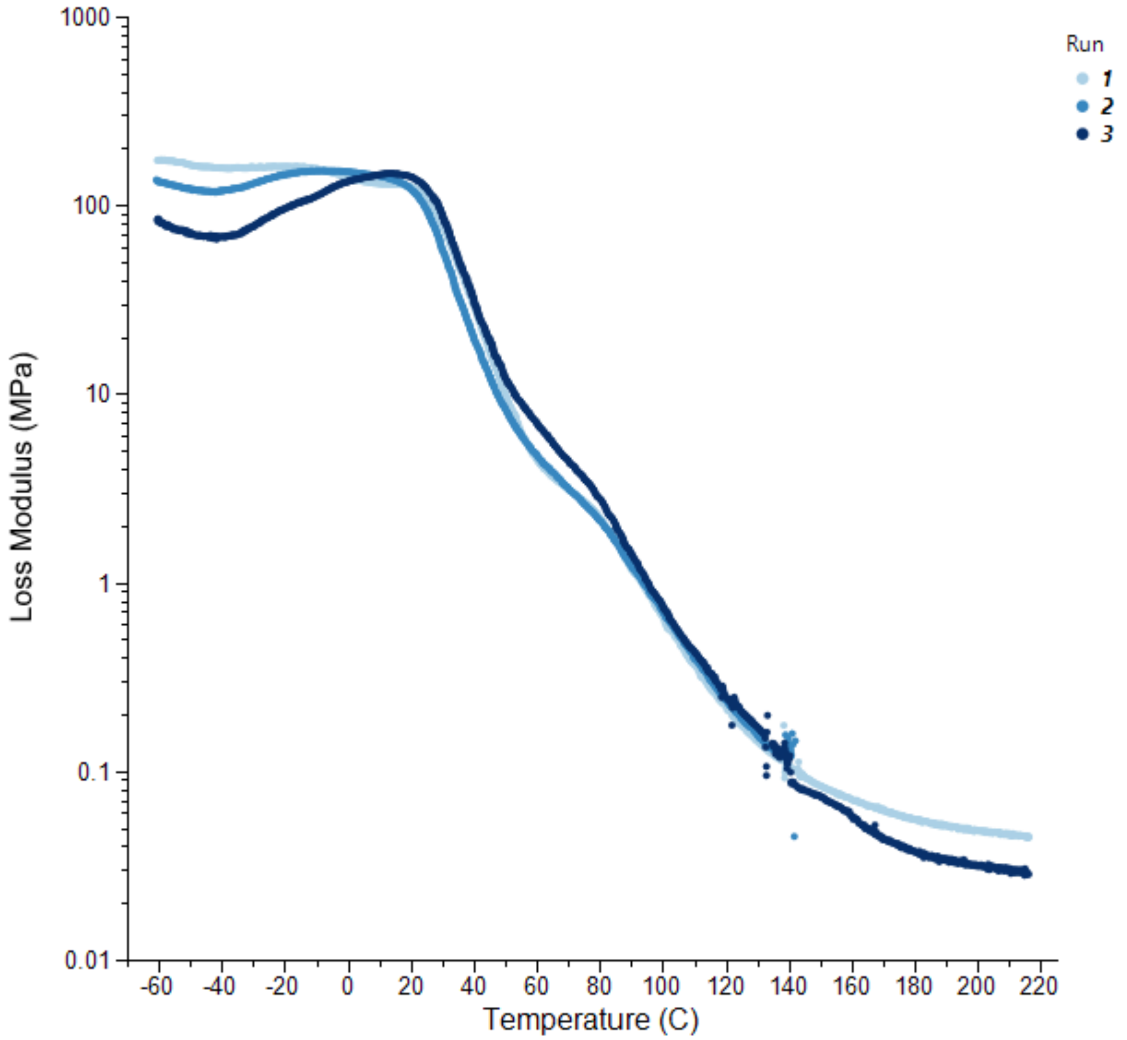

Figure S83. Loss modulus traces of pDCPD doped with 33% v/v **iPrSi7.**

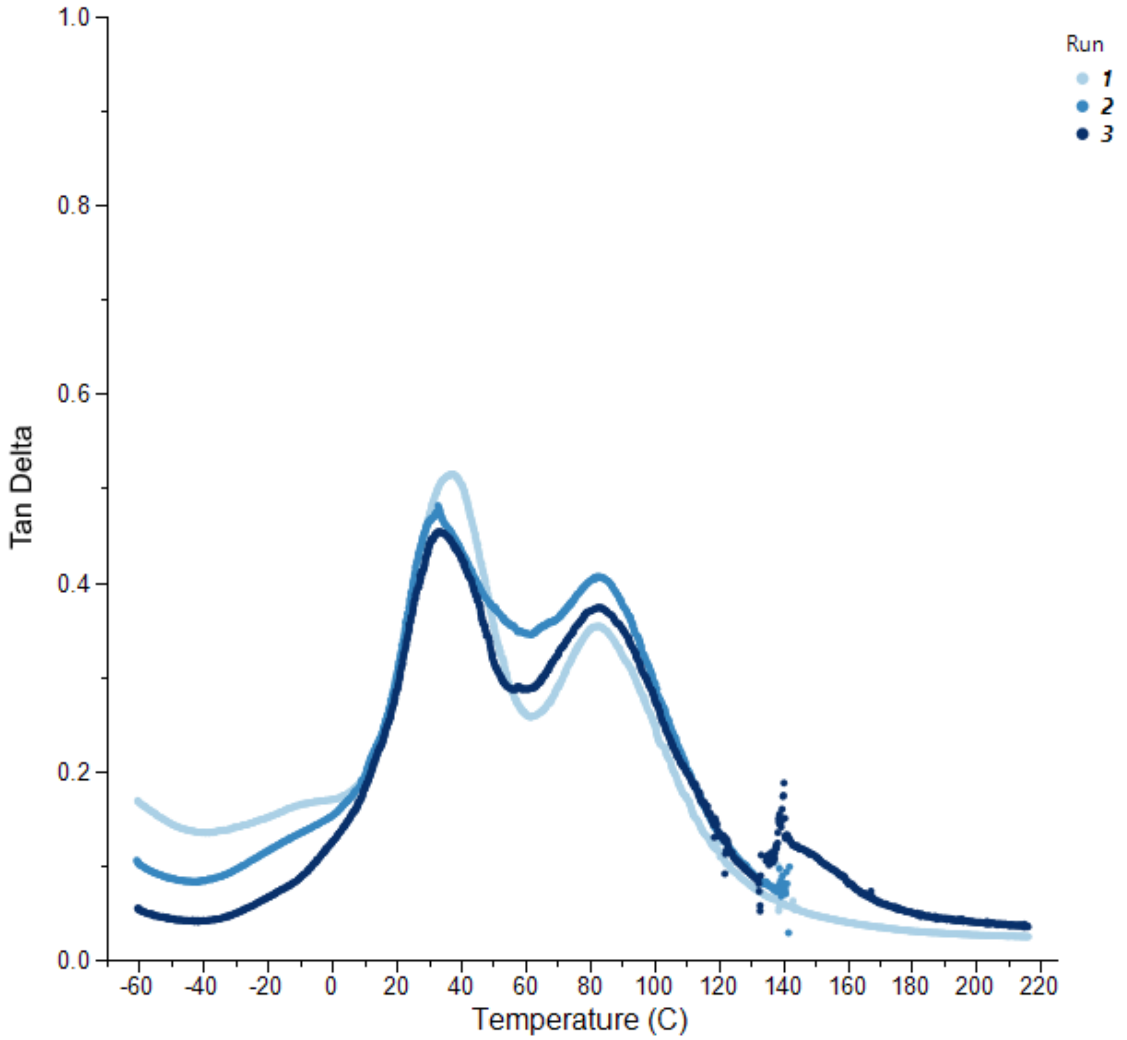

Figure S84. Tan delta traces of pDCPD doped with 33% v/v **iPrSi7.**

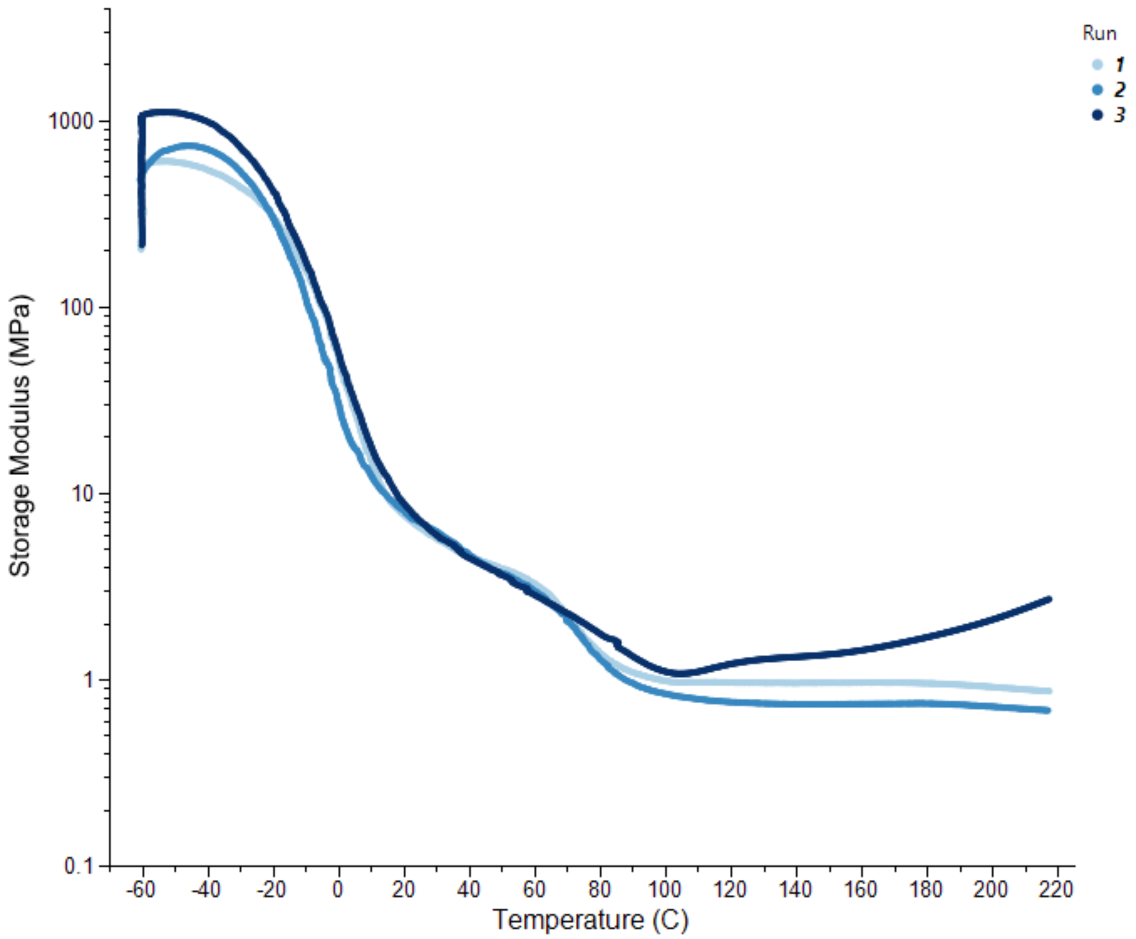

Figure S85. Storage modulus traces of pDCPD doped with 50% v/v **iPrSi7.**

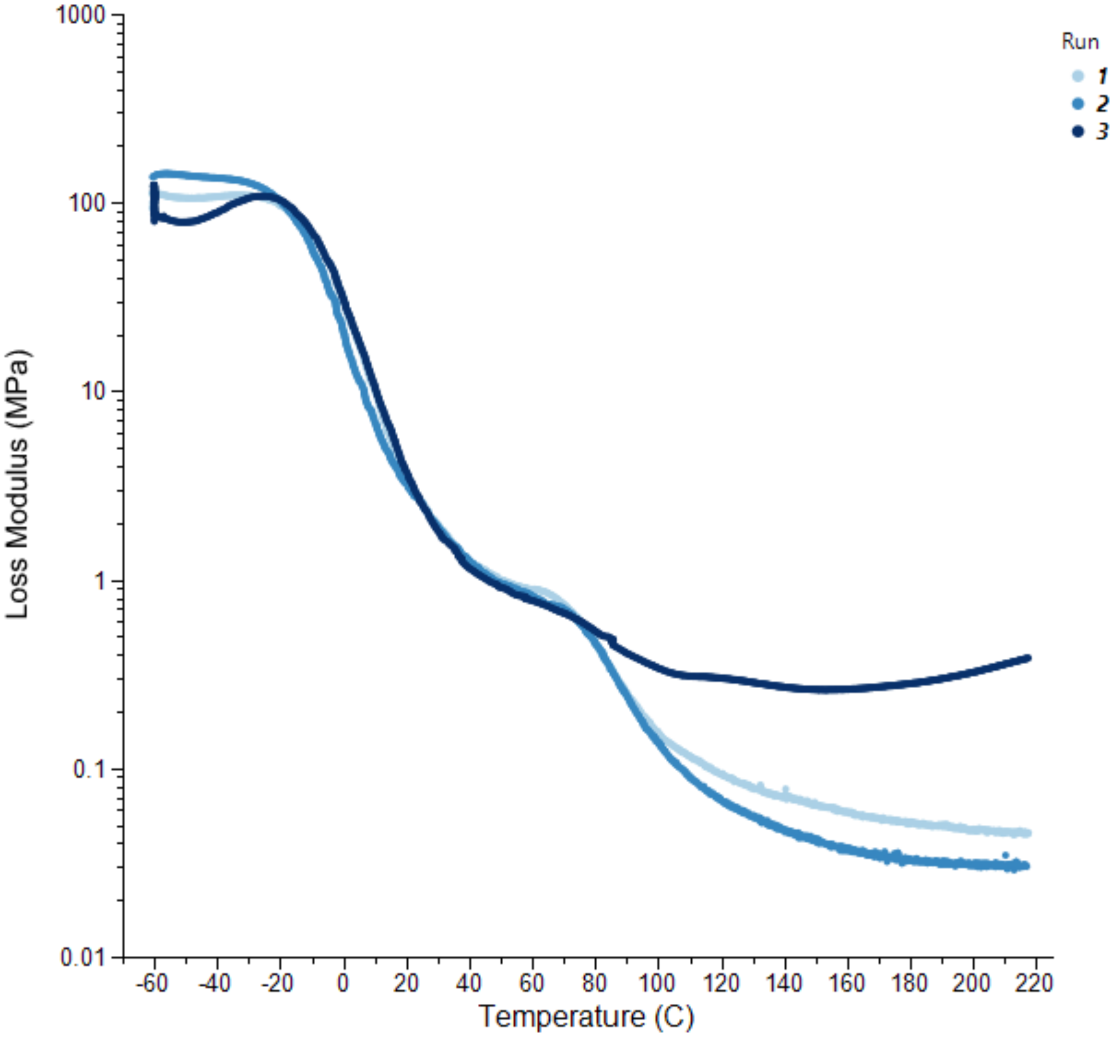

Figure S86. Loss modulus traces of pDCPD doped with 50% v/v **iPrSi7.**

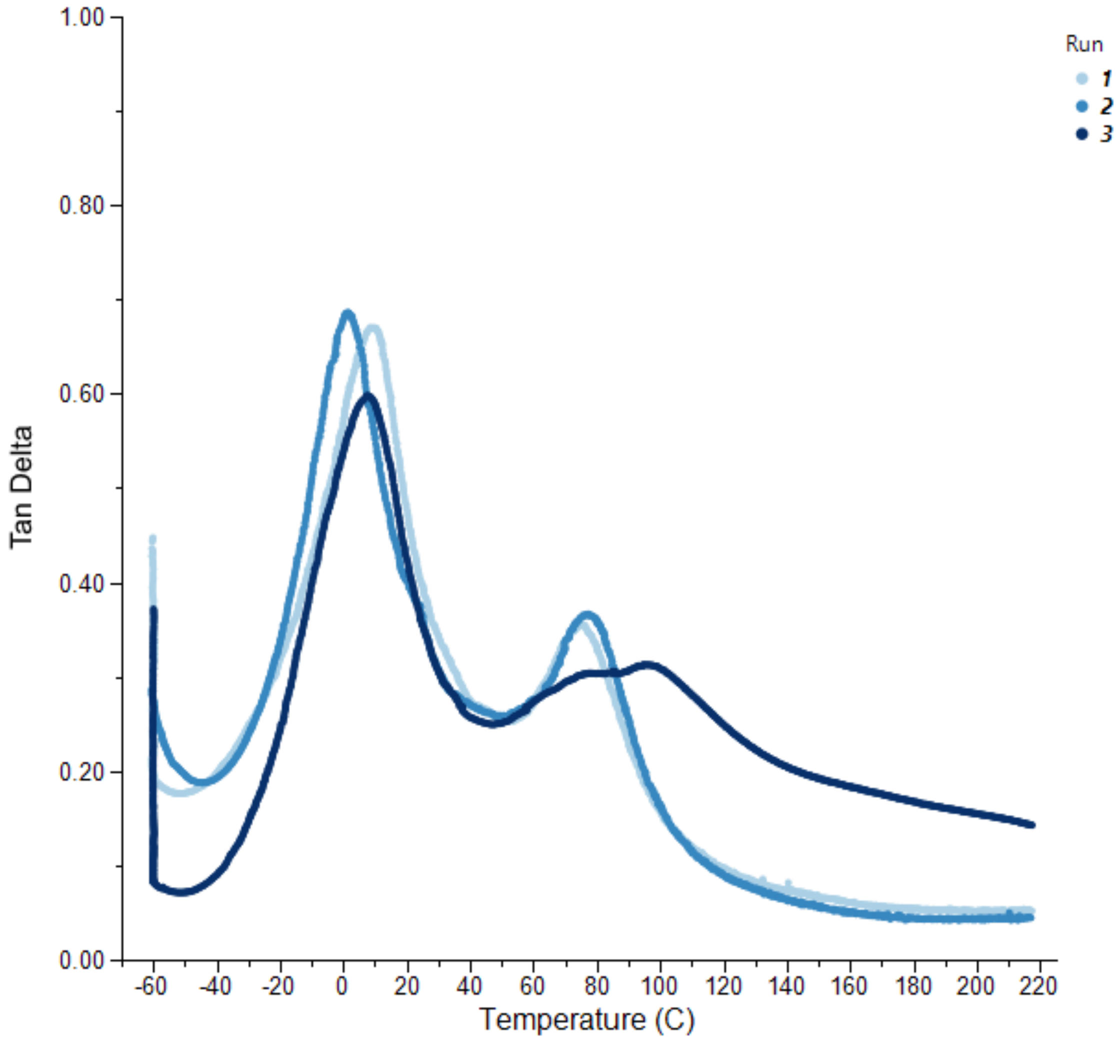

Figure S87. Tan delta traces of pDCPD doped with 50% v/v **iPrSi7.**

Training Results

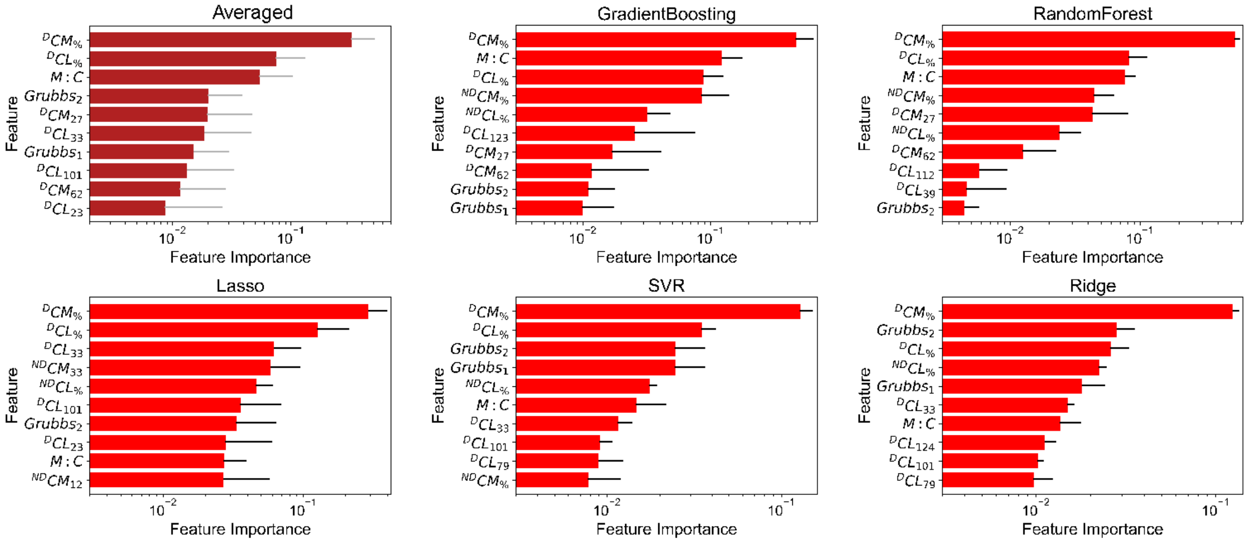

Figure S88. Feature importance averaged across all and individual fingerprint-based models. Each index, i.e., the subscript number, refers to a particular substructure in the extended connectivity fingerprint for the degradable/non-degradable comonomer/cross-linker. For example, $^{D}CM_{11}$ refers to the substructure at the 11th position of the fingerprint for a degradable comonomer. Superscript abbreviations - D: Degradable, ND: Non-degradable; Plain text - CM: Co-monomer, CL: Crosslinker, M : C: Monomer to catalyst ratio. Subscript – ECFP indices.

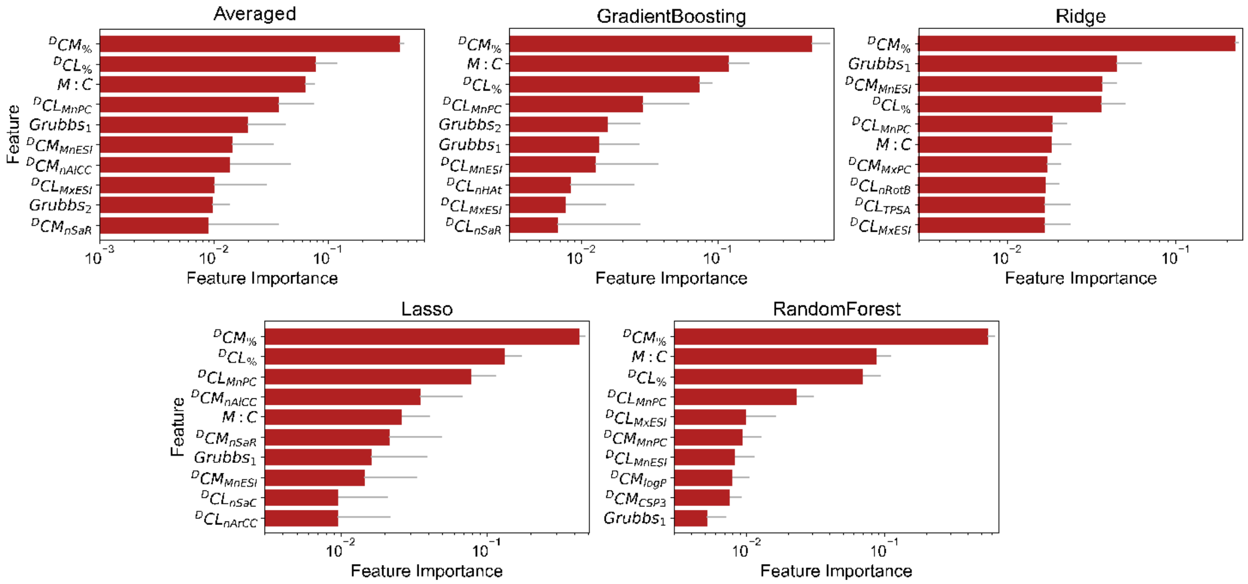

Figure S89. Feature importance averaged across all and individual descriptor-based models. In the y-axis, the descriptor for the specific degradable/non-degradable comonomer/cross-linker has been noted. For example, $^{D}CM_{nArCC}$ refers to the number of aromatic carbocycles for a degradable comonomer. Superscript abbreviations - D: degradable, ND: non-degradable; plain text - CM: co-monomer, CL: crosslinker, M : C: monomer to catalyst ratio, subscript – physicochemical descriptors – n (preceding): number, Mx: maximum, Mn: minimum, ESI: electrotopological state index, CSP3: fraction of sp3 carbon atoms, , PC: partial charge, Al: aliphatic, Sa: saturated, Ar: aromatic, CC: carbocycles, R: rings, G: hetero, At: atoms, Rot: rotatable, B: bonds, TPSA: topological polar surface area, P: partition coefficient.

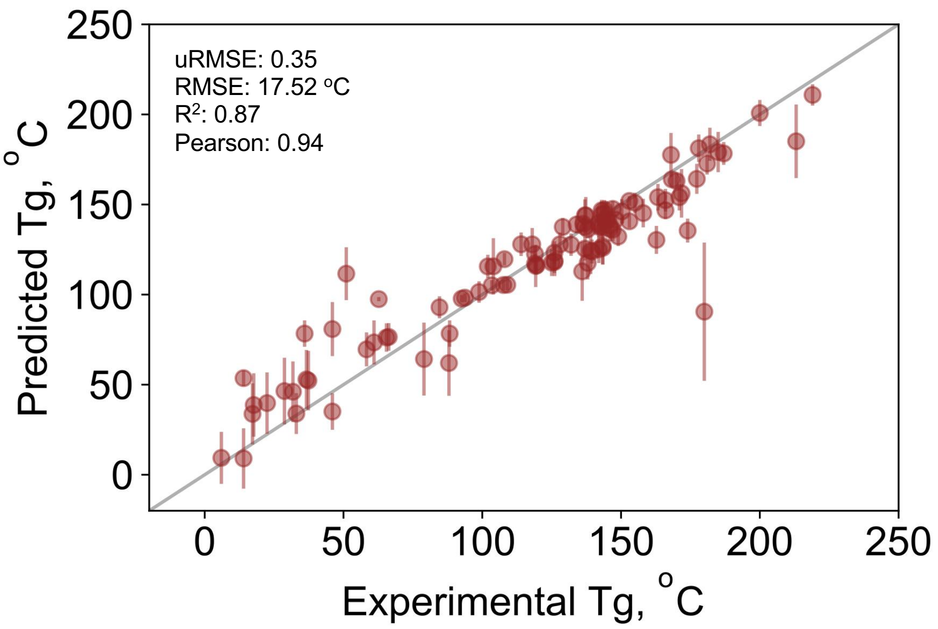

Figure S90. Parity plot for descriptor-based model shows good agreement of predicted and experimental Tg, for the training dataset, albeit with a higher standard deviation than fingerprint-based models. The inset text notes the model ensemble performance metrics. Error bars note standard deviation of the Tg predicted using the different models in the ensemble.

Experimental Figures

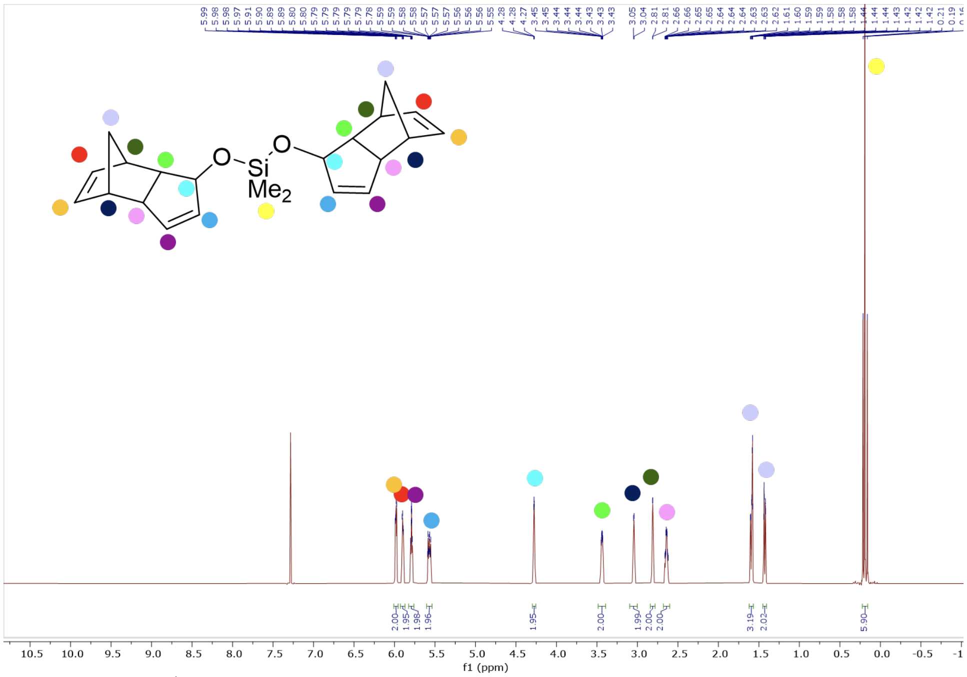

Figure S91. ^{1}H NMR of **DDMS**.

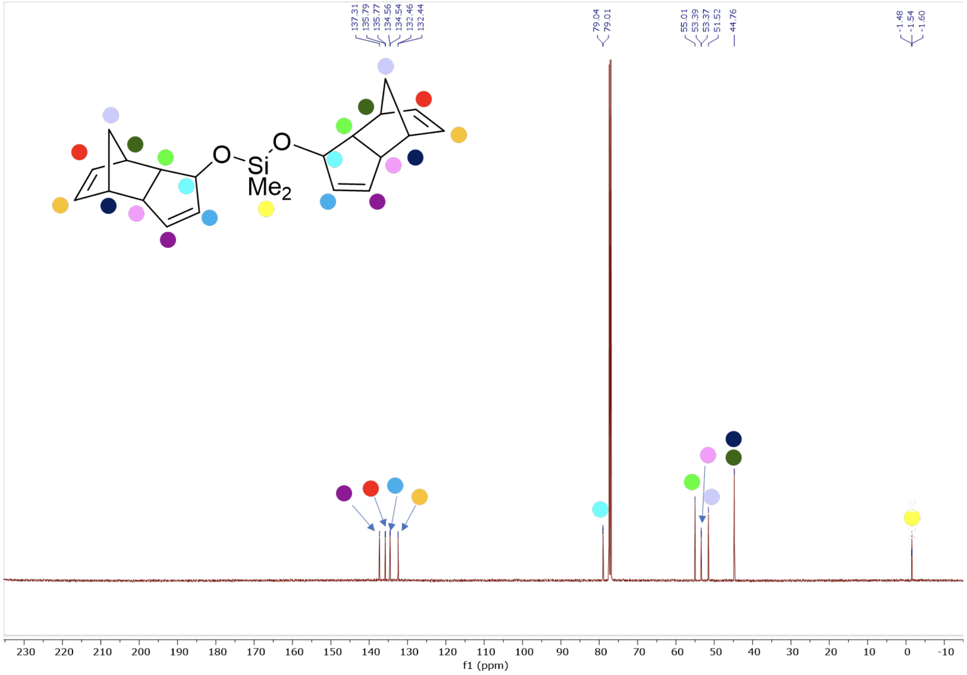

Figure S92. ^{13}C NMR of **DDMS**.

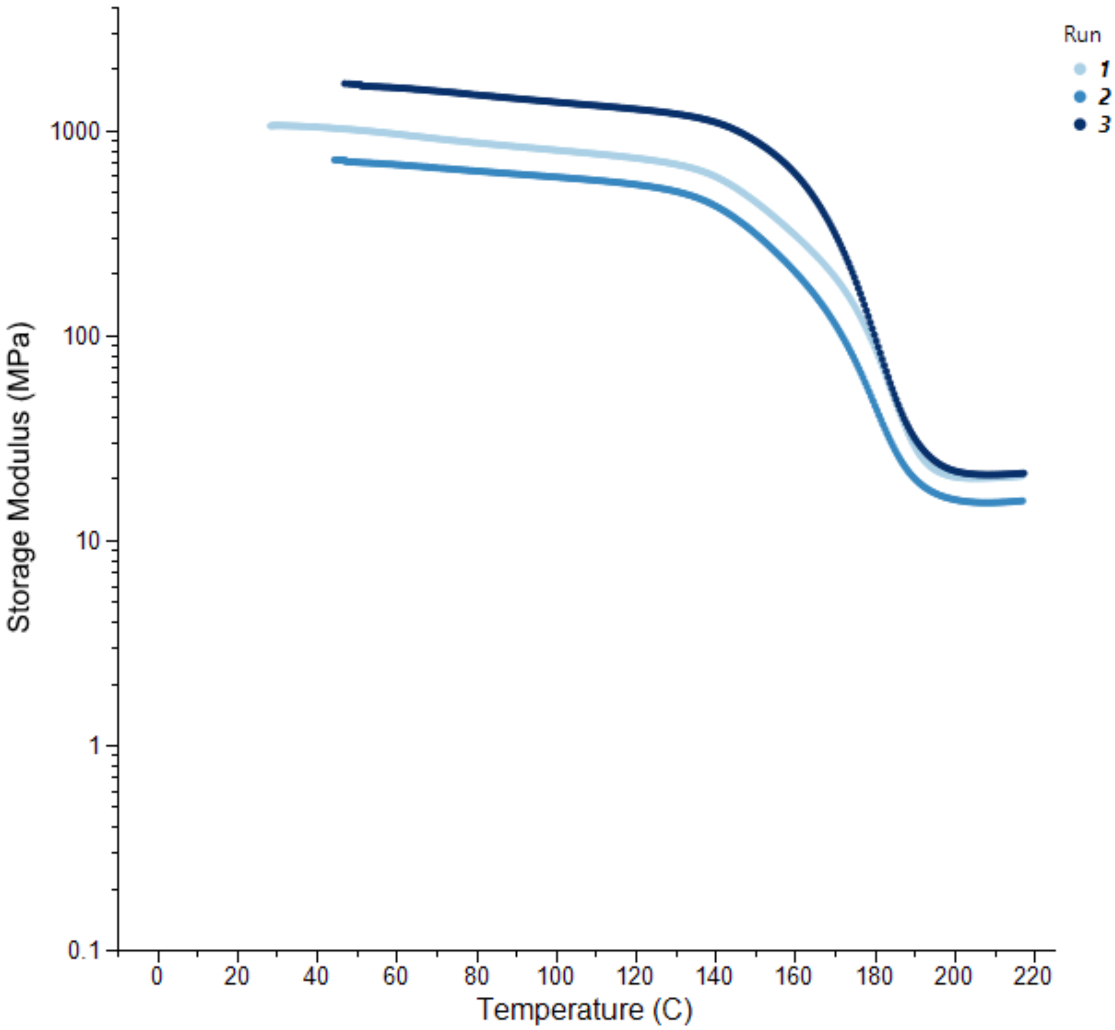

Figure S93. Storage modulus traces of pDCPD doped with 10 mol% **DDMS**.

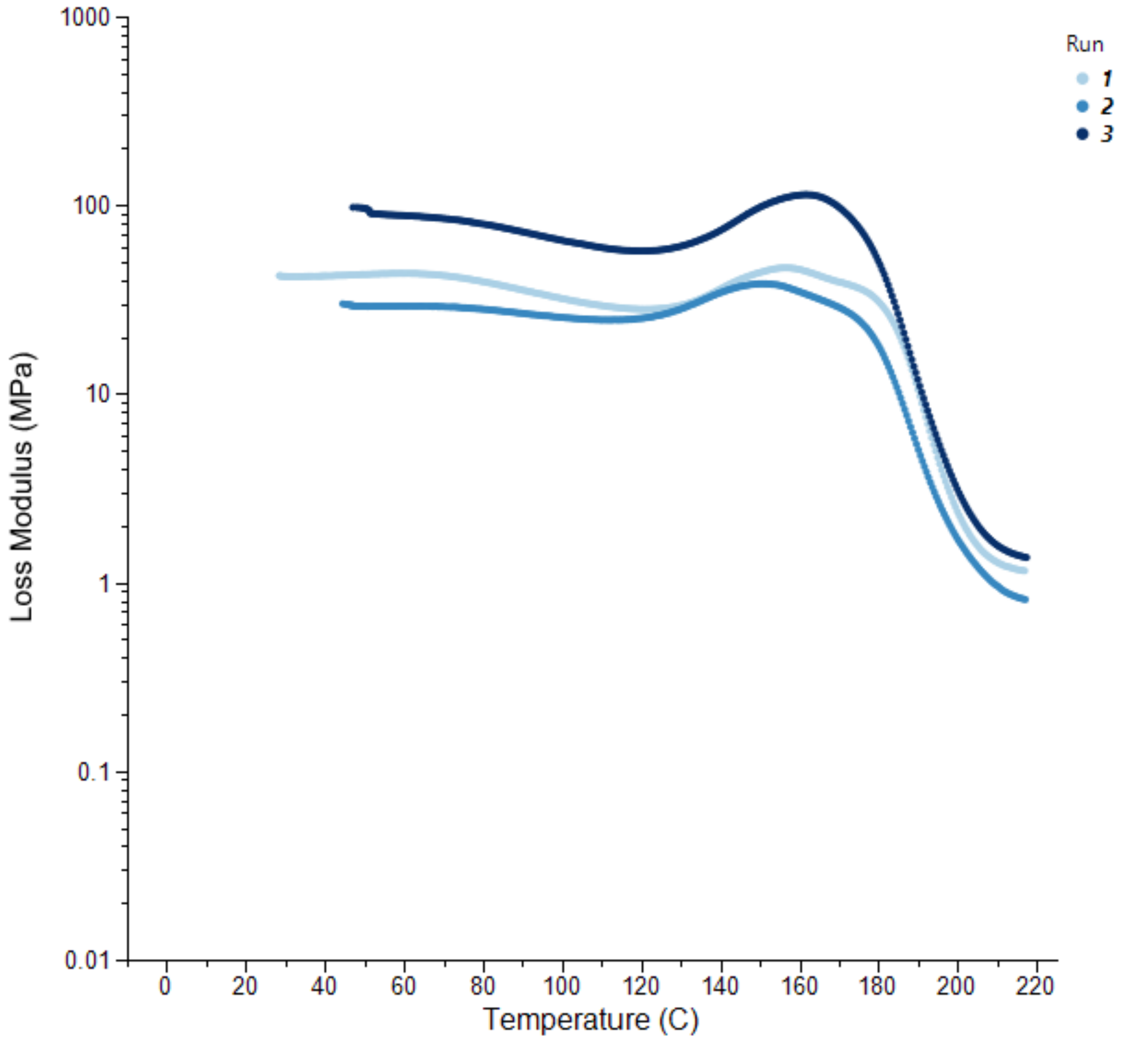

Figure S94. Loss modulus traces of pDCPD doped with 10 mol% **DDMS.**

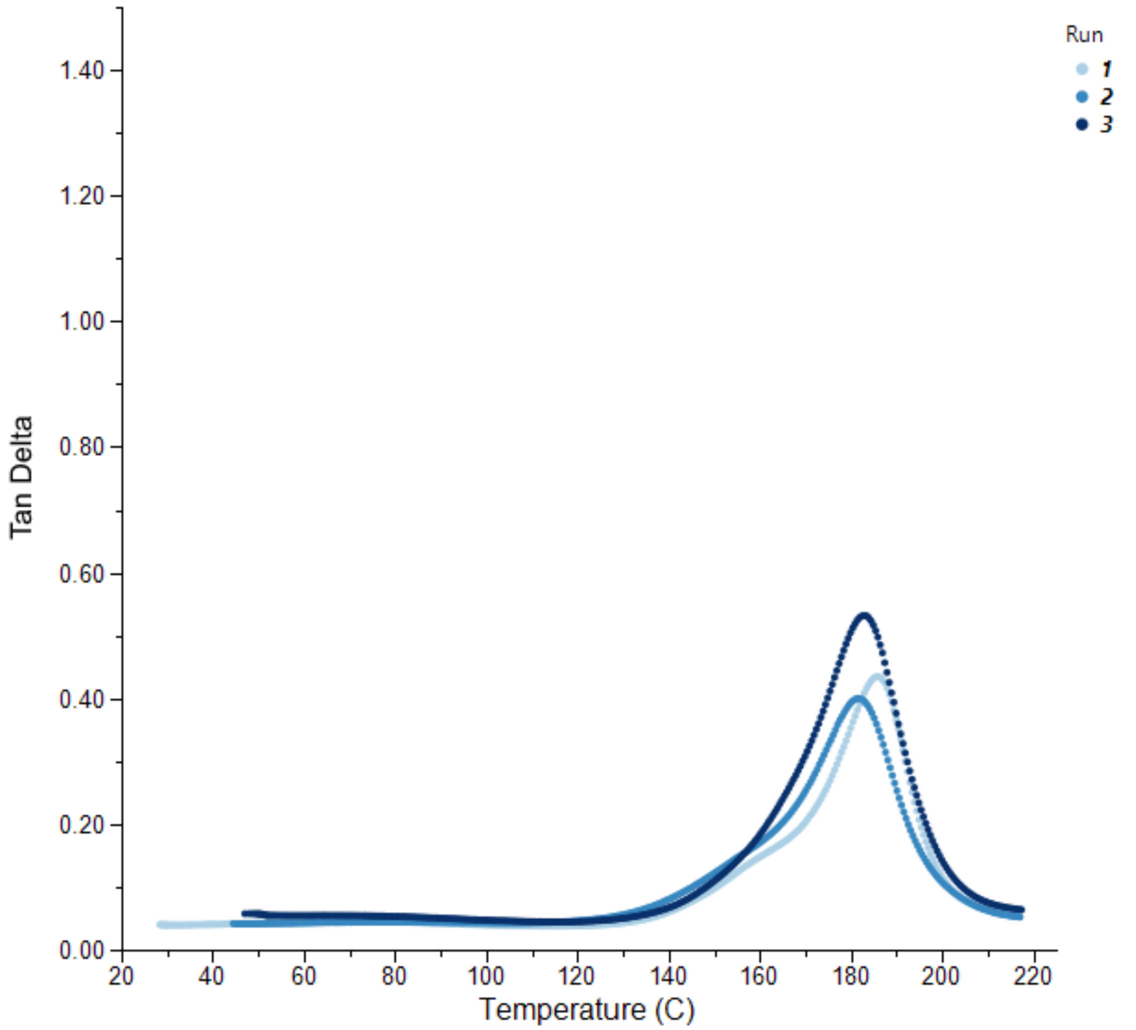

Figure S95. Tan delta traces of pDCPD doped with 10% v/v **PhSi8**.

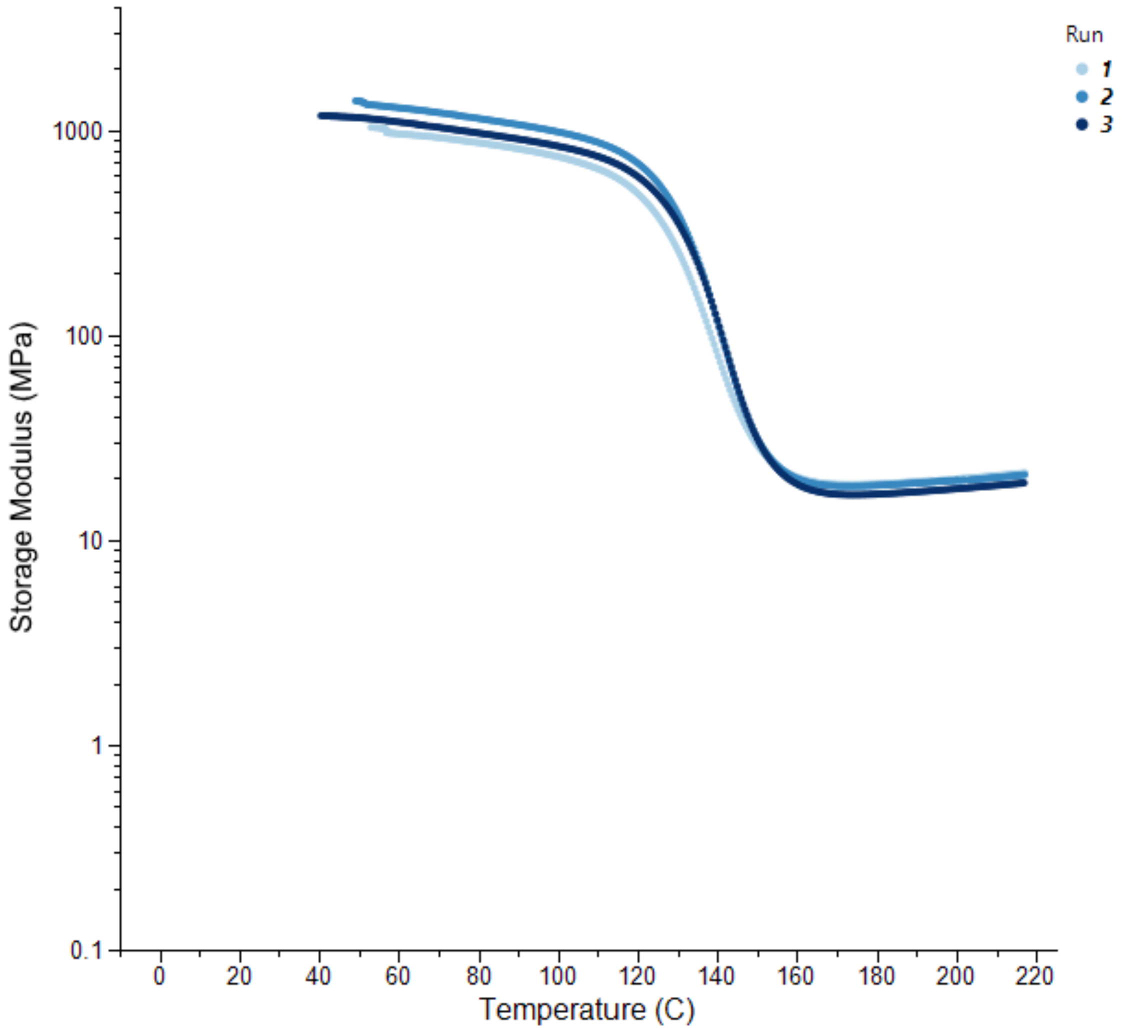

Figure S96. Storage modulus traces of pDCPD doped with 10 mol% **iPrSi8** and 10 mol% **DDMS**.

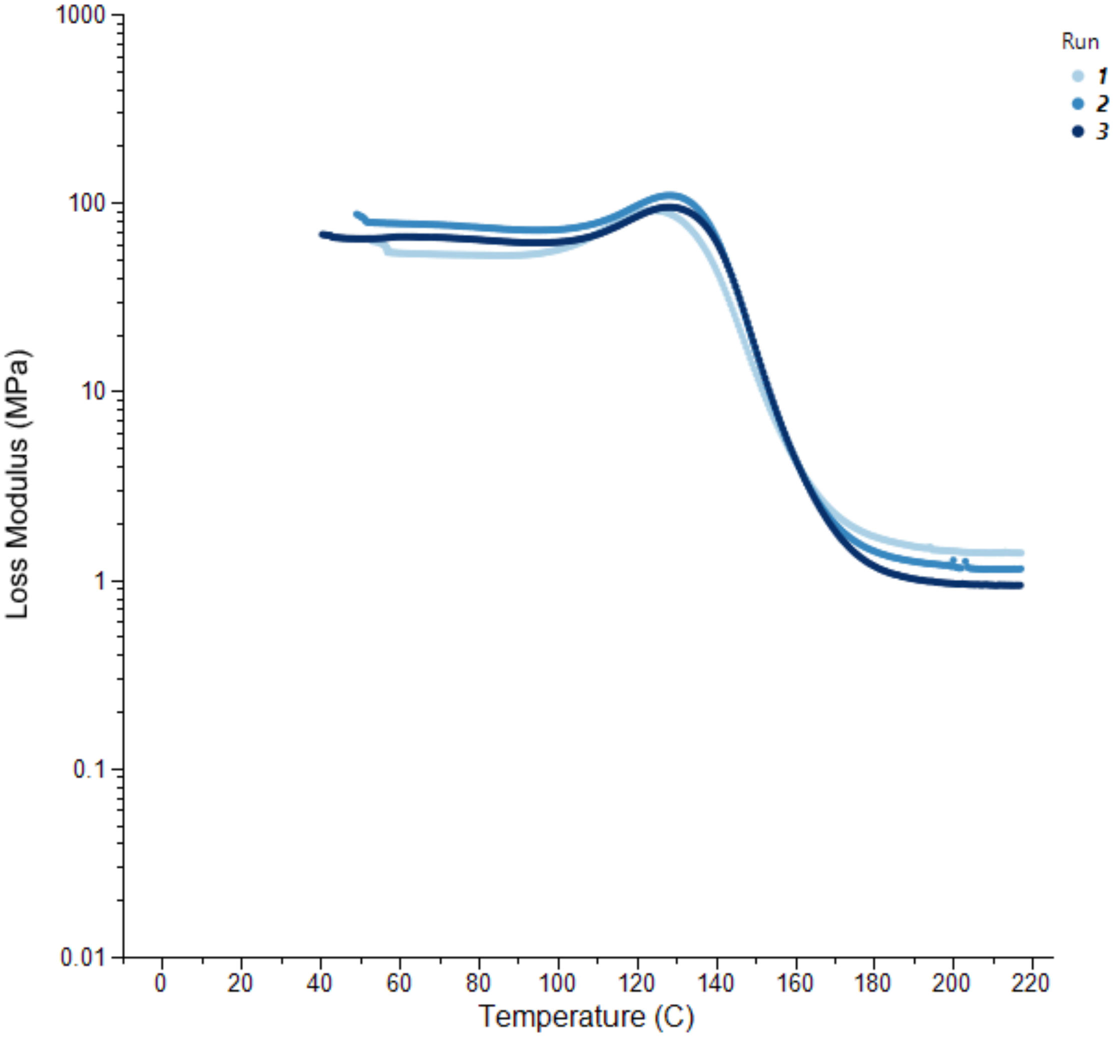

Figure S97. Loss modulus traces of pDCPD doped with 10 mol% **iPrSi8** and 10 mol% **DDMS**.

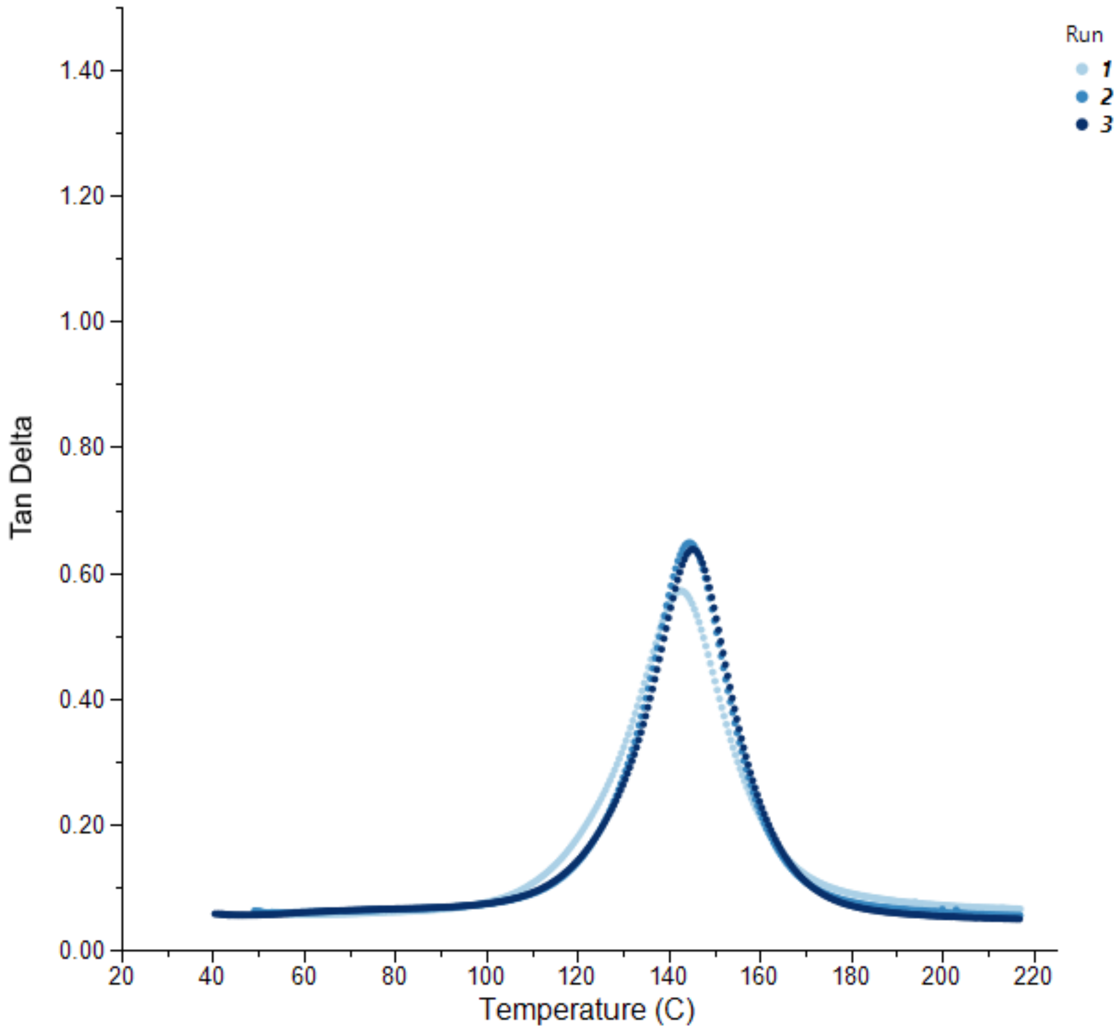

Figure S98. Tan delta traces of pDCPD doped with 10 mol% **iPrSi8** and 10 mol% **DDMS**.

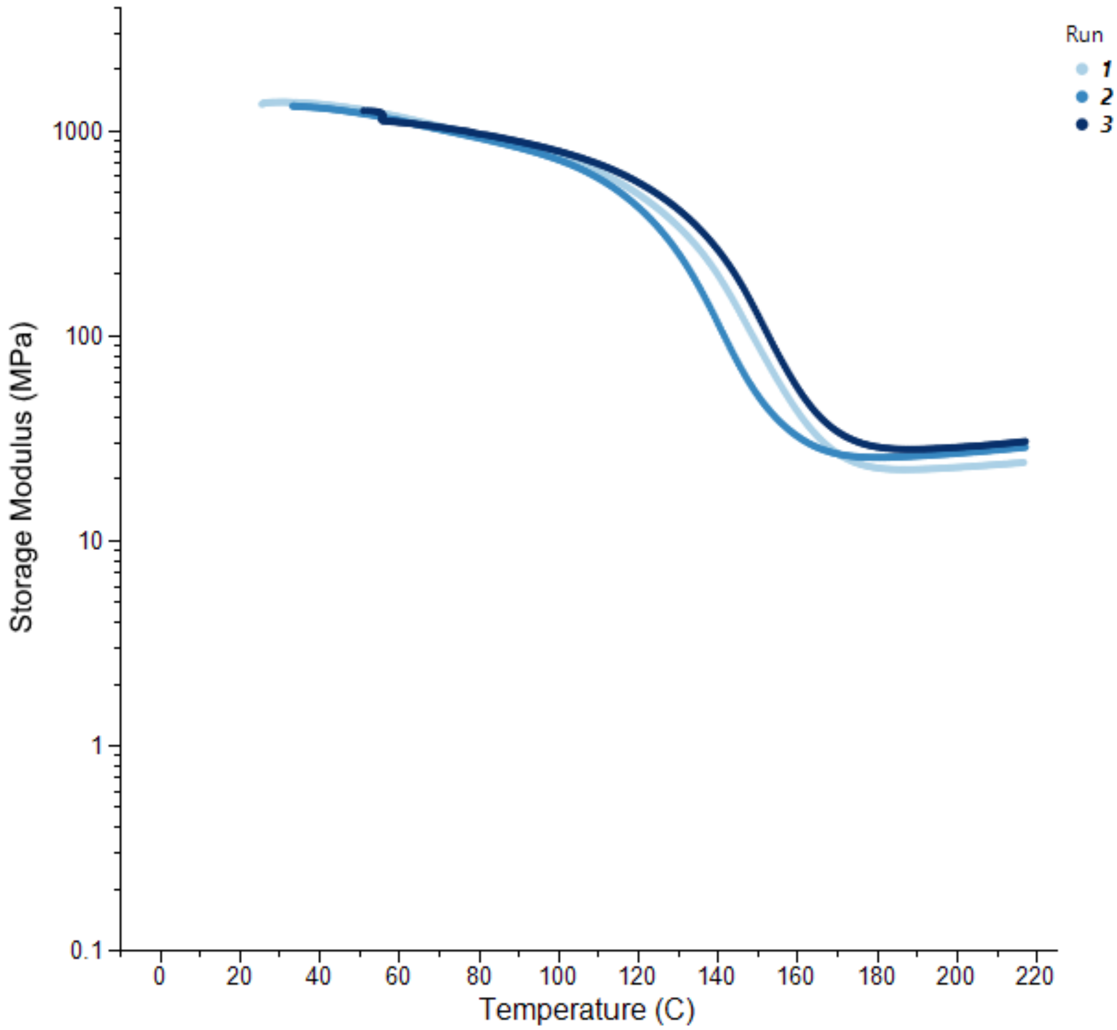

Figure S99. Storage modulus traces of pDCPD doped with 10 mol% **iPrSi8** and 20 mol% **DDMS**.

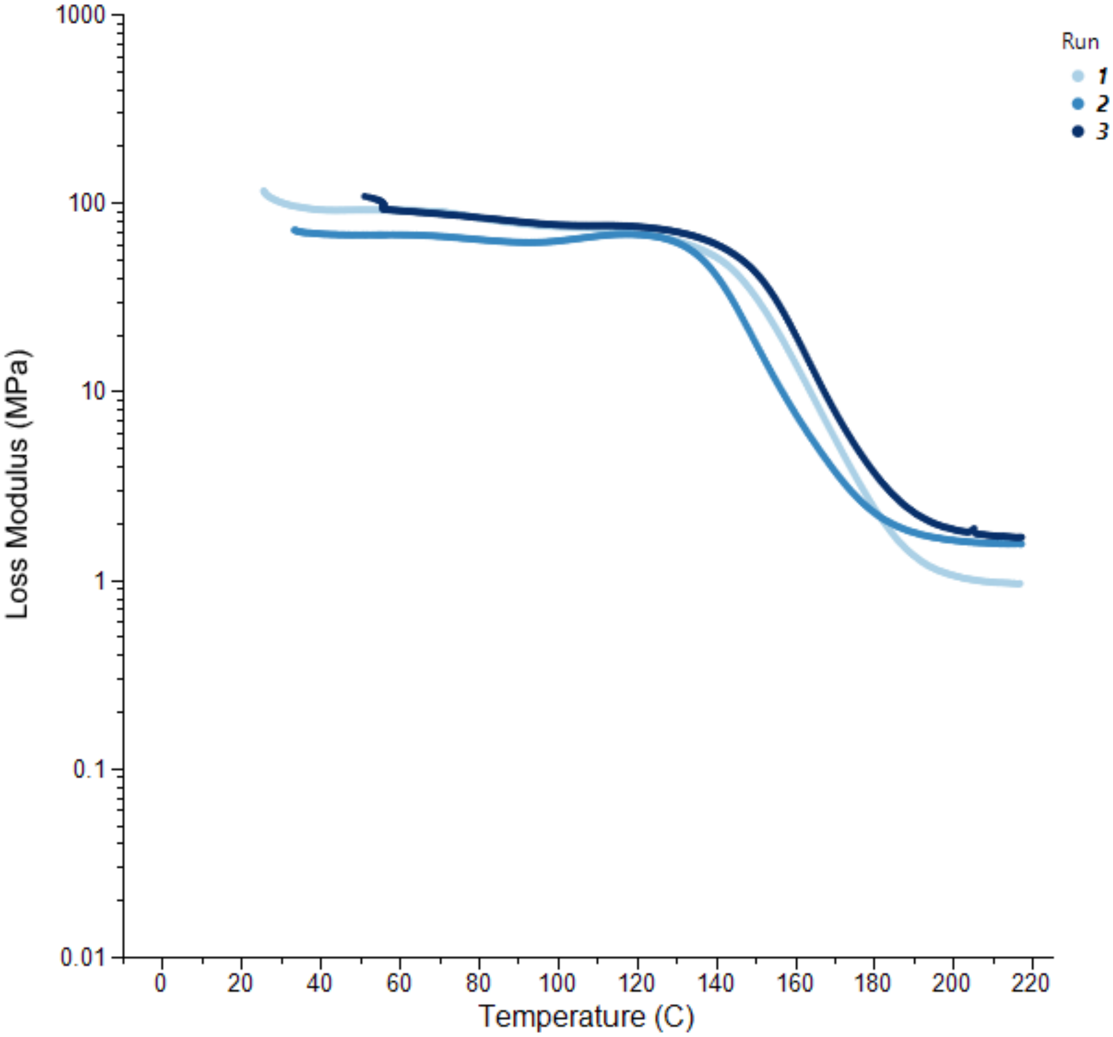

Figure S100. Loss modulus traces of pDCPD doped with 10 mol% **iPrSi8** and 20 mol% **DDMS**.

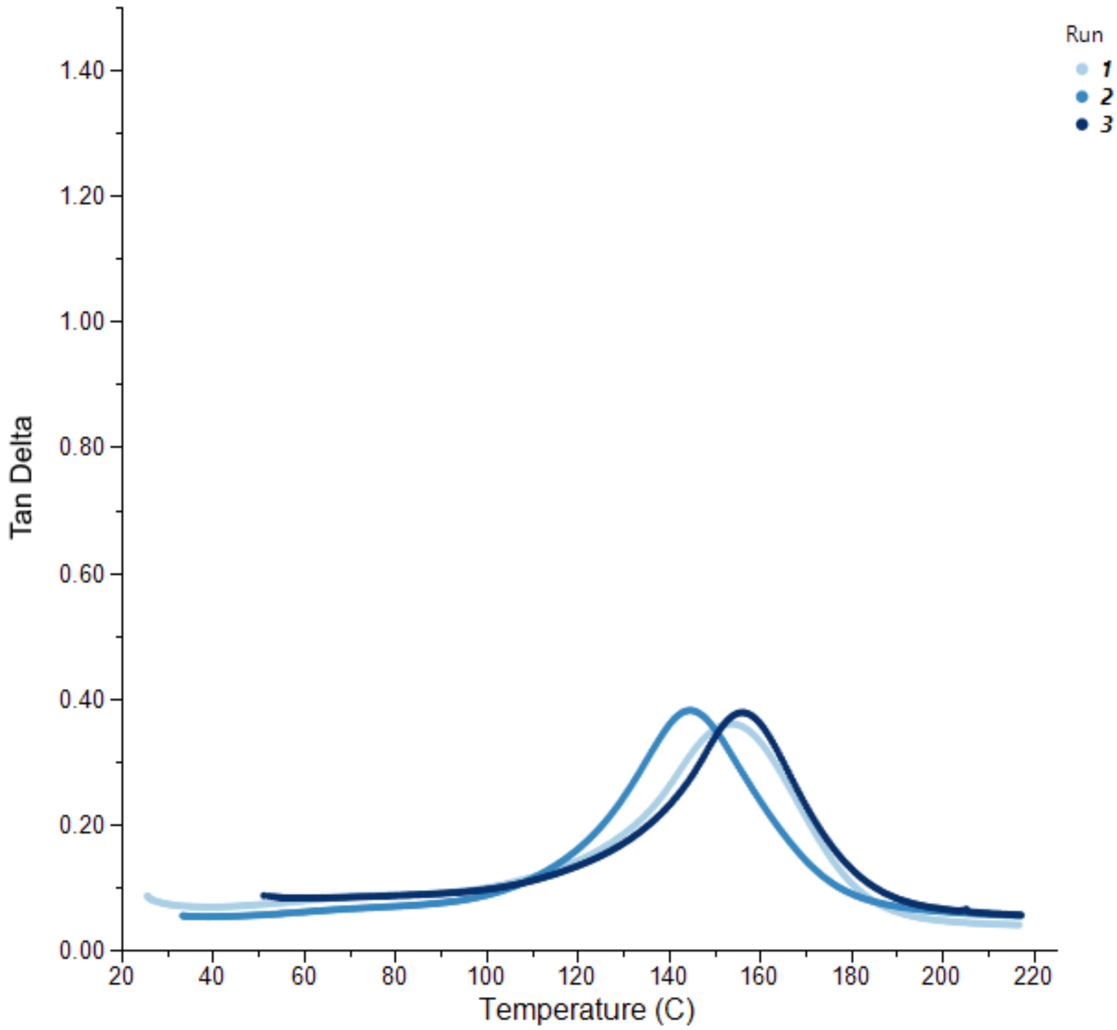

Figure S101. Tan delta traces of pDCPD doped with 10 mol% **iPrSi8** and 20 mol% **DDMS.**

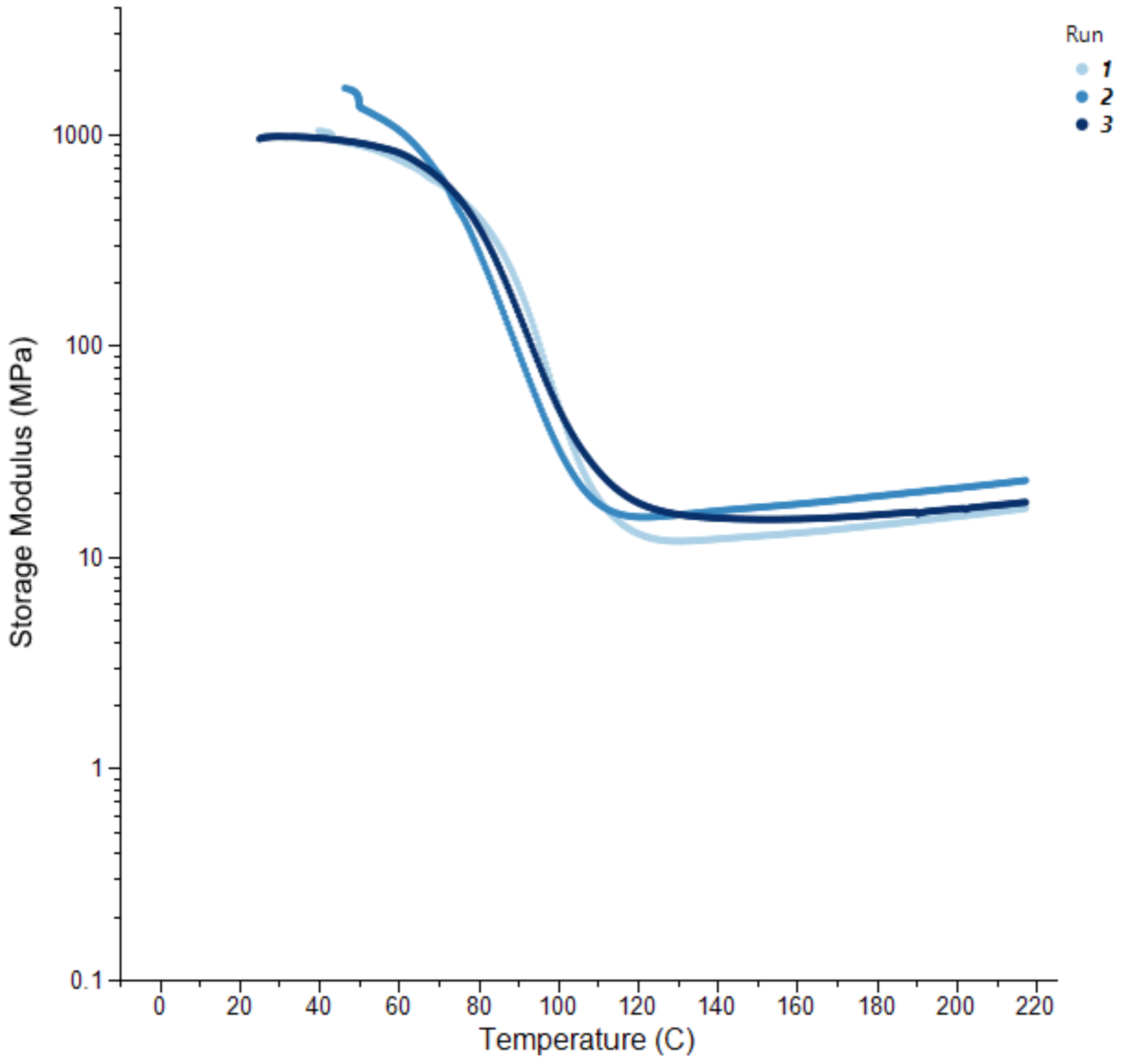

Figure S102. Storage modulus traces of pDCPD doped with 20 mol% **iPrSi8** and 10 mol% **DDMS**.

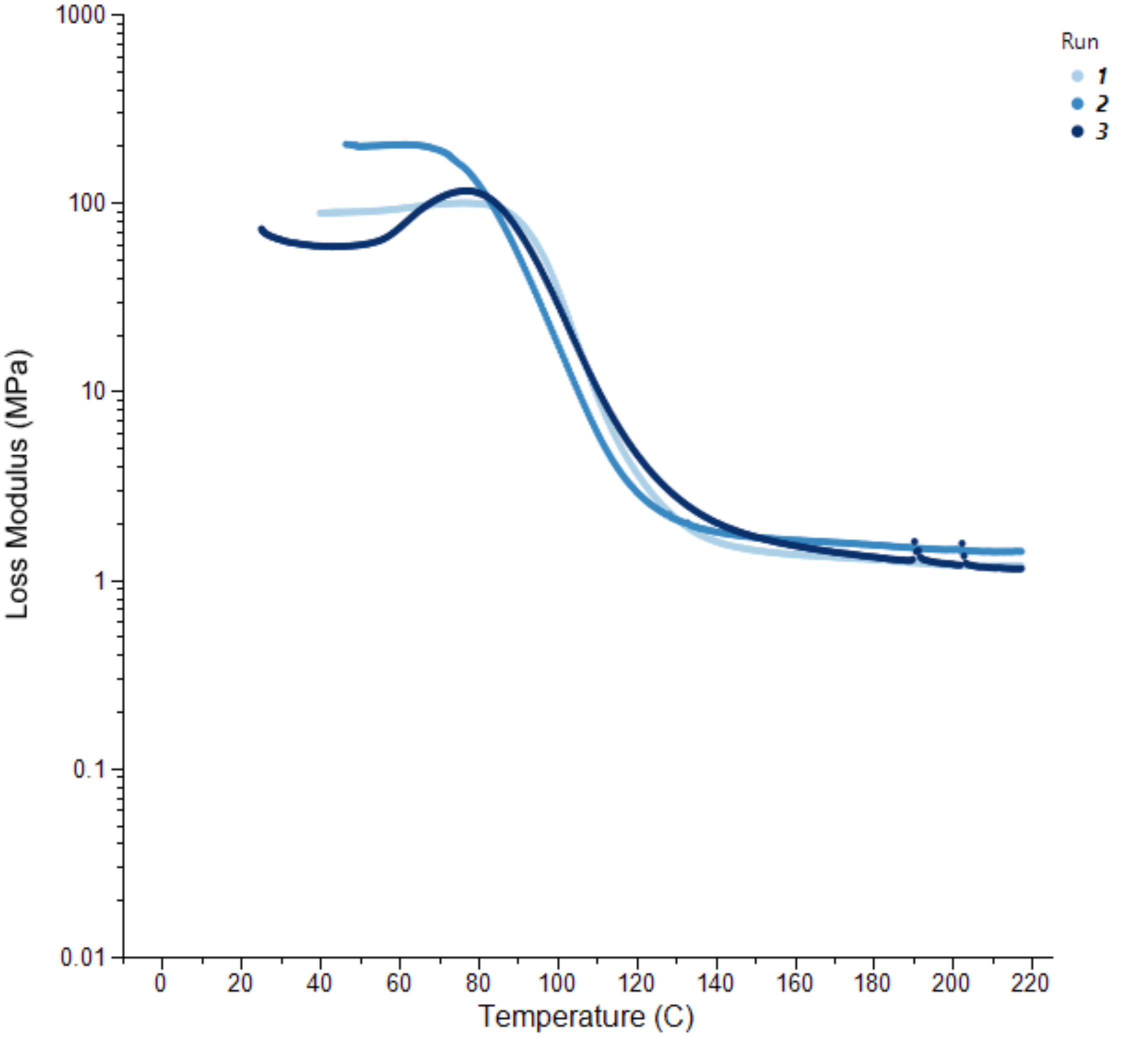

Figure S103. Loss modulus traces of pDCPD doped with 20 mol% **iPrSi8** and 10 mol% **DDMS**.

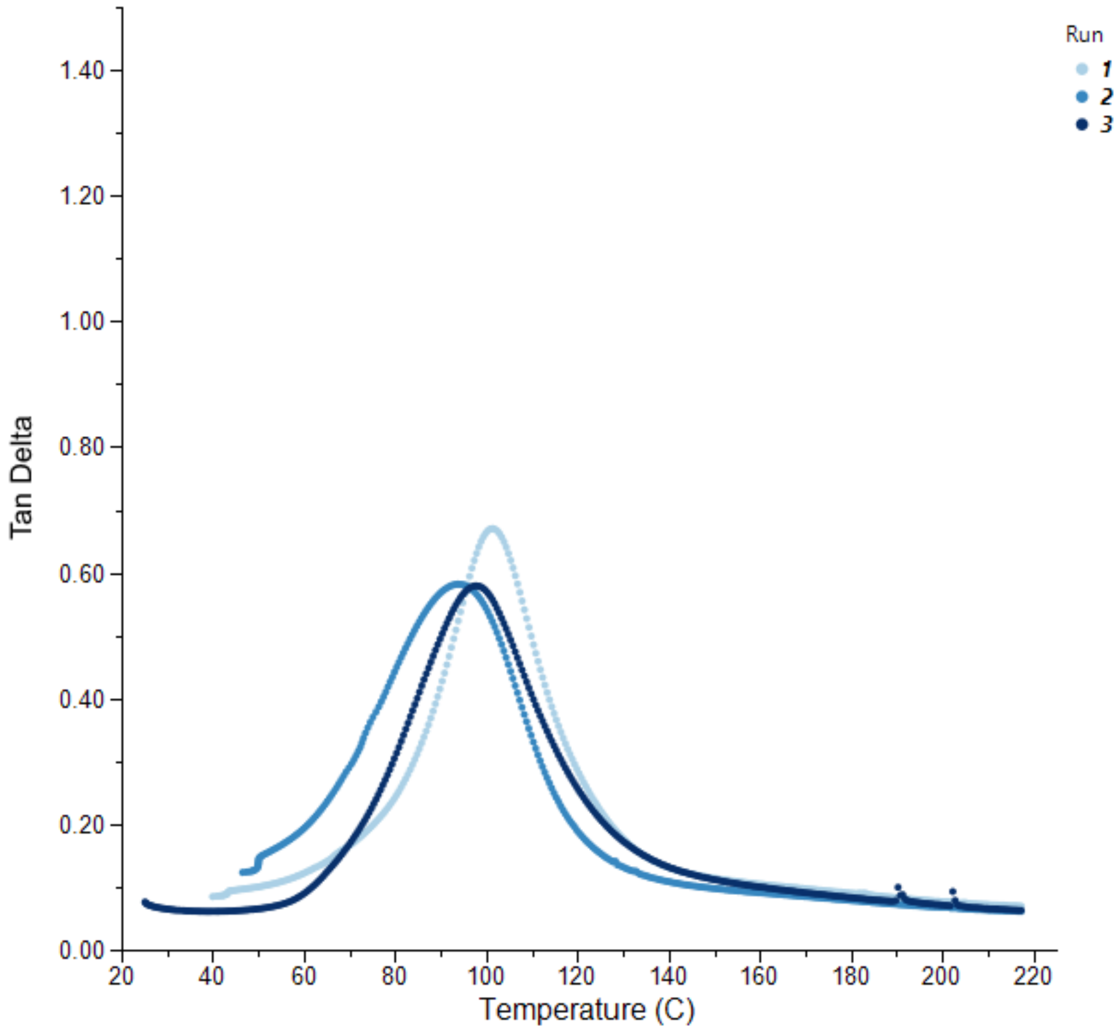

Figure S104. Tan delta traces of pDCPD doped with 20 mol% **iPrSi8** and 10 mol% **DDMS**.

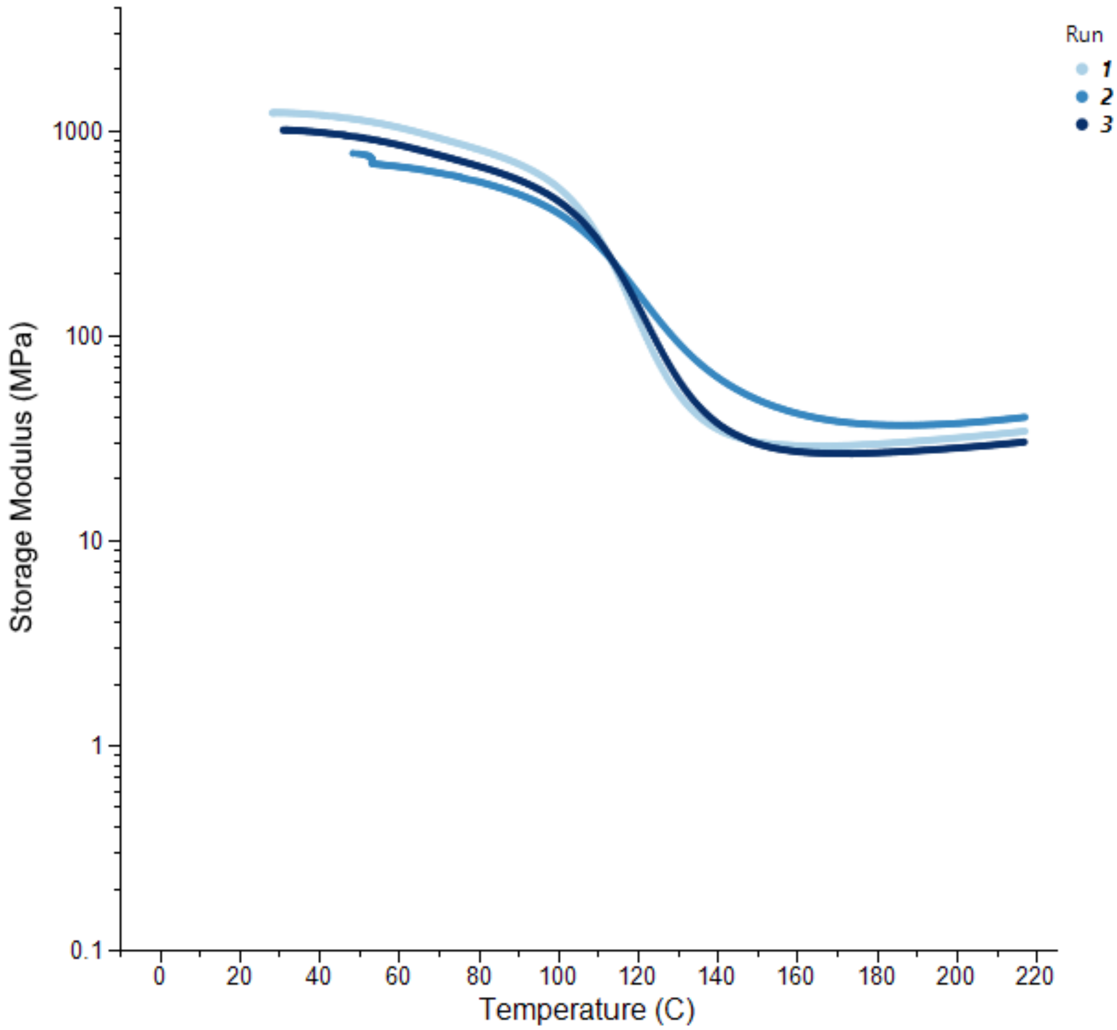

Figure S105. Storage modulus traces of pDCPD doped with 20 mol% **iPrSi8** and 20 mol% **DDMS**.

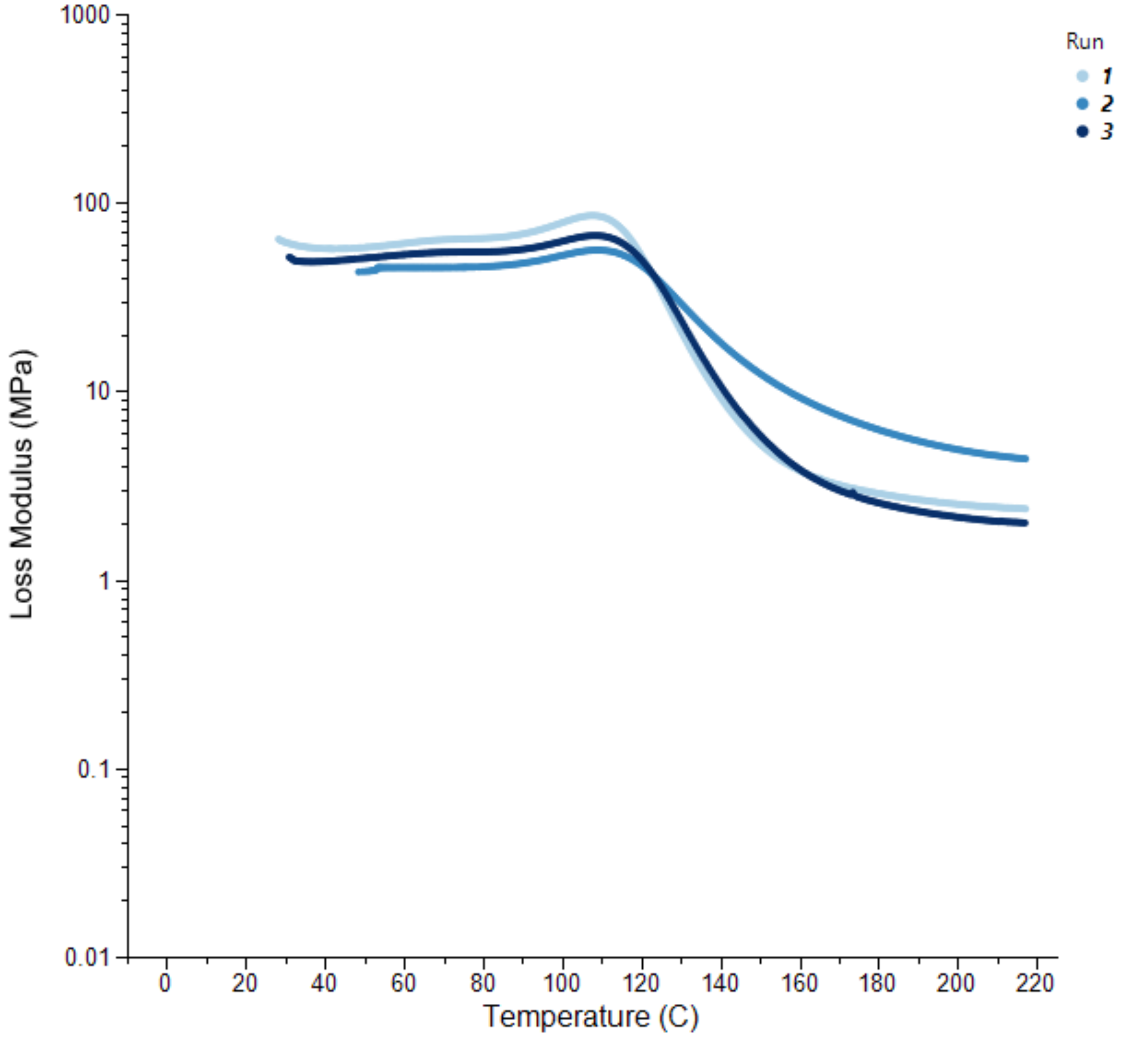

Figure S106. Loss modulus traces of pDCPD doped with 20 mol% **iPrSi8** and 20 mol% **DDMS**.

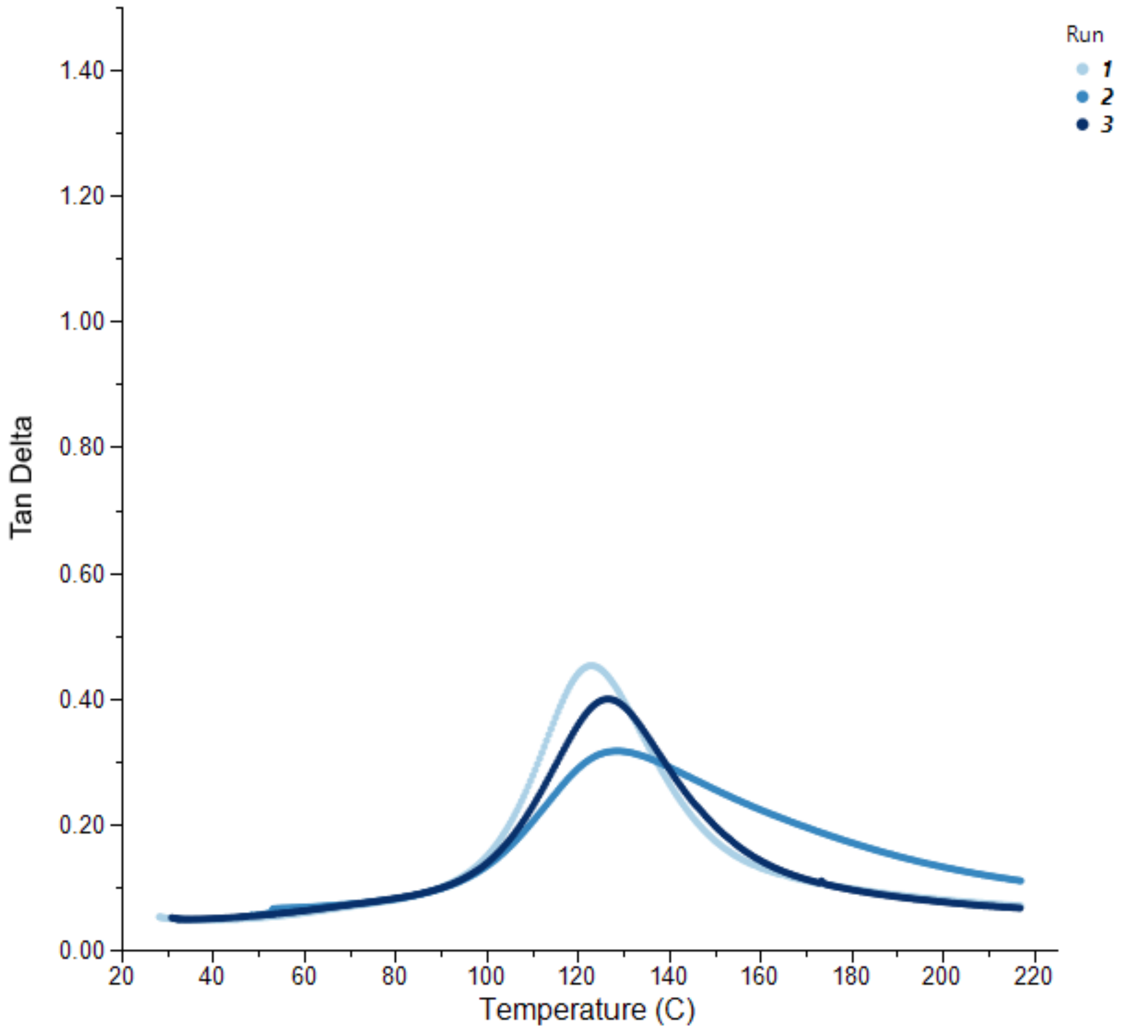

Figure S107. Tan delta traces of pDCPD doped with 20 mol% **iPrSi8** and 20 mol% **DDMS**.

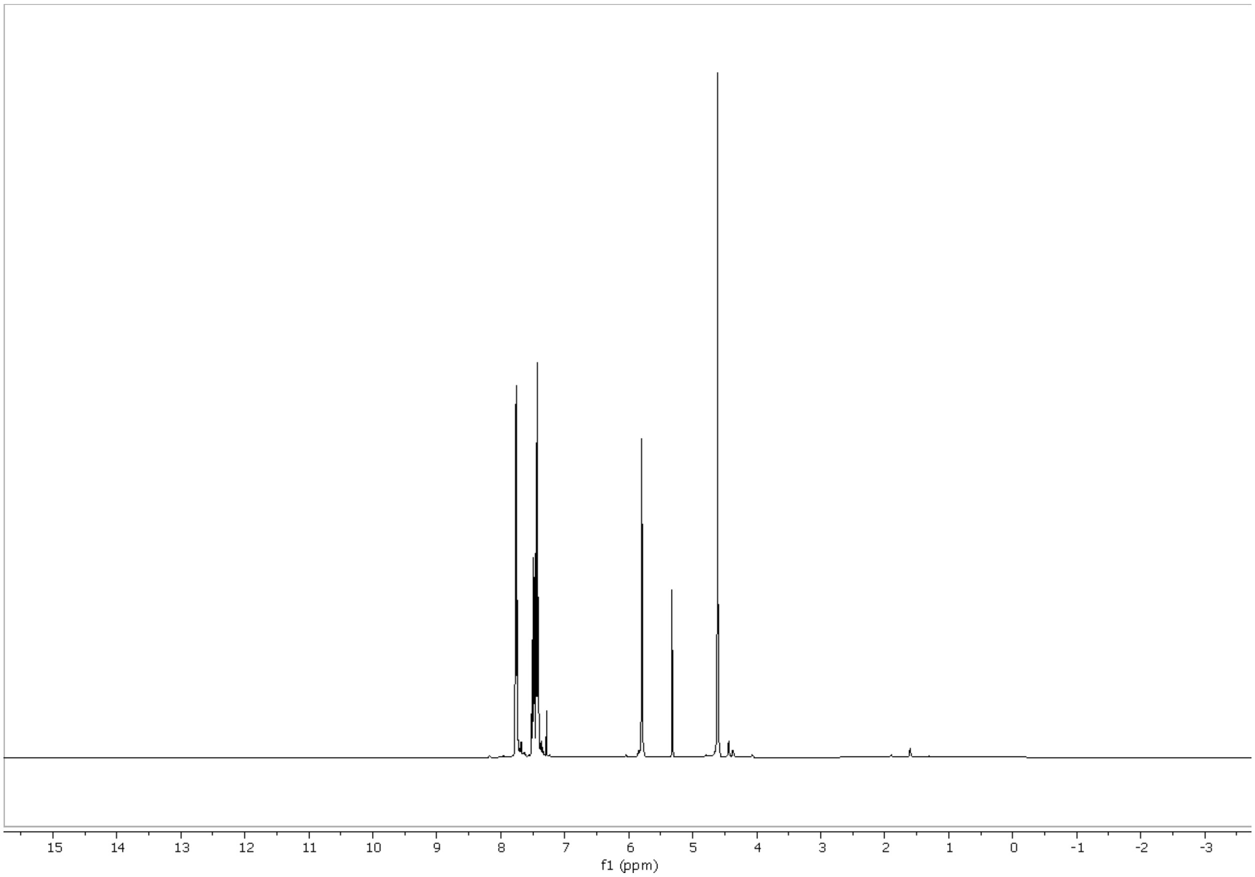

Figure S108. ^{1}H NMR of **PhSi7**.

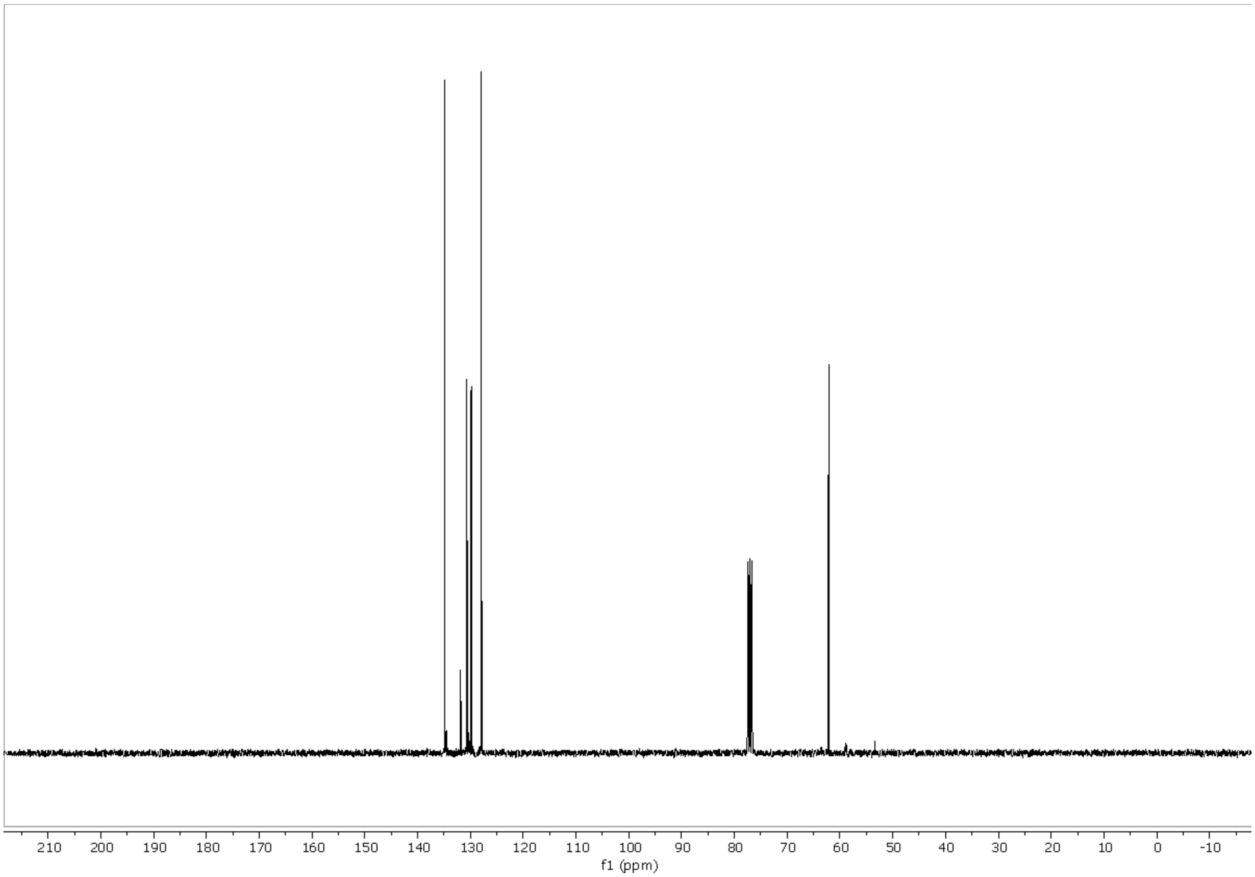

Figure S109. ^{13}C NMR of **PhSi7.**

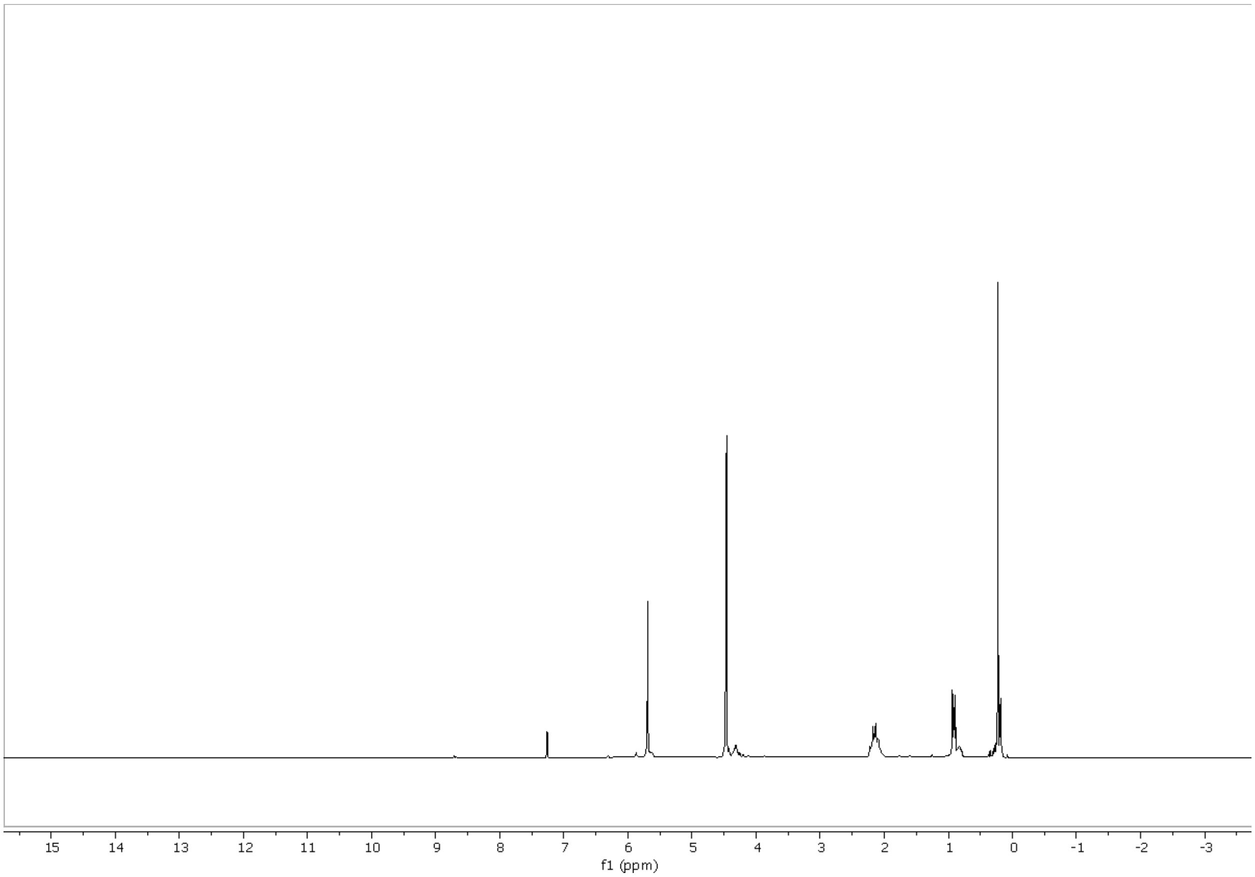

Figure S110. ^{1}H NMR of **LinF7**.

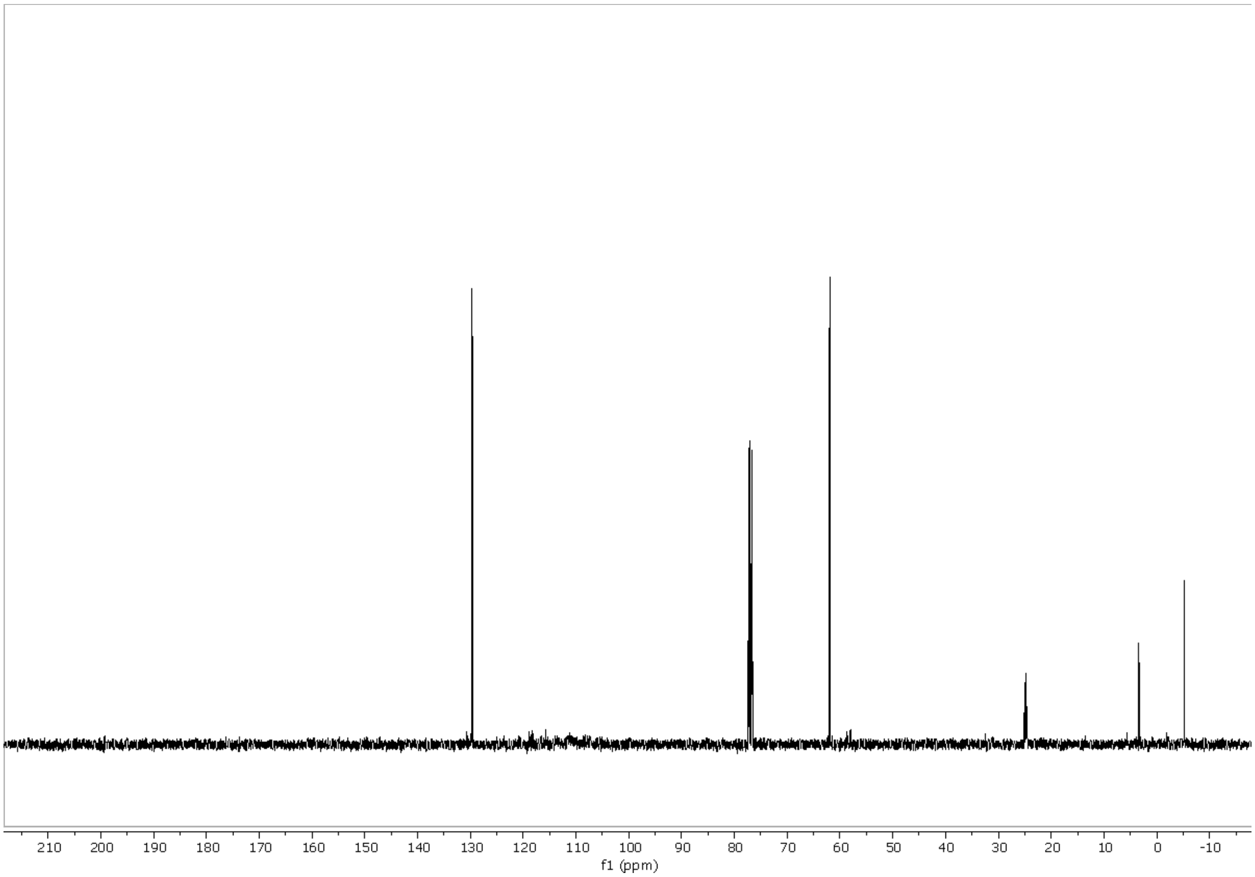

Figure S111. ^{13}C NMR of **LinF7**.

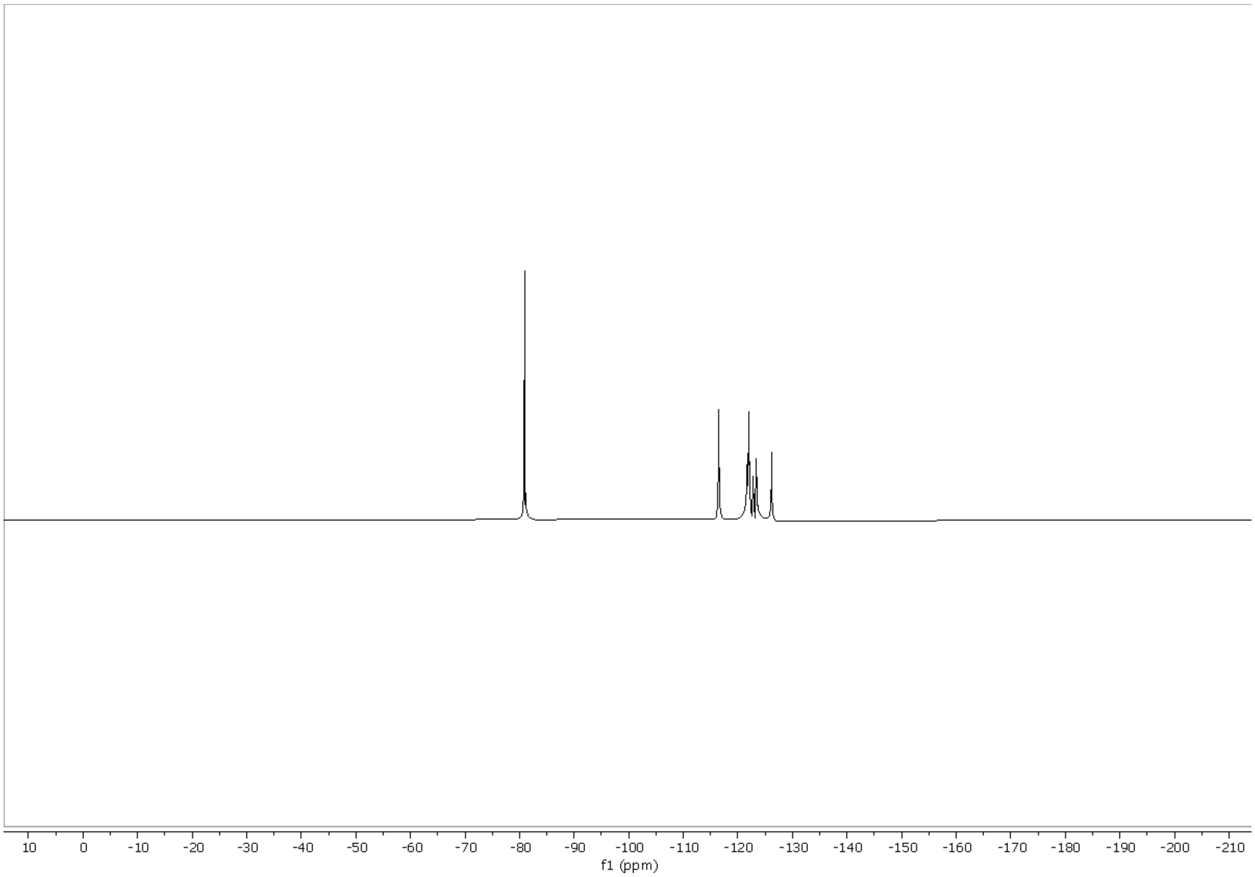

Figure S112. ^{19}F NMR of **LinF7**.

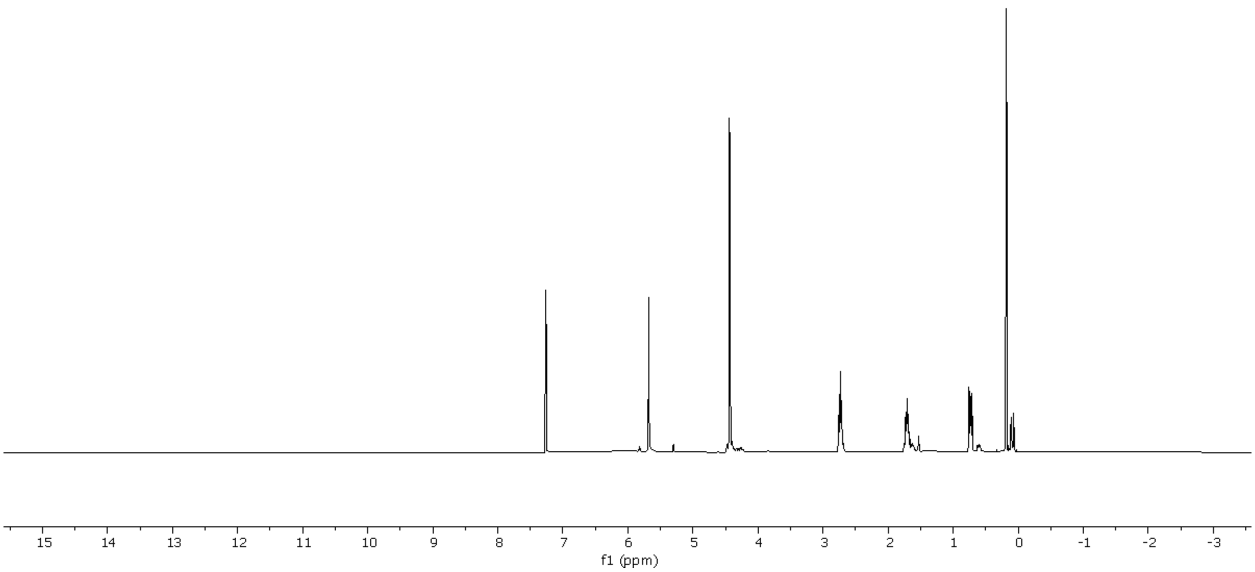

Figure S113. ^{1}H NMR of **PFP7**.

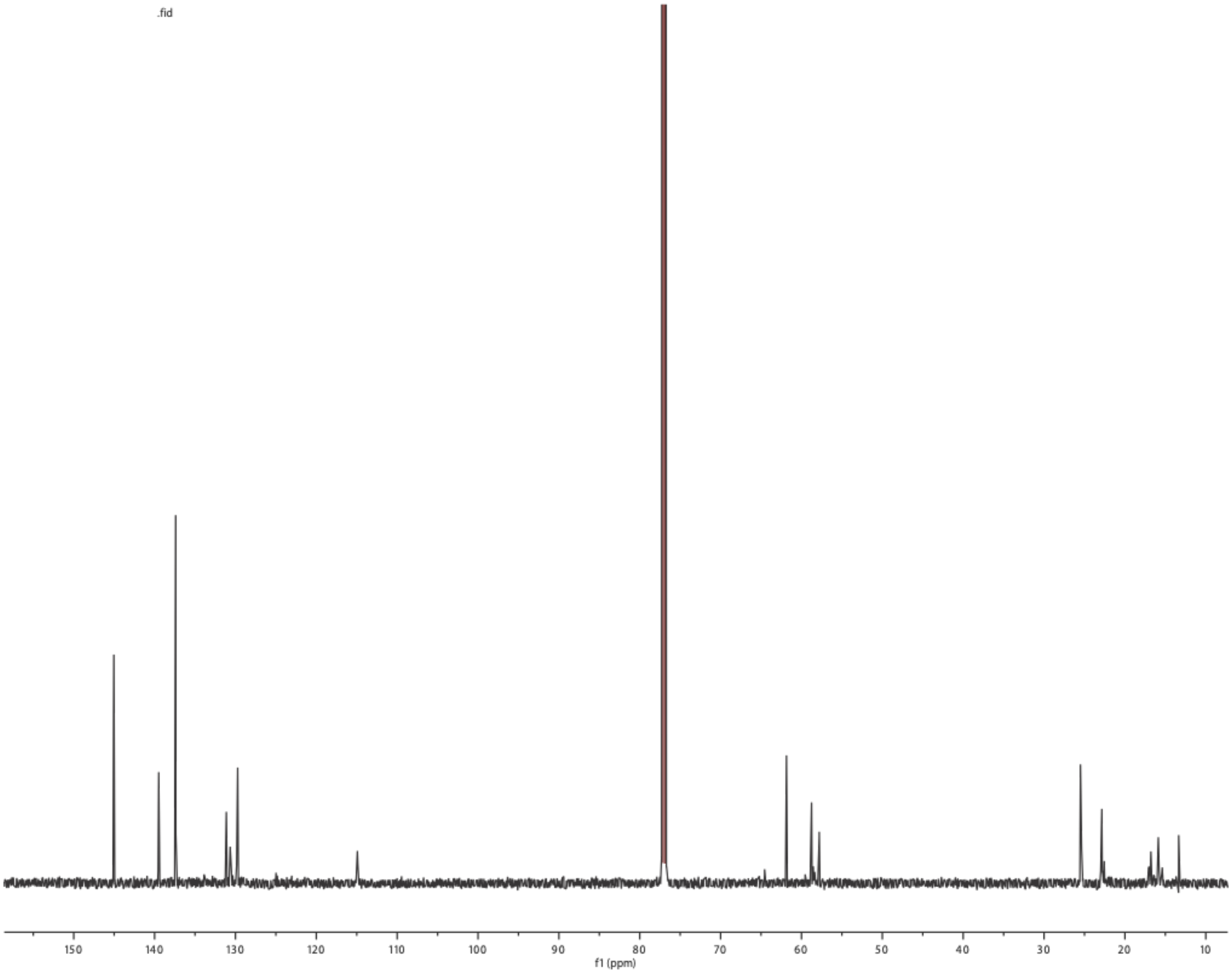

Figure S114. ^{13}C NMR of **PFP7**.

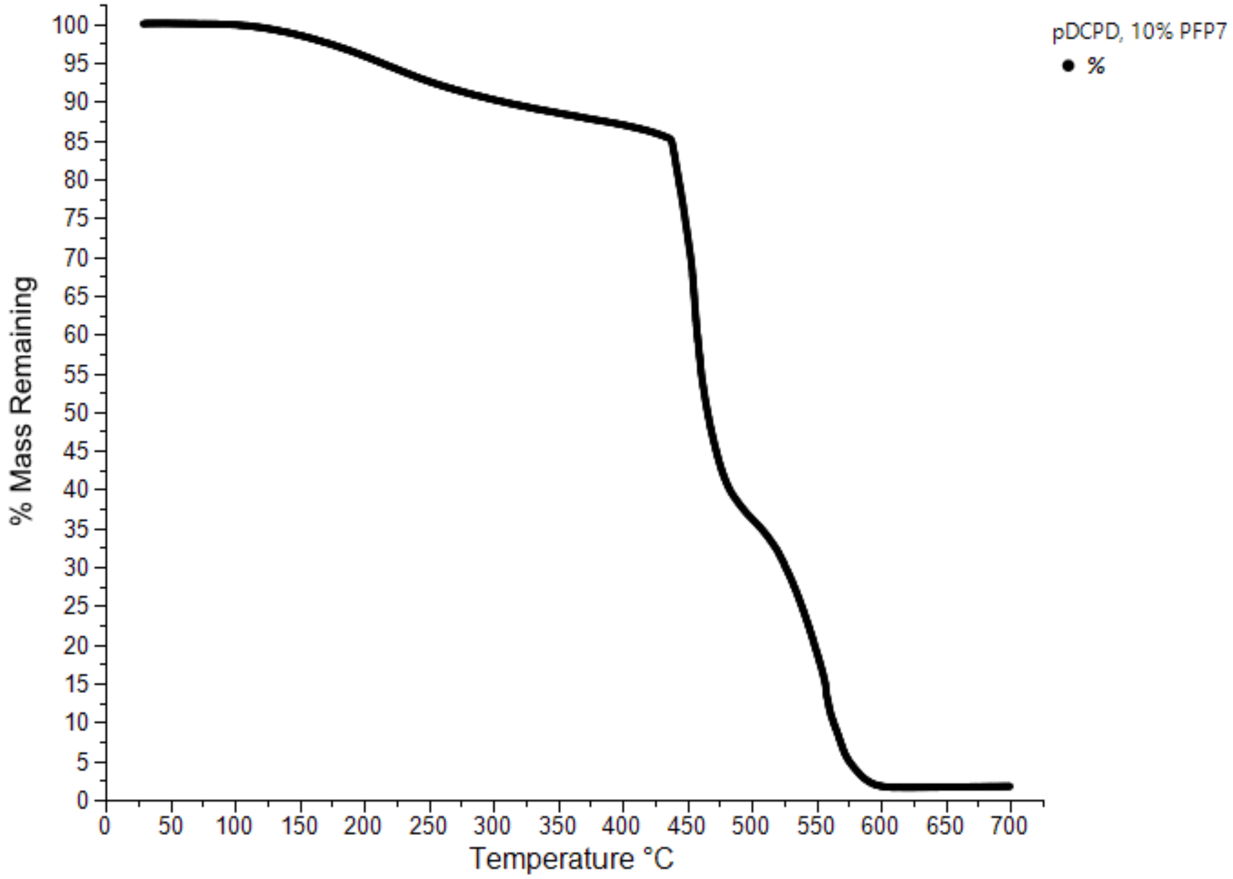

Figure S115. TGA, pDCPD with 10% v/v **PFP7**.

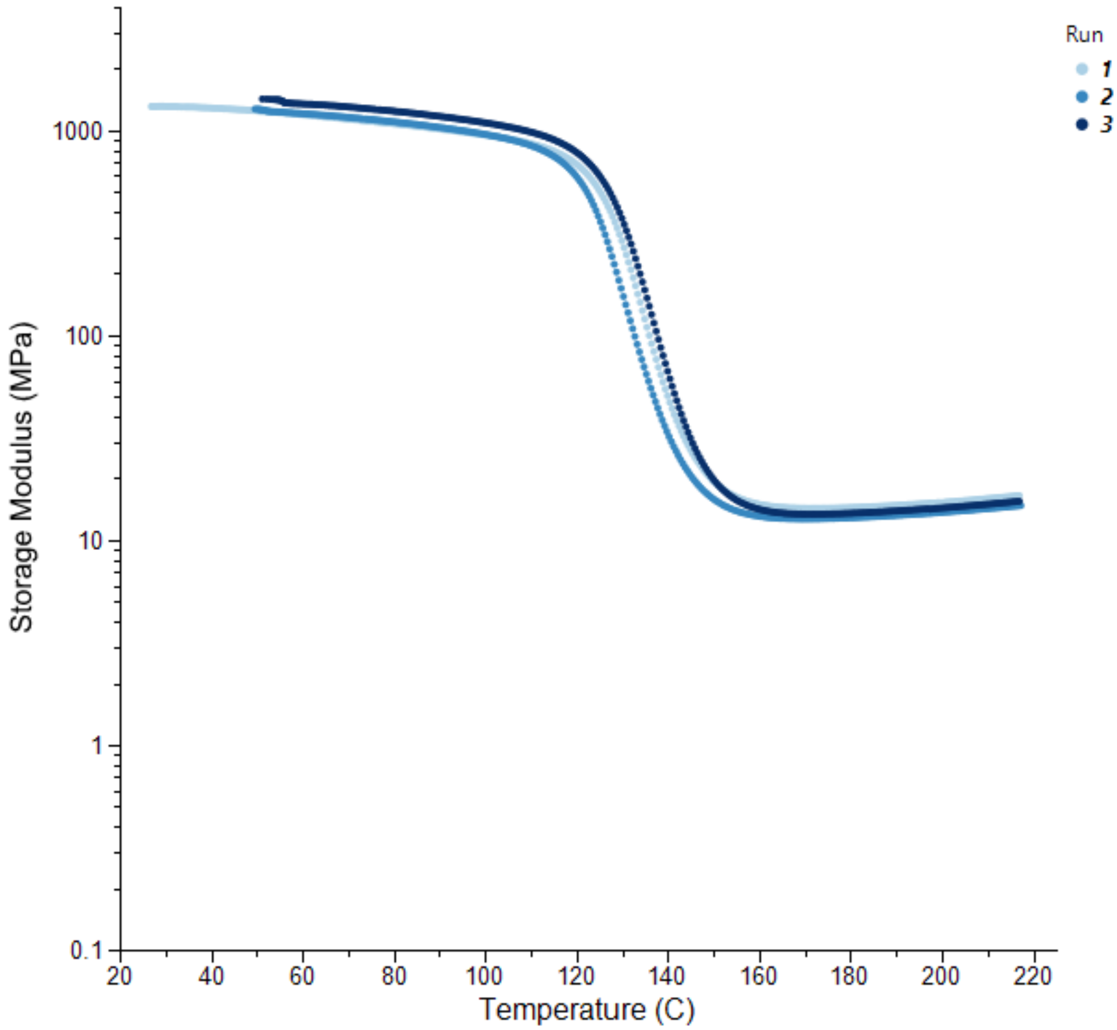

Figure S116. Storage modulus traces of pDCPD doped with 10 mol% **PhSi7** + 10 mol% **DDMS**.

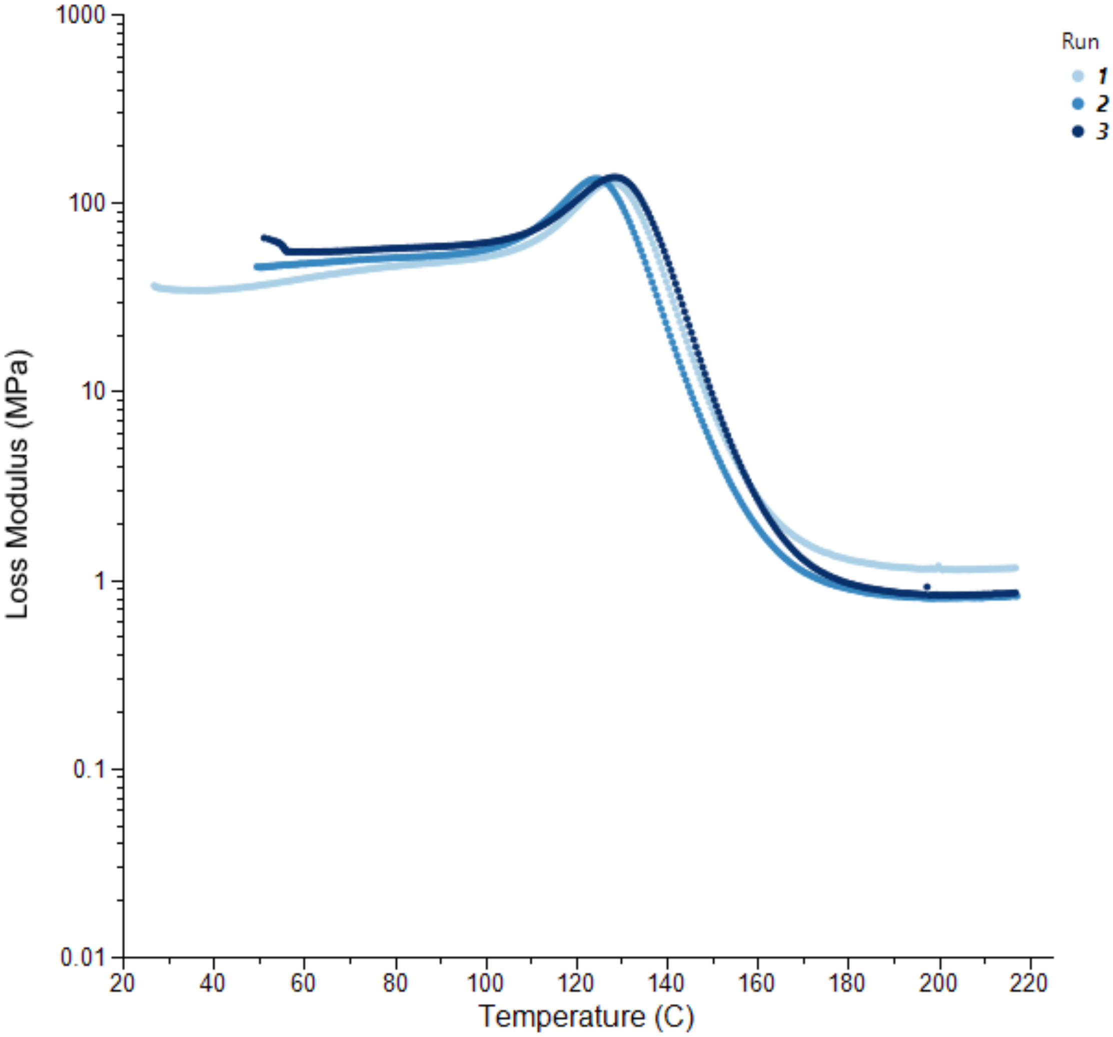

Figure S117. Loss modulus traces of pDCPD doped with 10 mol% **PhSi7** + 10 mol% **DDMS**.

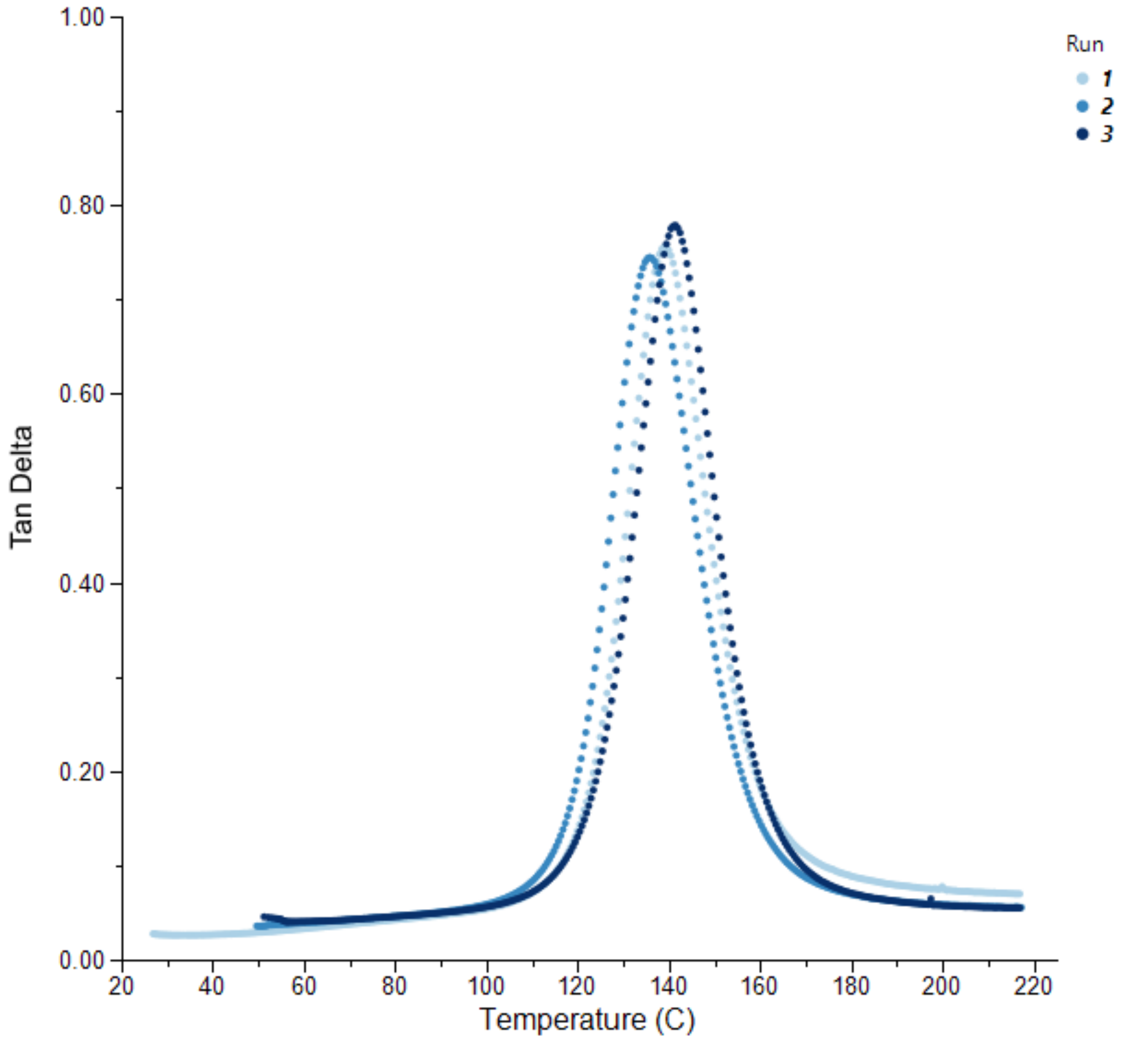

Figure S118. Tan delta traces of pDCPD doped with 10 mol% **PhSi7** + 10 mol% **DDMS**.

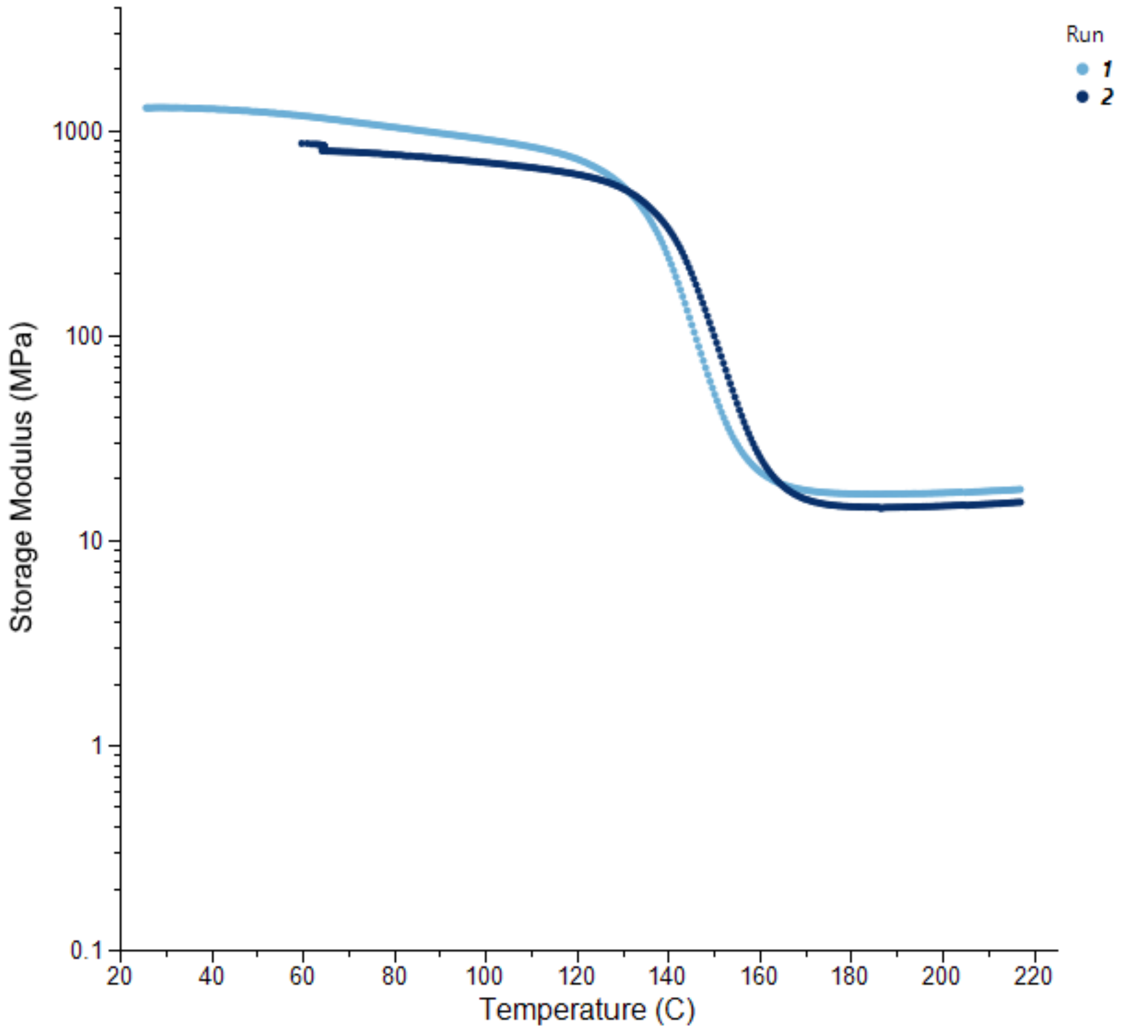

Figure S119. Storage modulus traces of pDCPD doped with 10 mol% **PhSi7** + 20 mol% **DDMS**.

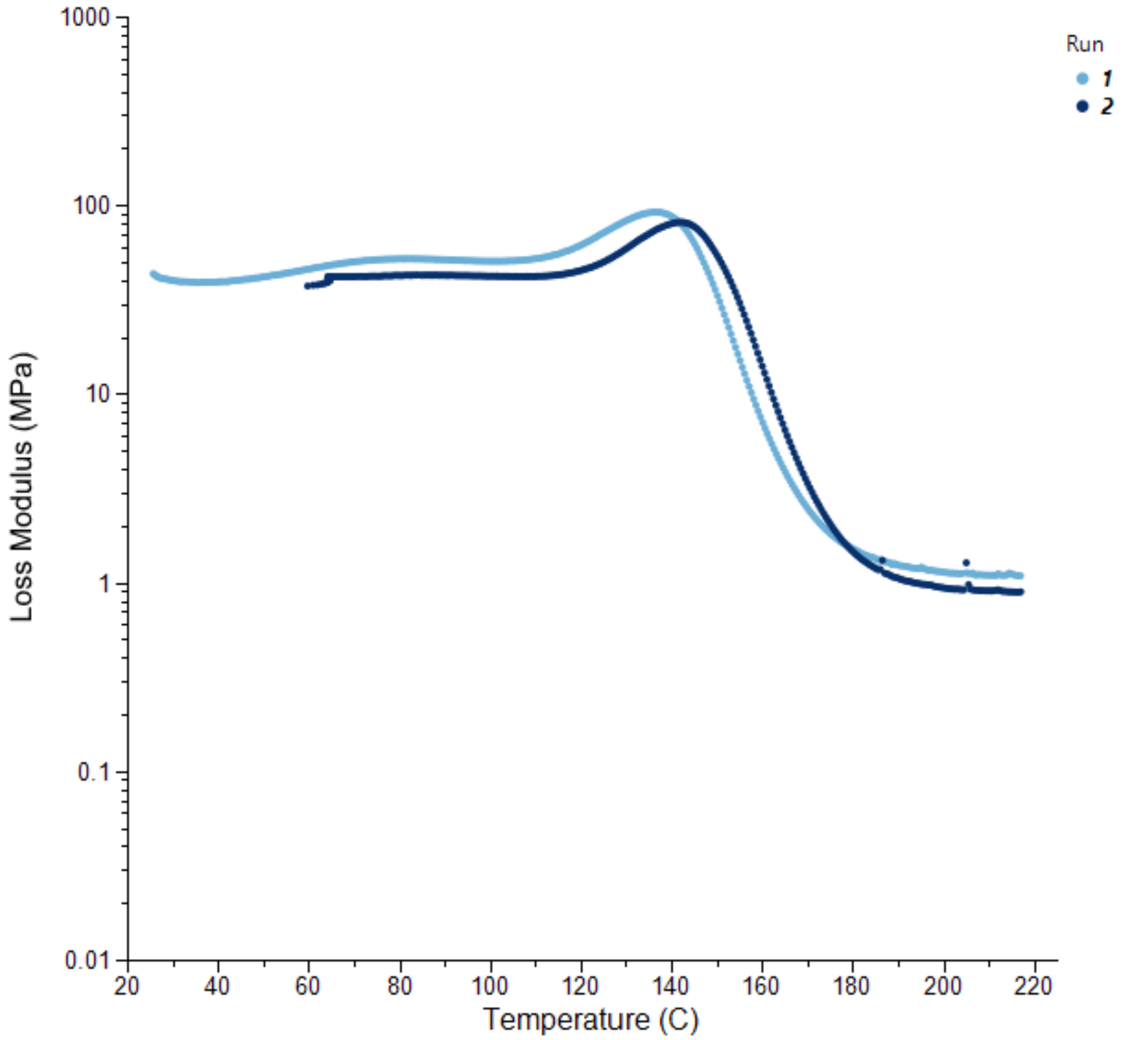

Figure S120. Loss modulus traces of pDCPD doped with 10 mol% **PhSi7** + 20 mol% **DDMS**.

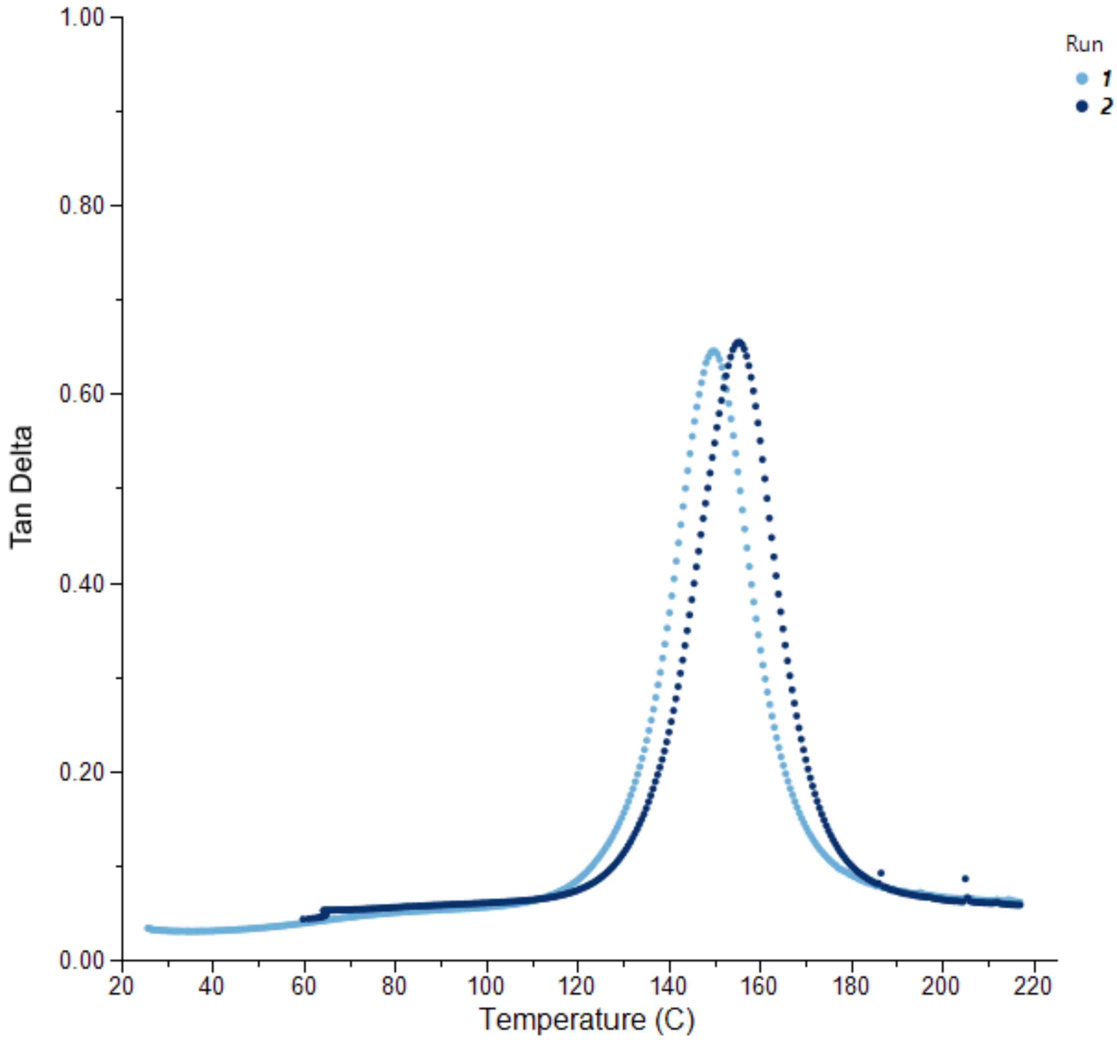

Figure S121. Tan delta traces of pDCPD doped with 10 mol% **PhSi7** + 20 mol% **DDMS**.

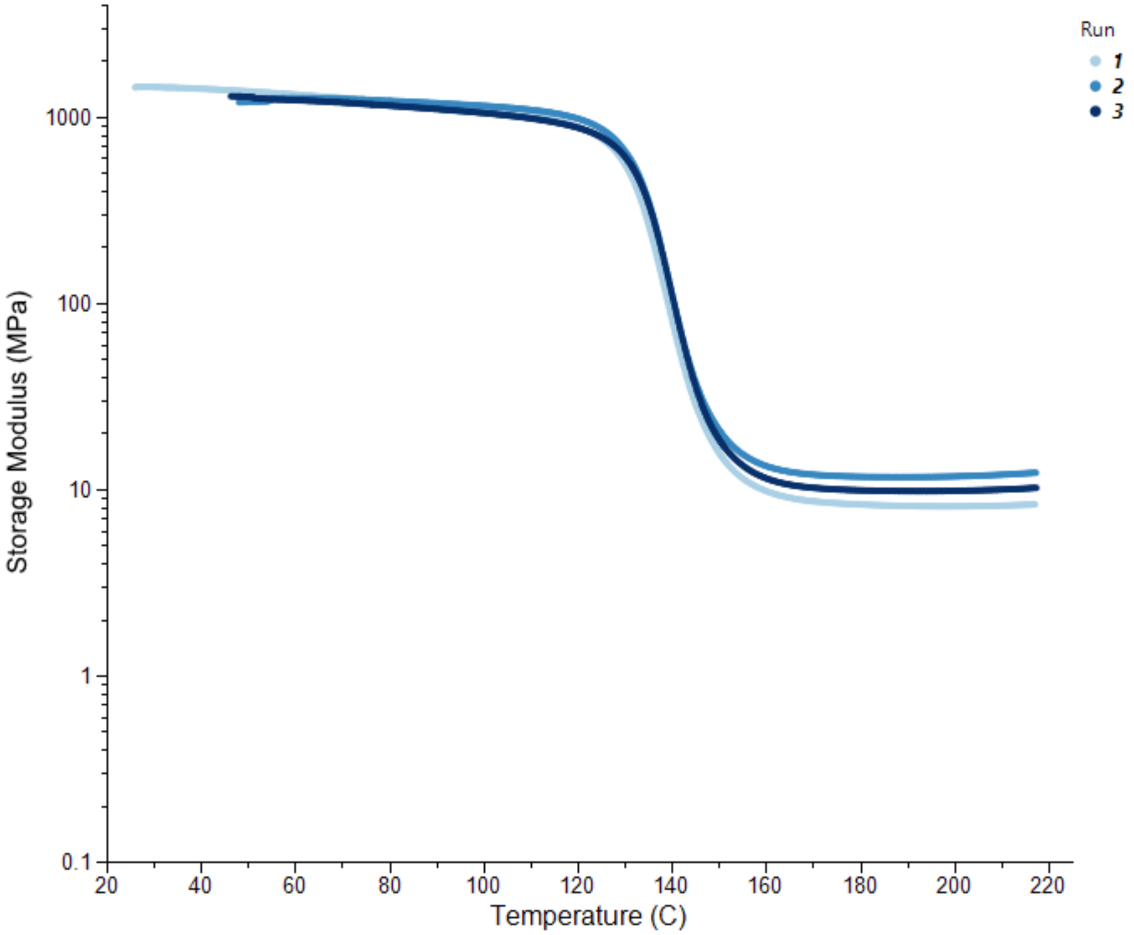

Figure S122. Storage modulus traces of pDCPD doped with 10 mol% **LinF7.**

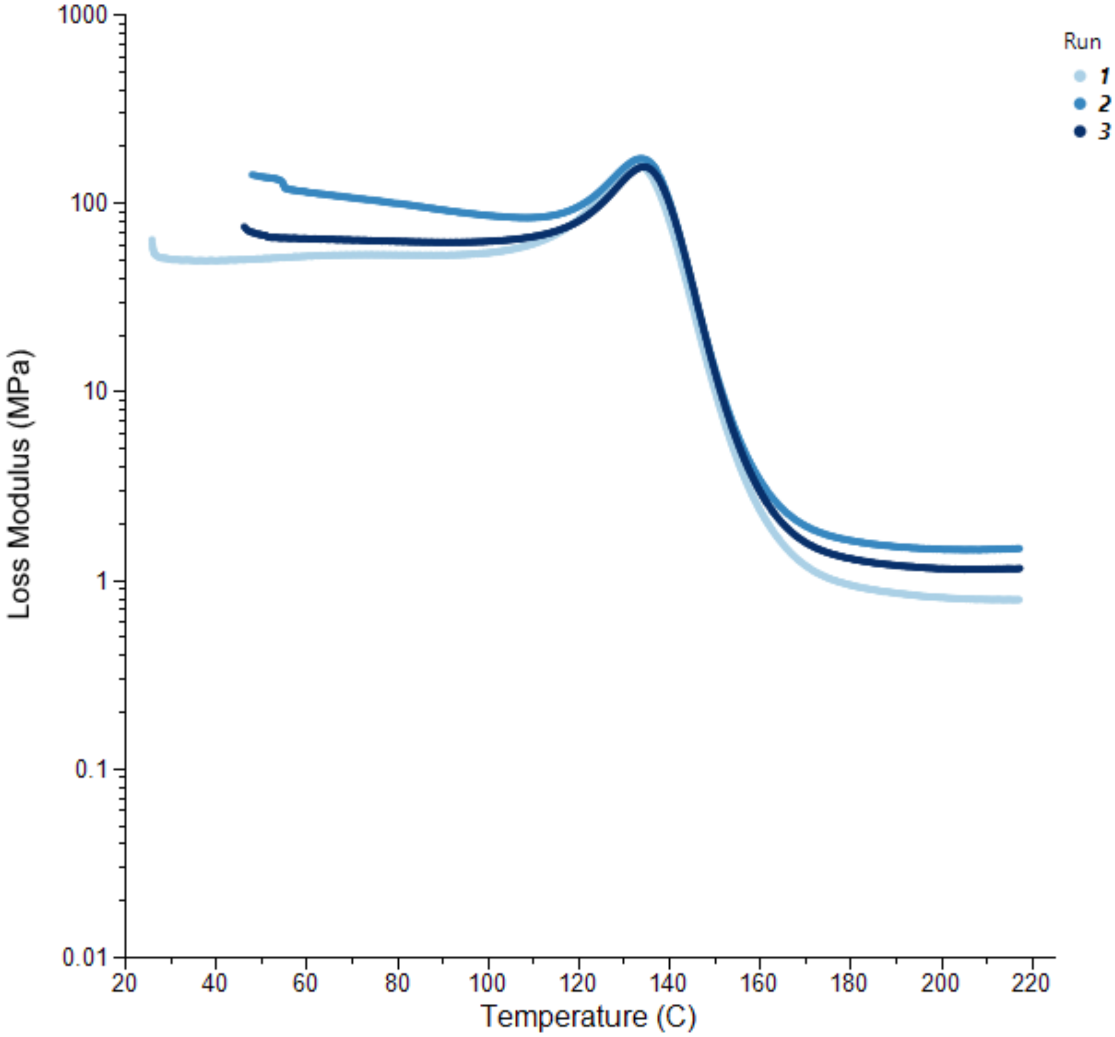

Figure S123. Loss modulus traces of pDCPD doped with 10 mol% **LinF7.**

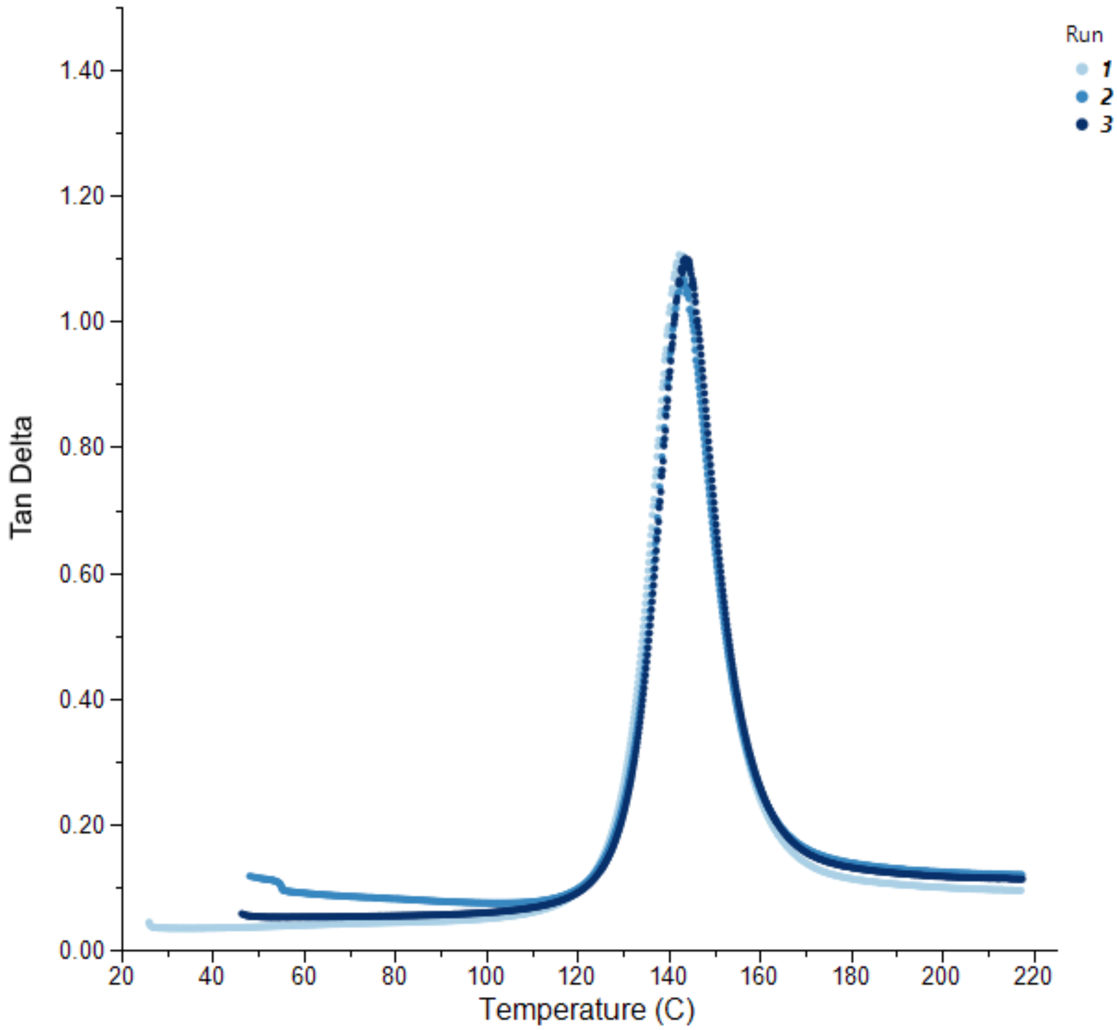

Figure S124. Tan delta traces of pDCPD doped with 10 mol% **LinF7**.

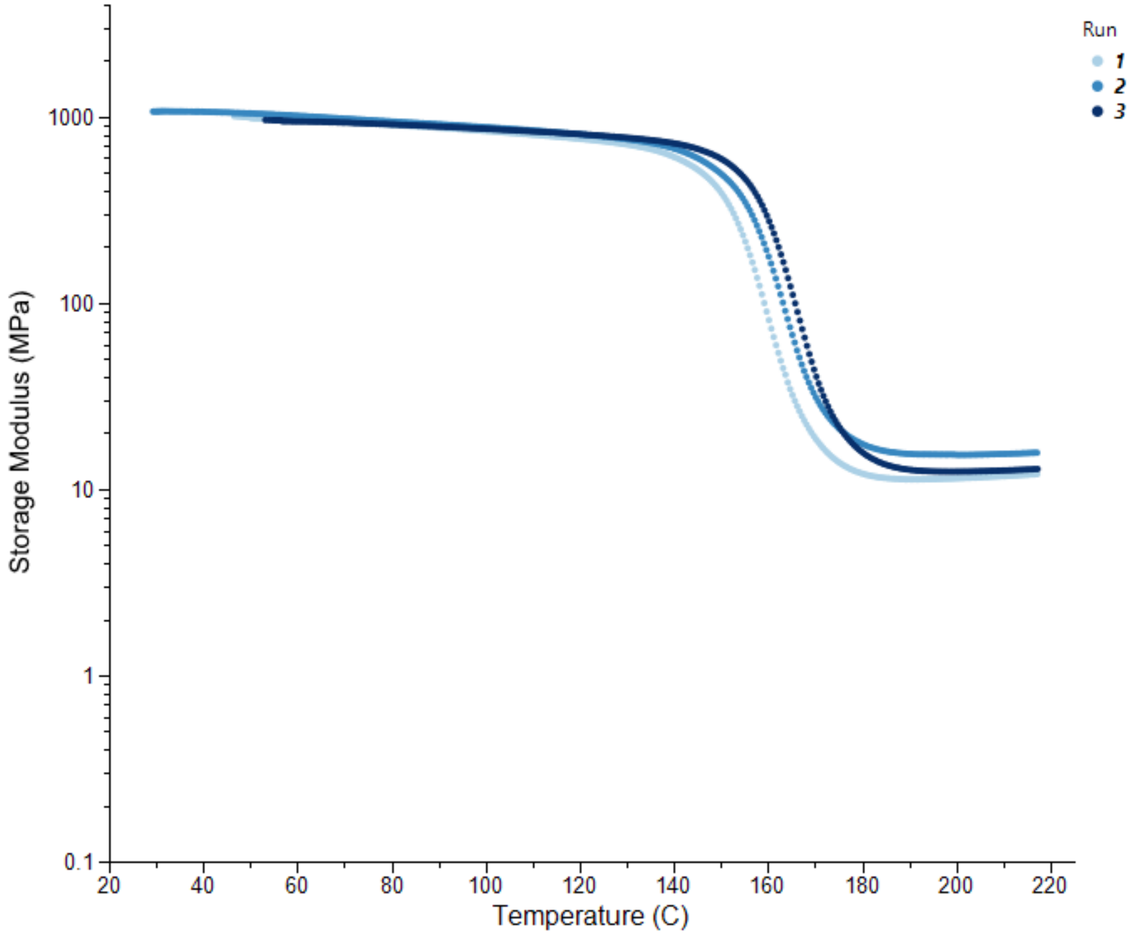

Figure S125. Storage modulus traces of pDCPD doped with 10 mol% **LinF7** and 10 mol% **DDMS**.

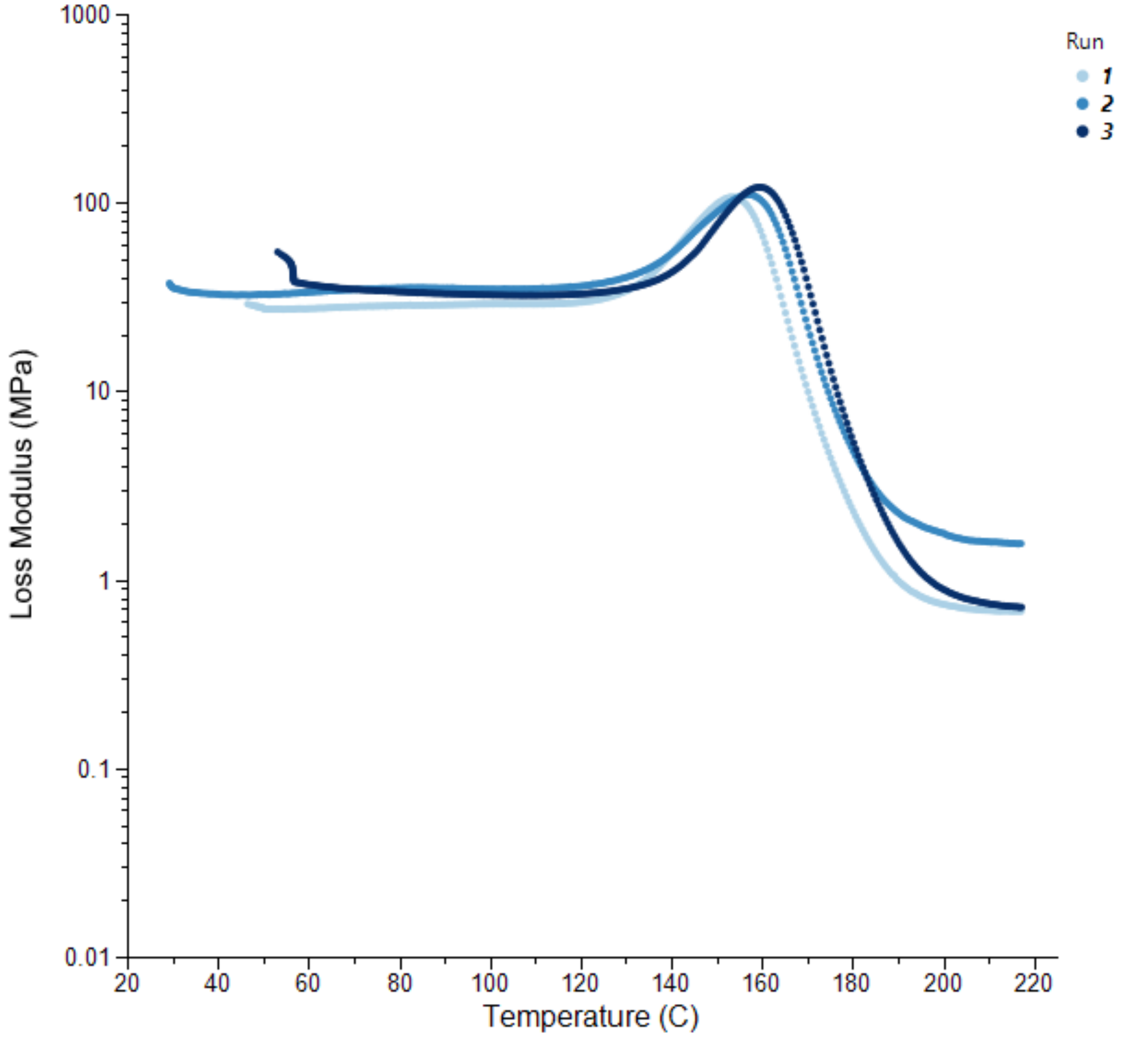

Figure S126. Loss modulus traces of pDCPD doped with 10 mol% **LinF7** and 10 mol% **DDMS**.

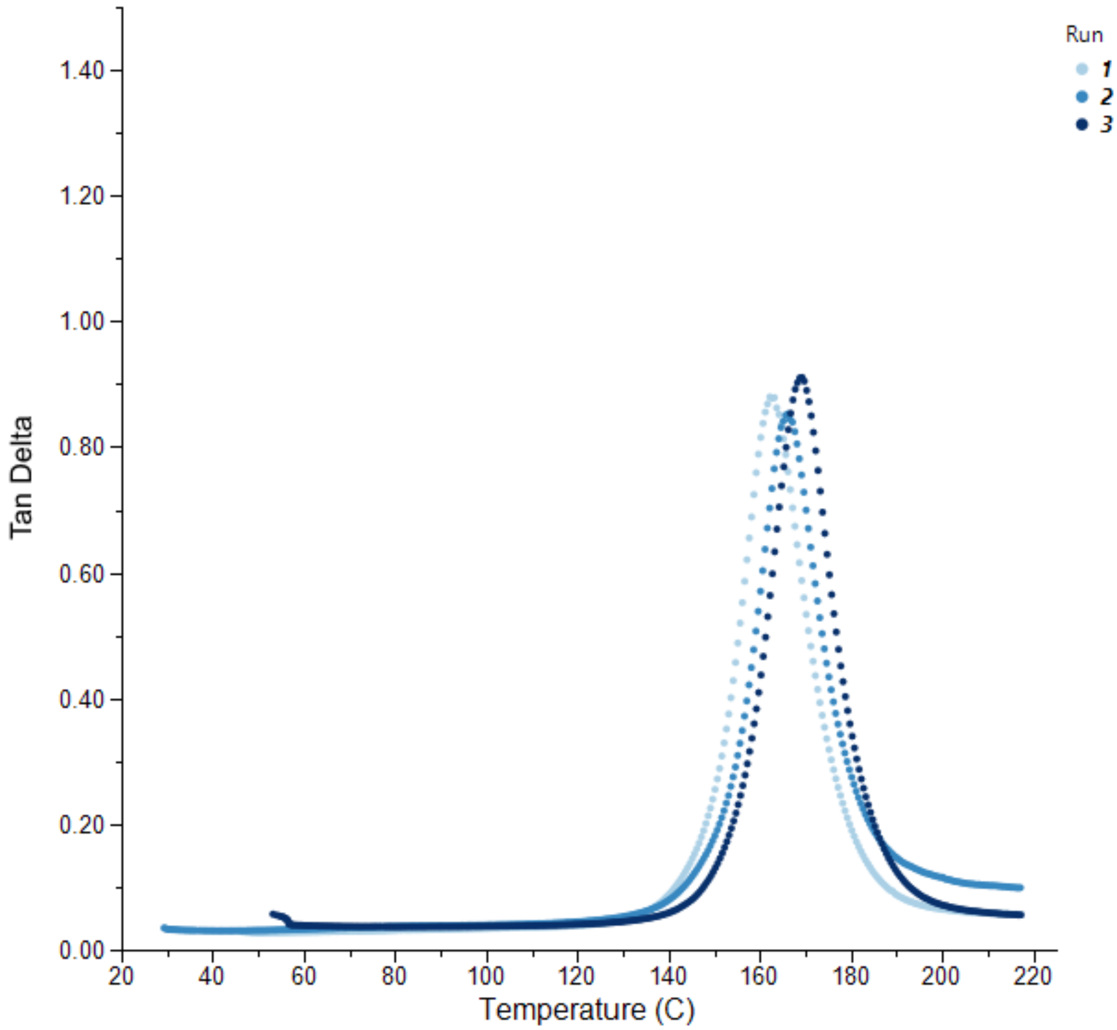

Figure S127. Loss modulus traces of pDCPD doped with 10 mol% **LinF7** and 20 mol% **DDMS.**

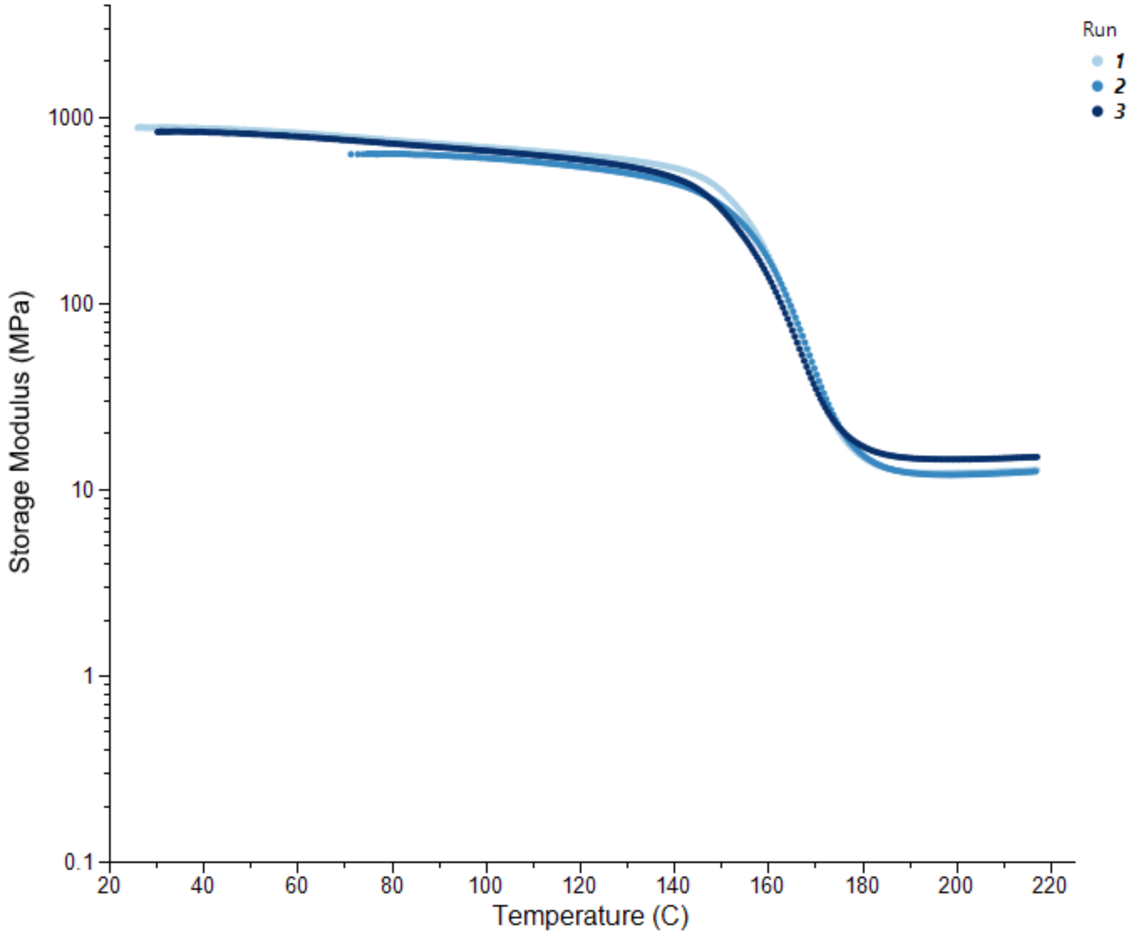

Figure S128. Storage modulus traces of pDCPD doped with 10 mol% **LinF7** and 20 mol% **DDMS**.

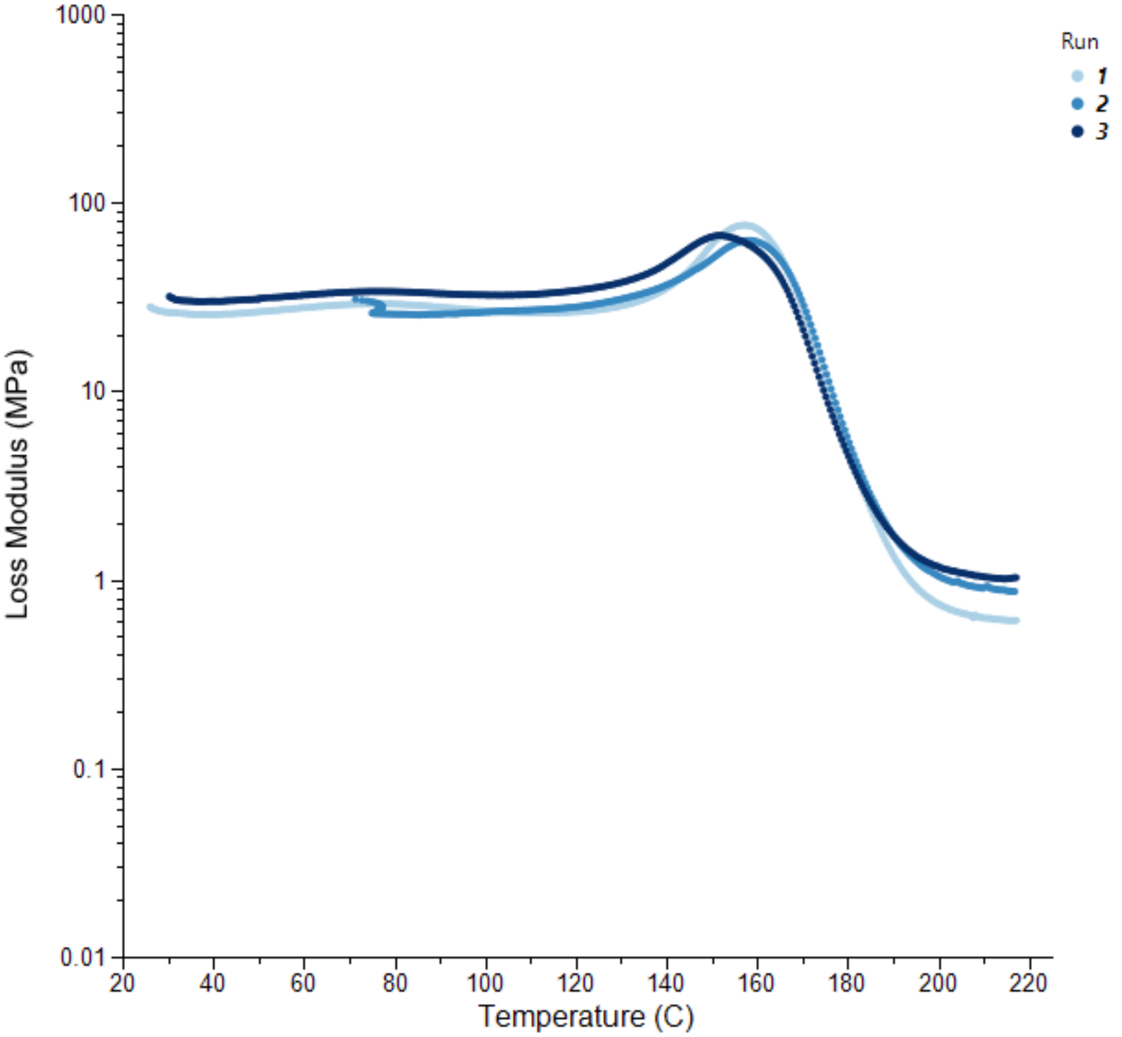

Figure S129. Loss modulus traces of pDCPD doped with 10 mol% **LinF7** and 20 mol% **DDMS**.

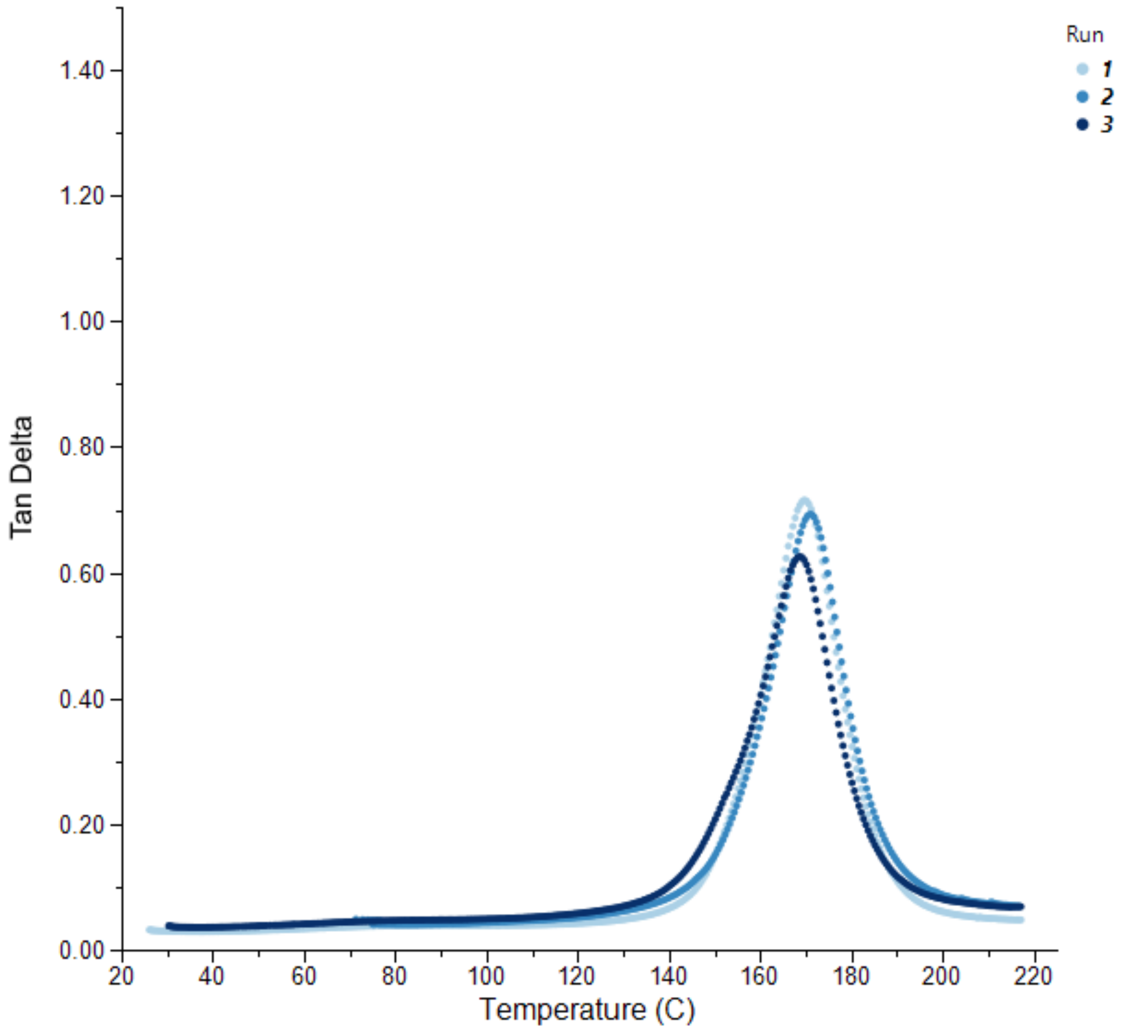

Figure S130. Tan delta traces of pDCPD doped with 10 mol% **LinF7** and 20 mol% **DDMS**.

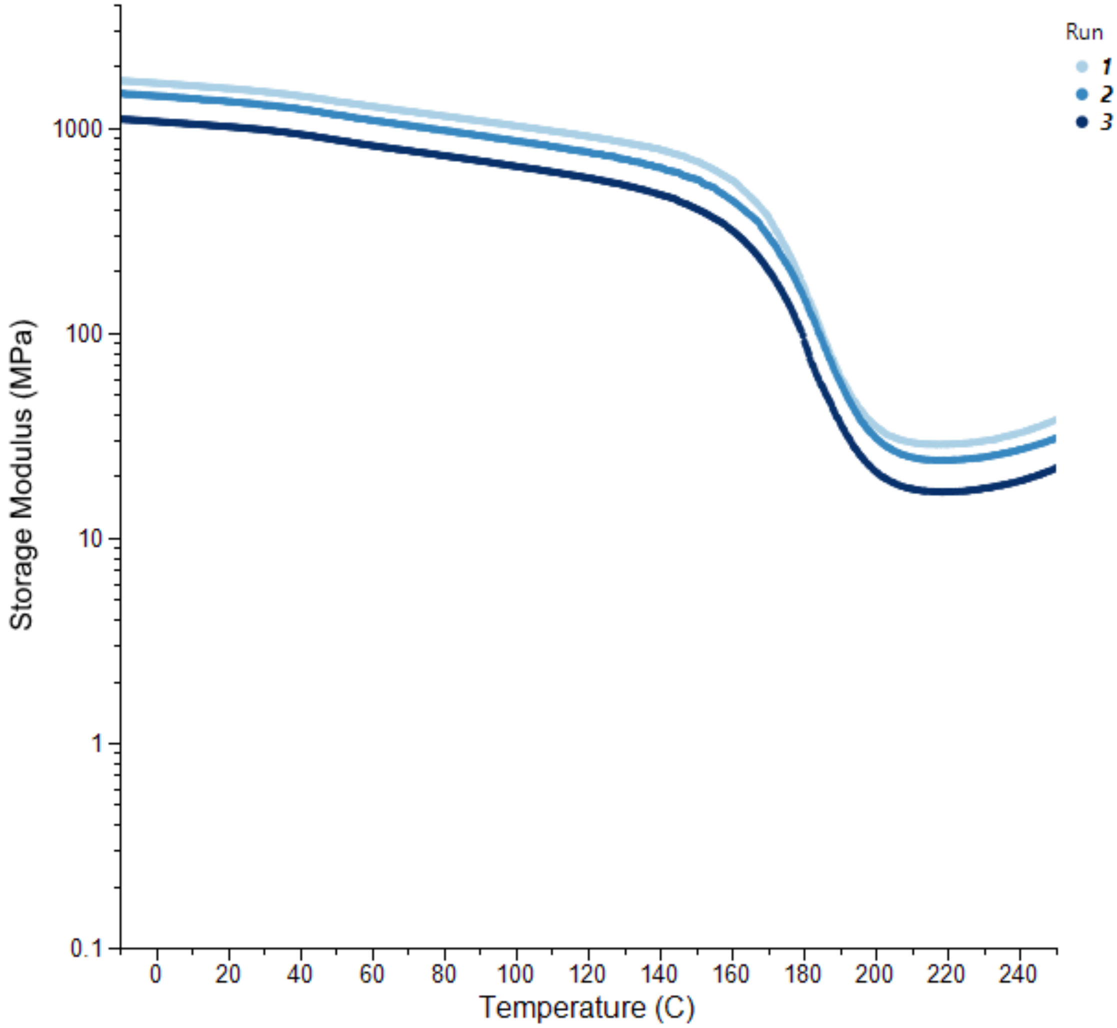

Figure S131. Storage modulus traces of pDCPD doped with 5 mol% **iPrSi8**, 20 mol% **DDMS**.

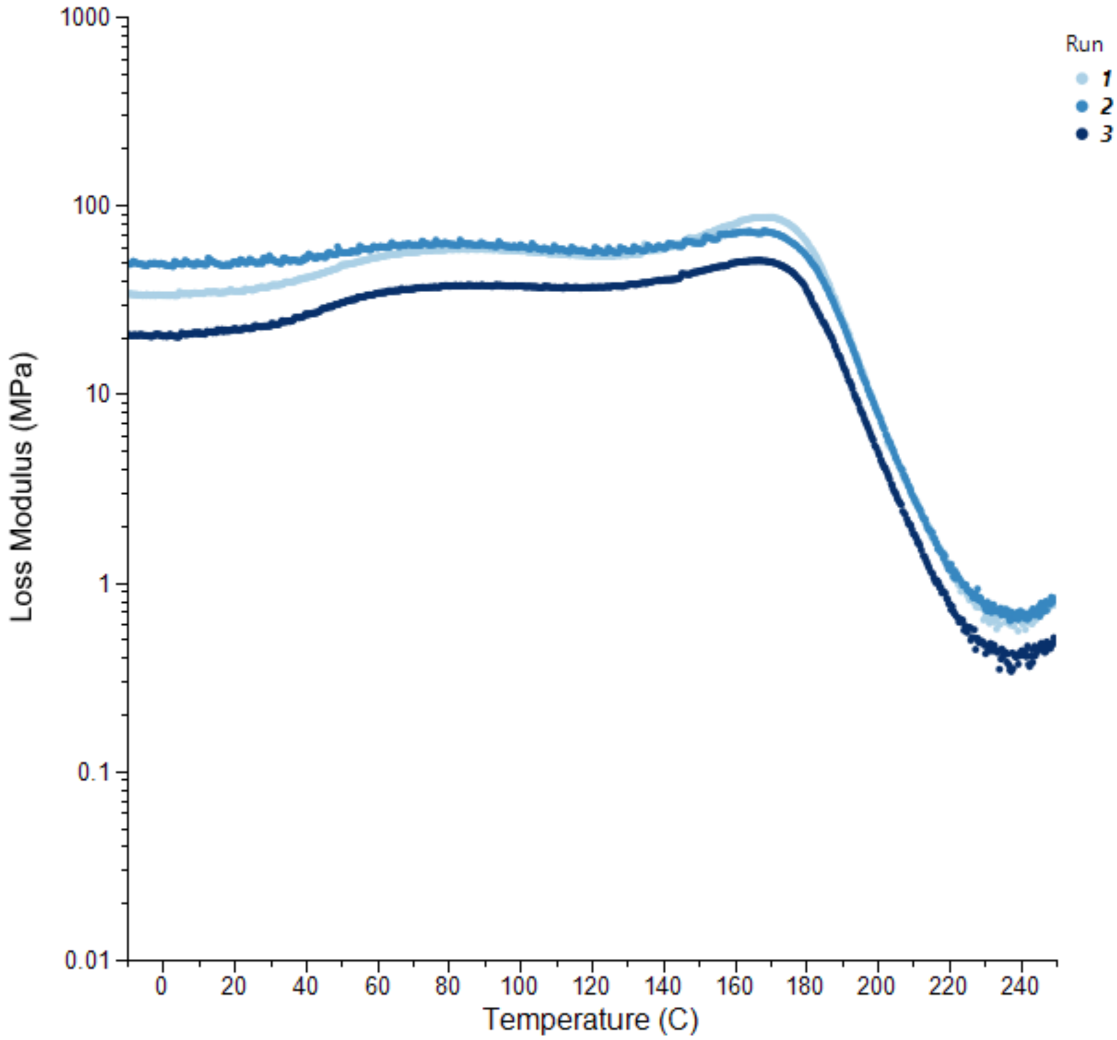

Figure S132. Loss modulus traces of pDCPD doped with 5 mol% **iPrSi8**, 20 mol% **DDMS**.

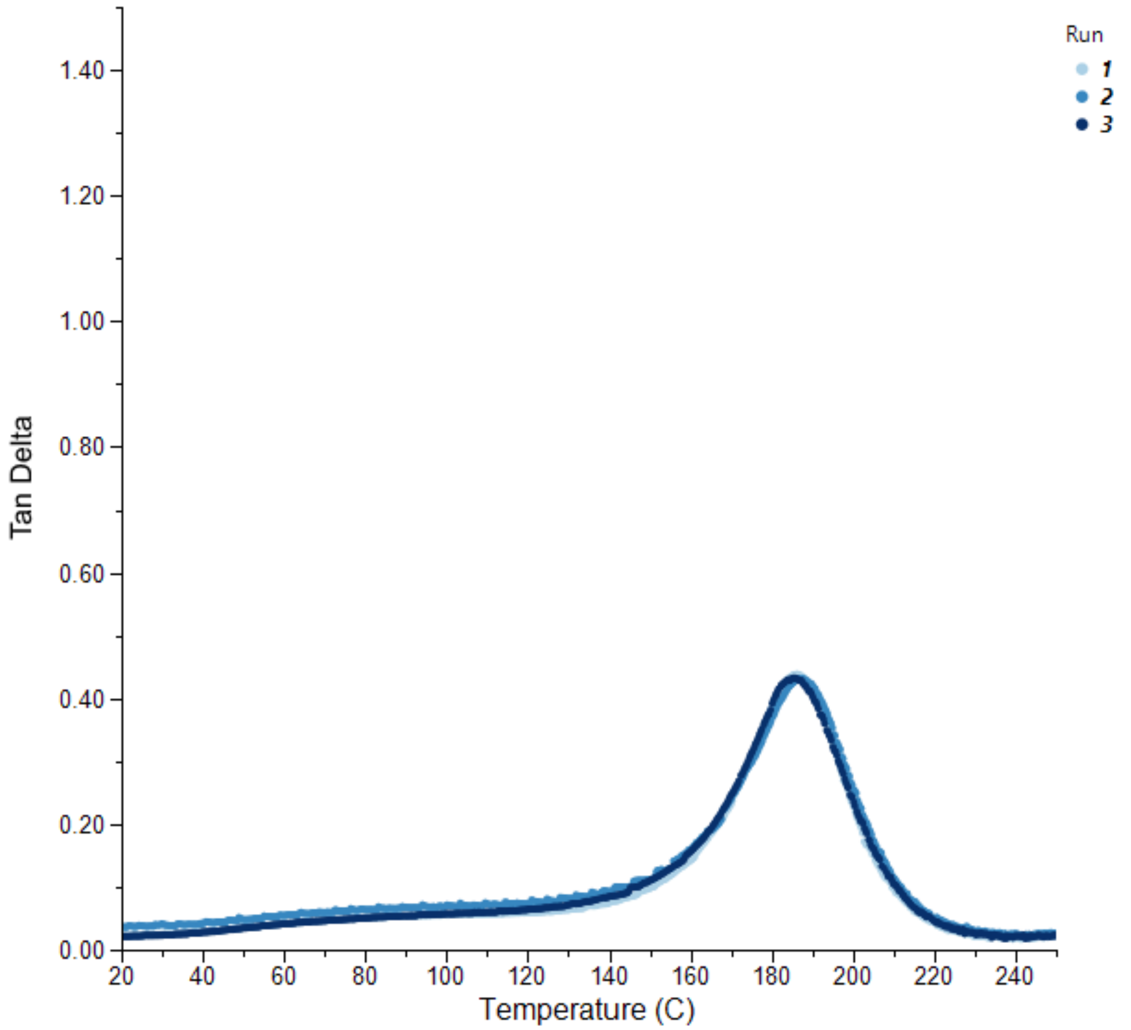

Figure S133. Tan delta traces of pDCPD doped with 5 mol% **iPrSi8**, 20 mol% **DDMS**.

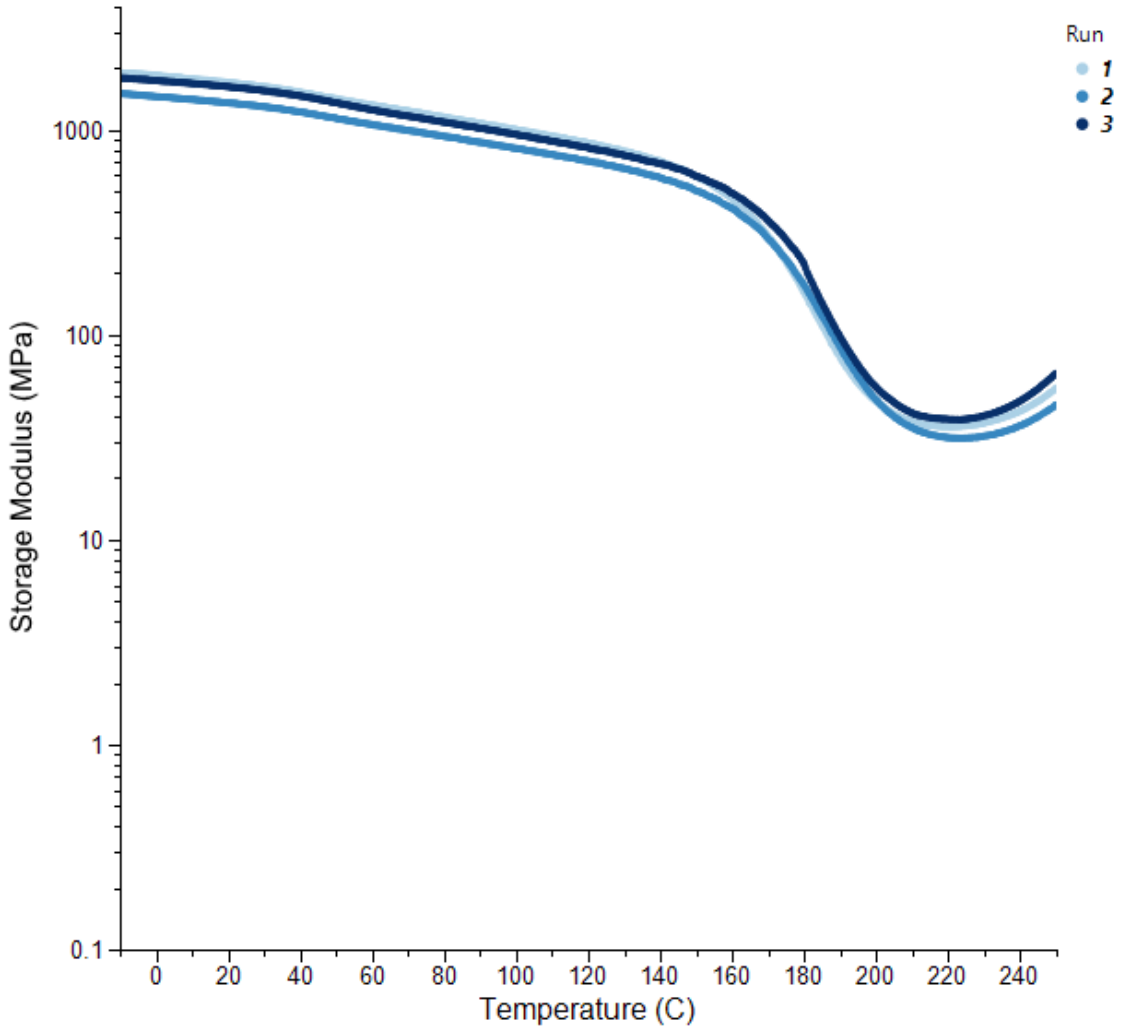

Figure S134. Storage modulus traces of pDCPD doped with 5 mol% **iPrSi8**, 25 mol% **DDMS**.

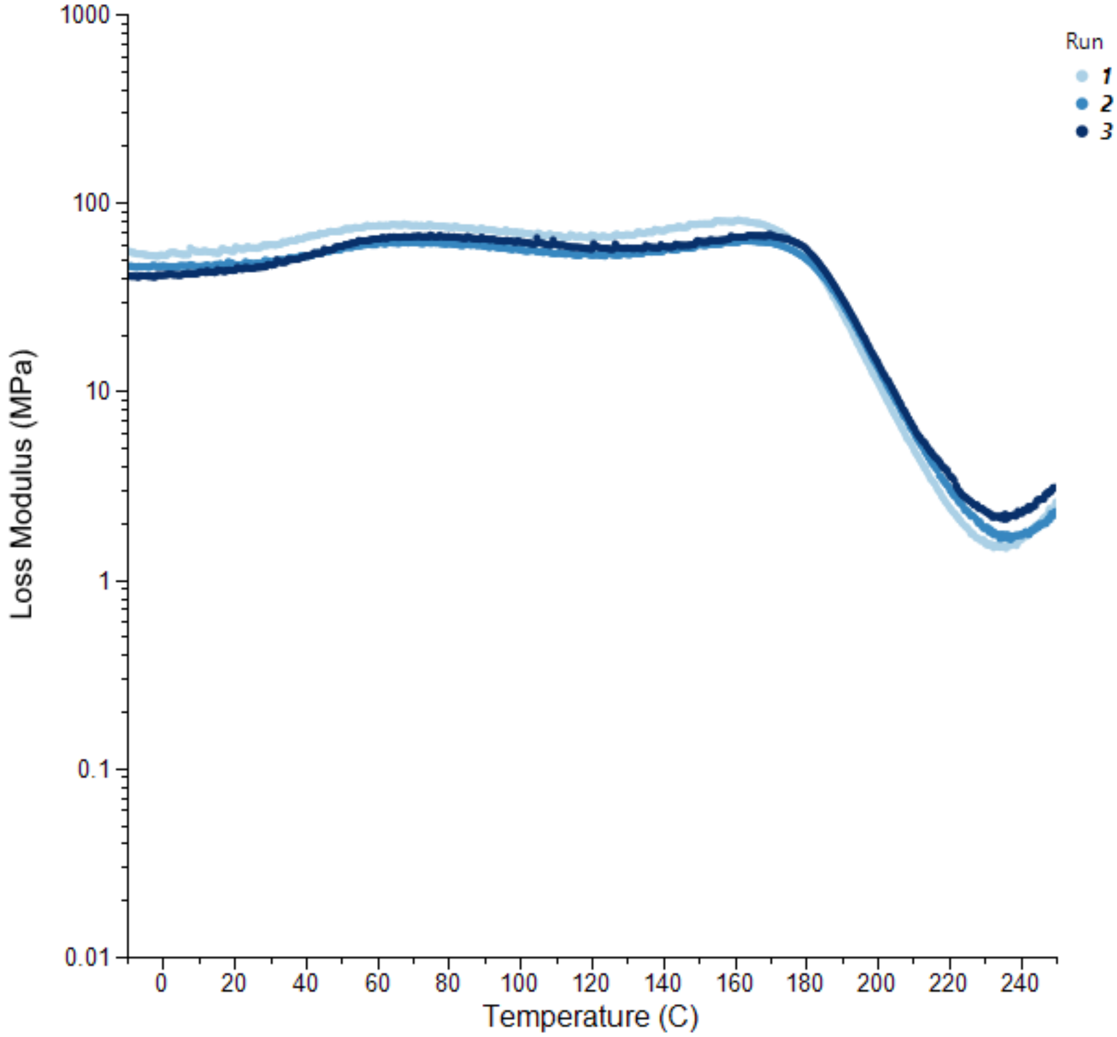

Figure S135. Loss modulus traces of pDCPD doped with 5 mol% **iPrSi8**, 25 mol% **DDMS**.

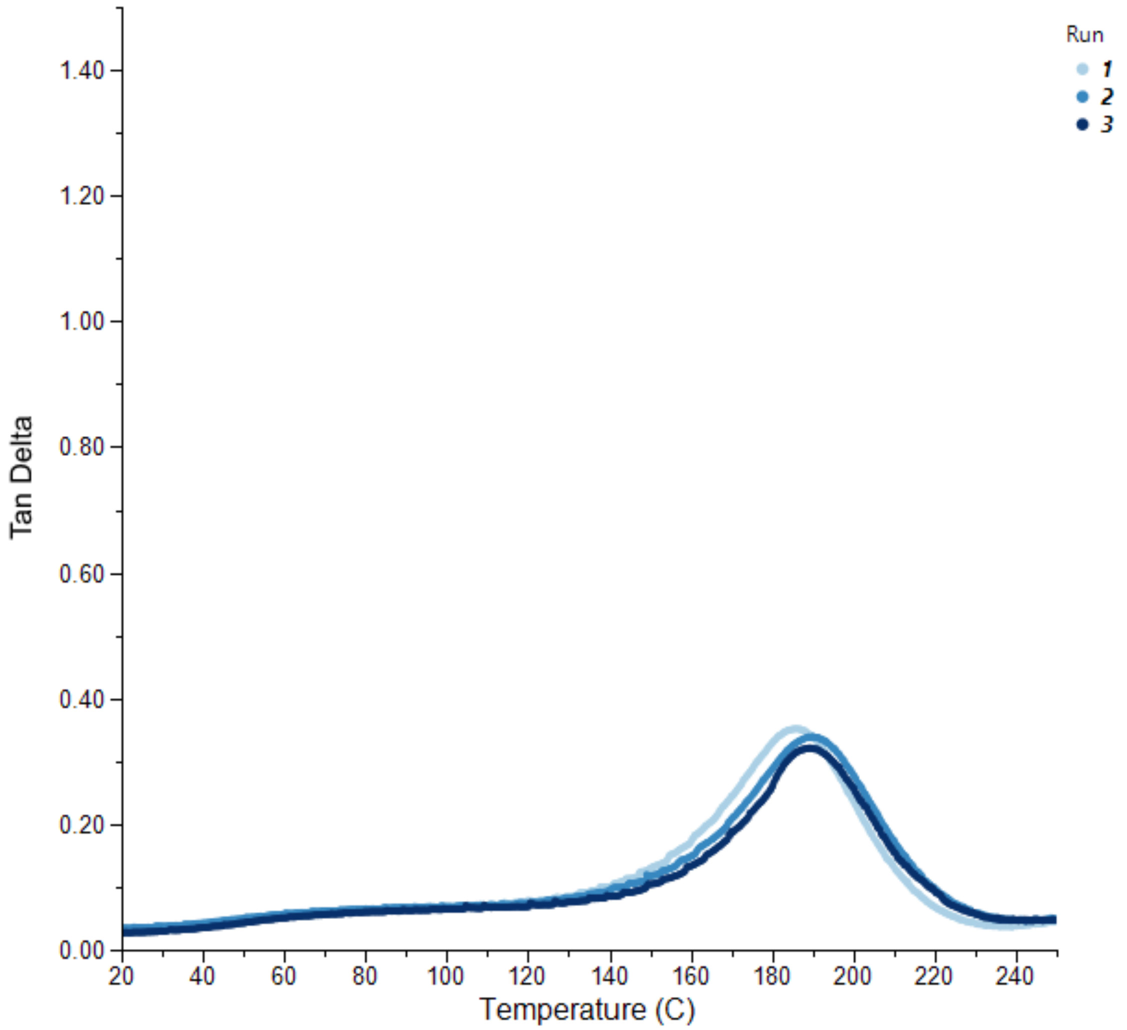

Figure S136. Tan delta traces of pDCPD doped with 5 mol% **iPrSi8**, 25 mol% **DDMS**.

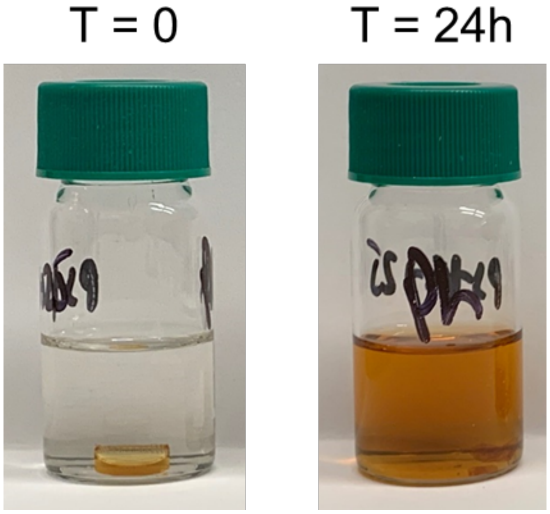

Figure S137. Images of dissolution of pDCPD samples doped with 10 mol% **PhSi7** and 20 mol% **DDMS** in 0.2 M TBAF in THF.

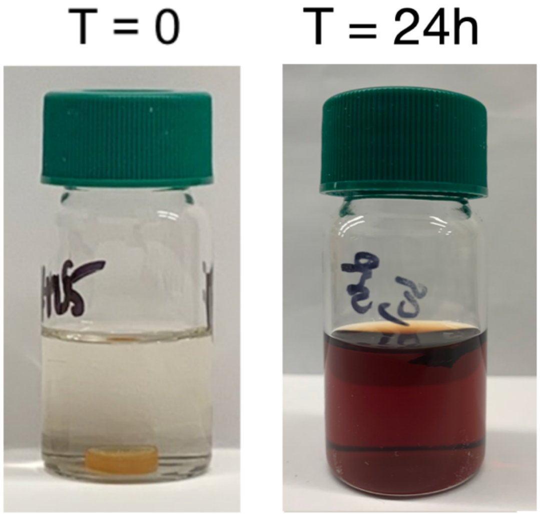

Figure S138. Images of pDCPD samples doped with 10 mol% **LinF7** and 20 mol% **DDMS** in 0.2 M TBAF in THF.

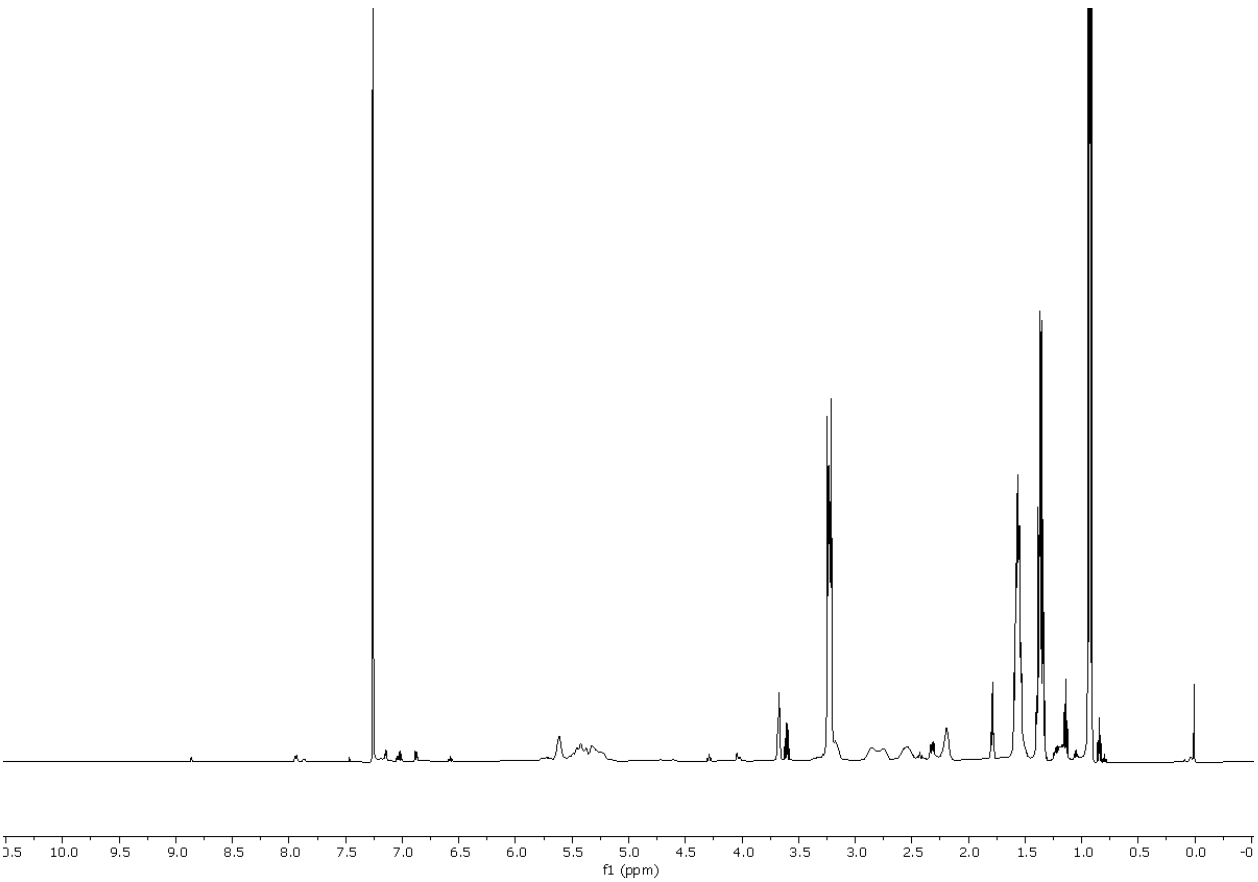

Figure S139. ^{1}H NMR of fragments from dissolution of pDCPD samples doped with 10 mol% **PhSi7** and 20 mol% **DDMS.**

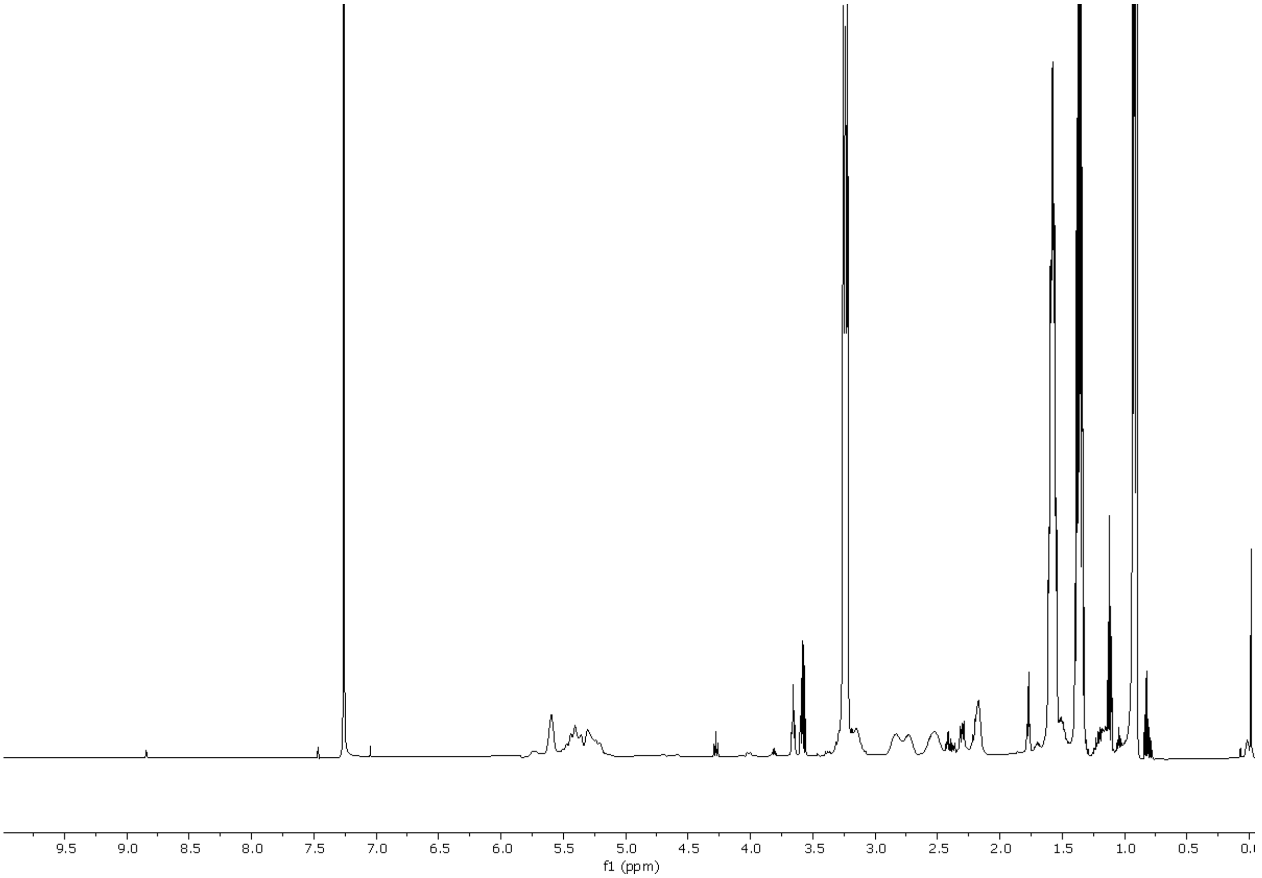

Figure S140. ^{1}H NMR of fragments from dissolution of pDCPD samples doped with 10 mol% **LinFSi7** and 20 mol% **DDMS.**

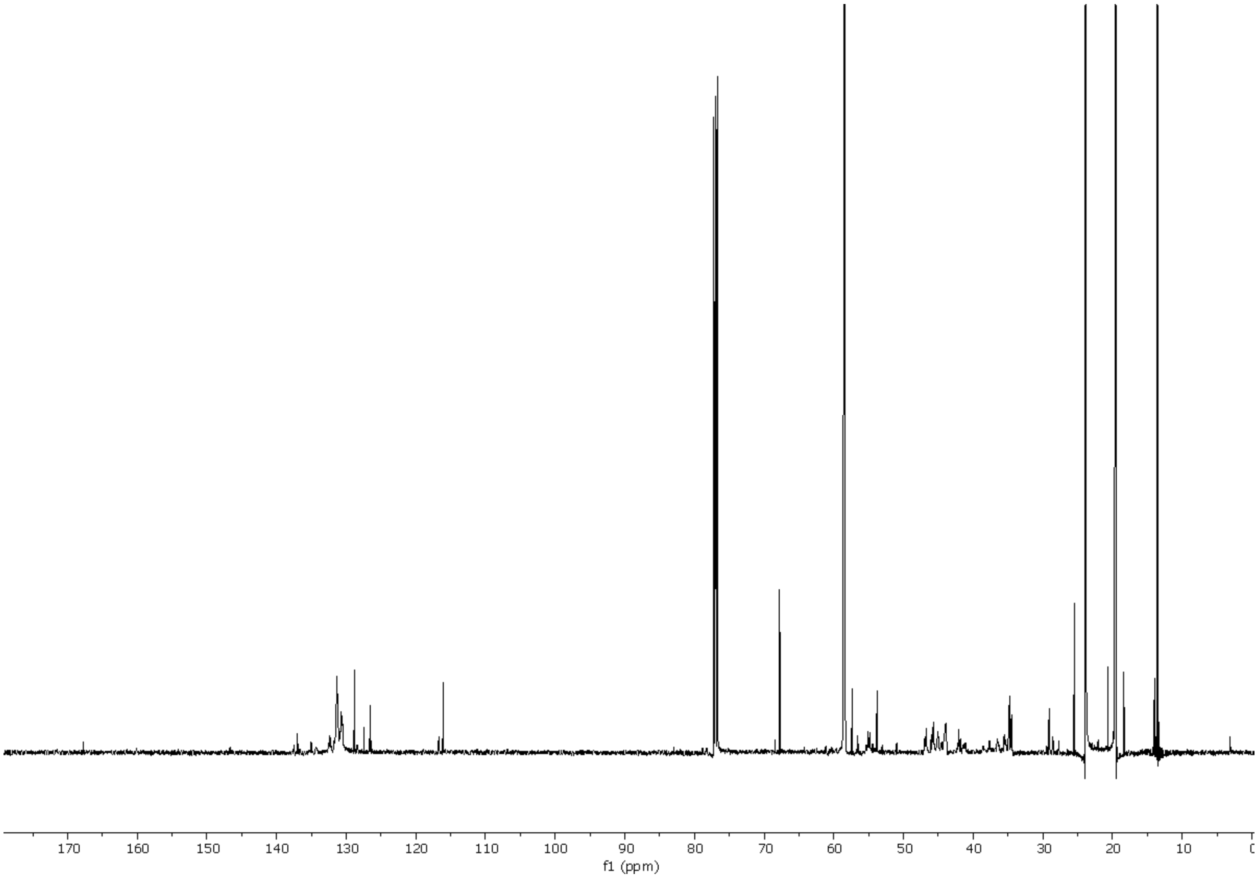

Figure S141. ^{13}C NMR of fragments from pDCPD samples doped with 10 mol% **PhSi7** and 20 mol% **DDMS.**

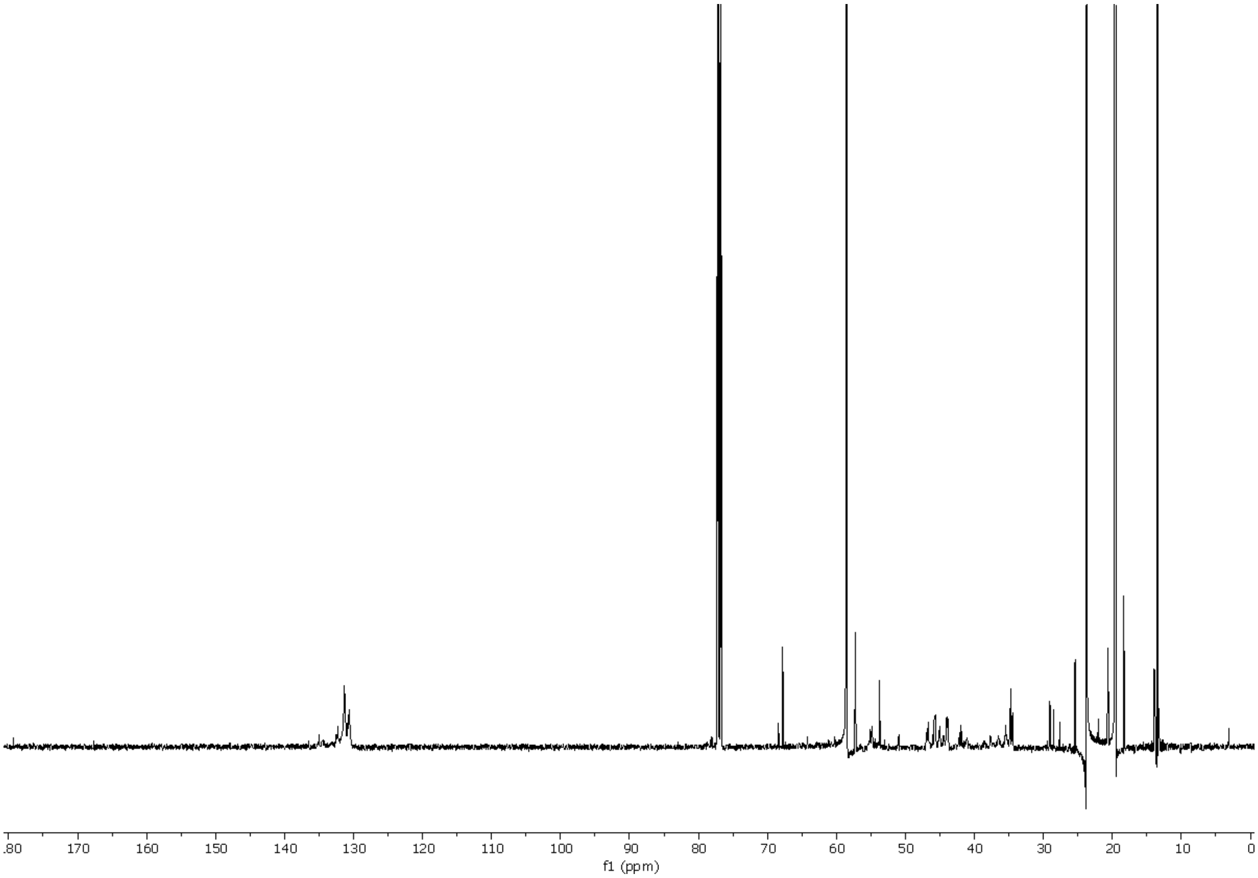

Figure S142. ^{13}C NMR of fragments from dissolution of pDCPD samples doped with 10 mol% **LinFSi7** and 20 mol% **DDMS.**

Tables

Table S1: Literature values used in model training.

M[a]	M%	CoM	CoM %	CL	CL %	C	M:C	Tg (˚C)	σ_Y (MPa)	E (GPa)	Method
DCPD [10]	100					G1	100000	118			DMA
DCPD [10]	100					G1	5000	145	54.8	1.851	DMA
DCPD [10]	100					G1	3000	134			DMA
DCPD [10]	75	*ENB*	25			G1	5000	132	52.7	1.931	DMA
DCPD [10]	50	*ENB*	50			G1	5000	119	52.6	2.004	DMA
DCPD [10]	25	*ENB*	75			G1	5000	108	50.6	1.972	DMA
DCPD [10]	10	*ENB*	90			G1	5000	102	49.5	1.974	DMA
DCPD [10]	95			*HDMN*	5	G1	3000	144			DMA
DCPD [10]	90			*HDMN*	10	G1	3000	153	54.2	1.871	DMA
DCPD [10]	80			*HDMN*	20	G1	3000	170	56.5	1.985	DMA
DCPD [10]	70			*HDMN*	30	G1	3000	181	58.1	2.044	DMA
DCPD [10]	60			*HDMN*	40	G1	3000	187		2.123	DMA
DCPD [11]	100					G2	5000	163.3			DMA
DCPD [12]	100					G2	10000	143	52	1.8	DMA
DCPD [12]	85	*NBOH*	15			G2	10000	142	55	1.9	DMA
DCPD [12]	50	*NBOH*	50			G2	10000	143	56	1.9	DMA
DCPD [13]	100					G1	15000	129	60.5	0.93	DMA
DCPD [13]	98.46	*1NB_POSS*	1.54			G1	15000	128	55.1	0.87	DMA
DCPD [13]	96.6	*1NB_POSS*	3.4			G1	15000	114	44.7	0.808	DMA
DCPD [13]	100					G1	15000	137	52.6	0.892	DMA
DCPD [13]	98.86			*3NB_POSS*	1.14	G1	15000	144	26	0.836	DMA
DCPD [13]	97.48			*3NB_POSS*	2.52	G1	15000	137	42.5	0.758	DMA
DCPD [14]	100					G1	6000	136.4	37.5		DMA
DCPD [15]	100					G1	2000	142	52.4	1.77	DMA
DCPD [16]	100					G2	5000	171	53		DSC

DCPD[17]	100					G2	125424	148			DSC
DCPD[17]	96.04	*CPD*	3.96			G2	125424	142			DSC
DCPD[17]	84.85	*CPD*	15.15			G2	125424	158			DSC
DCPD[9]	100					G2	10000	166			DMA
DCPD[9]	83			*s_c_ester*	17	G2	10000	182			DMA
DCPD[9]	83			*s_c_amide*	17	G2	10000	185			DMA
DCPD[9]	78			*l_c_ester*	22	G2	10000	168			DMA
DCPD[9]	69			*tri_arom_ester*	31	G2	10000	178			DMA
DCPD[9]	68			*s_c_ester*	32	G2	10000	200			DMA
DCPD[9]	56			*s_c_ester*	44	G2	10000	219			DMA

[a] M is monomer, CoM is comonomer, CL is crosslinker, C is catalyst, M:C is monomer to catalyst ratio, collection described Tg characterization method (DMA or DSC).

Table S2: Additional experimental data used in model training.

Deg_CM	Deg_CL	NonDeg_CM	NonDeg_CL	Base	Deg_CM_%	Deg_CL_%	NonDeg_CL_%	NonDeg_CM_%	Base_%	CatalystGen	Catalyst_monomer	Tg_C	YieldStress_MPa	YoungsModulus_GPa
				DCPD					100	G1	100000	118		
				DCPD					100	G1	5000	145	54.8	1.851
				DCPD					100	G1	3000	134		
		ENB		DCPD				25	75	G1	5000	132	52.7	1.931
		ENB		DCPD				50	50	G1	5000	119	52.6	2.004
		ENB		DCPD				75	25	G1	5000	108	50.6	1.972
		ENB		DCPD				90	10	G1	5000	102	49.5	1.974
			HDMN	DCPD			5		95	G1	3000	144		
			HDMN	DCPD			10		90	G1	3000	153	54.2	1.871
			HDMN	DCPD			20		80	G1	3000	170	56.5	1.985
			HDMN	DCPD			30		70	G1	3000	181	58.1	2.044
			HDMN	DCPD			40		60	G1	3000	187		2.123
				DCPD					100	G2	5000	163.3		
				DCPD					100	G2	10000	143	52	1.8
		NBOH		DCPD				15	85	G2	10000	142	55	1.9
		NBOH		DCPD				50	50	G2	10000	143	56	1.9
				DCPD					100	G1	15000	129	60.5	0.93
			1NB_POSS	DCPD				1.54	98.46	G1	15000	128	55.1	0.87
			1NB_POSS	DCPD				3.4	96.6	G1	15000	114	44.7	0.808
				DCPD					100	G1	15000	137	52.6	0.892
		3NB_POSS		DCPD		1.14			74	G1	15000	144	26	0.836
		3NB_POSS		DCPD		2.52			57.5	G1	15000	137	42.5	0.758
				DCPD					100	G1	6000	136.4	37.5	
				DCPD					100	G1	2000	142	52.4	1.77
				DCPD					100	G2	5000	171	53	
				DCPD					100	G2	125424	148		
		CPD		DCPD				3.96	96.04	G2	125424	142		
		CPD		DCPD				15.15	84.85	G2	125424	158		
				DCPD					100	G2	3140	166		0.981
iPrSi				DCPD	6.24				93.76	G2	3140	138		0.988
iPrSi				DCPD	13.03				86.97	G2	3140	51		0.869
iPrSi				DCPD	22.78				77.22	G2	3140	46		0.161
iPrSi				DCPD	37.46				62.54	G2	3140	14		0.0571
				DCPD					100	G2	10000	166		
MeSi				DCPD	12				88	G2	3086	126		
EtSi				DCPD	14				86	G2	3023	119		
PhSi				DCPD	19				81	G2	2826	136		
MeSi				DCPD	6				94	G2	3116	146		
EtSi				DCPD	7				93	G2	3084	146		
iPrSi	DNSiPr			DCPD	14	17			69	G2	2640	143		
SpiroSi-8,8	SpiroSi-8,8			DCPD	5	5			95	G2	3080	155		
SpiroSi-8,8	SpiroSi-8,8			DCPD	8	8			92	G2	3036	147		
SpiroSi-8,8	SpiroSi-8,8			DCPD	15	15			85	G2	2803	153		
NbMeSi	NbMeSi			DCPD	17	17			83	G2	2888	174		
NbMeSi	NbMeSi			DCPD	32	32			68	G2	2669	180		
iPrAc-7				DCPD	11				89	G2	3123	104		
iPrAc-7				DCPD	21				79	G2	3100	61		
iPrSi-7				DCPD	7				93	G2	3068	147		
iPrSi-7				DCPD	11				89	G2	3030	137		
iPrSi-7				DCPD	14				86	G2	2992	126		
iPrSi-7				DCPD	27				73	G2	2853	79		
iPrSi-7				DCPD	43				57	G2	2689	33		
iPrSi-7				DCPD	60				40	G2	2502	6		
iPrSi				DCPD	8				92	G2	3052	149		

iPrSi				DCPD	12				88	G2	3007	142		
iPrSi				DCPD	15				85	G2	2963	138		
iPrSi				DCPD	29				71	G2	2799	88		
iPrSi				DCPD	44				56	G2	2611	46		
iPrSi				DCPD	62				38	G2	2401	14		
	s_c_ester			DCPD		17			83	G2	10000	182		
	s_c_amide			DCPD		17			83	G2	10000	185		
	l_c_ester			DCPD		22			78	G2	10000	168		
	tri_arom_ester			DCPD		31			69	G2	10000	178		
	s_c_ester			DCPD		32			68	G2	10000	200		
	s_c_ester			DCPD		44			56	G2	10000	219		
iPrSi	s_c_ester	ENB		DCPD	5	25		3.8	66.2	G2	15000	213.1		1.37
iPrSi	s_c_ester	ENB		DCPD	10	20		3.8	66.2	G2	15000	171.8		1.3
	DNSM	ENB		DCPD		9.98		4.92	85.1	G2	15000	177.3		1.35
iPrSi	s_c_ester	ENB		DCPD	9.98	9.97		4.38	75.67	G2	15000	137.3		1.39
iPrSi	s_c_ester	ENB		DCPD	5	5		4.9	85.1	G2	15000	150.3		1.56
iPrSi	DDMS	ENB		DCPD	9.98	9.97		4.38	75.67	G2	15000	144.7		1.35
	TMDS	ENB		DCPD		14.953		4.654	80.393	G2	15000	168.3		1.26
		ENB		DCPD	0			5.472285	94.52772	G2	41659.45	162.7467		
iPrSi		ENB		DCPD	2.559726			5.332209	92.10806	G2	40998.57	143.45		
iPrSi		ENB		DCPD	4.995897			5.198895	89.80521	G2	40388.78	126.0867		
iPrSi		ENB		DCPD	7.469422			5.063537	87.46704	G2	39787.92	109.01		
iPrSi		ENB		DCPD	9.939859			4.928347	85.13179	G2	39205.4	93.75333		
iPrSi		ENB		DCPD	15.00507			4.651165	80.34377	G2	38062.81	65.49		
iPrSi		ENB		DCPD	19.86881			4.385007	75.74618	G2	37026.64	36.65667		
iPrSi		ENB		DCPD	24.93915			4.107543	70.9533	G2	36004.86	22.46		
iPrSi-7		ENB		DCPD	2.498523			5.335558	92.16592	G2	41122.02	143.36		
iPrSi-7		ENB		DCPD	5.057237			5.195538	89.74722	G2	40585.82	125.0267		
iPrSi-7		ENB		DCPD	7.569194			5.058077	87.37273	G2	40072.85	107.6133		
iPrSi-7		ENB		DCPD	9.849679			4.933282	85.21704	G2	39618.26	92.59		
iPrSi-7		ENB		DCPD	14.71369			4.66711	80.6192	G2	38682.3	66.31333		
iPrSi-7		ENB		DCPD	19.87067			4.384905	75.74442	G2	37737.09	37.35333		
iPrSi-7		ENB		DCPD	24.83896			4.113026	71.04801	G2	36869.14	17.65667		
PhSi		ENB		DCPD	2.459009			5.337721	92.20327	G2	40620.32	139.55		
PhSi		ENB		DCPD	5.013697			5.197921	89.78838	G2	39594.26	119.5767		
PhSi		ENB		DCPD	7.441912			5.065042	87.49305	G2	38665.93	103.4333		
PhSi		ENB		DCPD	14.92565			4.655511	80.41884	G2	36060.2	88.24333		

PhSi		ENB		DCPD	9.98923			4.925646	85.08512	G2		37737.73	62.71667	
PhSi		ENB		DCPD	14.92565			4.655511	80.41884	G2		36060.2	35.98667	
PhSi		ENB		DCPD	24.99054			4.104731	70.90473	G2		33063.51	28.72667	
iPrAc-7		ENB		DCPD	2.469778			5.337131	92.19309	G2		41576.26	139.06	
iPrAc-7		ENB		DCPD	5.015603			5.197817	89.78658	G2		41490.85	119.2467	
iPrAc-7		ENB		DCPD	7.500068			5.06186	87.43807	G2		41407.84	98.84333	
iPrAc-7		ENB		DCPD	9.975206			4.926413	85.09838	G2		41325.47	84.58667	
iPrAc-7		ENB		DCPD	14.99853			4.651522	80.34994	G2		41159.31	58.30333	
iPrAc-7		ENB		DCPD	19.99818			4.377927	75.62389	G2		40995.25	31.78333	
iPrAc-7		ENB		DCPD	24.82171			4.11397	71.06432	G2		40838.2	17.21667	

[a] M is monomer, CoM is comonomer, CL is crosslinker, C is catalyst, M:C is monomer to catalyst ratio

Table S3. SMILES of comonomer structures used in experimental validation and encoding scheme.

Molecule	SMILES	Deg_backbone	Deg_CL	NonDeg_CL	NonDeg_CM	Non Deg
DCPD	C12C=CCC1C3CC2C=C3	0	0	0		1
ENB	C/C=C1CC2C=CC\1C2	0	0	0	1	1
1NB_POSS	CC(C)C[Si]1(O[Si](O[Si](O[Si](O1)(CC(C)C)O2)(CC(C)C)O3)(CCC4CC5C=CC4C5)O6)O[Si]7(CC(C)C)O[Si]2(CC(C)C)O[Si]3(CC(C)C)O[Si]6(CC(C)C)O7	0	0	0	1	1
3NB_POSS	CC(C)C[Si]1(OC(C)(CCC2CC3C=CC2C3)C)O[Si]4(CC(C)C)O[Si]5(CC(C)C)O[Si](O[Si](O[Si](O1)(CC(C)C)O5)(CC(C)C)OC(C)(CCC6CC7C=CC6C7)C)(CC(C)C)O[Si](OC(C)(CCC8CC9C=CC8C9)C)(CC(C)C)O4	0	1	0		0
NBOH	OCC1CC2CC1C=C2	0	0	0		1
CPD	C1=CC=CC1	0	0	0		1
iPrSi	CC(C)[Si]1(C(C)C)OC/C=C\CCO1	1	0	0		0
HDMN	C12CC(C3C2C4CC3C=C4)C=C1	0	0	0		1
DCPD_CO2Me_V	O=C(OC)C(C1)=CC2C1C3C=CC2C3	0	0	0		1
DCPD_OH_A	OC1C=CC2C1C3C=CC2C3	0	0	0		1
DCPD_OMe_A	COC1C=CC2C1C3C=CC2C3	0	0	0		1
DCPD_OBz_A	O=C(C1=CC=CC=C1)OC2C=CC3C2C4C=CC3C4	0	0	0		1
DCPD_OPr_A	CCCOC1C=CC2C1C3C=CC2C3	0	0	0		1
DCPD_OOc_A	CCCCCCCCOC1C=CC2C1C3C=CC2C3	0	0	0		1
DCPD_OAc_A	O=C(C)OC1C=CC2C1C3C=CC2C3	0	0	0		1
tri_arom_ester	O=C(OCC1CC2C=CC1C2)C3=CC(C(OCC4CC5C=CC4C5)=O)=CC(C(OCC6C7C=CC(C7)C6)=O)=C3	0	1	0		0
s_c_ester	O=C(C1C2C=CC(C2)C1)OCC3CC4C=CC3C4	0	1	0		0
s_c_amide	O=C(C1C2C=CC(C2)C1)NCC3CC4C=CC3C4	0	1	0		0
l_c_ester	O=C(CCC(OCC1CC2C=CC1C2)=O)OCC3CC4C=CC3C4	0	1	0		0
DDMS	C[Si](C)(OC1C=CC2[C@H]3C=C[C@@H](C3)C21)OC4C=CC5[C@H]6C=C[C@@H](C6)C54	0	1	0		0
TMDS	C[Si](C)(O[Si](C)(C)CCC1CC2C=CC1C2)CCC3CC4C=CC3C4	0	1	0		0
DNSM	C[Si](OCC1CC2C=CC1C2)(C)OCC3CC4C=CC3C4	0	1	0		0
MeSi	C[Si]1(C)OC/C=C\CCO1	1	0	0		0
EtSi	CC[Si]1(CC)OC/C=C\CCO1	1	0	0		0
PhSi	C1([Si]2(OC/C=C\CCO2)C3=CC=CC=C3)=CC=CC=C1	1	0	0		0

DNSiPr	CC(C)[Si](OCC1CC2C=CC1C2)(C(C)C)OCC3CC4C=CC3C4	0	1	0		0
iPrSi-7	CC(C)[Si]1(C(C)C)OCC=CCO1	1	0	0		0
iPrAc-7	CC(C)C1OCC=CCO1	1	0	0		0
SpiroSi-8,8	C1=C\CCO[Si]2(OCC/C=C\CO2)OC/1	1	1	0		0
NbMeSi	C[Si]1(CC2C3C=CC(C3)C2)OCC/C=C\CO1	1	1	0		0
PhSi-7	C1([Si]2(C3=CC=CC=C3)OCC=CCO2)=CC=CC=C1	1	0	0		0

Table S4. Predicted and experimentally measured glass transition temperatures.

pDCPD Formulation	Predicted T_g (°C)	Experimental T_g (°C)
0% **iPrSi8**, 10% **DDMS**	170.64 ± 19.86	183.62 ± 2.51
10% **iPrSi8**, 10% **DDMS**	150.65 ± 11.91	144.53 ± 1.70
10% **iPrSi8**, 20% **DDMS**	158.06 ± 17.13	151.77 ± 6.25
20% **iPrSi8**, 20% **DDMS**	133.87 ± 19.81	125.92 ± 2.53
20% **iPrSi8**, 10% **DDMS**	127.56 ± 18.04	97.99 ± 3.85
10% **LinF7**	129.5 ± 5.7	143.7 ± 0.93
10% **PhSi7**	121.2 ± 4.45	127.2 ± 1.75
10% **LinF7**, 10% **DDMS**	174.21 ± 9.89	165.86 ± 3.54
10% **PhSi7**, 10% **DDMS**	166.25 ± 6.22	138.7 ± 2.84
10% **LinF7**, 20% **DDMS**	179.8 ± 9.0 °C	169.7 ± 1.1 °C
10% **PhSi7**, 20% **DDMS**	173.5 ± 6.0 °C	153.2 ± 3.1 °C

Table S5. SMILES and classification of potential novel silyl ether comonomers.

Molecule	SMILES	Deg_CM	Deg_CL	NonDeg_CL	NonDeg_CM
cm_iPr_iPr_8	[Si]1(OC/C=C\CCO1)(C(C)C)C(C)C	1	0	0	0
cm_Me_Me_8	[Si]1(OC/C=C\CCO1)(C)C	1	0	0	0
cm_H_Me_8	[SiH]1(OC/C=C\CCO1)C	1	0	0	0
cm_chloromethyl_Me_8	[Si]1(OC/C=C\CCO1)(CCl)C	1	0	0	0
cm_Et_Et_8	[Si]1(OC/C=C\CCO1)(CC)CC	1	0	0	0
cm_Me_Pr_8	[Si]1(OC/C=C\CCO1)(C)CCC	1	0	0	0
cm_tBu_tBu_8	[Si]1(OC/C=C\CCO1)(C(C)(C)C)C(C)(C)C	1	0	0	0
cm_octyl_octyl_8	[Si]1(OC/C=C\CCO1)(CCCCCCCC)CCCCCCCC	1	0	0	0
cm_Me_Ph_8	[Si]1(OC/C=C\CCO1)(C)C2=CC=CC=C2	1	0	0	0
cm_Ph_Ph_8	[Si]1(OC/C=C\CCO1)(C2=CC=CC=C2)C3=CC=CC=C3	1	0	0	0
cm_cyclobutane_8	[Si]1(OC/C=C\CCO1)2CCC2	1	0	0	0
cm_phenethyl_Me_8	[Si]1(OC/C=C\CCO1)(C)CCC2=CC=CC=C2	1	0	0	0
cm_H_H_8	[SiH2]1OC/C=C\CCO1	1	0	0	0

cm_2-methyl-2-phenylethyl_Me_8	[Si]1(OC/C=C\CCO1)(C)CC(C)C2=CC=CC=C2	1	0	0	0
cm_hexyl_hexyl_8	[Si]1(OC/C=C\CCO1)(CCCCCC)CCCCCC	1	0	0	0
cm_p-methylphenethyl_Me_8	[Si]1(OC/C=C\CCO1)(C)CCC3=CC=C(C)C=C3	1	0	0	0
cm_Ph_Et_8	[Si]1(OC/C=C\CCO1)(CC)C2=CC=CC=C2	1	0	0	0
cm_triphenylmethyl_Me_8	[Si]1(OC/C=C\CCO1)(C)C(C3=CC=CC=C3)(C4=CC=CC=C4)C2=CC=CC=C2	1	0	0	0
cm_3_3-dimethylbutyl_Me_8	[Si]1(OC/C=C\CCO1)(C)CCC(C)(C)C	1	0	0	0
cm_tBu_Me_8	[Si]1(OC/C=C\CCO1)(C)(C(C)(C)C)	1	0	0	0
cm_cyclopentyl_cyclopentyl_8	[Si]1(OC/C=C\CCO1)(C2CCCC2)C3CCCC3	1	0	0	0
cm_cyclohexyl_cyclohexyl_8	[Si]1(OC/C=C\CCO1)(C2CCCCC2)C3CCCCC3	1	0	0	0
cm_cyclohexyl_Me_8	[Si]1(OC/C=C\CCO1)(C)C2CCCCC2	1	0	0	0
cm_decyl_Me_8	[Si]1(OC/C=C\CCO1)(C)CCCCCCCCCC	1	0	0	0
cm_docosyl_Me_8	[Si]1(OC/C=C\CCO1)(C)CCCCCCCCCCCCCCCCCCCCCC	1	0	0	0
cm_3-p-methoxyphenylpropyl_Me_8	[Si]1(OC/C=C\CCO1)(C)CCCC2=CC=C(OC)C=C2	1	0	0	0
cm_octyl_Me_8	[Si]1(OC/C=C\CCO1)(C)CCCCCCCC	1	0	0	0
cm_heptyl_Me_8	[Si]1(OC/C=C\CCO1)(C)CCCCCCC	1	0	0	0
cm_dodecyl_Me_8	[Si]1(OC/C=C\CCO1)(C)CCCCCCCCCCCC	1	0	0	0
cm_3-phenoxypropyl_Me_8	[Si]1(OC/C=C\CCO1)(C)CCCOC2=CC=CC=C2	1	0	0	0
cm_octadecyl_methoxy_8	[Si]1(OC/C=C\CCO1)(OC)CCCCCCCCCCCCCCCCCC	1	0	0	0
cm_m-phenoxyphenyl_Me_8	[Si]1(OC/C=C\CCO1)(C)C2=CC(OC3=CC=CC=C3)=CC=C2	1	0	0	0
cm_9-fluorenyl_Me_8	[Si]1(OC/C=C\CCO1)(C)C2C(C=CC=C3)=C3C4=C2C=CC=C4	1	0	0	0
cm_dichloromethyl_Me_8	[Si]1(OC/C=C\CCO1)(C)C(Cl)Cl	1	0	0	0
cm_cyclopentane_8	[Si]1(OC/C=C\CCO1)2CCCC2	1	0	0	0
cm_iPr_Me_8	[Si]1(OC/C=C\CCO1)(C)C(C)C	1	0	0	0
cm_H_iBu_8	[SiH]1(OC/C=C\CCO1)CC(C)C	1	0	0	0
cm_3_3_3-trifluoropropyl_Me_8	[Si]1(OC/C=C\CCO1)(C)CCC(F)(F)F	1	0	0	0
cm_clohexane_8	[Si]1(OC/C=C\CCO1)2CCCCC2	1	0	0	0
cm_H_hexyl_8	[SiH]1(OC/C=C\CCO1)CCCCCC	1	0	0	0
cm_p-toyl_Me_8	[Si]1(OC/C=C\CCO1)(C)C2=CC=C(C)C=C2	1	0	0	0
cm_benzyloxy_benzyloxy_8	[Si]1(OC/C=C\CCO1)(OCC2=CC=CC=C2)OCC3=CC=CC=C3	1	0	0	0
cm_3-phenylpropyl_Me_8	[Si]1(OC/C=C\CCO1)(C)CCCC2=CC=CC=C2	1	0	0	0
cm_mesityl_mesityl_8	[Si]1(OC/C=C\CCO1)(C2=C(C)C=C(C)C=C2C)C3=C(C)C=C(C)C=C3C	1	0	0	0
cm_ethyoxy_ethyoxy_8	[Si]1(OC/C=C\CCO1)(OCC)OCC	1	0	0	0

cm_2-ethylhexyl_2-ethylhexyl_8	[Si]1(OC/C=C\CCO1)(CC(CC)CCCC)CC(CC)CCCC	1	0	0	0
cm_3-chloropropyl_Me_8	[Si]1(OC/C=C\CCO1)(C)CCCCl	1	0	0	0
cm_trimethylsilylmethyl_trimethylsilylmethyl_8	[Si]1(OC/C=C\CCO1)(C[Si](C)(C)C)C[Si](C)(C)C	1	0	0	0
cm_3-chlroisobutyl_Me_8	[Si]1(OC/C=C\CCO1)(C)CC(C)CCl	1	0	0	0
cm_2-chloroethyl_Me_8	[Si]1(OC/C=C\CCO1)(C)CCCl	1	0	0	0
cm_3-chloropropyl_3-chlrorpropyl_8	[Si]1(OC/C=C\CCO1)(CCCCl)CCCCl	1	0	0	0
cm_tBu_Ph_8	[Si]1(OC/C=C\CCO1)(C2=CC=CC=C2)C(C)(C)C	1	0	0	0
cm_H_tBu_8	[SiH]1(OC/C=C\CCO1)C(C)(C)C	1	0	0	0
cm_dodecyl_dodecyl_8	[Si]1(OC/C=C\CCO1)(CCCCCCCCCCCC)CCCCCCCCCCCC	1	0	0	0
cm_H_Et_8	[SiH]1(OC/C=C\CCO1)CC	1	0	0	0
cm_Bu_Bu_8	[Si]1(OC/C=C\CCO1)(CCCC)CCCC	1	0	0	0
cm_p-tolyl_p-tolyl_8	[Si]1(OC/C=C\CCO1)(C2=CC=C(C)C=C2)C3=CC=C(C)C=C3	1	0	0	0
cm_Et_Me_8	[Si]1(OC/C=C\CCO1)(C)CC	1	0	0	0
cm_4-phenylbityl_Me_8	[Si]1(OC/C=C\CCO1)(C)CCCCC2=CC=CC=C2	1	0	0	0
cm_Bu_Me_8	[Si]1(OC/C=C\CCO1)(C)CCCC	1	0	0	0
cm_hexyl_Me_8	[Si]1(OC/C=C\CCO1)(C)CCCCCC	1	0	0	0
cm_octadecyl_Me_8	[Si]1(OC/C=C\CCO1)(C)CCCCCCCCCCCCCCCCCC	1	0	0	0
cm_trimethylsiloxy_trimethylsiloxy_8	[Si]1(OC/C=C\CCO1)(O[Si](C)(C)C)O[Si](C)(C)C	1	0	0	0
cm_H_Ph_8	[SiH]1(OC/C=C\CCO1)C2=CC=CC=C2	1	0	0	0
cm_pentyl_Me_8	[Si]1(OC/C=C\CCO1)(C)CCCCC	1	0	0	0
cm_chlorophenyl_Me_8	[Si]1(OC/C=C\CCO1)(C)C2=CC=C(Cl)C=C2	1	0	0	0
cm_heptadecafluror-1_1_2_2-tetrahydrodecyl_Me_8	[Si]1(OC/C=C\CCO1)(C)CCC(F)(F)C(F)(F)C(F)(F)C(F)(F)C(F)(F)C(F)(F)C(F)(F)C(F)(F)F	1	0	0	0
cm_3-heptafluoroisopropoxypropyl_Me_8	[Si]1(OC/C=C\CCO1)(C)CCCOC(F)(C(F)(F)F)C(F)(F)F	1	0	0	0
cm_nonfluorohexyl_nonfluorohexyl_8	[Si]1(OC/C=C\CCO1)(CCC(F)(F)C(F)(F)C(F)(F)C(F)(F)F)CCC(F)(F)C(F)(F)C(F)(F)C(F)(F)F	1	0	0	0
cm_nonafluorohexyl_Me_8	[Si]1(OC/C=C\CCO1)(C)CCC(F)(F)C(F)(F)C(F)(F)C(F)(F)F	1	0	0	0
cm_tridecafluoro-1_1_2_2-tetrahydroocytyl_Me_8	[Si]1(OC/C=C\CCO1)(C)CCC(F)(F)C(F)(F)C(F)(F)C(F)(F)C(F)(F)C(F)(F)F	1	0	0	0
cm_pentafluorophenylpropyl_Me_8	[Si]1(OC/C=C\CCO1)(C)CCCC2=C(F)C(F)=C(F)C(F)=C2F	1	0	0	0
cm_iPr_iPr_7	[Si]1(OC/C=C\CO1)(C(C)C)C(C)C	1	0	0	0
cm_Me_Me_7	[Si]1(OC/C=C\CO1)(C)C	1	0	0	0
cm_H_Me_7	[SiH]1(OC/C=C\CO1)C	1	0	0	0
cm_chloromethyl_Me_7	[Si]1(OC/C=C\CO1)(CCl)C	1	0	0	0

cm_Et_Et_7	[Si]1(OC/C=C\CO1)(CC)CC	1	0	0	0
cm_Me_Pr_7	[Si]1(OC/C=C\CO1)(C)CCC	1	0	0	0
cm_tBu_tBu_7	[Si]1(OC/C=C\CO1)(C(C)(C)C)C(C)(C)C	1	0	0	0
cm_octyl_octyl_7	[Si]1(OC/C=C\CO1)(CCCCCCCC)CCCCCCCC	1	0	0	0
cm_Me_Ph_7	[Si]1(OC/C=C\CO1)(C)C2=CC=CC=C2	1	0	0	0
cm_Ph_Ph_7	[Si]1(OC/C=C\CO1)(C2=CC=CC=C2)C3=CC=CC=C3	1	0	0	0
cm_cyclobutane_7	[Si]1(OC/C=C\CO1)2CCC2	1	0	0	0
cm_phenethyl_Me_7	[Si]1(OC/C=C\CO1)(C)CCC2=CC=CC=C2	1	0	0	0
cm_H_H_7	[SiH2]1OC/C=C\CO1	1	0	0	0
cm_2-methyl-2-phenylethyl_Me_7	[Si]1(OC/C=C\CO1)(C)CC(C)C2=CC=CC=C2	1	0	0	0
cm_hexyl_hexyl_7	[Si]1(OC/C=C\CO1)(CCCCCC)CCCCCC	1	0	0	0
cm_p-methylphenethyl_Me_7	[Si]1(OC/C=C\CO1)(C)CCC3=CC=C(C)C=C3	1	0	0	0
cm_Ph_Et_7	[Si]1(OC/C=C\CO1)(CC)C2=CC=CC=C2	1	0	0	0
cm_triphenylmethyl_Me_7	[Si]1(OC/C=C\CO1)(C)C(C3=CC=CC=C3)(C4=CC=CC=C4)C2=CC=CC=C2	1	0	0	0
cm_3_3-dimethylbutyl_Me_7	[Si]1(OC/C=C\CO1)(C)CCC(C)(C)C	1	0	0	0
cm_tBu_Me_7	[Si]1(OC/C=C\CO1)(C)(C(C)(C)C)	1	0	0	0
cm_cyclopentyl_cyclopentyl_7	[Si]1(OC/C=C\CO1)(C2CCCC2)C3CCCC3	1	0	0	0
cm_cyclohexyl_cyclohexyl_7	[Si]1(OC/C=C\CO1)(C2CCCCC2)C3CCCCC3	1	0	0	0
cm_cyclohexyl_Me_7	[Si]1(OC/C=C\CO1)(C)C2CCCCC2	1	0	0	0
cm_decyl_Me_7	[Si]1(OC/C=C\CO1)(C)CCCCCCCCCC	1	0	0	0
cm_docosyl_Me_7	[Si]1(OC/C=C\CO1)(C)CCCCCCCCCCCCCCCCCCCCCC	1	0	0	0
cm_3-p-methoxyphenylpropyl_Me_7	[Si]1(OC/C=C\CO1)(C)CCCC2=CC=C(OC)C=C2	1	0	0	0
cm_octyl_Me_7	[Si]1(OC/C=C\CO1)(C)CCCCCCCC	1	0	0	0
cm_heptyl_Me_7	[Si]1(OC/C=C\CO1)(C)CCCCCCC	1	0	0	0
cm_dodecyl_Me_7	[Si]1(OC/C=C\CO1)(C)CCCCCCCCCCCC	1	0	0	0
cm_3-phenoxypropyl_Me_7	[Si]1(OC/C=C\CO1)(C)CCCOC2=CC=CC=C2	1	0	0	0
cm_octadecyl_methoxy_7	[Si]1(OC/C=C\CO1)(OC)CCCCCCCCCCCCCCCCCC	1	0	0	0
cm_m-phenoxyphenyl_Me_7	[Si]1(OC/C=C\CO1)(C)C2=CC(OC3=CC=CC=C3)=CC=C2	1	0	0	0
cm_9-fluorenyl_Me_7	[Si]1(OC/C=C\CO1)(C)C2C(C=CC=C3)=C3C4=C2C=CC=C4	1	0	0	0
cm_dichloromethyl_Me_7	[Si]1(OC/C=C\CO1)(C)C(Cl)Cl	1	0	0	0
cm_cyclopentane_7	[Si]1(OC/C=C\CO1)2CCCC2	1	0	0	0
cm_iPr_Me_7	[Si]1(OC/C=C\CO1)(C)C(C)C	1	0	0	0

cm_H_iBu_7	[SiH]1(OC/C=C\CO1)CC(C)C	1	0	0	0
cm_3_3_3-trifluoropropyl_Me_7	[Si]1(OC/C=C\CO1)(C)CCC(F)(F)F	1	0	0	0
cm_clohexane_7	[Si]1(OC/C=C\CO1)2CCCCC2	1	0	0	0
cm_H_hexyl_7	[SiH]1(OC/C=C\CO1)CCCCCC	1	0	0	0
cm_p-toyl_Me_7	[Si]1(OC/C=C\CO1)(C)C2=CC=C(C)C=C2	1	0	0	0
cm_benzyloxy_benzyloxy_7	[Si]1(OC/C=C\CO1)(OCC2=CC=CC=C2)OCC3=CC=CC=C3	1	0	0	0
cm_3-phenylpropyl_Me_7	[Si]1(OC/C=C\CO1)(C)CCCC2=CC=CC=C2	1	0	0	0
cm_mesityl_mesityl_7	[Si]1(OC/C=C\CO1)(C2=C(C)C=C(C)C=C2C)C3=C(C)C=C(C)C=C3C	1	0	0	0
cm_ethyoxy_ethyoxy_7	[Si]1(OC/C=C\CO1)(OCC)OCC	1	0	0	0
cm_2-ethylhexyl_2-ethylhexyl_7	[Si]1(OC/C=C\CO1)(CC(CC)CCCC)CC(CC)CCCC	1	0	0	0
cm_3-chloropropyl_Me_7	[Si]1(OC/C=C\CO1)(C)CCCCl	1	0	0	0
cm_trimethylsilylmethyl_trimethylsilylmethyl_7	[Si]1(OC/C=C\CO1)(C[Si](C)(C)C)C[Si](C)(C)C	1	0	0	0
cm_3-chlroisobutyl_Me_7	[Si]1(OC/C=C\CO1)(C)CC(C)CCl	1	0	0	0
cm_2-chloroethyl_Me_7	[Si]1(OC/C=C\CO1)(C)CCCl	1	0	0	0
cm_3-chloropropyl_3-chlrorpropyl_7	[Si]1(OC/C=C\CO1)(CCCCl)CCCCl	1	0	0	0
cm_tBu_Ph_7	[Si]1(OC/C=C\CO1)(C2=CC=CC=C2)C(C)(C)C	1	0	0	0
cm_H_tBu_7	[SiH]1(OC/C=C\CO1)C(C)(C)C	1	0	0	0
cm_dodecyl_dodecyl_7	[Si]1(OC/C=C\CO1)(CCCCCCCCCCCC)CCCCCCCCCCCC	1	0	0	0
cm_H_Et_7	[SiH]1(OC/C=C\CO1)CC	1	0	0	0
cm_Bu_Bu_7	[Si]1(OC/C=C\CO1)(CCCC)CCCC	1	0	0	0
cm_p-tolyl_p-tolyl_7	[Si]1(OC/C=C\CO1)(C2=CC=C(C)C=C2)C3=CC=C(C)C=C3	1	0	0	0
cm_Et_Me_7	[Si]1(OC/C=C\CO1)(C)CC	1	0	0	0
cm_4-phenylbityl_Me_7	[Si]1(OC/C=C\CO1)(C)CCCCC2=CC=CC=C2	1	0	0	0
cm_Bu_Me_7	[Si]1(OC/C=C\CO1)(C)CCCC	1	0	0	0
cm_hexyl_Me_7	[Si]1(OC/C=C\CO1)(C)CCCCCC	1	0	0	0
cm_octadecyl_Me_7	[Si]1(OC/C=C\CO1)(C)CCCCCCCCCCCCCCCCCC	1	0	0	0
cm_trimethylsiloxy_trimethylsiloxy_7	[Si]1(OC/C=C\CO1)(O[Si](C)(C)C)O[Si](C)(C)C	1	0	0	0
cm_H_Ph_7	[SiH]1(OC/C=C\CO1)C2=CC=CC=C2	1	0	0	0
cm_pentyl_Me_7	[Si]1(OC/C=C\CO1)(C)CCCCC	1	0	0	0
cm_chlorophenyl_Me_7	[Si]1(OC/C=C\CO1)(C)C2=CC=C(Cl)C=C2	1	0	0	0
cm_heptadecafluror-1_1_2_2-tetrahydrodecyl_Me_7	[Si]1(OC/C=C\CO1)(C)CCC(F)(F)C(F)(F)C(F)(F)C(F)(F)C(F)(F)C(F)(F)C(F)(F)C(F)(F)F	1	0	0	0

cm_3-heptafluoroisopropoxypropyl_Me_7	[Si]1(OC/C=C\CO1)(C)CCCOC(F)(C(F)(F)F)C(F)(F)F	1	0	0	0
cm_nonfluorohexyl_nonfluorohexyl_7	[Si]1(OC/C=C\CO1)(CCC(F)(F)C(F)(F)C(F)(F)C(F)(F)F)CCC(F)(F)C(F)(F)C(F)(F)C(F)(F)F	1	0	0	0
cm_nonafluorohexyl_Me_7	[Si]1(OC/C=C\CO1)(C)CCC(F)(F)C(F)(F)C(F)(F)C(F)(F)F	1	0	0	0
cm_tridecafluoro-1_1_2_2-tetrahydroocytyl_Me_7	[Si]1(OC/C=C\CO1)(C)CCC(F)(F)C(F)(F)C(F)(F)C(F)(F)C(F)(F)C(F)(F)F	1	0	0	0
cm_pentafluorophenylpropyl_Me_7	[Si]1(OC/C=C\CO1)(C)CCCC2=C(F)C(F)=C(F)C(F)=C2F	1	0	0	0
cm_cl_Nb_Nb_8	C1(C2)CC([Si]3(C4C(C5)C=CC5C4)OC/C=C\CCO3)C2C=C1	1	1	0	0
cm_cl_Nb_MeNb_8	C1(C2)CC(C[Si]3(C4C(C5)C=CC5C4)OC/C=C\CCO3)C2C=C1	1	1	0	0
cm_corsslinker_MeNb_MeNb_8	C1(C2)CC(C[Si]3(CC4C(C5)C=CC5C4)OC/C=C\CCO3)C2C=C1	1	1	0	0
cm_cl_OMeNb_Ph_8	C1(C2)C(CO[Si]3(C4=CC=CC=C4)OC/C=C\CCO3)CC2C=C1	1	1	0	0
cm_cl_Mesily_Ph_Mesily_8_8	C[Si]1(C2=CC=C([Si]3(OCC/C=C\CO3)C)C=C2)OC/C=C\CCO1	1	1	0	0

References:

(1) Shieh, P.; Nguyen, H. V.-T.; Johnson, J. A. Tailored Silyl Ether Monomers Enable Backbone-Degradable Polynorbornene-Based Linear, Bottlebrush and Star Copolymers through ROMP. *Nat. Chem.* **2019**, *11* (12), 1124–1132. https://doi.org/10.1038/s41557-019-0352-4.

(2) Kotha, S.; Ravikumar, O.; Majhi, J. Synthesis of a Tricyclic Lactam via Beckmann Rearrangement and Ring-Rearrangement Metathesis as Key Steps. *Beilstein J. Org. Chem.* **2015**, *11*, 1503–1508. https://doi.org/10.3762/bjoc.11.163.

(3) Rogers, D.; Hahn, M. Extended-Connectivity Fingerprints. *J. Chem. Inf. Model.* **2010**, *50* (5), 742–754. https://doi.org/10.1021/ci100050t.

(4) Landrum, G. RDKit: Open-Source Cheminformatics Software. **2016**.

(5) Mohapatra, S.; Yang, T.; Gómez-Bombarelli, R. Reusability Report: Designing Organic Photoelectronic Molecules with Descriptor Conditional Recurrent Neural Networks. *Nat Mach Intell* **2020**, *2* (12), 749–752. https://doi.org/10.1038/s42256-020-00268-w.

(6) Schissel, C. K.; Mohapatra, S.; Wolfe, J. M.; Fadzen, C. M.; Bellovoda, K.; Wu, C.-L.; Wood, J. A.; Malmberg, A. B.; Loas, A.; Gómez-Bombarelli, R.; Pentelute, B. L. *Interpretable Deep Learning for De Novo Design of Cell-Penetrating Abiotic Polymers*; preprint; Bioinformatics, 2020. https://doi.org/10.1101/2020.04.10.036566.

(7) Lloyd, E. M.; Cooper, J. C.; Shieh, P.; Ivanoff, D. G.; Parikh, N. A.; Mejia, E. B.; Husted, K. E. L.; Costa, L. C.; Sottos, N. R.; Johnson, J. A.; Moore, J. S. Efficient Manufacture, Deconstruction, and Upcycling of High-Performance Thermosets and Composites. *ACS Appl. Eng. Mater.* **2022**, acsaenm.2c00115. https://doi.org/10.1021/acsaenm.2c00115.

(8) Shieh, P.; Zhang, W.; Husted, K. E. L.; Kristufek, S. L.; Xiong, B.; Lundberg, D. J.; Lem, J.; Veysset, D.; Sun, Y.; Nelson, K. A.; Plata, D. L.; Johnson, J. A. Cleavable Comonomers Enable Degradable, Recyclable Thermoset Plastics. *Nature* **2020**, *583* (7817), 542–547. https://doi.org/10.1038/s41586-020-2495-2.

(9) Ivanoff, D. G.; Sung, J.; Butikofer, S. M.; Moore, J. S.; Sottos, N. R. Cross-Linking Agents for Enhanced Performance of Thermosets Prepared via Frontal Ring-Opening Metathesis Polymerization. *Macromolecules* **2020**, *53* (19), 8360–8366. https://doi.org/10.1021/acs.macromol.0c01530.

(10) Long, T. R.; Elder, R. M.; Bain, E. D.; Masser, K. A.; Sirk, T. W.; Yu, J. H.; Knorr, D. B.; Lenhart, J. L. Influence of Molecular Weight between Crosslinks on the Mechanical Properties of Polymers Formed *via* Ring-Opening Metathesis. *Soft Matter* **2018**, *14* (17), 3344–3360. https://doi.org/10.1039/C7SM02407J.

(11) Saha, S.; Ginzburg, Y.; Rozenberg, I.; Iliashevsky, O.; Ben-Asuly, A.; Gabriel Lemcoff, N. Cross-Linked ROMP Polymers Based on Odourless Dicyclopentadiene Derivatives. *Polym. Chem.* **2016**, *7* (18), 3071–3075. https://doi.org/10.1039/C6PY00378H.

(12) Elder, R. M.; Long, T. R.; Bain, E. D.; Lenhart, J. L.; Sirk, T. W. Mechanics and Nanovoid Nucleation Dynamics: Effects of Polar Functionality in Glassy Polymer Networks. *Soft Matter* **2018**, *14* (44), 8895–8911. https://doi.org/10.1039/C8SM01483C.

(13) Constable, G. S.; Lesser, A. J.; Coughlin, E. B. Morphological and Mechanical Evaluation of Hybrid Organic−Inorganic Thermoset Copolymers of Dicyclopentadiene and Mono- or Tris(Norbornenyl)-Substituted Polyhedral Oligomeric Silsesquioxanes. *Macromolecules* **2004**, *37* (4), 1276–1282. https://doi.org/10.1021/ma034989w.

(14) He, Z.; Sun, J.; Zhang, L.; Wang, Y.; Ren, H.; Bao, J. B. Synergistic Reinforcing and Toughening of Polydicyclopentadiene Nanocomposites with Low Loadings VinylFunctionalized Multi-Walled Carbon Nanotubes. Polymer (Guildf). 2018, 153 (August), 287–294. https://doi.org/10.1016/j.polymer.2018.08.016.

(15) Knorr, D. B.; Masser, K. A.; Elder, R. M.; Sirk, T. W.; Hindenlang, M. D.; Yu, J. H.; Richardson, A. D.; Boyd, S. E.; Spurgeon, W. A.; Lenhart, J. L. Overcoming the Structural versus Energy Dissipation Trade-off in Highly Crosslinked Polymer Networks: Ultrahigh Strain Rate Response in Polydicyclopentadiene. Compos. Sci. Technol. 2015, 114, 17–25. https://doi.org/10.1016/j.compscitech.2015.03.021.

(16) Rohde, B. J.; Le, K. M.; Krishnamoorti, R.; Robertson, M. L. Thermoset Blends of an Epoxy Resin and Polydicyclopentadiene. Macromolecules 2016, 49 (23), 8960–8970. https://doi.org/10.1021/acs.macromol.6b01649.

(17) Vidavsky, Y.; Navon, Y.; Ginzburg, Y.; Gottlieb, M.; Gabriel Lemcoff, N. Thermal Properties of Ruthenium Alkylidene-Polymerized Dicyclopentadiene. Beilstein J. Org. Chem. 2015, 11 (Figure 1), 1469–1474. https://doi.org/10.3762/bjoc.11.159.
